# Divergent Synthesis of Cyclopropane‐Containing Lead‐Like Compounds, Fragments and Building Blocks through a Cobalt Catalyzed Cyclopropanation of Phenyl Vinyl Sulfide

**DOI:** 10.1002/ejoc.201701030

**Published:** 2017-09-11

**Authors:** Stephen J. Chawner, Manuel J. Cases‐Thomas, James A. Bull

**Affiliations:** ^1^ Department of Chemistry Imperial College London South Kensington SW7 2AZ London UK; ^2^ Lilly Research Centre Eli Lilly and Company Erl Wood Manor, Sunninghill Road GU20 6PH Windlesham UK

**Keywords:** Cyclopropanes, Sulfoxides, Small ring systems, Homogeneous catalysis, Molecular diversity

## Abstract

Cyclopropanes provide important design elements in medicinal chemistry and are widely present in drug compounds. Here we describe a strategy and extensive synthetic studies for the preparation of a diverse collection of cyclopropane‐containing lead‐like compounds, fragments and building blocks exploiting a single precursor. The bifunctional cyclopropane (*E/Z*)‐ethyl 2‐(phenylsulfanyl)‐cyclopropane‐1‐carboxylate was designed to allow derivatization through the ester and sulfide functionalities to topologically varied compounds designed to fit in desirable chemical space for drug discovery. A cobalt‐catalyzed cyclopropanation of phenyl vinyl sulfide affords these scaffolds on multigram scale. Divergent, orthogonal derivatization is achieved through hydrolysis, reduction, amidation and oxidation reactions as well as sulfoxide–magnesium exchange/functionalization. The cyclopropyl Grignard reagent formed from sulfoxide exchange is stable at 0 °C for > 2 h, which enables trapping with various electrophiles and Pd‐catalyzed Negishi cross‐coupling reactions. The library prepared, as well as a further virtual elaboration, is analyzed against parameters of lipophilicity (ALog P), M_W_ and molecular shape by using the LLAMA (Lead‐Likeness and Molecular Analysis) software, to illustrate the success in generating lead‐like compounds and fragments.

## Introduction

A limitation in examining new, challenging pharmaceutical targets is the availability of innovative, novel fragments and building blocks that possess desirable physicochemical properties, and sample new regions of chemical space.^1^ Recent years have seen a focus on smaller, more polar compounds and less planar, sp^3^‐rich derivatives containing fewer aromatic rings, perceived to be more likely to successfully progress through drug development.^2^, ^3^ New synthetic strategies and methods can enable chemical space to be probed more effectively by enriching current lead‐like and fragment compound libraries with compounds that can present new design elements and novel bond vectors.^1^, ^4^, ^5^

Late‐stage compound attrition, particularly within phase II and phase III clinical trials, is extremely costly to the drug discovery industry, with the cost of ensuring appropriate molecular properties at a much earlier stage of development being significantly lower.^6^ Hence, attractive screening collections can offer significant value. Considerable effort has been expended in developing guidelines to describe and influence compound collections, which are frequently used to aid in the development of a compound, aiming to predispose derivatives to fall into regions of desirable chemical space.[3a], 7 Relevant parameters of interest include lipophilicity (Log P), molecular weight (M_W_), number of rotatable bonds, polar surface area (PSA) and the numbers of hydrogen‐bond donors and acceptors (HBD/HBA), and consideration of these has given rise to the terms drug‐like, lead‐like and fragment to describe screening compounds.^7^, ^8^

**Figure 1:**
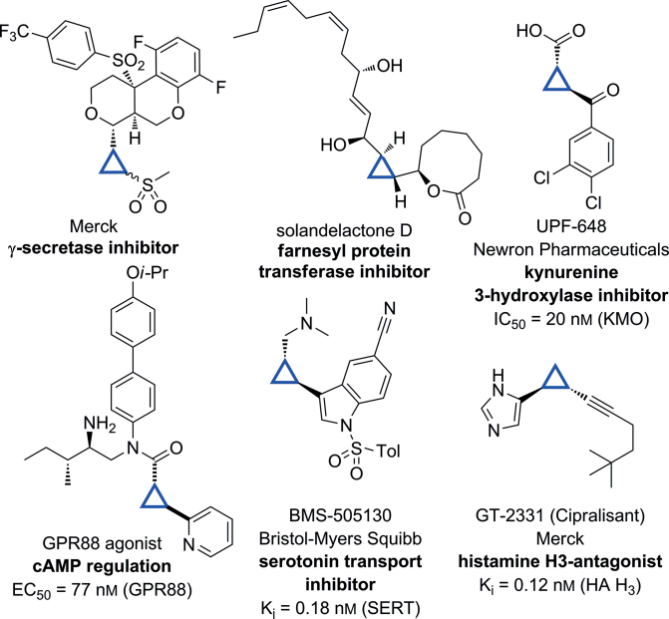
Selected cyclopropane‐containing natural products and pharmaceutical compounds.

The cyclopropane motif is highly significant in drug discovery as the 10^th^ most frequently found ring system in small molecule drugs.^9^, ^10^ It is also present in a variety of biologically active natural products and other medicinally‐important molecules (Figure 1).^11^, ^12^ Substituted cyclopropanes present a well‐defined 3‐dimensional shape, conformational rigidity, and electronic properties in between that of an alkene and a *gem*‐dimethyl group, for example, as a result of the small strained ring structure.

**Figure 2:**
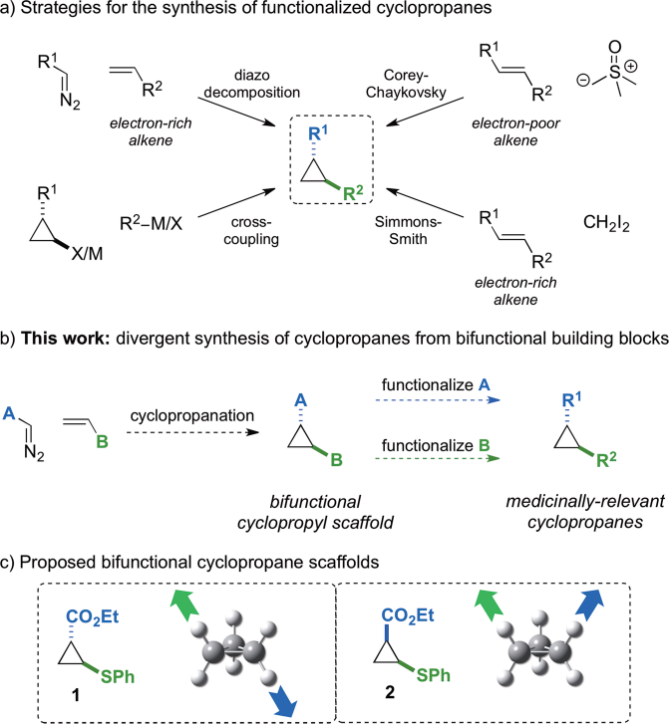
Strategies for the synthesis of cyclopropanes and designed bifunctional cyclopropane building blocks.

The synthesis of cyclopropane derivatives has been extensively investigated, exploiting numerous powerful synthetic methods (Figure 2a).^13^ Cyclopropanes bearing functional groups have been generated through Simmons–Smith cyclopropanation,^14^, ^15^ transition metal‐catalyzed carbene insertion to alkenes using diazo compounds,^16^, ^17^ and the reaction of sulfur ylides with electron deficient alkenes.^18^ Furthermore, the cross‐coupling of cyclopropyl organometallic or (pseudo)halide species has been exploited as a more divergent approach to cyclopropane derivatives through functionalization of the ring.^19^ Most recently this has been extended to include powerful C–H functionalization methods.^20^

We proposed that a divergent route to cyclopropane containing lead‐like compounds and fragments would present a valuable approach to a novel screening collection. Furthermore, this approach would provide interesting building blocks that may be more generally applicable for the construction of cyclopropane derivatives. As such we targeted small bifunctional cyclopropane‐containing scaffolds (Figure 2b).

Here we report the development of (*E*)‐ and (*Z*)‐ethyl 2‐(phenylsulfanyl)‐cyclopropane‐1‐carboxylate as cyclopropane scaffolds and extensive studies on the bidirectional functionalization. Derivatization of these scaffolds affords a collection of lead‐like and fragment compounds as well as further building blocks for the preparation of cyclopropane derivatives. Highlights include a cobalt‐catalyzed cyclopropanation of phenyl vinyl sulfide, formation of amido‐cyclopropyl sulfones, and generation of cyclopropyl Grignard reagent through sulfoxide–metal exchange followed by reaction with various electrophiles and use in Pd‐catalyzed Negishi cross‐coupling reactions. Finally, we present analysis on the physicochemical properties and molecular shape of the compounds prepared, to illustrate that these scaffolds afford medicinally relevant, non‐planar compounds that occupy desirable chemical space.

## Results and Discussion

### Scaffold Design and Hypothesis

We envisaged that a wide variety of medicinally‐relevant cyclopropane‐containing compounds could be prepared in a divergent manner from a single central bifunctional cyclopropyl scaffold. For this we required a low molecular weight scaffold that could be easily derivatized to lead‐like compounds or fragments, and functionalized in two directions, ideally through bond formation to the cyclopropane ring itself. The two scaffold functionalities should undergo derivatization orthogonally, granting access to a wide and diverse scope of functionality on the ring. We considered (*E*)‐ and (*Z*)‐ethyl 2‐(phenylsulfanyl)‐cyclopropane‐1‐carboxylates would meet these criteria, and provide suitable building blocks through functionalization of the ester or sulfide groups (Figure 2c). Especially valuable would be the potential to convert the sulfide to the sulfoxide and exploit sulfoxide–magnesium exchange to form bonds directly to the cyclopropane ring.^21^, ^22^, ^23^ Both the *E*‐ and *Z*‐diastereoisomers were of interest, possessing very different bond vectors, to increase the shape diversity of the compounds. Hence, the first objective was to prepare these building blocks on a large scale.

### Cyclopropanation of Phenyl Vinyl Sulfide

**Scheme 1:**
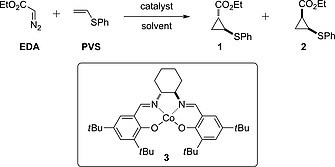
Proposed synthesis of *E*‐ and *Z*‐cyclopropyl scaffolds.

To generate cyclopropyl scaffolds **1** and **2** we envisaged the reaction of phenyl vinyl sulfide (PVS) with ethyl diazoacetate (EDA) in a transition metal‐catalyzed cyclopropanation (Scheme 1). The only prior report of the cyclopropanation of PVS with EDA was in 1962; an uncatalyzed reaction requiring heating of the neat reactants at 100 to 170 °C in a sealed vessel.^24^, ^25^, ^26^ In search of less hazardous, lower temperature reaction conditions suitable for multi‐gram scale, transition metal catalysts were investigated. Initially complexes of Pd^0^, Rh^0^, Cu^I^ and Cu^II^ were explored for catalytic activity (see the Supporting Information for further details).

From the initial screening, only Cu^I^OTf (0.5 mol‐%) promoted the reaction effectively, and a yield of up to 54 % was obtained by running the reaction in CHCl_3_ at 30 °C with a slow addition of the diazo compound. A mixture of *trans* and *cis* substituted cyclopropanes were formed in a 1:1 ratio. These were readily separated to single diastereoisomers. However, the relatively low yield and exacting practical considerations detracted from the reaction convenience and this approach was not suitable for scale‐up. Pleasingly on further investigation, Co^II^–(salen)‐type complex **3** was found to catalyze the desired cyclopropanation (Scheme 1).^17^, ^27^ The use of 5 mol‐% of the Co^II^ complex, in CH_2_Cl_2_ at 40 °C gave 40 % yield in 24 h (Table 1, entry 1). As has been observed with other Co‐catalyzed cyclopropanation reactions using diazo compounds,^17^ the reactions were facile to set up and the catalyst was easily stored and did not result in dimerization of the diazo reagent under the reaction conditions, removing the need for a slow addition protocol.

**Table 1:** Effect of solvent and reaction scale on the Co^II^‐catalyzed cyclopropanation reaction

Entry^a^	Solvent	*dr*	Yield **1** + **2**
		(*trans* **1**/*cis* **2**)^b^	[%]^c^
1	CH_2_Cl_2_	52:48	40
2	CHCl_3_	48:52	13
3	toluene	45:55	53
4	benzene	48:52	73
5	TBME	48:52	69
6	neat	46:54	93
7	H_2_O	47:53	100
8^d^	Neat	53:47^e^	70^f^
9^g^	Neat	52:48^e^	46^f^
10^d^	H_2_O	45:55^e^	85^f^
11^h^	H_2_O	46:54^e^	89^f^, ^i^

a Reaction conditions: EDA (0.3 mmol); PVS. (1.5 equiv.), catalyst **3** (5 mol‐%), solvent, 40 °C, 24 h.

b Calculated from the crude reaction mixture by ^1^H NMR unless stated otherwise.

c Yields were calculated using ^1^H NMR by comparison with an internal standard (dibenzyl ether) unless stated otherwise.

d 10 mmol scale.

e *dr* from isolated masses.

f Combined yield of separated isolated products.

g 20 mmol scale.

h 40 mmol scale.

i Corresponds to 7.9 g of product (**1** + **2**).

To optimize the promising Co^II^‐catalyzed reaction a solvent screen was conducted (Table 1). Cyclopropanes **1** and **2** were observed under each set of conditions tested (Entries 1–7), but the reaction was most efficient in the absence of a solvent (Entry 6) or on water, which gave quantitative yield (Entry 7). Both *E*‐ and Z‐products were formed in approximately equal amounts providing both diastereoisomers for further functionalization, as was desired for a divergent strategy.

Excellent yields were observed both for the reaction without solvent or run on H_2_O, up to a 10 mmol scale (Table 1, entries 6–8, 10). However, when the neat cyclopropanation was carried out at scales greater than 10 mmol, an exotherm was observed with concomitant gas evolution and a significant decrease in yield (Table 1, entry 9). This was not observed when the reaction was carried out on water, and under these conditions reaction on a 40 mmol scale afforded excellent yields without indication of a significant increase in temperature.

Purification and separation of the diastereoisomers was facile on smaller scales, but on larger scale, separation of a catalyst derived impurity became problematic. This was resolved through modification of the work‐up procedure: bubbling air through a diluted reaction mixture oxidized the remaining Co^II^ catalyst to a putative Co^III^–peroxo‐bridged dimer^28^ which was simply removed by filtration through a pad of silica. The rate of Co^II^ oxidation was highly dependent on the diluent due to different oxygen permeability, dissolution capability and coordinating effects of the solvents, with isohexane performing best (see the Supporting Information for further details).^29^ This protocol, diluting the reaction with isohexane then bubbling air through the solvent for 15 min followed by filtration through silica, enabled facile removal of the catalyst and derived impurities and then separation of the diastereoisomers by flash chromatography. Approximately 4 g of each separated diastereoisomer was readily formed in a single run (Table 1, entry 11).

**Figure 3:**
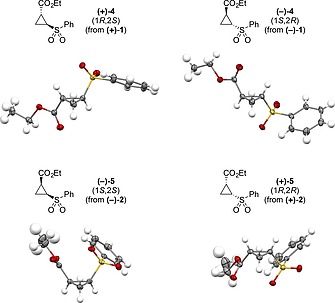
Enantiomerically pure cyclopropyl sulfides were obtained by preparative chiral SFC and analysed by X‐ray crystallography of the corresponding sulfones.

The enantiomers of both diastereoisomers were also readily separated by preparative chiral supercritical fluid chromatography (SFC) to afford all four possible stereoisomers, each in ≥ 97 % *ee*. Whereas the sulfides were oils, the corresponding cyclopropyl sulfones (vide infra) were crystalline. Absolute configurations and structural confirmation of the sulfones and hence precursor sulfides was proven through single‐crystal X‐ray diffraction analysis (Figure 3).^30^

This separation approach provided highly enantioenriched compounds **1** and **2**, hence granting access to each derivative as a single enantiomer. As an alternative approach to generate the enantioenriched cyclopropane derivatives, chiral Cu^I^ and Co^II^ catalysts were investigated (see the Supporting Information for further details). Enantioenriched catalyst **3** afforded moderate *ee* values for both the *trans* and *cis* compounds (52 % *ee* **1** and 77 % *ee* **2**, in H_2_O at 20 °C, 57 % overall yield). However, this was not the focus of this study, and for the derivatization reactions presented below the racemate was used to form a racemic screening set.

### Sulfide Oxidation and Ester Functionalization

**Scheme 2:**
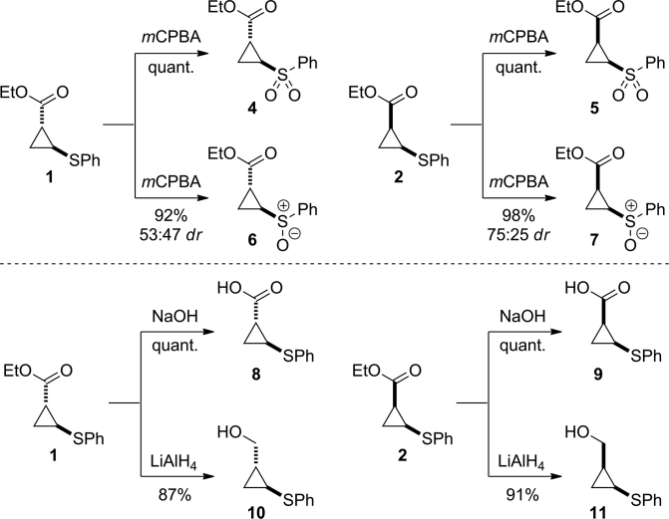
Scaffold derivatization by oxidation, reduction or hydrolysis.

With a practical, high yielding and scalable route to cyclopropyl scaffolds **1** and **2**, functionalization of the sulfide and ester groups was examined. Oxidation of the sulfide to the sulfone was readily achieved in quantitative yield using excess *m*CPBA (Scheme 2). This provided a short route to the functionalized cyclopropyl sulfone derivatives as single diastereoisomers, themselves interesting motifs in biologically active compounds.^31^, ^32^ Using 1 equiv. *m*CPBA at 0 °C gave sulfoxides **6** and **7** in high yields as a mixture of diastereoisomers at sulfur,^33^, ^34^ and as important compounds for our envisaged sulfoxide exchange strategy.

**Scheme 3:**
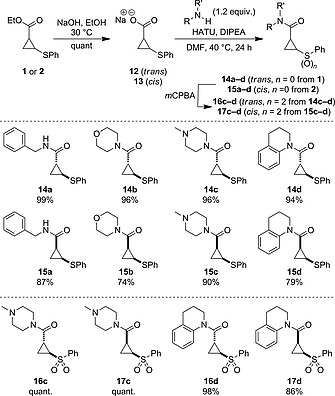
Synthesis of amide‐substituted cyclopropanes through amidation and oxidation. Yields for **14**/**15** quoted over 2 steps from **1**/**2**.

The ethyl ester was readily hydrolyzed to the carboxylic acid using sodium hydroxide, for both diastereoisomers (Scheme 2). Alternatively, reduction with LiAlH_4_ gave the primary alcohol‐cyclopropyl sulfide in high yields. To form the cyclopropyl amides, the cyclopropyl carboxylate salt was reacted directly, using a method described by Batey.^35^ Under the same conditions used for hydrolysis above, the reaction mixture was evaporated to form the carboxylate salt in quantitative yield, which was itself characterized. This was applied directly in a HATU‐promoted amidation reaction to form a series of amides **14a**–**14d** from *trans*‐cyclopropane **1**, and **15a**–**15d** from *cis*‐**2**. Such motifs are widely found in biologically active molecules (for example see Figure 1). A selection of the resulting cyclopropyl amides was oxidized to the corresponding sulfones to generate further interesting compounds with desirable physicochemical properties for drug discovery (**16c**,**d** and **17c**,**d**). For compounds **16c** and **17c**, aqueous work‐up proved problematic due to high water solubility. This issue was overcome by an aqueous‐free work‐up. Excess *m*CPBA was quenched by the addition of solid Na_2_S_2_O_5_ to the reaction, which was followed by removal of the reaction solvent, redissolution in acetone containing 5 % Et_3_N and filtration. Under this procedure, after collection of the filtrate, all *m*CPBA derived materials had been removed, providing the sulfones in excellent purity and yield. All of the transformations described were achieved without any epimerization to the corresponding diastereoisomers, as indicated by ^1^H NMR (Scheme 3).

### Stability and Reactions of Cyclopropylmagnesium Reagents Generated by Sulfoxide–Magnesium Exchange

A key aspect of our approach was that for increased diversity, the position of the sulfur group should be functionalizable, through its removal, in order to form bonds directly to the cyclopropyl ring. We intended to utilize cyclopropyl sulfoxides, and a sulfoxide–metal exchange strategy to allow functionalization of the anion.

Sulfoxide–metal exchange has been used to generate three‐membered ring organometallics, in the form of cyclopropanes,^21^, ^36^ aziridines,^22^ and epoxides,^23^ for reaction with limited examples of reactive electrophiles or protonation. Knochel has previously reported halogen–metal exchange to generate *cis*‐[2‐(ethoxycarbonyl)‐cyclopropyl]magnesium chloride,^37^, ^38^ comparable to the Grignard reagent that would be generated from **2** by sulfoxide–magnesium exchange. In the Knochel work, coordination between the ester and the Lewis acidic magnesium atom was proposed. Therefore, we concentrated our efforts on optimizing the exchange for the *trans*‐derivative, where such a potentially stabilizing interaction would not be possible.

Early investigation of this reaction showed that *i*PrMgCl formed the putative cyclopropyl Grignard reagent **18** from cyclopropane **6** efficiently at –78 °C, also generating isopropyl phenyl sulfoxide. Trapping the intermediate using I_2_ gave cyclopropyl iodide **19a** as a single *trans*‐diastereoisomer. Importantly, the reaction proceeded with retention of configuration for both the *E*‐ and the *Z*‐cyclopropyl sulfoxides.^39^

**Figure 4:**
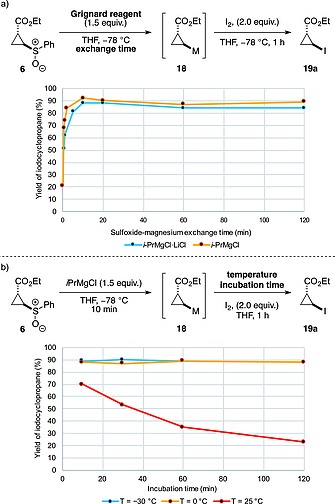
a) The effect of using *i*PrMgCl or *i*PrMgCl**·**LiCl on the stability of the cyclopropyl organometallic intermediate after various exchange periods. b) The effect of temperature on the stability of the cyclopropyl organometallic intermediate after various incubation periods.

To maximize the reactivity of the organometallic species while avoiding degradation, we assessed both the time required for the exchange to go to completion, and the stability of the cyclopropyl Grignard intermediate species. Sulfoxide–magnesium exchange reactions were conducted on cyclopropane **6** using *i*PrMgCl and *i*PrMgCl**·**LiCl,^40^ trapping with molecular iodine at –78 °C after different time periods (Figure 4a).

**Scheme 4:**
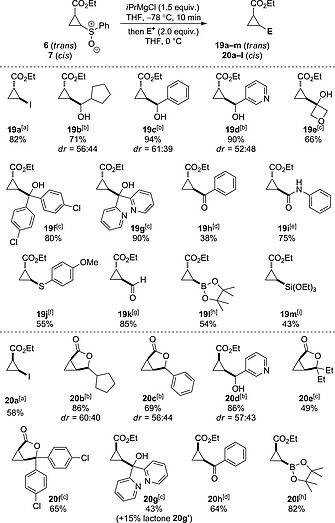
Scope of electrophiles for the sulfoxide–magnesium exchange, electrophilic trap protocol. [a] Trapping with I_2_. [b] From the corresponding aldehyde. [c] From the corresponding ketone. [d] Using benzoyl chloride. [e] Using phenylisocyanate. [f] Using bis(4‐methoxyphenyl)disulfide. [g] Using DMF [h] Using (pin)BO*i*Pr. [i] Using (EtO)_3_SiCl.

This study led to several observations i) the exchange was complete in 10 min, ii) the cyclopropyl Grignard reagent was stable for at least 2 hours at –78 °C prior to the addition of the electrophile, and iii) similar yields were obtained for the reactions using *i*PrMgCl and *i*PrMgCl**·**LiCl at each time point, indicating the addition of LiCl did not provide an advantage. Next, we investigated the thermal stability of the Grignard intermediate by conducting the sulfoxide–magnesium exchange with *i*PrMgCl at –78 °C (10 min), then incubating the reaction mixture at either –30, 0 or 25 °C for different time periods, prior to trapping at this temperature (Figure 4b). Pleasingly, the cyclopropyl intermediate was stable for over 2 h at temperatures up to 0 °C. However, at 25 °C significant decomposition was observed, which corresponded to a reduction of product yield by half after approximately 45 min of incubation, and <25 % yield after 2 h. Following this study, various electrophiles were investigated, trapping at 0 °C to maximize the reactivity. Both the *E*‐ and the *Z*‐cyclopropyl sulfoxides could be trapped efficiently with I_2_ to generate cyclopropanes **19a** and **20a** (Scheme 4).

With these exchange conditions in hand, a wide range of electrophiles were examined, intending to generate varied cyclopropane containing structures (Scheme 4). The Grignard reagent **18** originating from *E*‐cyclopropyl sulfoxide **6** could be trapped efficiently with aliphatic, aromatic and heteroaromatic aldehydes in excellent yields (**19b**–**19d**). Dialkyl, diaryl and diheteroaryl ketones also proceeded in good to excellent yields (**19e**–**19g**). Electrophilic trapping of the cyclopropyl Grignard was observed with benzoyl chloride to give the cyclopropyl ketone (**19h**). Trapping with phenyl isocyanate generated the cyclopropyl amide (**19i**) and trapping with a disulfide gave the corresponding cyclopropyl sulfide (**19j**). Finally, a series of potential cyclopropane building‐blocks were prepared. Trapping with *N*,*N*‐dimethylformamide allowed access to the cyclopropyl aldehyde in an excellent yield (**19k**), an isopropoxy dioxaborolane generated the corresponding cyclopropyl boronic ester (**19l**) and trapping with chlorotriethoxysilane produced the triethoxysilylcyclopropane (**19m**). The Grignard reagent originating from the *Z*‐cyclopropyl sulfoxide could also be trapped with a similar series of electrophiles, generating diversely substituted *cis*‐cyclopropanes. Interestingly, it was observed that with certain aldehyde and ketone electrophiles initial electrophilic attack was followed by lactonization to generate the bicyclic product. Complete lactonization was observed to generate **20b**, **20c** and **20f**, whereas alcohol **20g** was the major product with the dipyridyl ketone electrophile.

**Scheme 5:**
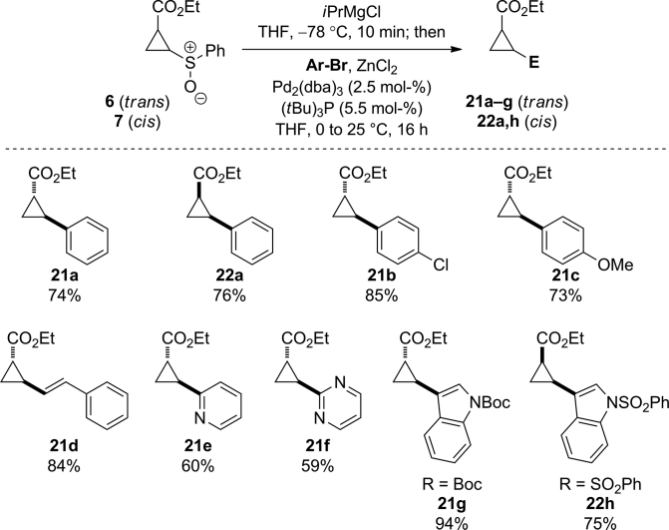
Substrate scope for the sulfoxide–magnesium exchange–Negishi cross‐coupling protocol.

Next we explored a sulfoxide–magnesium exchange–Negishi cross‐coupling protocol to form (hetero)aryl cyclopropanes which are important pharmacophores.[12c] There are no prior examples of cross‐coupling between aryl halides and cyclopropyl organometallics derived from cyclopropyl sulfoxides.[21c] We employed a protocol similar to that which we recently reported for aziridine sulfoxides,[22e] and were delighted to observe the successful Negishi cross‐coupling from both *trans* and *cis*‐cyclopropane derivatives with aryl bromides (Scheme 5).

The same sulfoxide–magnesium exchange protocol as developed above was used to generate the intermediate cyclopropyl Grignard reagent. A mixture of Pd_2_(dba)_3_, (*t*Bu)_3_P and ZnCl_2_ (1.5 equiv.) in THF was added and the mixture stirred for 1 h at 0 °C followed by the addition of the aryl bromide (2 equiv.). The coupling of bromobenzene proceeded at 25 °C over 15 h to give cyclopropanes **21a** and **22a** in excellent yields. Using the *trans*‐cyclopropyl sulfoxide, 1‐bromo‐4‐chlorobenzene and 2‐bromoanisole both gave high yields of the aryl‐cyclopropanes **21b** and **21c** respectively. Cross‐coupling with β‐bromostyrene gave an excellent yield of the corresponding vinyl cyclopropane **21d**, which would be challenging to form through carbenoid insertion to alkenes. It is notable that Lewis basic sites on electron‐poor heterocycles as well as electron‐rich heterocycles can present difficulties in other cyclopropane strategies, hence heteroaromatic bromides were investigated. Both electron‐poor and electron‐rich heterocycles could be readily incorporated to give pyridine, pyrimidine and indole cyclopropane derivatives **21e**–**21g** respectively. *cis*‐Cyclopropyl sulfoxide **7** also successfully underwent sulfoxide–magnesium exchange–Negishi cross‐coupling with a bromoindole to generate *cis*‐cyclopropane **22h**.

**Scheme 6:**
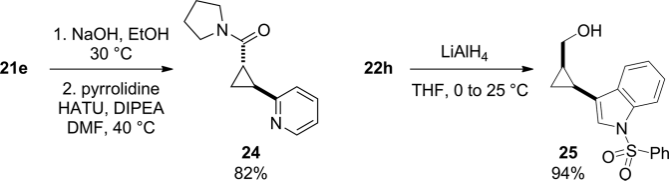
Synthesis of bifunctional cyclopropanes by utilizing both synthetic handles.

Finally, we chose to further elaborate compounds **21e** and **22h** through the remaining ester functionality (Scheme 6). From pyridyl cyclopropane **21e**, hydrolysis to give the *E*‐cyclopropyl carboxylate sodium salt **23** followed by amidation with pyrrolidine gave the corresponding cyclopropyl amide **24** in an 82 % yield as a relatively complex yet low molecular weight fragment. Similarly, reduction of indole‐cyclopropyl ester **22h** to the primary alcohol **25** proceeded in a 94 % yield. Both **24** and **25** presented structural features related to biologically active compounds shown in Figure 1, and were rapidly accessed as interesting fragment and lead‐like motifs.

### Fragment and Lead‐Likeness Analysis

**Figure 5:**
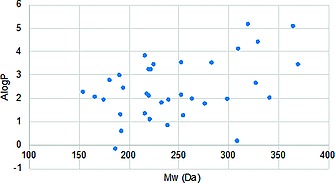
The relationship between ALog P and M_W_ for compounds prepared.

The cyclopropane‐containing compounds prepared in this study were designed to possess desirable physicochemical properties and sample new areas of chemical space for medicinal chemistry.^41^, ^42^ To illustrate the success of this approach the library was assessed against parameters of Alog P vs. M_W_, and molecular shape through a principal moments of inertia (PMI) plot,^43^ calculated using the Lead‐likeness and Molecular Analysis (LLAMA) software, developed by Nelson and Marsden.^41^ All compounds prepared in this study were included with the exception of those considered reactive building blocks (iodides **19a** and **20a**, aldehyde **19k**, pinacol boronates **19l** and **20l**, and ethoxysilane **19m**).^44^ The molecular properties of the 50 included compounds were shown to explore efficiently lead‐like and fragment space (Figure 5).

**Figure 6:**
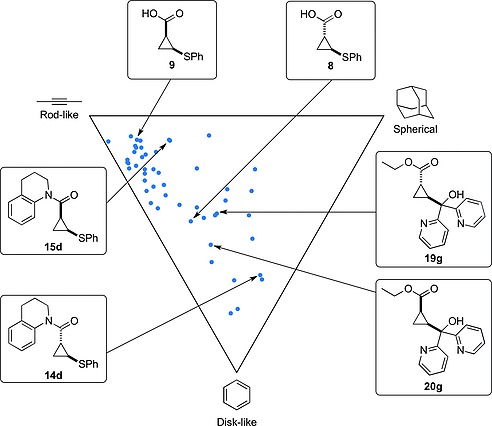
PMI plot showing the shape distribution of the synthesized compounds. Some examples have been selected to illustrate the difference between the *E*‐ and *Z*‐diastereoisomers.

The PMI plot generated from the normalized ratios of principal moments of inertia calculated through LLAMA, showed that the set of compounds possess varied 3‐dimensional structures, sampling chemical space away from the planar rod‐like–disk‐like axis (Figure 6). The different 3‐dimensional structures of the *E*‐ and *Z*‐diastereoisomers are exemplified by three sets of compounds highlighted.

The powerful LLAMA software also executes virtual elaboration of the molecular scaffolds and calculates the physicochemical properties of the resulting compounds.^41^ This indicates the potential of scaffolds to generate a much wider array of lead‐like or drug‐like compounds through elaboration by common reactions used in medicinal chemistry. The 56 cyclopropanes prepared in this study, including this time those compounds intended as building blocks, were examined as scaffolds in this program. Given the presence of ester functionality in several of the compounds in our study, a slight modification of default decoration inputs was applied, by the addition of an ester hydrolysis reaction option. Using the default set of 44 reactants within LLAMA, and permitting up to 2 reactions on the scaffolds, 1187 cyclopropane‐containing compounds were generated.^45^ Of these, 392 examples had a molecular weight less than 300 and 1033 examples had a molecular weight less than 500, indicative of fragment and drug‐like space respectively. A PMI plot of the decorated compounds shows that the full set of elaborated compounds displayed highly varied topologies, and were significantly removed from the often over‐populated region along the rod‐like–disk‐like axis (see Graph S2 in the Supporting Information).^45^

## Conclusions

In conclusion, we have developed a strategy for preparing a diverse range of cyclopropane‐containing fragments, lead‐like compounds and building blocks from a readily accessible cyclopropyl scaffold. An operationally facile and scalable Co^II^‐catalyzed cyclopropanation of phenyl vinyl sulfide was developed to prepare the bifunctional cyclopropyl scaffold. Divergent, orthogonal derivatization of the scaffold has been demonstrated through hydrolysis, amidation, reduction and oxidation reactions, as well as sulfoxide–magnesium exchange protocols. Investigations into the stability of the cyclopropyl Grignard species have led to successful trapping with a broad scope of electrophiles. A sulfoxide–magnesium exchange–Negishi cross‐coupling protocol enables (hetero)aryl rings to be installed directly onto the intact cyclopropane ring. Finally, we have presented the calculated physicochemical properties of the synthesized compounds and of potential derivatives which supports their value as potential screening compounds.

## Experimental Section

**General Experimental Considerations:** All non‐aqueous reactions were run under an inert atmosphere (argon) with flame‐dried glassware using standard techniques. Anhydrous solvents were obtained by filtration through drying columns (THF, CH_2_Cl_2_, toluene, DMF). Where applicable, room temp. denotes a room temperature of approximately 22 °C, and a specifically noted temperature e.g. “stirred at 25 °C” indicates the stated temperature was accurately maintained. Flash column chromatography was performed using 230–400 mesh silica with the indicated solvent system according to standard techniques. Analytical thin‐layer chromatography (TLC) was performed on precoated, glass‐backed silica gel plates. Visualization of the developed chromatogram was performed by UV absorbance (254 nm), aqueous potassium permanganate, vanillin, ninhydrin or *p*‐anisaldehyde stains as appropriate.

Infrared spectra (ν̃_max_, FTIR ATR) were recorded in reciprocal centimeters [cm^–1^]. Nuclear magnetic resonance spectra were recorded on 400 or 500 MHz spectrometers. Chemical shifts for ^1^H NMR spectra are recorded in parts per million from tetramethylsilane with the solvent resonance as the internal standard (chloroform: *δ* = 7.27 ppm, DMSO: *δ* = 2.50 ppm). Data is reported as follows: chemical shift [multiplicity (s = singlet, d = doublet, t = triplet, m = multiplet and br = broad), coupling constant in Hz, integration, assignment]. ^13^C NMR spectra were recorded with complete proton decoupling. Chemical shifts are reported in parts per million from tetramethylsilane with the solvent resonance as the internal standard [^13^CDCl_3_: *δ* = 77.0 ppm, (^13^CD_3_)_2_SO: *δ* = 39.5 ppm]. *J* values are reported in Hz. Assignments of ^1^H and ^13^C spectra were based upon the analysis of *δ* and *J* values, as well as COSY, HSQC, HMBC and NOESY experiments where appropriate. Melting points are uncorrected. Optical rotations (*α*′) were recorded at the indicated temperature (*T* °C) and were converted into the corresponding specific rotations [*α*]_D_^*T*^. Commercial reagents were used as supplied or purified by standard techniques where necessary. Use of Diazo Compounds: Although we have not experienced any problems in the handling or reaction of diazo reagents, extreme care should be taken when manipulating them due to their potentially explosive nature. Co^II^‐Catalyzed Cyclopropanation: For the Co^II^‐catalyzed procedure, no special precautions were taken to exclude air or moisture from the catalyst during storage or handling. After all reagents were added the reaction vessel was sealed with either a crimp seal microwave vial lid with a septum, or a suba seal and the reaction vessel flushed with Ar_(g)_. Ar_(g)_ flushed, deflated balloons were attached to the flask, so that the total potential volume of the balloons when inflated was greater than the volume of N_2(g)_ evolved from the reaction. On scales where ≥ 10 mmol of diazo compound were used, a precautionary blast shield was placed between the reaction flask and the fume hood sash.

**Supporting Information** (see footnote on the first page of this article): Further details can be found in Supporting Information.

## Supporting information

Supporting Information

## References

[1] a) C. W. Murray and D. C. Rees , Angew. Chem. Int. Ed., 2016, 55, 488–492; 10.1002/anie.201506783 26526786

[2] a) F. Lovering , J. Bikker and C. Humblet , J. Med. Chem., 2009, 52, 6752–6756; 19827778 10.1021/jm901241e

[3] a) A. Nadin , C. Hattotuwagama and I. Churcher , Angew. Chem. Int. Ed., 2012, 51, 1114–1122; 10.1002/anie.201105840 22271624

[4] a) A. W. Hung , A. Ramek , Y. Wang , T. Kaya , J. A. Wilson , P. A. Clemons and D. W. Young , Proc. Natl. Acad. Sci. USA, 2011, 108, 6799–6804; 21482811 10.1073/pnas.1015271108 PMC3084099

[5] For recent examples, see: a) N. Kato , E. Comer , T. Sakata‐Kato , A. Sharma , M. Sharma , M. Maetani , J. Bastien , N. M. Brancucci , J. A. Bittker , V. Corey , D. Clarke , E. R. Derbyshire , G. L. Dornan , S. Duffy , S. Eckley , M. A. Itoe , K. M. J. Koolen , T. A. Lewis , P. S. Lui , A. K. Lukens , E. Lund , S. March , E. Meibalan , B. C. Meier , J. A. McPhail , B. Mitasev , E. L. Moss , M. Sayes , Y. Van Gessel , M. J. Wawer , T. Yoshinaga , A.‐M. Zeeman , V. M. Avery , S. N. Bhatia , J. E. Burke , F. Catteruccia , J. C. Clardy , P. A. Clemons , K. J. Dechering , J. R. Duvall , M. A. Foley , F. Gusovsky , C. H. M. Kocken , M. Marti , M. L. Morningstar , B. Munoz , D. E. Neafsey , A. Sharma , E. A. Winzeler , D. F. Wirth , C. A. Scherer and S. L. Schreiber , Nature, 2016, 538, 344–349; 27602946 10.1038/nature19804 PMC5515376

[6] S. M. Paul , D. S. Mytelka , C. T. Dunwiddie , C. C. Persinger , B. H. Munos , S. R. Lindborg and A. L. Schacht , Nat. Rev. Drug Discovery, 2010, 9, 203–214. 20168317 10.1038/nrd3078

[7] a) C. A. Lipinski , F. Lombardo , B. W. Dominy and P. J. Feeney , Adv. Drug Delivery Rev., 1997, 23, 3–25; 10.1016/s0169-409x(00)00129-0 11259830

[8] Astex have described fragment guidelines that advise M_W_ < 300, HBD ≤ 3, HBA ≤ 3, clog P ≤ 3, see reference 7b; GlaxoSmithKline described lead‐like compounds as possessing –1 ≤ clog P ≤ 3 and 14 ≤ heavy atoms ≤ 26 (200 ≤ M_W_ ≤ 350), see reference 3a. Also see: S. J. Teague , A. M. Davis , P. D. Leeson and T. Oprea , Angew. Chem. Int. Ed., 1999, 38, 3743–3748;

[9] T. T. Talele , J. Med. Chem., 2016, 59, 8712–8756. 27299736 10.1021/acs.jmedchem.6b00472

[10] R. D. Taylor , M. MacCoss and A. D. G. Lawson , J. Med. Chem., 2014, 57, 5845–5859. 24471928 10.1021/jm4017625

[11] For compounds in Figure 1; a) W.‐L. Wu , T. A. Bara , D. A. Burnett , J. W. Clader , M. S. Domalski , Y. Jin , H. B. Josien , H. Li , X. Liang , D. A. Pissarnitski , T. K. Sasikumar , J. K. Wong , R. Xu , Z. Zhao , P. McNamara (Schering Corporation), WO 2009/008980 A2, 2009;

[12] For selected recent examples of cyclopropanes in medicinal chemistry, see: a) L.‐Q. Sun , E. Mull , B. Zheng , S. D'Andrea , Q. Zhao , A. X. Wang , N. Sin , B. L. Venables , S.‐Y. Sit , Y. Chen , J. Chen , A. Cocuzza , D. M. Bilder , A. Mathur , R. Rampulla , B.‐C. Chen , T. Palani , S. Ganesan , P. N. Arunachalam , P. Falk , S. Levine , C. Chen , J. Friborg , F. Yu , D. Hernandez , A. K. Sheaffer , J. O. Knipe , Y.‐H. Han , R. Schartman , M. Donoso , K. Mosure , M. W. Sinz , T. Zvyaga , R. Rajamani , K. Kish , J. Tredup , H. E. Klei , Q. Gao , A. Ng , L. Mueller , D. M. Grasela , S. Adams , J. Loy , P. C. Levesque , H. Sun , H. Shi , L. Sun , W. Warner , D. Li , J. Zhu , Y.‐K. Wang , H. Fang , M. I. Cockett , N. A. Meanwell , F. McPhee and P. M. Scola , J. Med. Chem., 2016, 59, 8042–8060; 27564532 10.1021/acs.jmedchem.6b00821

[13] a) H. Y. Kim and P. J. Walsh , Acc. Chem. Res., 2012, 45, 1533–1547; 22725974 10.1021/ar300052s

[14] a) H. E. Simmons and R. D. Smith , J. Am. Chem. Soc., 1958, 80, 5323–5324;

[15] For other carbenoid species see: Z. Ke , Y. Zhou , H. Gao , C. Zhao and D. L. Phillips , Chem. Eur. J., 2007, 13, 6724–6731. 17508383 10.1002/chem.200700145

[16] a) A. Ford , H. Miel , A. Ring , C. N. Slattery , A. R. Maguire and M. A. McKervey , Chem. Rev., 2015, 115, 9981–10080; 26284754 10.1021/acs.chemrev.5b00121

[17] For examples of Co‐catalyzed cyclopropanation reactions, see: a) T. Fukuda and T. Katsuki , Tetrahedron, 1997, 53, 7201–7208;

[18] a) E. J. Corey and M. Chaykovsky , J. Am. Chem. Soc., 1965, 87, 1353–1364;

[19] a) Z. Qureshi , C. Toker and M. Lautens , Synthesis, 2017, 49, 1–16;

[20] a) S. Miyamura , M. Araki , T. Suzuki , J. Yamaguchi and K. Itami , Angew. Chem. Int. Ed., 2015, 54, 846–851; 10.1002/anie.201409186 25348582

[21] For previous examples of sulfoxide–metal exchange on cyclopropanes: a) A. Abramovitch , L. Fensterbank , M. Malacria and I. Marek , Angew. Chem. Int. Ed., 2008, 47, 6865–6868; 10.1002/anie.200802093 18661465

[22] For examples of sulfoxide–metal exchange on aziridines: a) T. Satoh and Y. Fukuda , Tetrahedron, 2003, 59, 9803–9810;

[23] For examples of sulfoxide–metal exchange on epoxides: a) T. Satoh , T. Oohara , Y. Ueda and K. Yamakawa , J. Org. Chem., 1989, 54, 3130–3136;

[24] C. Kaiser , B. M. Lester , C. L. Zirkle , A. Burger , C. S. Davis , T. J. Delia and L. Zirngibl , J. Med. Chem., 1962, 5, 1243–1265. 10.1021/jm01241a017 14056458

[25] For a related thermal reaction with methyl diazoacetate, see: W. Ando , J. Org. Chem., 1977, 42, 3365–3372.

[26] For alternative, classical approaches to cyclopropyl sulfides, see for example: a) K. Tanaka , I. Funaki , A. Kaji , K. Minami , M. Sawada and T. Tanaka , J. Am. Chem. Soc., 1988, 110, 7185–7188;

[27] For a recent example of cyclopropanation of 1,1‐disubstituted alkenes using a Co‐catalyst, including a single example of a vinyl sulfide, see: J. D. White and S. Shaw , Org. Lett., 2014, 16, 3880–3883. 25020111 10.1021/ol501549x

[28] Similar peroxo‐bridged dimeric structures are known and characterized by X‐ray crystallography, see: a) R. G. Wilkins in Bioinorganic Chemistry, Volume 100, Chapter 6, (Eds.: Dessy R., Dillard J., Taylor L.), American Chemical Society, 1971, pp. 111–134;

[29] M. Quaranta , M. Murkovic and I. Klimant , Analyst, 2013, 138, 6243–6245. 23803965 10.1039/c3an36782g

[30] CCDC 1548245 [for (+)‐**4**], 1548246 [for (–)‐**4**], 1550178 [for (–)‐**5**], and 1550151 [for (+)‐**5**] contain the supplementary crystallographic data for this paper. These data can be obtained free of charge from The Cambridge Crystallographic Data Centre.

[31] For cyclopropyl sulfones in biologically active compounds see: a) ref.[11a];

[32] For alternative synthetic approaches to cyclopropyl sulfones, see: a) ref.[17d];

[33] For alternative routes for the synthesis of cyclopropyl sulfoxides, see: a) reference 21;

[34] The relative stereochemistry of the individual sulfoxide diastereoisomers was not determined (with respect to the diastereoisomers at sulfur).

[35] J. D. Goodreid , P. A. Duspara , C. Bosch and R. A. Batey , J. Org. Chem., 2014, 79, 943–954. 24354665 10.1021/jo402374c

[36] For examples of the generation of cyclopropylmagnesium carbenoids by sulfoxide–magnesium exchange, see: a) T. Kimura , N. Wada , T. Tsuru , T. Sampei and T. Satoh , Tetrahedron, 2015, 71, 5952–5958;

[37] V. A. Vu , I. Marek , K. Polborn and P. Knochel , Angew. Chem. Int. Ed., 2002, 41, 351–352; 10.1002/1521-3773(20020118)41:2<351::aid-anie351>3.0.co;2-5 12491430

[38] The substrate *cis*‐ethyl 2‐iodocyclopropane‐1‐carboxylate was itself prepared in 5 steps. For reactions with this compound, see reference 37. For full experimental details, see: V. A. Vu , PhD thesis, Ludwig‐Maximilians‐Universität München (Germany), 2003.

[39] There was no difference in reactivity observed for the cyclopropyl sulfoxide diastereoisomers (with respect to the diastereoisomers at sulfur).

[40] a) A. Krasovskiy and P. Knochel , Angew. Chem. Int. Ed., 2004, 43, 3333–3336; 10.1002/anie.200454084 15213967

[41] Molecular properties were calculated using LLAMA (Lead‐Likeness and Molecular Analysis) software, available freely online at https://llama.leeds.ac.uk;

[42] See the Supporting Information for a list of the physicochemical properties (M_W_, Alog P, HBD/HBA, Fsp^3^ and PSA) of synthesized compounds.

[43] W. H. B. Sauer and M. K. Schwarz , J. Chem. Inf. Comput. Sci., 2003, 43, 987–1003. 12767158 10.1021/ci025599w

[44] Note that the software gives the same Alog P values for *cis* and *trans* derivatives with the same substituents. Compounds **6**, **7**, **19b**, **19c**, **19d**, **20b**, **20c** and **20d** were formed as a mixture of two diastereoisomers, and were entered into LLAMA with undefined stereochemistry for relevant the unassigned stereocenter.

[45] See the Supporting Information for M_W_ vs. Alog P and PMI plots for the decorated compounds produced using LLAMA.

