# Supplementary material for: Divergent Synthesis of Cyclopropane‐Containing Lead‐Like Compounds, Fragments and Building Blocks through a Cobalt Catalyzed Cyclopropanation of Phenyl Vinyl Sulfide
Source: European J Org Chem. 2017 Sep 11;2017(34):5015–24. doi: 10.1002/ejoc.201701030 (PMC5601191; doi:10.1002/ejoc.201701030)
Supplement: Supplementary file 1 — Supporting Information [file EJOC-2017-5015-s001.pdf]

Eur. J. Org. Chem. **2017** · ISSN 1099–0690

https://doi.org/10.1002/ejoc.201701030

SUPPORTING INFORMATION

Title: Divergent Synthesis of Cyclopropane-Containing Lead-Like Compounds, Fragments and Building Blocks through a Cobalt Catalyzed Cyclopropanation of Phenyl Vinyl Sulfide

Author(s): Stephen J. Chawner, Manuel J. Cases-Thomas, James A. Bull*

Table of Contents S1
General Experimental Considerations S2
SMILES and InChI codes for synthesized compounds S3-S6
Cyclopropanation of phenyl vinyl sulfide: initial catalyst screening S7
Cyclopropanation of phenyl vinyl sulfide: product purification S8
Calculated fragment and lead-like properties for cyclopropane derivatives S9–11
Virtual scaffold decoration and LLAMA compound analysis S12–S13
Experimental Details and Charaterization Data S14–S35
- Synthesis of **1** and **2** by Co^{II}-catalyzed cyclopropanation S14
- Synthesis of **4**–**7** through sulfide oxidation S15–S16
- Synthesis of **8**–**13** through ester derivatization S16–S18
- Synthesis of **14a**–**15d** through amide bond formation S18–S21
- Synthesis of **16c**–**17d** through sulfide oxidation S21–S22
- Synthesis of **19a**–**20l** through sulfoxide-magnesium exchange, electrophilic trapping S22–S30
- Synthesis of **21a**–**22h** through sulfoxide-magnesium exchange, Negishi cross-coupling S31–S34
- Synthesis of **23**, **24** and **25** S34–S35

Optimization of an enantioselective cyclopropanation of phenyl vinyl sulfide S36–S37
Synthesis of enantiopure C_2-symmetric Co^{II}(salen)-type complexes S38
HPLC and SFC traces and conditions S39–S45
Optical rotation data for enantiopure cyclopropanes 1, 2, 4 and 5 .... S46
^{1}H, ^{13}C and ^{11}B NMR spectra S47–S108
X-ray crystallography data S109–S141
References S142

General Experimental Considerations

All non-aqueous reactions were run under an inert atmosphere (argon) with flame-dried glassware using standard techniques. Anhydrous solvents were obtained by filtration through drying columns (THF, CH_2Cl_2, toluene, DMF). Where applicable, rt denotes a room temperature of approximately 22 °C, and a specifically noted temperature e.g. "stirred at 25 °C" indicates the stated temperature was accurately maintained.

Flash column chromatography was performed using 230-400 mesh silica with the indicated solvent system according to standard techniques. Analytical thin-layer chromatography (TLC) was performed on precoated, glass-backed silica gel plates. Visualization of the developed chromatogram was performed by UV absorbance (254 nm), aqueous potassium permanganate, vanillin, ninhydrin or *p*-anisaldehyde stains as appropriate.

Infrared spectra (ν_{max}, FTIR ATR) were recorded in reciprocal centimeters (cm^{-1}).

Nuclear magnetic resonance spectra were recorded on 400 or 500 MHz spectrometers. Chemical shifts for ^1H NMR spectra are recorded in parts per million from tetramethylsilane with the solvent resonance as the internal standard (chloroform: δ = 7.27 ppm, DMSO: δ = 2.50 ppm). Data is reported as follows: chemical shift [multiplicity (s = singlet, d = doublet, t = triplet, m = multiplet and br = broad), coupling constant in Hz, integration, assignment]. ^{13}C NMR spectra were recorded with complete proton decoupling. Chemical shifts are reported in parts per million from tetramethylsilane with the solvent resonance as the internal standard ($^{13}CDCl_3$: δ = 77.0 ppm, $(^{13}CD_3)_2SO$: δ = 39.5 ppm). *J* values are reported in Hz. Assignments of ^1H and ^{13}C spectra were based upon the analysis of δ and *J* values, as well as COSY, HSQC, HMBC and NOESY experiments where appropriate.

Melting points are uncorrected.

Optical rotations (α') were recorded at the indicated temperature (T °C) and were converted to the corresponding specific rotations $[\alpha]_D^T$.

Commercial reagents were used as supplied or purified by standard techniques where necessary.

Use of diazo compounds: Although we have not experienced any problems in the handling or reaction of diazo reagents, extreme care should be taken when manipulating them due to their potentially explosive nature.

Cu^I-catalyzed cyclopropanation: For the Cu^I-catalyzed procedure, all catalysts were stored in a dessicator, except for Cu^IOTf which was stored and handled in a glovebox. Reactions were conducted in a sealed microwave vial. Slow addition of the diazo compound solution was achieved with a syringe pump.

Co^{II}-catalyzed cyclopropanation: For the Co^{II}-catalyzed procedure, no special precautions were taken to exclude air or moisture from the catalyst during storage or handling. After all reagents were added the reaction vessel was sealed with either a crimp seal microwave vial lid with a septum, or a suba seal and the reaction vessel flushed with $Ar_{(g)}$. $Ar_{(g)}$ flushed, deflated balloons were attached to the flask, so that the total potential volume of the balloons when inflated was greater than the volume of $N_{2(g)}$ evolved from the reaction. On scales where ≥10 mmol of diazo compound were used, a precautionary blast shield was placed between the reaction flask and the fume hood sash.

SMILES and InChI codes for synthesized compounds

Compound	SMILES	InChI
**1**	O=C(OCC)[C@H]1C[C@@H]1SC2=CC=CC=C2	InChI=1S/C12H14O2S/c1-2-14-12(13)10-8-11(10)15-9-6-4-3-5-7-9/h3-7,10-11H,2,8H2,1H3/t10-,11-/m0/s1
**2**	O=C(OCC)[C@@H]1C[C@@H]1SC2=CC=CC=C2	InChI=1S/C12H14O2S/c1-2-14-12(13)10-8-11(10)15-9-6-4-3-5-7-9/h3-7,10-11H,2,8H2,1H3/t10-,11+/m1/s1
**4**	O=C(OCC)[C@H]1C[C@@H]1S(C2=CC=CC=C2)(=O)=O	InChI=1S/C12H14O4S/c1-2-16-12(13)10-8-11(10)17(14,15)9-6-4-3-5-7-9/h3-7,10-11H,2,8H2,1H3/t10-,11-/m0/s1
**5**	O=C(OCC)[C@@H]1C[C@@H]1S(C2=CC=CC=C2)(=O)=O	InChI=1S/C12H14O4S/c1-2-16-12(13)10-8-11(10)17(14,15)9-6-4-3-5-7-9/h3-7,10-11H,2,8H2,1H3/t10-,11+/m1/s1
**6**	O=C(OCC)[C@H]1C[C@@H]1[S@+]([O-])C2=CC=CC=C2	InChI=1S/C12H14O3S/c1-2-15-12(13)10-8-11(10)16(14)9-6-4-3-5-7-9/h3-7,10-11H,2,8H2,1H3/t10-,11-,16+/m0/s1
**7**	O=C(OCC)[C@@H]1C[C@@H]1[S@+]([O-])C2=CC=CC=C2	InChI=1S/C12H14O3S/c1-2-15-12(13)10-8-11(10)16(14)9-6-4-3-5-7-9/h3-7,10-11H,2,8H2,1H3/t10-,11+,16-/m1/s1
**8**	O=C(O)[C@H]1C[C@@H]1SC2=CC=CC=C2	InChI=1S/C10H10O2S/c11-10(12)8-6-9(8)13-7-4-2-1-3-5-7/h1-5,8-9H,6H2,(H,11,12)/t8-,9-/m0/s1
**9**	OC([C@@H]1C[C@@H]1SC2=CC=CC=C2)=O	InChI=1S/C10H10O2S/c11-10(12)8-6-9(8)13-7-4-2-1-3-5-7/h1-5,8-9H,6H2,(H,11,12)/t8-,9+/m1/s1
**10**	OC[C@H]1C[C@@H]1SC2=CC=CC=C2	InChI=1S/C10H12OS/c11-7-8-6-10(8)12-9-4-2-1-3-5-9/h1-5,8,10-11H,6-7H2/t8-,10+/m1/s1
**11**	OC[C@@H]1C[C@@H]1SC2=CC=CC=C2	InChI=1S/C10H12OS/c11-7-8-6-10(8)12-9-4-2-1-3-5-9/h1-5,8,10-11H,6-7H2/t8-,10-/m0/s1
**12**	O=C([O-])[C@H]1C[C@@H]1SC2=CC=CC=C2.[Na+]	InChI=1S/C10H10O2S.Na/c11-10(12)8-6-9(8)13-7-4-2-1-3-5-7;/h1-5,8-9H,6H2,(H,11,12);/q;+1/p-1/t8-,9-;/m0./s1
**13**	[O]C([C@@H]1C[C@@H]1SC2=CC=CC=C2)=O.[Na]	InChI=1S/C10H9O2S.Na/c11-10(12)8-6-9(8)13-7-4-2-1-3-5-7;/h1-5,8-9H,6H2;/t8-,9+;/m1./s1
**14a**	O=C(NCC1=CC=CC=C1)[C@H]2C[C@@H]2SC3=CC=CC=C3	InChI=1S/C17H17NOS/c19-17(18-12-13-7-3-1-4-8-13)15-11-16(15)20-14-9-5-2-6-10-14/h1-10,15-16H,11-12H2,(H,18,19)/t15-,16-/m0/s1
**14b**	O=C(N1CCOCC1)[C@H]2C[C@@H]2SC3=CC=CC=C3	InChI=1S/C14H17NO2S/c16-14(15-6-8-17-9-7-15)12-10-13(12)18-11-4-2-1-3-5-11/h1-5,12-13H,6-10H2/t12-,13-/m0/s1
**14c**	O=C(N1CCN(C)CC1)[C@H]2C[C@@H]2SC3=CC=CC=C3	InChI=1S/C15H20N2OS/c1-16-7-9-17(10-8-16)15(18)13-11-14(13)19-12-5-3-2-4-6-12/h2-6,13-14H,7-11H2,1H3/t13-,14-/m0/s1
**14d**	O=C(N1C(C=CC=C2)=C2CCC1)[C@H]3C[C@@H]3SC4=CC=CC=C4	InChI=1S/C19H19NOS/c21-19(16-13-18(16)22-15-9-2-1-3-10-15)20-12-6-8-14-7-4-5-11-17(14)20/h1-5,7,9-11,16,18H,6,8,12-13H2/t16-,18-/m0/s1
**15a**	O=C(NCC1=CC=CC=C1)[C@@H]2C[C@@H]2SC3=CC=CC=C3	InChI=1S/C17H17NOS/c19-17(18-12-13-7-3-1-4-8-13)15-11-16(15)20-14-9-5-2-6-10-14/h1-10,15-16H,11-12H2,(H,18,19)/t15-,16+/m1/s1
**15b**	O=C(N1CCOCC1)[C@@H]2C[C@@H]2SC3=CC=CC=C3	InChI=1S/C14H17NO2S/c16-14(15-6-8-17-9-7-15)12-10-13(12)18-11-4-2-1-3-5-11/h1-5,12-13H,6-10H2/t12-,13+/m1/s1
**15c**	O=C(N1CCN(C)CC1)[C@@H]2C[C@@H]2SC3=CC=CC=C3	InChI=1S/C15H20N2OS/c1-16-7-9-17(10-8-16)15(18)13-11-14(13)19-12-5-3-2-4-6-12/h2-6,13-14H,7-11H2,1H3/t13-,14+/m1/s1

**15d**	O=C(N1C(C=CC=C2)=C2CCC1)[C@@H]3C[C@@H]3SC4=CC=CC=C4	InChI=1S/C19H19NOS/c21-19(16-13-18(16)22-15-9-2-1-3-10-15)20-12-6-8-14-7-4-5-11-17(14)20/h1-5,7,9-11,16,18H,6,8,12-13H2/t16-,18+/m1/s1
**16c**	O=C(N1CCN(C)CC1)[C@H]2C[C@@H]2S(C3=CC=CC=C3)(=O)=O	InChI=1S/C15H20N2O3S/c1-16-7-9-17(10-8-16)15(18)13-11-14(13)21(19,20)12-5-3-2-4-6-12/h2-6,13-14H,7-11H2,1H3/t13-,14-/m0/s1
**17c**	O=C(N1CCN(C)CC1)[C@@H]2C[C@@H]2S(C3=CC=CC=C3)(=O)=O	InChI=1S/C15H20N2O3S/c1-16-7-9-17(10-8-16)15(18)13-11-14(13)21(19,20)12-5-3-2-4-6-12/h2-6,13-14H,7-11H2,1H3/t13-,14+/m1/s1
**16d**	O=C(N1C(C=CC=C2)=C2CCC1)[C@H]3C[C@@H]3S(C4=CC=CC=C4)(=O)=O	InChI=1S/C19H19NO3S/c21-19(20-12-6-8-14-7-4-5-11-17(14)20)16-13-18(16)24(22,23)15-9-2-1-3-10-15/h1-5,7,9-11,16,18H,6,8,12-13H2/t16-,18-/m0/s1
**17d**	O=C(N1C(C=CC=C2)=C2CCC1)[C@@H]3C[C@@H]3S(C4=CC=CC=C4)(=O)=O	InChI=1S/C19H19NO3S/c21-19(20-12-6-8-14-7-4-5-11-17(14)20)16-13-18(16)24(22,23)15-9-2-1-3-10-15/h1-5,7,9-11,16,18H,6,8,12-13H2/t16-,18+/m1/s1
**19a**	I[C@H]1C[C@@H]1C(OCC)=O	InChI=1S/C6H9IO2/c1-2-9-6(8)4-3-5(4)7/h4-5H,2-3H2,1H3/t4-,5-/m0/s1
**19b**	O[C@H](C1CCCC1)[C@H]2C[C@@H]2C(OCC)=O	InChI=1S/C12H20O3/c1-2-15-12(14)10-7-9(10)11(13)8-5-3-4-6-8/h8-11,13H,2-7H2,1H3/t9-,10-,11+/m0/s1
**19c**	O[C@H](C1=CC=CC=C1)[C@H]2C[C@@H]2C(OCC)=O	InChI=1S/C13H16O3/c1-2-16-13(15)11-8-10(11)12(14)9-6-4-3-5-7-9/h3-7,10-12,14H,2,8H2,1H3/t10-,11-,12+/m0/s1
**19d**	O=C(OCC)[C@H]1C[C@@H]1[C@H](O)C2=CN=CC=C2	InChI=1S/C12H15NO3/c1-2-16-12(15)10-6-9(10)11(14)8-4-3-5-13-7-8/h3-5,7,9-11,14H,2,6H2,1H3/t9-,10-,11+/m0/s1
**19e**	O=C(OCC)[C@H]1C[C@@H]1C2(COC2)O	InChI=1S/C9H14O4/c1-2-13-8(10)6-3-7(6)9(11)4-12-5-9/h6-7,11H,2-5H2,1H3/t6-,7-/m0/s1
**19f**	O=C(OCC)[C@H]1C[C@@H]1C(C2=CC=C(Cl)C=C2)(C3=CC=C(Cl)C=C3)O	InChI=1S/C19H18Cl2O3/c1-2-24-18(22)16-11-17(16)19(23,12-3-7-14(20)8-4-12)13-5-9-15(21)10-6-13/h3-10,16-17,23H,2,11H2,1H3/t16-,17-/m0/s1
**19g**	O=C(OCC)[C@H]1C[C@@H]1C(C2=CC=CC=N2)(C3=NC=CC=C3)O	InChI=1S/C17H18N2O3/c1-2-22-16(20)12-11-13(12)17(21,14-7-3-5-9-18-14)15-8-4-6-10-19-15/h3-10,12-13,21H,2,11H2,1H3/t12-,13-/m0/s1
**19h**	O=C(OCC)[C@H]1C[C@@H]1C(C2=CC=CC=C2)=O	InChI=1S/C13H14O3/c1-2-16-13(15)11-8-10(11)12(14)9-6-4-3-5-7-9/h3-7,10-11H,2,8H2,1H3/t10-,11-/m0/s1
**19i**	O=C(OCC)[C@H]1C[C@@H]1C(NC2=CC=CC=C2)=O	InChI=1S/C13H15NO3/c1-2-17-13(16)11-8-10(11)12(15)14-9-6-4-3-5-7-9/h3-7,10-11H,2,8H2,1H3,(H,14,15)/t10-,11-/m0/s1

**19j**	O=C(OCC)[C@H]1C[C@@H]1SC2=CC=C(OC)C=C2	InChI=1S/C13H16O3S/c1-3-16-13(14)11-8-12(11)17-10-6-4-9(15-2)5-7-10/h4-7,11-12H,3,8H2,1-2H3/t11-,12-/m0/s1
**19k**	O=C(OCC)[C@H]1C[C@@H]1C=O	InChI=1S/C7H10O3/c1-2-10-7(9)6-3-5(6)4-8/h4-6H,2-3H2,1H3/t5-,6+/m1/s1
**19l**	O=C(OCC)[C@H]1C[C@@H]1B2OC(C)(C)C(C)(C)O2	InChI=1S/C12H21BO4/c1-6-15-10(14)8-7-9(8)13-16-11(2,3)12(4,5)17-13/h8-9H,6-7H2,1-5H3/t8-,9-/m0/s1
**19m**	O=C(OCC)[C@H]1C[C@@H]1[Si](OCC)(OCC)OCC	InChI=1S/C12H24O5Si/c1-5-14-12(13)10-9-11(10)18(15-6-2,16-7-3)17-8-4/h10-11H,5-9H2,1-4H3/t10-,11-/m0/s1
**20a**	I[C@H]1C[C@H]1C(OCC)=O	InChI=1S/C6H9IO2/c1-2-9-6(8)4-3-5(4)7/h4-5H,2-3H2,1H3/t4-,5+/m1/s1
**20b**	O=C1O[C@@H](C2CCCC2)[C@H]3C[C@H]31	InChI=1S/C10H14O2/c11-10-8-5-7(8)9(12-10)6-3-1-2-4-6/h6-9H,1-5H2/t7-,8+,9-/m0/s1
**20c**	O=C1O[C@@H](C2=CC=CC=C2)[C@H]3C[C@H]31	InChI=1S/C11H10O2/c12-11-9-6-8(9)10(13-11)7-4-2-1-3-5-7/h1-5,8-10H,6H2/t8-,9+,10-/m0/s1
**20d**	O=C(OCC)[C@@H]1C[C@@H]1[C@H](O)C2=CN=CC=C2	InChI=1S/C12H15NO3/c1-2-16-12(15)10-6-9(10)11(14)8-4-3-5-13-7-8/h3-5,7,9-11,14H,2,6H2,1H3/t9-,10+,11+/m0/s1
**20e**	O=C1OC(CC)(CC)[C@H]2C[C@H]21	InChI=1S/C9H14O2/c1-3-9(4-2)7-5-6(7)8(10)11-9/h6-7H,3-5H2,1-2H3/t6-,7+/m1/s1
**20f**	O=C1OC(C2=CC=C(Cl)C=C2)(C3=CC=C(Cl)C=C3)[C@H]4C[C@H]41	InChI=1S/C17H12Cl2O2/c18-12-5-1-10(2-6-12)17(11-3-7-13(19)8-4-11)15-9-14(15)16(20)21-17/h1-8,14-15H,9H2/t14-,15+/m1/s1
**20g**	O=C(OCC)[C@@H]1C[C@@H]1C(C2=NC=CC=C2)(O)C3=NC=CC=C3	InChI=1S/C17H18N2O3/c1-2-22-16(20)12-11-13(12)17(21,14-7-3-5-9-18-14)15-8-4-6-10-19-15/h3-10,12-13,21H,2,11H2,1H3/t12-,13+/m1/s1
**20g'**	O=C1OC(C2=CC=CC=N2)(C3=CC=CC=N3)[C@H]4C[C@H]41	InChI=1S/C15H12N2O2/c18-14-10-9-11(10)15(19-14,12-5-1-3-7-16-12)13-6-2-4-8-17-13/h1-8,10-11H,9H2/t10-,11+/m1/s1
**20h**	O=C(OCC)[C@@H]1C[C@@H]1C(C2=CC=CC=C2)=O	InChI=1S/C13H14O3/c1-2-16-13(15)11-8-10(11)12(14)9-6-4-3-5-7-9/h3-7,10-11H,2,8H2,1H3/t10-,11+/m0/s1
**20l**	O=C(OCC)[C@@H]1C[C@@H]1B2OC(C)(C)C(C)(C)O2	InChI=1S/C12H21BO4/c1-6-15-10(14)8-7-9(8)13-16-11(2,3)12(4,5)17-13/h8-9H,6-7H2,1-5H3/t8-,9+/m1/s1
**21a**	O=C(OCC)[C@H]1C[C@@H]1C2=CC=CC=C2	InChI=1S/C12H14O2/c1-2-14-12(13)11-8-10(11)9-6-4-3-5-7-9/h3-7,10-11H,2,8H2,1H3/t10-,11+/m1/s1
**22a**	O=C(OCC)[C@@H]1C[C@@H]1C2=CC=CC=C2	InChI=1S/C12H14O2/c1-2-14-12(13)11-8-10(11)9-6-4-3-5-7-9/h3-7,10-11H,2,8H2,1H3/t10-,11-/m1/s1

**21b**	O=C(OCC)[C@H]1C[C@@H]1C2=CC=C(Cl)C=C2	InChI=1S/C12H13ClO2/c1-2-15-12(14)11-7-10(11)8-3-5-9(13)6-4-8/h3-6,10-11H,2,7H2,1H3/t10-,11+/m1/s1
**21c**	O=C(OCC)[C@H]1C[C@@H]1C2=CC=C(OC)C=C2	InChI=1S/C13H16O3/c1-3-16-13(14)12-8-11(12)9-4-6-10(15-2)7-5-9/h4-7,11-12H,3,8H2,1-2H3/t11-,12+/m1/s1
**21d**	O=C(OCC)[C@H]1C[C@@H]1/C=C/C2=CC=CC=C2	InChI=1S/C14H16O2/c1-2-16-14(15)13-10-12(13)9-8-11-6-4-3-5-7-11/h3-9,12-13H,2,10H2,1H3/b9-8+/t12-,13-/m0/s1
**21e**	O=C(OCC)[C@H]1C[C@@H]1C2=NC=CC=C2	InChI=1S/C11H13NO2/c1-2-14-11(13)9-7-8(9)10-5-3-4-6-12-10/h3-6,8-9H,2,7H2,1H3/t8-,9-/m0/s1
**21f**	O=C(OCC)[C@H]1C[C@@H]1C2=NC=CC=N2	InChI=1S/C10H12N2O2/c1-2-14-10(13)8-6-7(8)9-11-4-3-5-12-9/h3-5,7-8H,2,6H2,1H3/t7-,8-/m0/s1
**21g**	O=C(OCC)[C@H]1C[C@@H]1C2=CN(C(OC(C)(C)C)=O)C3=C2C=CC=C3	InChI=1S/C19H23NO4/c1-5-23-17(21)14-10-13(14)15-11-20(18(22)24-19(2,3)4)16-9-7-6-8-12(15)16/h6-9,11,13-14H,5,10H2,1-4H3/t13-,14-/m0/s1
**22h**	O=C(OCC)[C@@H]1C[C@@H]1C2=CN(S(C3=CC=CC=C3(=O)=O)C4=C2C=CC=C4	InChI=1S/C20H19NO4S/c1-2-25-20(22)17-12-16(17)18-13-21(19-11-7-6-10-15(18)19)26(23,24)14-8-4-3-5-9-14/h3-11,13,16-17H,2,12H2,1H3/t16-,17+/m0/s1
**23**	[O]C([C@H]1C[C@@H]1C2=NC=CC=C2)=O.[Na]	InChI=1S/C9H8NO2.Na/c11-9(12)7-5-6(7)8-3-1-2-4-10-8;/h1-4,6-7H,5H2;/t6-,7-;/m0./s1
**24**	O=C(N1CCCC1)[C@H]2C[C@@H]2C3=NC=CC=C3	InChI=1S/C13H16N2O/c16-13(15-7-3-4-8-15)11-9-10(11)12-5-1-2-6-14-12/h1-2,5-6,10-11H,3-4,7-9H2/t10-,11-/m0/s1
**25**	OC[C@@H]1C[C@@H]1C2=CN(S(C3=CC=CC=C3)(=O)=O)C4=C2C=CC=C4	InChI=1S/C18H17NO3S/c20-12-13-10-16(13)17-11-19(18-9-5-4-8-15(17)18)23(21,22)14-6-2-1-3-7-14/h1-9,11,13,16,20H,10,12H2/t13-,16-/m0/s1

Cyclopropanation of phenyl vinyl sulfide: initial catalyst screening

During optimization of the cyclopropanation of phenyl vinyl sulfide and ethyl diazoacetate a variety of transition metals were investigated (Table S1). CuOTf was a good catalyst and gave improved yields on addition of a BOX ligand (BOX1). However, the reaction could not be further optimized to give above approximately 50% yield (entry 10). Therefore, the cyclopropanation was reoptimized for the Co^{II}(salen)-type catalyst **3**, which gave excellent yields and a convenient reaction set-up (Table S1 entries 15-21 and Table 1 in manuscript). For all reactions the dr (*trans*:*cis*) of product cyclopropanes was between 57:43-38:62.

Table S1. Optimization of the transition-metal catalyzed cyclopropanation of phenyl vinyl sulfide and ethyl diazoacetate

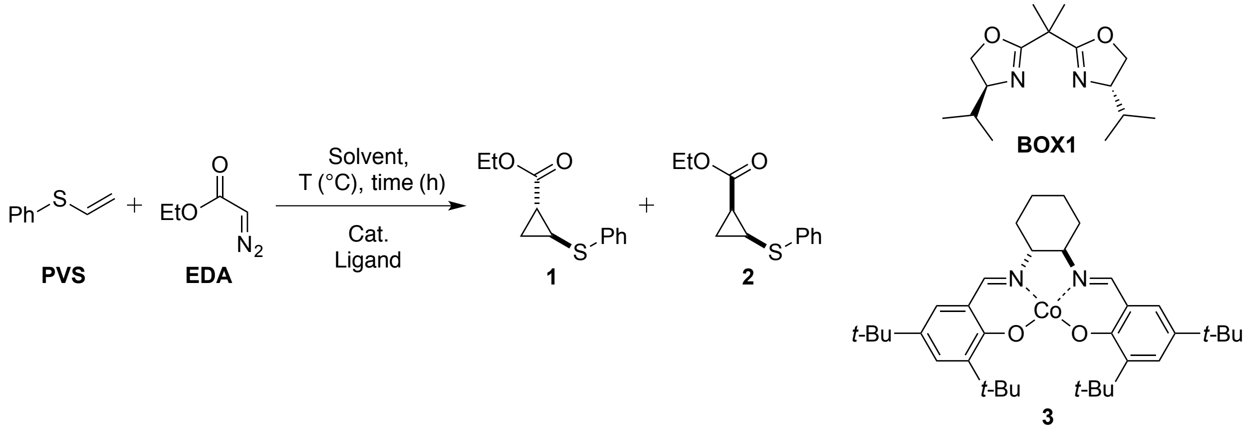

Entry	Catalyst	Solvent	Ligand	T (°C)	Yield (%)
1[a]	$Rh(OAc)_2$	CH_2Cl_2	–	30	0
2[b]	$PdCl_2$	CH_2Cl_2	–	30	0
3[b]	$Pd(OAc)_2$	CH_2Cl_2	–	30	0
4[b]	$Cu(acac)_2$	CH_2Cl_2	–	30	5
5[b]	$Cu(OTf)_2$	CH_2Cl_2	–	30	4
6[b]	Cu(OTf)	CH_2Cl_2	–	30	33
7[c]	Cu(OTf)	Toluene	–	30	12[f]
8[c]	Cu(OTf)	THF	–	30	26[f]
9[c]	Cu(OTf)	$CHCl_3$	–	30	41[f]
10[d]	Cu(OTf)	$CHCl_3$	BOX1	30	46-54
11[d]	CuCl	$CHCl_3$	BOX1	30	4[f]
12[d]	CuBr	$CHCl_3$	BOX1	30	0[f]
13[d]	CuI	$CHCl_3$	BOX1	30	8[f]
14[d]	$Cu(MeCN)_4BF_4$	$CHCl_3$	BOX1	30	36[f]
15[e]	**3**	CH_2Cl_2	–	40	40[f]
16[e]	**3**	$CHCl_3$	–	40	13[f]
17[e]	**3**	TBME	–	40	69[f]
18[e]	**3**	Neat	–	40	93[f]
19[e]	**3**	H_2O	–	40	100[f]
20[e]	**3**	H_2O	–	30	92[f]
21[e]	**3**	H_2O	–	20	57[f]

[a] Cat. (0.5 mol%) added to flame-dried vial, sealed and flushed with $Ar_{(g)}$. A solution of phenyl vinyl sulfide (131 μL, 1.0 mmol) in CH_2Cl_2 (7.0 mL, 0.14 M) was added and warmed with stirring to 30 °C. A solution of ethyl diazoacetate (118 μL, 1.0 mmol) in CH_2Cl_2 (118 μL, 8.5 M) was added over 8 h and then stirred for a further 17 h. [b] Same method as for [a] but stirred for a total of 48 h. [c] CuOTf·toluene complex (2.6 mg, 0.5 mol%) was added to a flame-dried vial, the vial sealed and flushed with $Ar_{(g)}$. A solution of phenyl vinyl sulfide (131 μL, 1.0 mmol) in solvent (3.3 mL, 0.30 M) was added and warmed with stirring to 30 °C. A solution of ethyl diazoacetate (118 μL, 1.0 mmol) in solvent (3.7 mL, 0.27 M) was added over 1.5 h and then stirred for a further 22 h 30 min. [d] Ligand (0.55 mol%) was added to a flame-dried vial and flushed with $Ar_{(g)}$. Catalyst (0.5 mol%) was added, the vial sealed and flushed with $Ar_{(g)}$. $CHCl_3$ (1.85 mL) was added and the mixture stirred at 30 °C for 1 h. Phenyl vinyl sulfide (66 μL, 0.5 mmol) was added to the solution. A solution of ethyl diazoacetate (118 μL, 1.0 mmol) in $CHCl_3$ (1.65 mL, 0.61 M) was added over 1.5 h and then stirred for a further 22 h 30 min. [e] Method used is the same as that detailed for the synthesis of **1** and **2** on page S14. [f] Yield was determined using ^{1}H NMR spectroscopy through comparison to an internal standard (dibenzyl ether or 1,3,5-trimethoxybenzene).

Cyclopropanation of phenyl vinyl sulfide: product purification

With the larger scale cyclopropanation reactions, using EDA and PVS with cobalt catalyst **3**, flash chromatography was not successful in separating cyclopropanes **1** and **2** from a catalyst derived impurity. This impurity coeluted with the products in all eluent systems tested. An oxidative work-up was developed that allowed removal of this impurity through simple filtration. Initial observations were made on the addition of *iso*-hexane to the reaction mixture on small scale, leading to a change in the nature of the impurity so it could be easily removed. This was ascribed to the dissolved O_2 in the *iso*-hexane likely forming a peroxo-bridged dimeric Co-species, resulting in a very deep brown mixture.[1] On larger scales, more O_2 was required for the same effect. Various solvents were tested for effectiveness in oxidation of the catalyst, and hence facile removal, by adding O_2 (from either an O_2 or compressed air cylinder) for approximately 15 minutes. The resulting mixture was then filtered through a pad of silica (size dependant upon the amount of salen-based material being removed), washing with CH_2Cl_2. *i*-Hexane was chosen as the most effective solvent as it has a relatively high oxygen permeability,[2] it solubilises the Co-salen-based species, and allows the mixture to be directly filtered through a silica pad without the solvent eluting impurities.

Table S2. Solvent effects on the oxidation of **3** with $O_{2(g)}$.

Entry	Solvent	Oxidation observed through colour change?	Time
**1**	***i*-Hexane**	**Yes**	**<10 s**
2	*n*-Hexane	Yes	1 min
3	Et_2O	Yes	2 min
4	EtOAc	No	–
5	TBME	No	–
6	CH_2Cl_2	Yes	2 h
7	Toluene	No	–
8	*i*-Propanol	No	–
9	Acetone	No	–
10	THF	Yes	<10s
11	2-MeTHF	Yes	<10s
12	DMF	Yes	<10s

Calculated fragment and lead-like properties for cyclopropane derivatives

The compounds synthesized in this work were intended to fit desirable criteria for fragment or lead-like compounds for drug discovery.

Guidelines for fragments as defined by Congreve and co-workers.[3]

- $M_W < 300$ Da
- clogP < 3
- Hydrogen bond donors (HBD) ≤ 3
- Hydrogen bond acceptors (HBA) ≤ 3

Klebe and co-workers proposed that HBA ≤ 6 is appropriate for fragments to allow for fragment growing/merging due to appropriate functional groups frequently display properties as hydrogen bond acceptors.[4]

Guidelines for lead-like compounds as defined by Churcher and co-workers.[5]

- $200 < M_W < 350$ Da
- –1 ≤ clogP ≤ 3

The polar surface area (PSA) of a compound is important to the permeability through biological membranes, and has been correlated with the likelihood of *in vivo* toxicity (ClogP < 3 and PSA > 75 prefered).[6] Finally, recently less planar compounds, more "3-dimensional" as measured by Fsp^3, have been investigated to increase the shape diversity of libraries, and with potential benefits of improved developability and linked to reduced biological promiscuity.[7]

Molecular properties for the prepared compounds are below (Figure **1**):

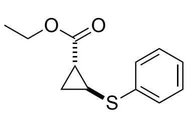

1
M_W = 222
AlogP = 3.21
HBD/HBA = 0/2
Fsp^3 = 0.416
PSA = 26.30 Å^2

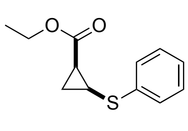

2
M_W = 222
AlogP = 3.21
HBD/HBA = 0/2
Fsp^3 = 0.416
PSA = 26.30 Å^2

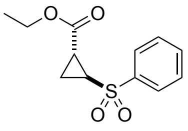

4
M_W = 254
AlogP = 1.24
HBD/HBA = 0/4
Fsp^3 = 0.417
PSA = 60.44 Å^2

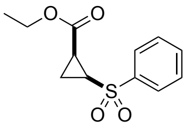

5
M_W = 254
AlogP = 1.24
HBD/HBA = 0/4
Fsp^3 = 0.417
PSA = 60.44 Å^2

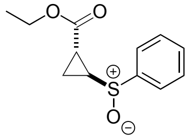

6
M_W = 238
AlogP = 0.82
HBD/HBA = 0/3
Fsp^3 = 0.42
PSA = 43.37 Å^2

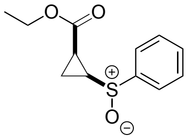

7
M_W = 238
AlogP = 0.82
HBD/HBA = 0/3
Fsp^3 = 0.42
PSA = 43.37 Å^2

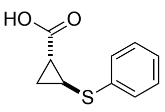

8
M_W = 194
AlogP = 2.44
HBD/HBA = 1/2
Fsp^3 = 0.300
PSA = 37.30 Å^2

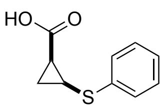

9
M_W = 194
AlogP = 2.44
HBD/HBA = 1/2
Fsp^3 = 0.300
PSA = 37.30 Å^2

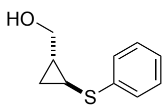

10
M_W = 180
AlogP = 2.74
HBD/HBA = 1/1
Fsp^3 = 0.400
PSA = 20.23 Å^2

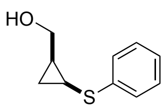

11
M_W = 180
AlogP = 2.74
HBD/HBA = 1/1
Fsp^3 = 0.400
PSA = 20.23 Å^2

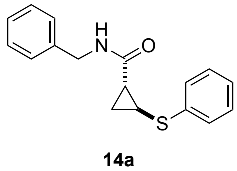

14a
M_W = 283
AlogP = 3.51
HBD/HBA = 1/2
Fsp^3 = 0.235
PSA = 29.10 Å^2

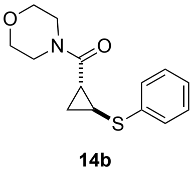

14b
M_W = 263
AlogP = 1.98
HBD/HBA = 0/3
Fsp^3 = 0.50
PSA = 29.54 Å^2

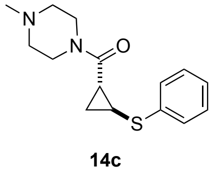

14c
M_W = 276
AlogP = 1.75
HBD/HBA = 0/3
Fsp^3 = 0.53
PSA = 23.55 Å^2

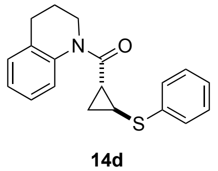

14d
M_W = 309
AlogP = 4.08
HBD/HBA = 0/2
Fsp^3 = 0.32
PSA = 20.31 Å^2

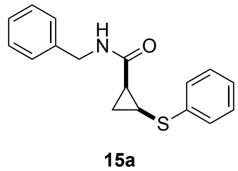

15a
M_W = 283
AlogP = 3.51
HBD/HBA = 1/2
Fsp^3 = 0.235
PSA = 29.10 Å^2

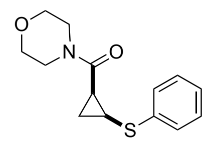

15b
M_W = 263
AlogP = 1.98
HBD/HBA = 0/3
Fsp^3 = 0.50
PSA = 29.54 Å^2

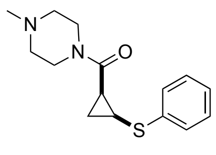

15c
M_W = 276
AlogP = 1.75
HBD/HBA = 0/3
Fsp^3 = 0.53
PSA = 23.55 Å^2

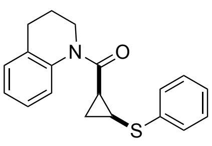

15d
M_W = 309
AlogP = 4.08
HBD/HBA = 0/2
Fsp^3 = 0.32
PSA = 20.31 Å^2

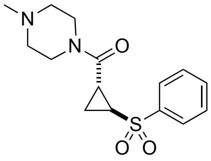

16c
M_W = 308
AlogP = 0.16
HBD/HBA = 0/5
Fsp^3 = 0.53
PSA = 57.69 Å^2

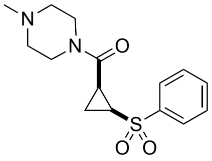

17c
M_W = 308
AlogP = 0.16
HBD/HBA = 0/5
Fsp^3 = 0.53
PSA = 57.69 Å^2

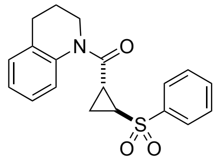

16d
M_W = 341
AlogP = 2.00
HBD/HBA = 0/4
Fsp^3 = 0.32
PSA = 54.45 Å^2

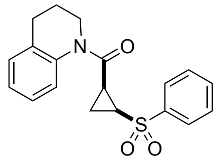

17d
M_W = 341
AlogP = 2.00
HBD/HBA = 0/4
Fsp^3 = 0.32
PSA = 54.45 Å^2

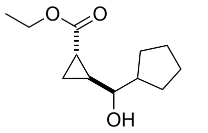

19b
M_W = 240
AlogP = 1.91
HBD/HBA = 1/3
Fsp^3 = 0.92
PSA = 46.53 Å^2

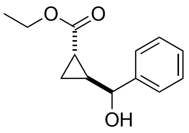

19c
M_W = 220
AlogP = 2.09
HBD/HBA = 1/3
Fsp^3 = 0.46
PSA = 46.53 Å^2

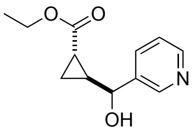

19d
M_W = 221
AlogP = 1.08
HBD/HBA = 1/4
Fsp^3 = 0.50
PSA = 59.42 Å^2

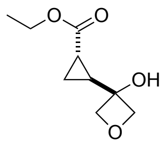

19e
M_W = 186
AlogP = −0.16
HBD/HBA = 1/4
Fsp^3 = 0.89
PSA = 55.76 Å^2

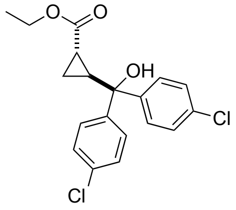

19f
M_W = 365
AlogP = 5.05
HBD/HBA = 1/3
Fsp^3 = 0.32
PSA = 46.53 Å^2

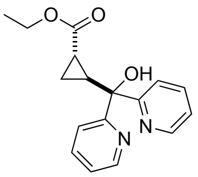

19g
M_W = 298
AlogP = 1.98
HBD/HBA = 1/5
Fsp^3 = 0.35
PSA = 72.31 Å^2

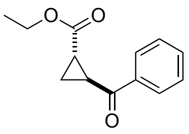

19h
M_W = 218
AlogP = 2.19
HBD/HBA = 0/3
Fsp^3 = 0.38
PSA = 43.37 Å^2

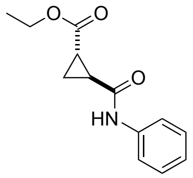

19i
M_W = 233
AlogP = 1.81
HBD/HBA = 1/4
Fsp^3 = 0.38
PSA = 55.40 Å^2

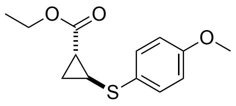

19j
M_W = 252
AlogP = 3.53
HBD/HBA = 0/3
Fsp^3 = 0.46
PSA = 35.53 Å^2

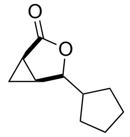

20b
M_W = 166
AlogP = 2.04
HBD/HBA = 0/2
Fsp^3 = 0.90
PSA = 26.30 Å^2

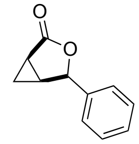

20c
M_W = 174
AlogP = 1.90
HBD/HBA = 0/2
Fsp^3 = 0.36
PSA = 26.30 Å^2

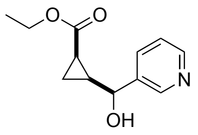

20d
M_W = 221
AlogP = 1.08
HBD/HBA = 1/4
Fsp^3 = 0.50
PSA = 59.42 Å^2

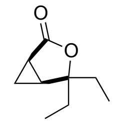

20e
M_W = 154
AlogP = 2.25
HBD/HBA = 0/2
Fsp^3 = 0.89
PSA = 26.30 Å^2

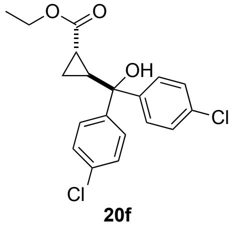

20f
M_W = 365
AlogP = 5.05
HBD/HBA = 1/3
Fsp^3 = 0.32
PSA = 46.53 Å^2

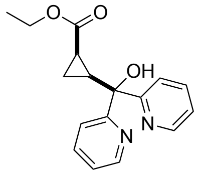

20g
M_W = 298
AlogP = 1.98
HBD/HBA = 1/5
Fsp^3 = 0.35
PSA = 72.31 Å^2

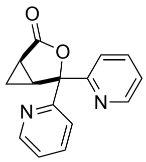

20g'
M_W = 252
AlogP =2.13
HBD/HBA = 0/4
Fsp^3 = 0.27
PSA = 52.08 Å^2

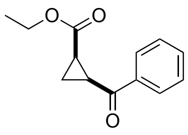

20h
M_W = 218
AlogP = 2.19
HBD/HBA = 0/3
Fsp^3 = 0.38
PSA = 43.37 Å^2

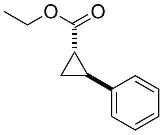

21a
M_W = 190
AlogP = 2.95
HBD/HBA = 0/2
Fsp^3 = 0.42
PSA = 26.30 Å^2

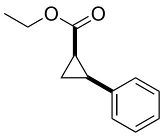

22a
M_W = 190
AlogP = 2.95
HBD/HBA = 0/2
Fsp^3 = 0.42
PSA = 26.30 Å^2

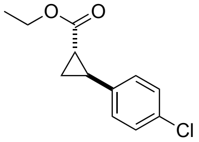

21b
M_W = 225
AlogP = 3.45
HBD/HBA = 0/2
Fsp^3 = 0.42
PSA = 26.30 Å^2

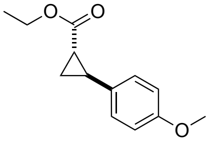

21c
M_W = 220
AlogP = 3.20
HBD/HBA = 0/3
Fsp^3 = 0.46
PSA = 35.53 Å^2

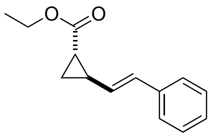

21d
M_W = 216
AlogP = 3.81
HBD/HBA = 0/2
Fsp^3 = 0.36
PSA = 26.30 Å^2

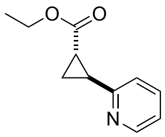

21e
M_W = 191
AlogP = 1.31
HBD/HBA = 0/3
Fsp^3 = 0.46
PSA = 39.19 Å^2

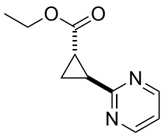

21f
M_W = 192
AlogP = 0.56
HBD/HBA = 0/4
Fsp^3 = 0.5
PSA = 52.08 Å^2

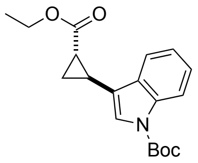

21g
M_W = 329
AlogP = 4.39
HBD/HBA = 0/5
Fsp^3 = 0.47
PSA = 57.53 Å^2

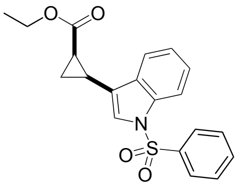

22h
M_W = 369
AlogP = 3.43
HBD/HBA = 0/5
Fsp^3 = 0.25
PSA = 65.37 Å^2

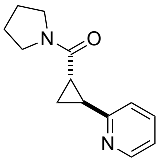

24
M_W = 216
AlogP = 1.35
HBD/HBA = 0/3
Fsp^3 = 0.54
PSA = 33.20 Å^2

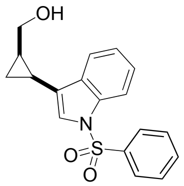

25
M_W = 327
AlogP = 2.62
HBD/HBA = 1/4
Fsp^3 = 0.22
PSA = 59.30 Å^2

Figure S1: Physicochemical properties for the synthesized compounds deemed relevant to drug discovery

Molecular properties were calculated using LLAMA (Lead-Likeness and Molecular Analysis) software, available freely online at https://llama.leeds.ac.uk.[8]

Virtual scaffold decoration and LLAMA compound analysis

The LLAMA software was used to perform a virtual decoration of the synthesized cyclopropyl compounds. The 56 compounds (all 50 compounds shown in Figure **S1** plus **19a**, **19k**, **19l**, **19m**, **20a** and **20l**) were utilized as scaffolds, along with the 44 reagents from the ‘LLAMA Default’ reactant set (Figure **S2**).

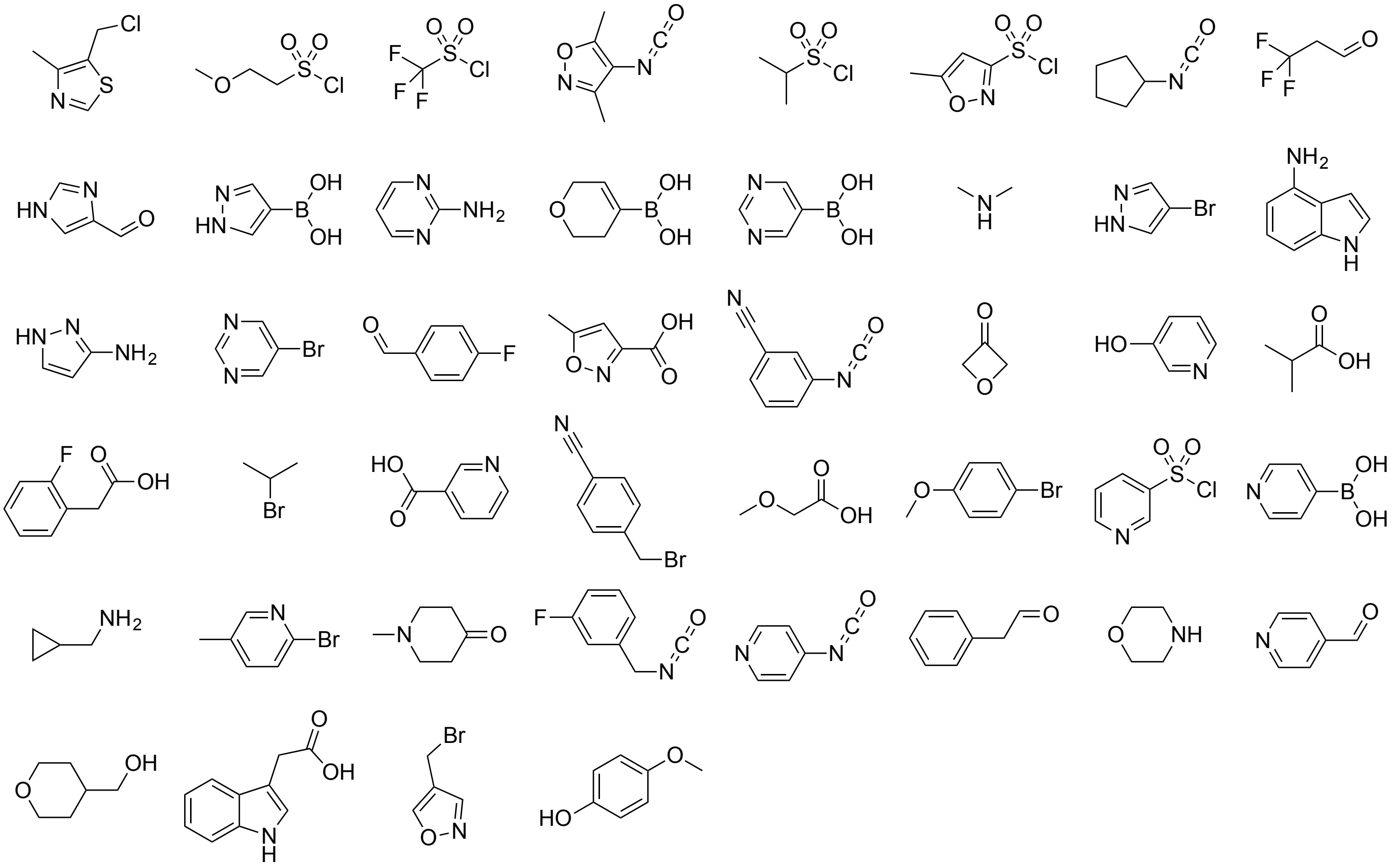

Figure S2: ‘LLAMA Default’ reaction set

The following reactions were enabled for the virtual scaffold functionalization:
BOC deprotection, reductive amination, Suzuki-Miyaura cross-coupling, Buchwald-Hartwig amination, sulfonamide formation, urea formation, alcohol alkylation, carbamate formation, secondary amide alkylation, secondary amide arylation, amide formation, alcohol arylation, urea alkylation, urea arylation, esterification and ester hydrolysis.

The reaction for ester hydrolysis to the corresponding carboxylic acid was not present in the default reaction library and so was added *via* ‘Advanced settings > Add a new reaction to the library’ and using the below SMARTS code as the reaction description:

```
[*;$([CX3](=O)):1][OX2H0][#6]>>[*:1][OX2H1]
```

Elaboration of the scaffolds with LLAMA elaboration generated 1187 compounds. These are represented below through plots of AlogP against molecular weight (Graph **S1**), and a PMI plot (Graph **S2**) to indicate the molecular shapes.

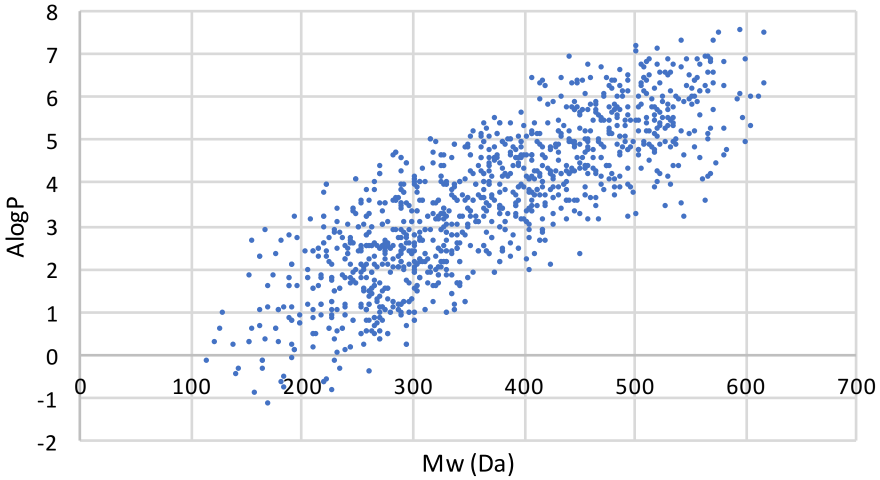

Graph S1: AlogP vs. M_W for the decorated cyclopropane-containing compounds

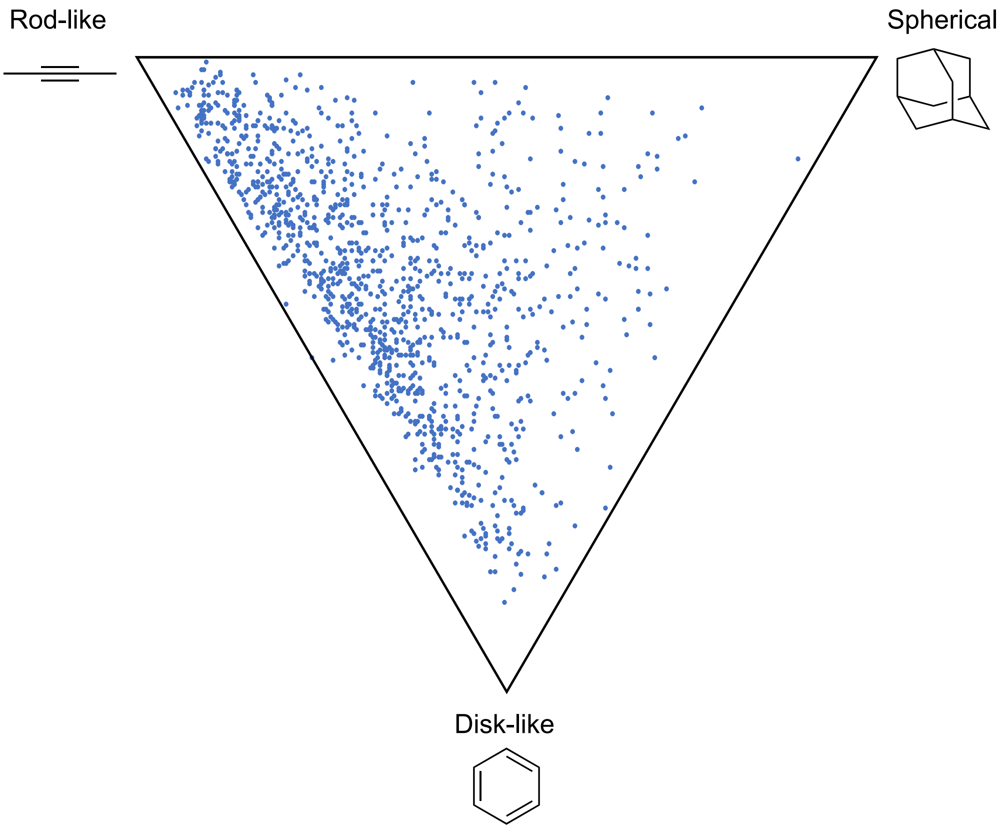

Graph S2: PMI plot for the decorated cyclopropane-containing compounds

Experimental Details and Charaterization Data

Synthesis of 1 and 2 by Co^{II}-catalyzed cyclopropanation

(*E*)-Ethyl 2-(phenylsulfanyl)cyclopropane-1-carboxylate (1) and
(*Z*)-Ethyl 2-(phenylsulfanyl)cyclopropane-1-carboxylate (2)

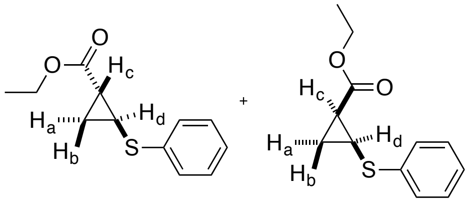

A flask containing (±)*N,N′*-bis(3,5-di-*tert*-butylsalicylidene)-1,2-cyclohexanediaminocobalt(II) (1.21 g, 2.0 mmol, 5 mol%) was flushed with $Ar_{(g)}$ for 15 min. Water (80 mL, degassed with $Ar_{(g)}$), phenyl vinyl sulfide (7.84 mL, 60 mmol, 1.5 equiv) and ethyl diazoacetate (4.84 mL, containing 13 wt% CH_2Cl_2, 40 mmol, 1.0 equiv) were added and the mixture was warmed to 40 °C. After stirring at 40 °C for 24 h, the mixture was cooled to rt. *i*-Hexane (20 mL) was added and air bubbled through the stirring mixture for 15 min. Filtration of the mixture through a pad of silica, washing with CH_2Cl_2, followed by purification by flash column chromatography (15:1 pentane:Et_2O) gave (*E*)-ethyl 2-thiophenylcyclopropane carboxylate **1** (3.66 g, 41%) as a yellow oil followed by (*Z*)-ethyl 2-thiophenylcyclopropane carboxylate **2** (4.25 g, 48%) as a yellow oil.

<u>(E)-Ethyl 2-(phenylsulfanyl)cyclopropane-1-carboxylate (1)</u>
R_f = 0.54 (4:1 *n*-hexane:Et_2O). IR (film)/cm^{-1} 3078 (CH), 3059 (CH), 2981 (CH), 2941 (CH), 2906 (CH), 1725 (C=O), 1584, 1480, 1380. ^{1}H NMR (400 MHz, $CDCl_3$) δ 7.35–7.28 (m, 4 H, 4 × Ph-H), 7.21–7.17 (m, 1 H, Ph-H), 4.25–4.13 (m, 2 H, C$H_2$$CH_3$), 2.77 (ddd, *J* = 8.2, 5.6, 3.6 Hz, 1 H, H_c), 1.92 (ddd, *J* = 8.8, 5.4, 3.6 Hz, 1 H, H_d), 1.67 (ddd, *J* = 8.2, 5.4, 4.9 Hz, 1 H, H_b), 1.29 (t, *J* = 7.1 Hz, 3 H, CH_3), 1.25 (ddd, *J* = 8.8, 5.6, 4.9 Hz, 1 H, H_a). ^{13}C NMR (101 MHz, $CDCl_3$) δ 172.3 (C=O), 136.8 (Ph-C quat), 128.9 (2 × Ph-C), 127.2 (2 × Ph-C), 125.7 (Ph-C), 61.0 (CH_2CH_3), 24.2 (C(H_d)), 22.3 (C(H_c)), 17.2 (C(H_a)(H_b)), 14.2 (CH_3). HRMS (ES) *m/z* Calcd for $C_{12}H_{15}O_2S^+$ $[M+H]^+$: 223.0793; Found: 223.0795.

<u>(Z)-Ethyl 2-(phenylsulfanyl)cyclopropane-1-carboxylate (2)</u>
R_f = 0.35 (4:1 *n*-hexane:Et_2O). IR (film)/cm^{-1} 3074 (CH), 3059 (CH), 2981 (CH), 2937 (CH), 2906 (CH), 2874, 1728 (C=O), 1585, 1480, 1380. ^{1}H NMR (400 MHz, $CDCl_3$) δ 7.37–7.34 (m, 2 H, 2 × Ph-H), 7.30–7.25 (m, 2 H, 2 × Ph-H), 7.17–7.13 (m, 1 H, Ph-H), 4.06 (q, *J* = 7.1 Hz, 2 H, C$H_2$$CH_3$), 2.70 (ddd, *J* = 7.8, 7.8, 6.7 Hz, 1 H, H_c), 2.25 (ddd, *J* = 7.8, 7.8, 6.7 Hz, 1 H, H_d), 1.49–1.45 (m, 2 H, H_a + H_b), 1.11 (t, *J* = 7.1 Hz, 3 H, CH_3). ^{13}C NMR (101 MHz, $CDCl_3$) δ 169.6 (C=O), 137.1 (Ph-C quat), 128.7 (2 × Ph-C), 127.5 (2 × Ph-C), 125.5 (Ph-C), 60.8 (CH_2CH_3), 22.07 (C(H_d)), 22.05 (C(H_c)), 14.1 (CH_3), 13.2 (C(H_a)(H_b)). HRMS (ES) *m/z* Calcd for $C_{12}H_{15}O_2S^+$ $[M+H]^+$: 223.0793; Found: 223.0801.

These compounds display characteristic *J*-values which have been considered in all assignments:[9]

(*E*)-Ethyl 2-(phenylsulfanyl)
cyclopropane-1-carboxylate (**1**)

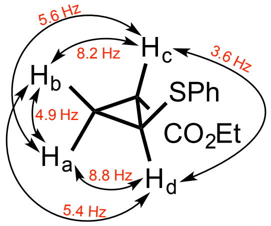

(*Z*)-Ethyl 2-(phenylsulfanyl)
cyclopropane-1-carboxylate (**2**)

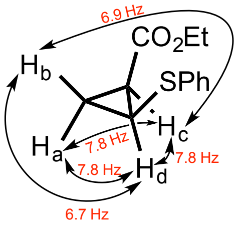

$^2J_{gem}$ = 4.5–7.0 Hz
$^3J_{trans}$ = 4.5–7.5 Hz
$^3J_{cis}$ = 7.5–11.0 Hz

Synthesis of 4–7 through sulfide oxidation

(*E*)-Ethyl-2-(benzenesulfonyl)cyclopropane-1-carboxylate (4)

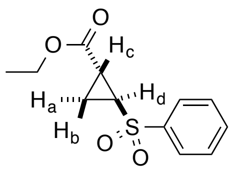

*m*CPBA (62 mg, 0.36 mmol, 2.5 equiv) was added to a solution of (*E*)-ethyl 2-thiophenylcyclopropane carboxylate **1** (32 mg, 0.14 mmol, 1.0 equiv) in CH_2Cl_2 (5.0 mL) and the mixture stirred at 25 °C for 24 h. Water (15 mL) and sat. aq. $NaHCO_3$ (15 mL) were added and the mixture was extracted with CH_2Cl_2 (3 × 15 mL). The combined organic phases were washed with brine (15 mL), dried ($MgSO_4$), filtered and concentrated under reduced pressure to give sulfone **4** (39 mg, quant.) as a pale yellow crystalline solid. R_f = 0.45 (1:2 *n*-hexane:Et_2O). mp = 53–56 °C. IR (film)/cm^{-1} 3074 (CH), 3041 (CH), 2991 (CH), 2964 (CH), 2906 (CH), 1721 (C=O), 1449, 1306 (S=O), 1147 (S=O). ^{1}H NMR (400 MHz, $CDCl_3$) δ 7.93–7.90 (m, 2 H, 2 × Ph-H), 7.68 (t, *J* = 7.4 Hz, 1 H, Ph-H), 7.59 (t, *J* = 7.6 Hz, 2 H, 2 × Ph-H), 4.13 (q, *J* = 7.1 Hz, 2 H, C*H*$_2$CH$_3$), 2.98 (ddd, *J* = 8.8, 5.9, 4.1 Hz, 1 H, H_c), 2.52 (ddd, *J* = 9.7, 5.8, 4.1 Hz, 1 H, H_d), 1.73 (ddd, *J* = 9.6, 5.5, 5.5 Hz, 1 H, H_a), 1.55 (ddd, *J* = 8.8, 5.7, 5.7 Hz, 1 H, H_b), 1.25 (t, *J* = 7.1 Hz, 3 H, CH_3). ^{13}C NMR (101 MHz, $CDCl_3$) δ 170.2 (C=O), 139.7 (Ph-C quat.), 133.9 (Ph-C), 129.4 (2 × Ph-C), 127.7 (2 × Ph-C), 61.6 (CH_2), 40.3 (C(H_c)), 20.1 (C(H_d)), 14.0 (CH_3), 13.3 (C(H_a)(H_b)). HRMS (EI) *m/z* Calcd for $C_{12}H_{14}O_4S^+$ [M]$^+$: 254.0613; Found: 254.0619.

(*Z*)-Ethyl-2-(benzenesulfonyl)cyclopropane-1-carboxylate (5)

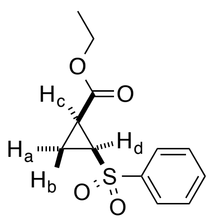

*m*CPBA (56 mg, 0.33 mmol, 2.5 equiv.) was added to a solution of (*Z*)-ethyl 2-thiophenylcyclopropane carboxylate **2** (29 mg, 0.13 mmol, 1.0 equiv) in CH_2Cl_2 (5.0 mL) and the mixture stirred at 25 °C for 25 h. H_2O (10 mL) and sat. aq. $NaHCO_3$ (15 mL) were added and the mixture was extracted with CH_2Cl_2 (3 × 15 mL). The combined organic phases were washed with brine (20 mL), dried ($MgSO_4$), filtered and concentrated under reduced pressure to give sulfone **5** (33 mg, quant.) as a pale yellow crystalline solid. R_f = 0.33 (1:2 *n*-hexane:Et_2O). mp = 100–102 °C. IR (film) / cm^{-1} 3098 (CH), 3063 (CH), 3042 (CH), 2984 (CH), 2937 (CH), 2910 (CH), 1733, 1447, 1324 S=O), 1150 (S=O). ^{1}H NMR (400 MHz, $CDCl_3$) δ 7.95–7.93 (m, 2 H, 2 × Ph-H), 7.68–7.64 (m, 1 H, Ph-H), 7.59–7.55 (m, 2 H, 2 × Ph-H), 4.28–4.14 (m, 2 H, C*H*$_2$CH$_3$), 2.81 (ddd, *J* = 8.8, 8.8, 6.6 Hz, 1 H, H_c), 2.25 (ddd, *J* = 8.6, 8.6, 7.5 Hz, 1 H, H_d), 2.08 (ddd, *J* = 7.4, 6.2, 6.2 Hz, 1 H, H_b), 1.45 (ddd, *J* = 8.5, 8.5, 5.9 Hz, 1 H, H_a), 1.29 (t, *J* = 7.1 Hz, 3 H, CH_3). ^{13}C NMR (101 MHz, $CDCl_3$) 166.9 (C=O), 140.4 (Ph-C quat.), 133.6 (Ph-C), 129.1 (2 × Ph-C), 127.7 (2 × Ph-C), 61.8 (CH_2), 39.6 (C(H_c)), 23.5 (C(H_d)), 13.9 (CH_3), 10.4 (C(H_a)(H_b)). HRMS (ES) *m/z* Calcd for $C_{12}H_{15}O_4S^+$ [M+H]$^+$: 255.0691; Found: 255.0694.

(*E*)-Ethyl 2-(benzenesulfinyl)cyclopropane-1-carboxylate (6)

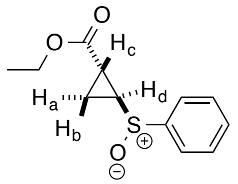

*m*CPBA (2.40 g, 13.9 mmol, 1.05 equiv) was added portionwise (3 × 0.80 g over 30 min) to a solution of (*E*)-ethyl 2-(phenylsulfanyl)cyclopropane-1-carboxylate **1** (2.95 g, 13.3 mmol, 1.0 equiv) in CH_2Cl_2 (133 mL) at 0 °C. The reaction was stirred at 0 °C for 2 h, then aqueous KOH (3 M, 50 mL) was added and the phases separated. The aqueous phase was extracted with CH_2Cl_2 (5 × 50 mL), then the organic phases were combined, washed with brine (50 mL), dried ($MgSO_4$), filtered and concentrated under reduced pressure. Purification by flash column chromatography (1:1 to 1:2 *n*-hexane:Et_2O) afforded a yellow oil which was dissolved in CH_2Cl_2, dried ($MgSO_4$), filtered and concentrated under reduced pressure to give sulfoxides **6** (2.91 g, 92%, d.r = 53:47) as a pale yellow oil. R_f = 0.24 (1:2 *n*-hexane:Et_2O). IR (film) cm^{-1} 3094 (CH), 3055 (CH), 2982 (CH), 2941 (CH), 2910 (CH), 1726 (C=O), 1444, 1381, 1258, 1182, 1048 (S-O).

Major diastereoisomer: ^{1}H NMR (400 MHz, $CDCl_3$) δ 7.67–7.61 (m, 2 H, 2 × Ph-H), 7.57–7.52 (m, 3 H, 3 × Ph-H), 4.16 (dddd, *J* = 7.1, 7.1, 7.1, 1.3 Hz, 2 H, C*H*$_2$CH$_3$), 2.75–2.68 (m, 1 H, H_c), 2.21 (ddd, *J* = 9.4, 5.6, 4.2 Hz, 1 H, H_d), 1.69 (ddd, *J* = 9.3, 6.0, 5.1 Hz, 1 H, H_a), 1.34 (ddd, *J* = 8.5, 5.3, 5.3 Hz, 1 H, H_b), 1.27 (t, *J* = 7.2 Hz, 3 H, CH_3). ^{13}C NMR (101 MHz, $CDCl_3$) δ 171.5 (C=O), 143.8 (Ph-C quat), 131.4 (Ph-C), 129.3 (2 × Ph-C), 123.9 (2 × Ph-C), 61.3 (*C*H$_2$CH$_3$), 40.6 (C(H_c)), 18.2 (C(H_d)), 14.1 (CH_3), 8.1 (C(H_a)(H_b)).

Minor diastereoisomer ^{1}H NMR (400 MHz, $CDCl_3$) δ 7.69–7.61 (m, 2 H, 2 × Ph-H), 7.57–7.52 (m, 3 H, 3 × Ph-H), 4.07 (ddd, *J* = 7.2, 7.2, 7.2 Hz, 2 H, C*H*$_2$CH$_3$), 2.75–2.68 (m, 1 H, H_c), 2.40 (ddd, *J* = 9.1, 5.7, 4.1 Hz, 1 H, H_d), 1.57 (ddd, *J* = 8.9, 5.4, 5.4 Hz, 1 H, H_b), 1.51 (ddd, *J* = 9.1, 6.2, 5.2 Hz, 1 H, H_a), 1.20 (t, *J* = 7.1 Hz,

3 H, CH_3). ^{13}C NMR (101 MHz, $CDCl_3$) δ 171.4 (C=O), 143.9 (Ph-C quat), 131.3 (Ph-C), 129.3 (2 × Ph-C), 123.9 (2 × Ph-C), 61.2 (CH_2CH_3), 41.4 ($C(H_c)$), 15.8 ($C(H_d)$), 14.1 (CH_3), 11.7 ($C(H_a)(H_b)$).
HRMS (ES) *m/z* Calcd for $C_{12}H_{15}O_3S^+$ $[M+H]^+$: 239.0742; Found: 239.0731.

(*Z*)-Ethyl-2-(benzenesulfinyl)cyclopropane-1-carboxylate (7)

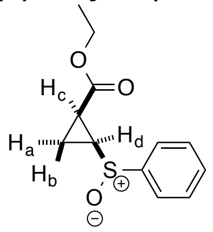

*m*CPBA (1.21 g, 7.03 mmol, 1.05 equiv) was added portion wise (3 × 0.40 g over 30 min) to a solution of (*Z*)-ethyl 2-thiophenylcyclopropane carboxylate **2** (1.49 g, 6.69 mmol, 1.0 equiv) in CH_2Cl_2 (67 mL) at 0 °C. The reaction was stirred at 0 °C for 3 h then warmed to 25 °C for 16 h 30 min. Aqueous KOH (3M, 50 mL) was added and the phases separated. The aqueous phase was extracted with CH_2Cl_2 (5 × 30 mL), then the organic phases were combined, washed with brine (50 mL), dried ($MgSO_4$), filtered and concentrated under reduced pressure to give sulfoxides **7** (1.56 g, 98%, d.r = 75:25) as a pale yellow oil. IR (film) cm^{-1} 3059 (CH), 2983 (CH), 2941 (CH), 1725 (C=O), 1444, 1382, 1188, 1040 (S-O).
Major diastereoisomer: R_f = 0.23 (Et_2O). ^1H NMR (400 MHz, $CDCl_3$) δ 7.68–7.64 (m, 2 H, 2 × Ph-H), 7.57–7.50 (m, 3 H, 3 × Ph-H), 4.23 (dddd, *J* = 7.1, 7.1, 7.1, 0.7 Hz, 2 H, CH_2CH_3), 2.61 (ddd, *J* = 8.4, 8.4, 6.7 Hz, 1 H, H_c), 2.13 (ddd, *J* = 8.2, 8.2, 6.5 Hz, 1 H, H_d), 2.07 (ddd, *J* = 6.5, 6.5, 5.6 Hz, 1 H, H_b), 1.63 (ddd, *J* = 8.2, 8.2, 5.6 Hz, 1 H, H_a), 1.30 (t, *J* = 7.1 Hz, 3 H, CH_3). ^{13}C NMR (101 MHz, $CDCl_3$) δ 170.0 (C=O), 145.1 (Ph-C quat.), 131.2 (Ph-C), 129.3 (2 × Ph-C), 124.0 (2 × Ph-C), 61.5 (CH_2CH_3), 43.3 ($C(H_c)$), 21.2 ($C(H_d)$), 14.2 (CH_3), 14.1 ($C(H_a)(H_b)$).
Minor diastereoisomer: R_f = 0.31 (Et_2O). ^1H NMR (400 MHz, $CDCl_3$) δ 7.76–7.73 (m, 2 H, 2 × Ph-H), 7.57–7.50 (m, 3 H, 3 × Ph-H), 4.31 (dddd, *J* = 7.1, 7.1, 7.1, 4.5 Hz, 2 H, CH_2CH_3), 2.68 (ddd, *J* = 8.7, 8.0, 6.7 Hz, 1 H, H_c), 2.31 (ddd, *J* = 8.0, 8.0, 6.4 Hz, 1 H, H_d), 1.75 (ddd, *J* = 6.5, 6.5, 5.7 Hz, 1 H, H_b), 1.38 (ddd, *J* = 8.8, 8.0, 5.7 Hz, 1 H, H_a), 1.35 (t, *J* = 7.2 Hz, 3 H, CH_3). ^{13}C NMR (101 MHz, $CDCl_3$) δ 170.1 (C=O), 145.2 (Ph-C quat), 131.0 (Ph-C), 129.3 (2 × Ph-C), 124.0 (2 × Ph-C), 61.7 (CH_2CH_3), 44.3 ($C(H_c)$), 20.9 ($C(H_d)$), 13.6 (CH_3), 11.8 ($C(H_a)(H_b)$).
HRMS (ES) *m/z* Calcd for $C_{12}H_{15}O_3S^+$ $[M+H]^+$: 239.0742; Found: 239.0754.

Synthesis of 8–13 through ester derivatization

(*E*)-2-(Phenylsulfanyl)cyclopropane-1-carboxylic acid (8)

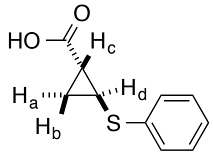

Aqueous NaOH (1.0 M, 0.55 mL, 0.55 mmol, 1.1 equiv) was added to a solution of (*E*)-ethyl 2-thiophenylcyclopropane carboxylate **1** (111 mg, 0.50 mmol, 1.0 equiv) in ethanol (2.5 mL) and the solution stirred at 30 °C for 24 h. $HCl_{(aq)}$ (1.0 M, 10 mL) was added and the mixture extracted with EtOAc (5 × 10 mL). The combined organic phases were dried ($MgSO_4$), filtered and concentrated under reduced pressure to give carboxylic acid **8** (97 mg, quant) as a white crystalline solid. R_f = 0.18 (1:1 pentane:Et_2O). mp = 106–110 °C. IR (film)/cm^{-1} 3008 (CH), 2863 (CH), 2530, 1679 (C=O), 1585, 1440, 1272, 1223, 928. ^1H NMR (400 MHz, $CDCl_3$) δ 7.37–7.29 (m, 4 H, 4 × Ph-H), 7.25–7.18 (m, 1 H, Ph-H), 2.85 (ddd, *J* = 8.4, 5.8, 3.5 Hz, 1 H, H_c), 1.95 (ddd, *J* = 8.7, 5.3, 3.6 Hz, 1 H, H_d), 1.73 (ddd, *J* = 8.3, 5.1, 5.1 Hz, 1 H, H_b), 1.35 (ddd, *J* = 8.6, 5.8, 5.0 Hz, 1 H, H_a). ^{13}C NMR (101 MHz, $CDCl_3$) δ 178.3 (C=O), 136.3 (Ph-C quat), 129.0 (2 × Ph-C), 127.6 (2 × Ph-C), 126.0 (Ph-C), 24.1 ($C(H_d)$), 23.5 ($C(H_c)$), 17.9 ($C(H_a)(H_b)$). HRMS (ES) *m/z* Calcd for $C_{10}H_9O_2S^+$ $[M-H]^+$: 193.0323; Found: 193.0332.

(*Z*)-2-(Phenylsulfanyl)cyclopropane-1-carboxylic acid (9)

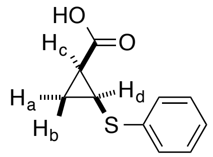

Aqueous NaOH (1.0 M, 0.55 mL, 0.55 mmol, 1.1 equiv) was added to a solution of (*E*)-ethyl 2-thiophenylcyclopropane carboxylate **2** (111 mg, 0.50 mmol, 1.0 equiv) in ethanol (2.5 mL) and the solution stirred at 30 °C for 24 h. $HCl_{(aq)}$ (1.0 M, 10 mL) was added and the mixture extracted with EtOAc (5 × 10 mL). The combined organic phases were dried ($MgSO_4$), filtered and concentrated under reduced pressure to give carboxylic acid **9** (97 mg, quant) as a cream crystalline solid. R_f = 0.22 (1:1 pentane:Et_2O). mp = 76–79 °C. IR (film)/cm^{-1} 3044 (CH), 2685 (CH), 2533, 1688 (C=O), 1478, 1436, 1202, 902. ^1H NMR (400 MHz, DMSO-d_6) δ 12.25 (br s, 1 H, CO_2H), 7.36–7.29 (m, 4 H, 4 × Ph-H), 7.18–7.13 (m, 1 H, Ph-H), 2.77 (ddd, *J* = 8.1, 8.1, 6.4 Hz, 1 H, H_c),

2.23 (ddd, *J* = 7.9, 7.9, 6.3 Hz, 1 H, H_d), 1.45 (ddd, *J* = 8.1, 8.1, 4.9 Hz, 1 H, H_a), 1.13 (ddd, *J* = 6.3, 6.3, 5.0 Hz, 1 H, H_b). ^{13}C NMR (101 MHz, DMSO-d_6) δ 170.6 (C=O), 137.3 (Ph-C quat), 129.0 (2 × Ph-C), 126.4 (2 × Ph-C), 125.2 (Ph-C), 21.3 (C(H_d)), 20.9 (C(H_c)), 12.6 (C(H_a)(H_b)). HRMS (ES) *m/z* Calcd for $C_{10}H_9O_2S^+$ $[M–H]^+$: 193.0323; Found: 193.0311.

(*E*)-[2-(Phenylsulfanyl)cyclopropyl]methanol (10)

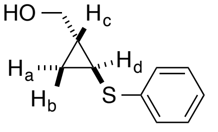

Lithium aluminum tetrahydride (1.0 M in THF, 1.0 mL, 1.0 mmol, 2.5 equiv) was added dropwise over 5 min to a 0 °C solution of (*E*)-ethyl 2-thiophenylcyclopropane carboxylate **1** (89 mg, 0.40 mmol, 1.0 equiv) in THF (0.5 mL) and the solution stirred at 0 °C for 10 min. The solution was warmed to 25 °C and stirred for 3 h. The reaction mixture was cooled to 0 °C, EtOAc (5 ml) was added and the mixture stirred for 15 min. The mixture was warmed to 25 °C, sat. aq. potassium sodium tartrate (5.0 mL) was added and the mixture stirred for 1 h. The organic phase was separated, and the aqueous phase extracted with Et_2O (6 × 10 mL). The combined organic phases were washed with brine (20 mL), dried ($MgSO_4$), filtered and concentrated under reduced pressure. Purification by flash column chromatography (2:1 pentane:Et_2O) gave alcohol **10** (63 mg, 87%) as a yellow oil. R_f = 0.13 (2:1 pentane:Et_2O). IR (film)/cm^{-1} 3340 (OH), 3059 (CH), 3004 (CH), 2923 (CH), 2873 (CH), 1584, 1480, 1271, 1025, 738, 690. ^{1}H NMR (400 MHz, $CDCl_3$) δ 7.41–7.39 (m, 2 H, 2 × Ph-H), 7.32–7.28 (m, 2 H, 2 × Ph-H), 7.18–7.14 (m, 1 H, Ph-H), 3.73 (dd, *J* = 11.4, 6.1 Hz, 1 H, OCH(H)), 3.56 (dd, *J* = 11.4, 7.1 Hz, 1 H, OCH(H)), 2.17 (ddd, *J* = 8.1, 4.3, 4.3 Hz, 1 H, H_c), 1.48–1.40 (m, 1 H, H_d), 1.05 (ddd, *J* = 7.8, 5.5, 5.5 Hz, 1 H, H_b), 0.95 (ddd, *J* = 8.9, 4.9, 4.9 Hz, 1 H, H_a). ^{13}C NMR (101 MHz, $CDCl_3$) δ 138.2 (Ph-C quat), 128.8 (2 × Ph-C), 126.8 (2 × Ph-C), 125.3 (Ph-C), 65.2 (OCH_2), 24.9 (C(H_c)), 17.4 (C(H_d)), 13.2 (C(H_a)(H_b)). HRMS (EI) *m/z* Calcd for $C_{10}H_{12}OS^+$ $[M]^+$: 180.0609; Found: 180.0599.

(*Z*)-[2-(Phenylsulfanyl)cyclopropyl]methanol (11)

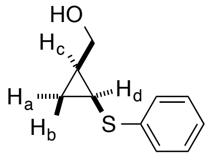

Lithium aluminum tetrahydride (1.0 M in THF, 1.0 mL, 1.0 mmol, 2.5 equiv) was added dropwise over 5 min to a solution of (*Z*)-ethyl 2-thiophenylcyclopropane carboxylate **2** (89 mg, 0.40 mmol, 1.0 equiv) in THF (0.5 mL) at 0 °C. The solution was stirred at 0 °C for 10 min then 25 °C for 3 h. The reaction mixture was cooled to 0 °C, EtOAc (5 ml) was added and the mixture stirred for 15 min. The mixture was warmed to 25 °C, sat. aq. potassium sodium tartrate (5.0 mL) was added and the mixture stirred for 1 h. The organic phase was separated, and the aqueous phase extracted with Et_2O (6 × 10 mL). The combined organic phases were washed with brine (20 mL), dried ($MgSO_4$), filtered and concentrated under reduced pressure to give cyclopropane **11** (63 mg, 87%) as a colourless oil. R_f = 0.40 (1:1 pentane:Et_2O). IR (film)/cm^{-1} 3245 (OH), 3069 (CH), 3008 (CH), 2928 (CH), 2869 (CH), 1582, 1478, 1438, 1056, 1022, 733. ^{1}H NMR (400 MHz, $CDCl_3$) δ 7.41–7.38 (m, 2 H, 2 × Ph-H), 7.33–7.29 (m, 2 H, 2 × Ph-H), 7.19–7.14 (m, 1 H, Ph-H), 3.87 (dd, *J* = 11.7, 4.4 Hz, 1 H, OCH(*H*)), 3.69 (dd, *J* = 11.8, 8.6 Hz, 1 H, OCH(*H*)), 2.48 (ddd, *J* = 8.0, 7.1, 5.1 Hz, 1 H, H_c), 1.71–1.62 (m, 1 H, H_d), 1.28 (ddd, *J* = 8.4, 8.4, 5.5 Hz, 1 H, H_a), 0.61 (ddd, *J* = 5.4, 5.4, 5.4 Hz, 1 H, H_b). ^{13}C NMR (101 MHz, $CDCl_3$) δ 138.2 (Ph-C quat), 129.0 (2 × Ph-C), 126.4 (2 × Ph-C), 125.4 (Ph-C), 62.5 (OCH_2), 21.2 (C(H_d)), 18.1 (C(H_c)), 10.9 (C(H_a)(H_b)). HRMS (EI) *m/z* Calcd for $C_{10}H_{10}S^+$ $[M–H_2O]^+$: 162.0503; Found: 162.0507.

(*E*)-Sodium 2-(phenylsulfanyl)cyclopropane-1-carboxylate (12)

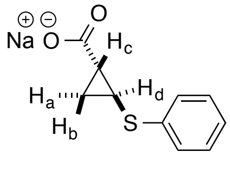

Aqueous NaOH (1.0 M, 2.9 mL, 2.9 mmol, 1.0 equiv) was added to a solution of (*E*)-ethyl 2-thiophenylcyclopropane carboxylate **1** (646 mg, 2.9 mmol, 1.0 equiv) in ethanol (14.5 mL) and the solution stirred at 30 °C for 24 h. The reaction mixture was concentrated under reduced pressure to give sodium carboxylate salt **12** (622 mg, quant) as a cream solid. mp = 207–212 °C. IR (film)/cm^{-1} 3521 (CH), 3232 (CH), 1567 (C=O), 1417, 1318, 1251, 955. ^{1}H NMR (400 MHz, DMSO-d_6) δ 7.34–7.27 (m, 4 H, 4 × Ph-H), 7.15–7.10 (m, 1 H, Ph-H), 2.39 (ddd, *J* = 7.9, 4.9, 3.7 Hz, 1 H, H_c), 1.43 (ddd, *J* = 8.8, 5.5, 3.6 Hz, 1 H, H_d), 1.30 (ddd, *J* = 7.7, 5.5, 3.8 Hz, 1 H, H_b), 0.76 (ddd, *J* = 8.6, 4.7, 3.9 Hz, 1 H, H_a). ^{13}C NMR (101 MHz, DMSO-d_6) δ 174.9 (C=O), 138.4 (Ph-C quat), 128.9 (2 × Ph-C), 125.8 (2 × Ph-C), 124.8 (Ph-C), 27.2 (C(H_d)), 18.3 (C(H_c)), 15.0 (C(H_a)(H_b)). HRMS (ES) *m/z* Calcd for $C_{10}H_9O_2S^+$ $[M–Na]^+$: 193.0323; Found: 193.0317.

(*Z*)-Sodium 2-(phenylsulfanyl)cyclopropane-1-carboxylate (13)

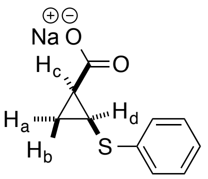

Aqueous NaOH (1.0 M, 4.5 mL, 4.5 mmol, 1.0 equiv) was added to a solution of (*Z*)-ethyl 2-thiophenylcyclopropane carboxylate **2** (1.00 g, 4.50 mmol, 1.0 equiv) in ethanol (22.5 mL) and the solution stirred at 30 °C for 24 h. The reaction mixture was concentrated under reduced pressure to give sodium carboxylate salt **13** (0.98 g, quant) as a cream solid. mp = 236–240 °C. IR (film)/cm^{-1} 3078 (CH), 3055 (CH), 3016 (CH), 1586 (C=O), 1424, 1315, 1282, 956. ^{1}H NMR (400 MHz, DMSO-d_6) δ 7.36–7.33 (m, 2 H, 2 × Ph-H), 7.27–7.22 (m, 2 H, 2 × Ph-H), 7.09–7.05 (m, 1 H, Ph-H), 2.24 (ddd, *J* = 8.0, 8.0, 5.9 Hz, 1 H, H_c), 1.77 (ddd, *J* = 8.2, 8.2, 6.5 Hz, 1 H, H_d), 1.10 (ddd, *J* = 8.0, 8.0, 3.9 Hz, 1 H, H_a), 0.90 (ddd, *J* = 6.2, 6.2, 4.0 Hz, 1 H, H_b). ^{13}C NMR (101 MHz, DMSO-d_6) δ 173.2 (C=O), 139.8 (Ph-C quat), 128.6 (2 × Ph-C), 126.3 (2 × Ph-C), 124.3 (Ph-C), 24.2 (C(H_d)), 19.4 (C(H_c)), 13.3 (C(H_a)(H_b)). HRMS (ES) *m/z* Calcd for $C_{10}H_9O_2S^+$ [M-Na]$^+$: 193.0323; Found: 193.0319.

Synthesis of 14a–15d through amide bond formation

(*E*)-*N*-Benzyl-2-(phenylsulfanyl)cyclopropane-1-carboxamide (14a)

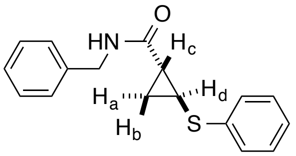

HATU (183 mg, 0.48 mmol, 1.2 equiv) was added to a solution of (*E*)-sodium 2-(phenylsulfanyl)cyclopropane-1-carboxylate **12** (87 mg, 0.40 mmol, 1.0 equiv) in *N*,*N*-dimethylformamide (2.0 mL) and stirred at 40 °C for 10 min. Benzylamine (53 μL, 0.48 mmol, 1.2 equiv) was added and the solution stirred for 20 min. Diisopropylethylamine (0.21 mL, 1.20 mmol, 3.0 equiv) was added and the solution stirred for 24 h. Water (100 mL) was added and the mixture extracted with CH_2Cl_2 (5 × 20 mL). The combined organic phases were washed with brine (30 mL), dried ($MgSO_4$), filtered and concentrated under reduced pressure. Purification by flash column chromatography (2:1 pentane:Et_2O) gave amide **14a** (112 mg, 99%) as a white solid. R_f = 0.17 (2:1 pentane:Et_2O). mp = 129–130 °C. IR (film)/cm^{-1} 3287 (NH), 3092 (CH), 2916 (CH), 1636 (C=O), 1559, 1391, 1222, 736, 695. ^{1}H NMR (400 MHz, $CDCl_3$) δ 7.39–7.24 (m, 9 H, 9 × Ph-H), 7.18–7.13 (m, 1 H, Ph-H), 6.04 (br s, 1 H, NH), 4.57 (dd, *J* = 14.7, 6.1 Hz, 1 H, NC(*H*)H), 4.41 (dd, *J* = 14.7, 5.4 Hz, 1 H, NC(*H*)H), 2.80 (ddd, *J* = 8.3, 5.5, 3.5 Hz, 1 H, H_c), 1.74 (ddd, *J* = 8.3, 5.3, 4.7 Hz, 1 H, H_a), 1.61 (ddd, 1 H, *J* = 8.5, 5.5, 3.6 Hz, H_d), 1.19 (ddd, *J* = 8.5, 5.4, 4.8 Hz, 1 H, H_b). ^{13}C NMR (101 MHz, $CDCl_3$) δ 170.6 (C=O), 138.1 (Ph-C quat), 137.4 (Ph-C quat), 128.9 (2 × Ph-C), 128.8 (2 × Ph-C), 127.8 (2 × Ph-C), 127.6 (Ph-C), 126.7 (2 × Ph-C), 125.4 (Ph-C), 43.9 ($PhCH_2$), 26.3 (C(H_d)), 21.1 (C(H_c)), 16.5 (C(H_a)(H_b)). HRMS (ES) *m/z* Calcd for $C_{17}H_{18}NOS^+$ [M+H]$^+$: 284.1109; Found: 284.1103.

(*E*)-(Morpholin-4-yl)-2-[(phenylsulfanyl)cyclopropyl]methanone (14b)

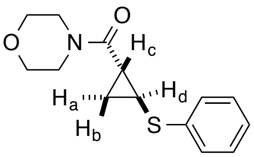

HATU (183 mg, 0.48 mmol, 1.2 equiv) was added to a solution of (*E*)-sodium 2-(phenylsulfanyl)cyclopropane-1-carboxylate **12** (87 mg, 0.40 mmol, 1.0 equiv) in *N*,*N*-dimethylformamide (2.0 mL) and stirred at 40 °C for 10 min. Morpholine (42 μL, 0.48 mmol, 1.2 equiv) was added and the solution stirred for 20 min. Diisopropylethylamine (0.21 mL, 1.20 mmol, 3.0 equiv) was added and the solution stirred for 24 h. H_2O (100 mL) was added and the mixture extracted with EtOAc (6 × 20 mL). The combined organic phases were washed with brine (30 mL), dried ($MgSO_4$), filtered and concentrated under reduced pressure. Purification by flash column chromatography (Et_2O) gave amide **14b** (101 mg, 96%) as a white solid. R_f = 0.36 (Et_2O). mp = 77–78 °C. IR (film)/cm^{-1} 2962 (CH), 2893 (CH), 2856 (CH), 1629 (C=O), 1441, 1233, 1117, 880. ^{1}H NMR (400 MHz, $CDCl_3$) δ 7.33–7.28 (m, 4 H, 4 × Ph-H), 7.21–7.16 (m, 1 H, Ph-H), 3.70–3.52 (m, 8 H, 2 × OCH_2 + 2 × NCH_2), 2.80 (ddd, *J* = 8.2, 5.4, 3.6 Hz, 1 H, H_c), 1.94 (ddd, *J* = 8.7, 5.4, 3.6 Hz, 1 H, H_d), 1.74 (ddd, *J* = 8.1, 5.4, 4.6 Hz, 1 H, H_b), 1.22 (ddd, *J* = 8.6, 5.3, 4.6 Hz, 1 H, H_a). ^{13}C NMR (101 MHz, $CDCl_3$) δ 169.4 (C=O), 137.2 (Ph-C quat), 128.9 (2 × Ph-H), 127.3 (2 × Ph-H), 125.7 (Ph-H), 66.8 (OCH_2), 66.7 (OCH_2), 46.0 (NCH_2), 42.6 (NCH_2), 22.6 (C(H_d)), 22.0 (C(H_c)), 16.9 (C(H_a)(H_b)). HRMS (ES) *m/z* Calcd for $C_{14}H_{18}NO_2S^+$ [M+H]$^+$: 264.1058; Found: 264.1056.

(*E*)-(4-Methylpiperazin-1-yl)[2-(phenylsulfanyl)cyclopropyl]methanone (14c)

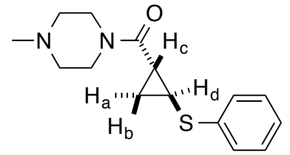

HATU (183 mg, 0.48 mmol, 1.2 equiv) was added to a solution of (*E*)-sodium 2-(phenylsulfanyl)cyclopropane-1-carboxylate **12** (87 mg, 0.40 mmol, 1.0 equiv) in *N*,*N*-dimethylformamide (2.0 mL) and stirred at 40 °C for 10 min. 1-Methylpiperazine (54 μL, 0.48 mmol, 1.2 equiv) was added and the solution stirred for 20 min. Diisopropylethylamine (0.21 mL, 1.20 mmol, 3.0 equiv) was added and the solution stirred for 24 h. Water (100 mL) was added and the mixture extracted with EtOAc (6 × 20 mL). The combined organic phases were washed with brine (30 mL), dried ($MgSO_4$), filtered and concentrated under reduced pressure. Purification by flash column chromatography (1:19 MeOH:CH_2Cl_2) gave amide **14c** (106 mg, 96%) as a brown gum. R_f = 0.17 (1:19 MeOH:CH_2Cl_2). IR (film)/cm^{-1} 3078 (CH), 3005 (CH), 2938 (CH), 2846 (CH), 2791, 1632 (C=O), 1439, 737. ^{1}H NMR (400 MHz, $CDCl_3$) δ 7.33–7.27 (m, 4 H, 4 × Ph-H), 7.21–7.15 (m, 1 H, Ph-H), 3.76–3.70 (m, 1 H, NCH(*H*)), 3.63–3.55 (m, 3 H, 3 × NCH(*H*)), 2.78 (ddd, *J* = 8.2, 5.4, 3.7 Hz, 1 H, H_c), 2.42–2.37 (m, 3 H, 3 × NCH(*H*)), 2.34–2.29 (m, 4 H, 1 × NCH(*H*) + NCH_3), 1.98 (ddd, *J* = 8.8, 5.4, 3.7 Hz, 1 H, H_d), 1.72 (ddd, *J* = 8.1, 5.4, 4.6 Hz, 1 H, H_b), 1.20 (ddd, *J* = 8.8, 5.4, 4.6 Hz, 1 H, H_a). ^{13}C NMR (101 MHz, $CDCl_3$) δ 169.2 (C=O), 137.3 (Ph-C quat), 128.9 (2 × Ph-C), 127.1 (2 × Ph-C), 125.6 (Ph-C), 55.2 (NCH_2), 54.7 (NCH_2), 46.0 (CH_3), 45.5 (NCH_2), 42.2 (NCH_2), 22.7 (C(H_d)), 21.8 (C(H_c)), 16.8 (C(H_a)(H_b)). HRMS (ES) *m/z* Calcd for $C_{15}H_{21}N_2OS^+$ [M+H]$^+$: 277.1375; Found: 277.1374.

(*E*)-(3,4-Dihydroquinolin-1(2*H*)-yl)[2-(phenylsulfanyl)cyclopropyl]methanone (14d)

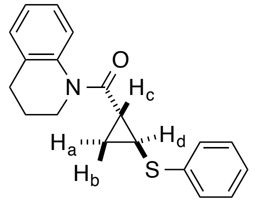

HATU (183 mg, 0.48 mmol, 1.2 equiv) was added to a solution of (*E*)-sodium 2-(phenylsulfanyl)cyclopropane-1-carboxylate **12** (87 mg, 0.40 mmol, 1.0 equiv) in *N*,*N*-dimethylformamide (2.0 mL) and stirred at 40 °C for 10 min. 1,2,3,4-Tetrahydroquinoline (60 μL, 0.48 mmol, 1.2 equiv) was added and the solution stirred for 20 min. Diisopropylethylamine (0.21 mL, 1.20 mmol, 3.0 equiv) was added and the solution stirred for 24 h. Water (100 mL) was added and the mixture extracted with EtOAc (6 × 20 mL). The combined organic phases were washed with brine (30 mL), dried ($MgSO_4$), filtered and concentrated under reduced pressure. Purification by flash column chromatography (2:1 pentane:Et_2O) gave amide **14d** (116 mg, 94%) as a yellow gum. R_f = 0.23 (2:1 pentane:Et_2O). IR (film)/cm^{-1} 3058 (CH), 2945 (CH), 2887 (CH), 1641 (C=O), 1491, 1397, 737. ^{1}H NMR (400 MHz, $CDCl_3$) δ 7.35–7.27 (m, 4 H, 4 × Ar-H), 7.20–7.09 (m, 5 H, 5 × Ph-H), 3.82 (t, *J* = 6.7 Hz, 2 H, CH_2), 2.96 (ddd, *J* = 8.1, 5.5, 3.7 Hz, 1 H, H_c), 2.73 (t, *J* = 6.6 Hz, 2 H, CH_2), 2.26 (ddd, *J* = 8.8, 5.3, 3.7 Hz, 1 H, H_d), 1.96 (m, 2 H, CH_2), 1.82 (ddd, *J* = 8.1, 5.4, 4.4 Hz, 1 H, H_b), 1.17 (ddd, *J* = 8.7, 4.9, 4.9 Hz, 1 H, H_a). ^{13}C NMR (101 MHz, $CDCl_3$) δ 170.6 (C=O), 138.6 (Ar-C quat), 137.1 (2 × Ar-C quat), 128.9 (2 × Ar-C), 128.5 (Ar-C), 127.6 (2 × Ar-C), 126.2 (Ar-C), 125.7 (Ar-C), 125.3 (Ar-C), 124.7 (Ar-C), 43.1 (NCH_2), 26.8 (CH_2), 24.5 (CH_2), 24.1 (C(H_d)), 23.3 (C(H_c)), 18.1 (C(H_a)(H_b)). HRMS (ES) *m/z* Calcd for $C_{19}H_{20}NOS^+$ [M+H]$^+$: 310.1266; Found: 310.1259.

(*Z*)-*N*-Benzyl-2-(phenylsulfanyl)cyclopropane-1-carboxamide (15a)

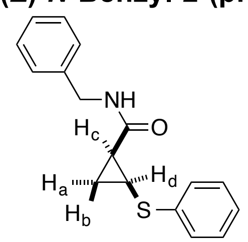

HATU (228 mg, 0.60 mmol, 1.2 equiv) was added to a solution of (*Z*)-sodium 2-(phenylsulfanyl)cyclopropane-1-carboxylate **13** (108 mg, 0.50 mmol, 1.0 equiv) in *N*,*N*-dimethylformamide (2.5 mL) and stirred at 40 °C for 10 min. Benzylamine (66 μL, 0.60 mmol, 1.2 equiv) was added and the solution stirred for 20 min. Diisopropylethylamine (0.27 mL, 1.50 mmol, 3.0 equiv) was added and the solution stirred for 24 h. Water (100 mL) was added and the mixture extracted with EtOAc (5 × 20 mL). The combined organic phases were washed with brine (30 mL), dried ($MgSO_4$), filtered and concentrated under reduced pressure. Purification by flash column chromatography (Et_2O) gave amide **15a** (123 mg, 87%) as a white solid. R_f = 0.32 (Et_2O). mp = 137−140 °C. IR (film)/cm^{-1} 3303 (NH), 3060 (CH), 1645 (C=O), 1552, 1244, 687. ^{1}H NMR (400 MHz, $CDCl_3$) δ 7.36–7.09 (m, 10 H, 10 × Ph-H), 6.11 (br s, 1 H, NH), 4.37 (d, *J* = 5.7 Hz, 2 H, $PhCH_2$), 2.64 (ddd, *J* = 8.2, 7.7, 6.4 Hz, 1 H, H_c), 2.10 (ddd, *J* = 8.2, 7.7, 6.6 Hz, 1 H, H_d), 1.49–1.41 (m, 2 H, H_a + H_b). ^{13}C NMR (101 MHz, $CDCl_3$) δ 168.5 (C=O), 138.0 (Ph-C quat), 137.1 (Ph-C quat), 128.9 (2 × Ph-C), 128.5 (2 × Ph-C), 127.7 (2 × Ph-C), 127.34 (2 × Ph-C), 127.28 (Ph-C), 125.6 (Ph-C), 43.9 ($PhCH_2$), 23.4 (C(H_d)), 21.1 (C(H_c)), 12.9 (C(H_a)(H_b)). HRMS (ES) *m/z* Calcd for $C_{17}H_{18}NOS^+$ [M+H]$^+$: 284.1109; Found: 284.1117.

(*Z*)-(Morpholin-4-yl)-2-[(phenylsulfanyl)cyclopropyl]methanone (15b)

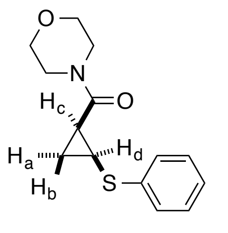

HATU (228 mg, 0.60 mmol, 1.2 equiv) was added to a solution of (*Z*)-sodium 2-(phenylsulfanyl)cyclopropane-1-carboxylate **13** (108 mg, 0.5 mmol, 1.0 equiv) in *N*,*N*-dimethylformamide (2.5 mL) and stirred at 40 °C for 10 min. Morpholine (52 μL, 0.60 mmol, 1.2 equiv) was added and the solution stirred for 20 min. Diisopropylethylamine (0.27 mL, 1.50 mmol, 3.0 equiv) was added and the solution stirred for 24 h. Water (100 mL) was added and the mixture extracted with EtOAc (5 × 20 mL). The combined organic phases were washed with brine (30 mL), dried ($MgSO_4$), filtered and concentrated under reduced pressure. Purification by flash column chromatography (EtOAc) gave amide **15b** (98 mg, 74%) as a yellow gum. R_f = 0.26 (EtOAc). IR (film)/cm^{-1} 3055 (CH), 2963 (CH), 2855 (CH), 1635 (C=O), 1437, 1228, 1112, 1035, 840, 739. ^{1}H NMR (400 MHz, $CDCl_3$) δ 7.39–7.36 (m, 2 H, 2 × Ph-H), 7.31–7.29 (m, 2 H, 2 × Ph-H), 7.21–7.17 (m, 1 H, Ph-H), 3.74–3.58 (m, 3 H, 3 × C(*H*)H), 3.55–3.45 (m, 3 H, 3 × C(*H*)H), 3.38–3.28 (m, 2 H, 2 × C(*H*)H), 2.76 (ddd, *J* = 8.0, 8.0, 5.9 Hz, 1 H, H_c), 2.18 (ddd, *J* = 8.0, 8.0, 6.2 Hz, 1 H, H_d), 1.61 (ddd, *J* = 5.9, 5.9, 5.9 Hz, 1 H, H_b), 1.43 (ddd, *J* = 8.1, 8.1, 5.3 Hz, 1 H, H_a). ^{13}C NMR (101 MHz, $CDCl_3$) δ 166.5 (C=O), 137.0 (Ph-C quat), 128.8 (2 × Ph-C), 128.4 (2 × Ph-C), 126.0 (Ph-C), 66.8 (OCH_2), 66.4 (OCH_2), 45.7 (NCH_2), 42.3 (NCH_2), 21.5 (C(H_d)), 20.9 (C(H_c)), 12.8 (C(H_a)(H_b)). HRMS (ES) *m/z* Calcd for $C_{14}H_{18}NO_2S^+$ $[M+H]^+$: 264.1058; Found: 264.1058.

(*Z*)-(4-Methylpiperazin-1-yl)[2-(phenylsulfanyl)cyclopropyl]methanone (15c)

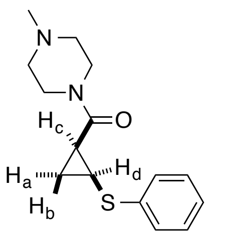

HATU (228 mg, 0.60 mmol, 1.2 equiv) was added to a solution of (*Z*)-sodium 2-(phenylsulfanyl)cyclopropane-1-carboxylate **13** (108 mg, 0.50 mmol, 1.0 equiv) in *N*,*N*-dimethylformamide (2.5 mL) and stirred at 40 °C for 10 min. 1-Methylpiperazine (67 μL, 0.60 mmol, 1.2 equiv) was added and the solution stirred for 20 min. Diisopropylethylamine (0.27 mL, 1.50 mmol, 3.0 equiv) was added and the solution stirred for 24 h. Water (100 mL) was added and the mixture extracted with EtOAc (5 × 20 mL). The combined organic phases were washed with brine (30 mL), dried ($MgSO_4$), filtered and concentrated under reduced pressure. Purification by flash column chromatography (1:19 MeOH:CH_2Cl_2) gave amide **15c** (125 mg, 90%) as a brown gum. R_f = 0.08 (1:19 MeOH:CH_2Cl_2). IR (film)/cm^{-1} 3004 (CH), 2937 (CH), 2793 (CH), 1635 (C=O), 1438, 1291, 1225, 1001, 739. ^{1}H NMR (400 MHz, $CDCl_3$) δ 7.38–7.36 (m, 2 H, 2 × Ph-H), 7.30–7.26 (m, 2 H, 2 × Ph-H), 7.19–7.15 (m, 1 H, Ph-H), 3.72–3.66 (m, 1 H, NC(*H*)H), 3.59–3.52 (m, 2 H, 2 × NC(*H*)H), 3.41–3.36 (m, 1 H, NCH(*H*)), 2.74 (ddd, *J* = 8.0, 8.0, 5.9 Hz, 1 H, H_c), 2.44–2.31 (m, 2 H, 2 × NCH(*H*)), 2.31–2.24 (m, 1 H, NCH(*H*)), 2.26 (s, 3 H, CH_3), 2.20 (ddd, *J* = 8.1, 8.1, 6.3 Hz, 1 H, H_d), 2.12 (ddd, *J* = 11.1, 7.6, 3.1 Hz, 1 H, NCH(*H*)), 1.59 (ddd, *J* = 5.8, 5.8, 5.8 Hz, 1 H, H_b), 1.41 (ddd, *J* = 8.2, 8.2, 5.3 Hz, 1 H, H_a). ^{13}C NMR (101 MHz, $CDCl_3$) δ 166.2 (C=O), 137.1 (Ph-C quat), 128.8 (2 × Ph-C), 128.4 (2 × Ph-C), 125.9 (Ph-C), 54.9 (NCH_2), 54.6 (NCH_2), 46.0 (CH_3), 45.3 (NCH_2), 41.9 (NCH_2), 21.6 (C(H_d)), 20.9 (C(H_c)), 12.9 (C(H_a)(H_b)). HRMS (ES) *m/z* Calcd for $C_{15}H_{21}N_2OS^+$ $[M+H]^+$: 277.1375; Found: 277.1371.

(*Z*)-(3,4-Dihydroquinolin-1(2*H*)-yl)[2-(phenylsulfanyl)cyclopropyl]methanone (15d)

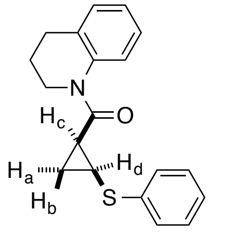

HATU (228 mg, 0.60 mmol, 1.2 equiv) was added to a solution of (*Z*)-sodium 2-(phenylsulfanyl)cyclopropane-1-carboxylate **13** (108 mg, 0.50 mmol, 1.0 equiv) in *N*,*N*-dimethylformamide (2.5 mL) and stirred at 40 °C for 10 min. 1,2,3,4-Tetrahydroquinoline (75 μL, 0.60 mmol, 1.2 equiv) was added and the solution stirred for 20 min. Diisopropylethylamine (0.27 mL, 1.50 mmol, 3.0 equiv) was added and the solution stirred for 24 h. Water (100 mL) was added and the mixture extracted with EtOAc (5 × 20 mL). The combined organic phases were washed with brine (30 mL), dried ($MgSO_4$), filtered and concentrated under reduced pressure. Purification by flash column chromatography (2:1 pentane:Et_2O) gave amide **15d** (122 mg, 79%) as a yellow gum. R_f = 0.15 (2:1 pentane:Et_2O). IR (film)/cm^{-1} 3004 (CH), 2937 (CH), 2793 (CH), 1635 (C=O), 1438, 1291, 1225, 1001, 739. ^{1}H NMR (400 MHz, $CDCl_3$) δ 7.42–7.40 (m, 2 H, 2 × Ph-H), 7.29–7.26 (m, 2 H, 2 × Ph-H), 7.19–7.07 (m, 5 H, 5 × Ar-H), 4.14–4.12 (m 1 H, 1 × CH(*H*)), 3.57–3.54 (m, 1 H, CH(*H*)), 2.68–2.64 (m, 3 H, 2 × CH(*H*) + H_c), 2.42 (ddd, *J* = 8.0, 8.0, 6.3 Hz, 1 H, H_d), 2.05–1.98 (m, 1 H, CH(*H*)), 1.85–1.75 (m, 1 H, CH(*H*)), 1.70 (ddd, *J* = 6.2, 6.2, 5.3 Hz, 1 H, H_b), 1.44 (ddd, *J* = 8.0, 8.0, 5.1 Hz, 1 H, H_a). ^{13}C NMR (101 MHz, $CDCl_3$) δ 167.8 (C=O), 138.9 (Ar-C quat), 137.1

(2 × Ar-C quat), 128.82 (2 × Ph-C), 128.78 (2 × Ph-C), 128.5 (Ar-C), 126.0 (Ar-C), 125.9 (Ar-C), 125.1 (2 × Ar-C), 124.5 (Ar-C), 43.4 (NCH_2), 26.9 (CH_2), 24.0 (CH_2), 23.8 (C(H_c)), 22.6 (C(H_d)), 15.0 (C(H_a)(H_b)). HRMS (ES) *m/z* Calcd for $C_{19}H_{20}NOS^+$ $[M+H]^+$: 310.1266; Found: 310.1267.

Synthesis of 16c–17d through sulfide oxidation

(*E*)-[2-(Benzenesulfonyl)cyclopropyl](4-methylpiperazin-1-yl)methanone (16c)

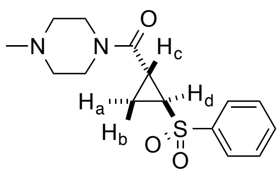

*m*CPBA (24 mg, 0.14 mmol, 3.0 equiv) was added to a solution of amide **14c** (12.6 mg, 0.046 mmol, 1.0 equiv) in CH_2Cl_2 (2.3 mL) and the solution stirred for 6 h at 25 °C. Solid $Na_2S_2O_5$ (26 mg, 3.0 equiv) was added and the mixture stirred for 30 min before being concentrated under reduced pressure. The mixture was redissolved in acetone containing 5% Et_3N, then filtered. The filtrate was concentrated under reduced pressure then purified by flash column chromatography (2:8 MeOH:CH_2Cl_2 + 2% Et_3N to 3:7 MeOH:CH_2Cl_2 + 2% Et_3N). The material was dissolved in CH_2Cl_2 and filtered to give sulfone **16c** (14 mg, quant) as a cream gum. R_f = 0.13 (3:7 MeOH:CH_2Cl_2). IR (film)/cm^{-1} 2958 (CH), 1640 (C=O), 1447, 1306 (S=O), 1150, 1088, 979, 745, 726, 692. ^1H NMR (400 MHz, $CDCl_3$, 58:42 rotameric mixture, asterix denotes a rotameric signal) δ 7.92–9.90 (m, 2 H, 2 × Ph-H), 7.71–7.67 (m, 1 H, Ph-H), 7.61–7.58 (m, 2 H, 2 × Ph-H), 4.44 (t, *J* = 15.1 Hz, 1 H, NC(*H*)H), 4.22–4.01 (m, 2 H, 2 × NC(*H*)H), 3.66–3.63 (m, 1 H, NC(*H*)H), 3.47–3.23 (m, 4 H, 4 × NC(*H*)H), 3.01 (ddd, *J* = 8.2, 5.6, 4.3 Hz, 1 H, H_c*), 2.70 (ddd, *J* = 9.6, 6.0, 4.6 Hz, 1 H, H_d*), 1.70–1.57 (m, 1 H, H_b*), 1.25 (s, 3 H, CH_3), 0.90–0.83 (m, 1 H, H_a*). ^{13}C NMR (asterix denotes minor rotamer, 101 MHz, $CDCl_3$) δ 166.9 (C=O), 139.5 (Ph-C quat), 134.0 (Ph-C), 129.5 (2 × Ph-C), 127.6 (2 × Ph-C), 65.3 (NCH_2), 65.1 (NCH_2) 60.2 (NCH_2), 40.4 (C(H_c)), 37.0 (NCH_2), 29.7 (CH_3), 18.1* (C(H_d)), 17.6 (C(H_d)), 13.4 (C(H_a)(H_b)), 13.1* (C(H_a)(H_b)). HRMS (ES) *m/z* Calcd for $C_{15}H_{21}N_2O_3S^+$ $[M+H]^+$: 309.1273; Found: 309.1278.

(*Z*)-[2-(Benzenesulfonyl)cyclopropyl](4-methylpiperazin-1-yl)methanone (17c)

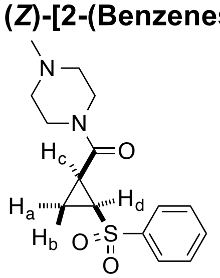

*m*CPBA (28 mg, 0.16 mmol, 3.0 equiv) was added to a 25 °C solution of amide **15c** (15 mg, 0.05 mmol, 1.0 equiv) in CH_2Cl_2 (2.7 mL) and the solution stirred for 6 h. Solid $Na_2S_2O_5$ (31 mg, 3.0 equiv) was added and the mixture stirred for 30 min before being concentrated under reduced pressure. The mixture was redissolved in acetone containing 5% Et_3N, then filtered. The filtrate was concentrated under reduced pressure then purified by flash column chromatography (3:7 MeOH:CH_2Cl_2 + 2% Et_3N). The material was dissolved in CH_2Cl_2, filtered and concentrated under reduced pressure to give sulfone **17c** (17 mg, quant) as a golden gum. R_f = 0.29 (3:7 MeOH:CH_2Cl_2). IR (film)/cm^{-1} 3032 (CH), 2947 (CH), 1643 (C=O), 1446, 1290 (S=O), 1147, 730, 690. ^1H NMR (400 MHz, $CDCl_3$) δ 7.90 (m, 2 H, 2 × Ph-H), 7.72–7.67 (m, 1 H, Ph-H), 7.62–7.58 (m, 2 H, 2 × Ph-H), 4.68–4.64 (m, 1 H, NC(*H*)H), 4.26–4.22 (m, 2 H, 2 × NC(*H*)H), 3.80–3.76 (m, 2 H, 2 × NC(*H*)H), 3.52–3.48 (m, 1 H, NC(*H*)H), 3.42 (ddd, *J* = 11.4, 2.2, 1.8 Hz, 1 H, NC(*H*)H), 3.37 (s, 3 H, CH_3), 3.35–3.23 (m, 1 H, NC(*H*)H), 2.83 (ddd, *J* = 8.6, 8.6, 6.6 Hz, 1 H, H_c), 2.34 (ddd, *J* = 8.6, 8.6, 7.5 Hz, 1 H, H_d), 1.97 (ddd, *J* = 7.0, 6.3, 6.3 Hz, 1 H, H_b), 1.39 (ddd, *J* = 8.6, 8.6, 5.9 Hz, 1 H, H_a). ^{13}C NMR (101 MHz, $CDCl_3$) δ 164.1 (C=O), 139.8 (Ph-C quat), 134.0 (Ph-C), 129.5 (2 × Ph-C), 127.6 (2 × Ph-C), 64.5 (NCH_2), 64.3 (NCH_2), 59.6 (CH_3), 40.4 (NCH_2), 40.2 (C(H_c)), 36.6 (NCH_2), 23.8 (C(H_d)), 11.4 (C(H_a)(H_b)). HRMS (ES) *m/z* Calcd for $C_{15}H_{21}N_2O_3S^+$ $[M+H]^+$: 309.1273; Found: 309.1270.

(*E*)-[2-(Benzenesulfonyl)cyclopropyl](3,4-dihydroquinolin-1(2*H*)-yl)methanone (16d)

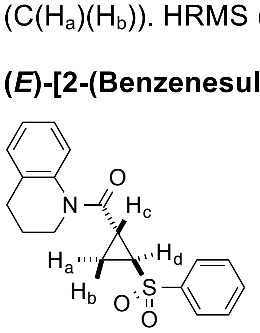

*m*CPBA (104 mg, 0.60 mmol, 3.0 equiv) was added to a 25 °C solution of amide **14d** (62 mg, 0.20 mmol, 1.0 equiv) in CH_2Cl_2 (10 mL) and the solution stirred for 6 h. Water (30 mL) was added and the phases separated. The aqueous phase was extracted with Et_2O (5 × 30 mL). The combined organic phases were washed with brine, dried ($MgSO_4$), filtered and concentrated under reduced pressure. Purification by flash column chromatography (1:2 pentane:Et_2O) gave sulfone **16d** (67 mg, 98%) as a cream oil. R_f = 0.29 (1:2 pentane:Et_2O). IR (film)/cm^{-1} 3040 (CH), 2950 (CH), 1642 (C=O), 1581, 1492, 1402, 1306, 1148, 911, 727. ^1H NMR (400 MHz, $CDCl_3$) δ 7.85 (d, *J* = 7.6 Hz, 2 H, 2 × Ph-H), 7.67 (t, *J* =

7.4 Hz, 1 H, Ph-H), 7.57 (t, *J* = 7.7 Hz, 2 H, 2 × Ph-H), 7.25–7.20 (m, 4 H, 4 × Ar-H), 3.80–3.71 (m, 2 H, 2 × NC(*H*)H), 3.14 (ddd, *J* = 8.8, 5.5, 5.5 Hz, 1 H, H_c), 2.93 (ddd, *J* = 9.6, 5.7, 4.4 Hz, 1 H, H_d), 2.76–2.63 (m, 2 H, 2 × ArC(*H*)H), 2.01–1.86 (m, 2 H, 2 × NCH_2C(*H*)H), 1.73 (ddd, *J* = 8.4, 5.8, 4.9 Hz, 1 H, H_b), 1.65–1.63 (m, 1 H, H_a). ^{13}C NMR (101 MHz, $CDCl_3$) δ 168.2 (C=O), 140.1 (Ph-C quat + Ar-C quat), 137.9 (Ar-C quat), 133.7 (Ph-C), 129.4 (2 × Ph-C), 128.6 (Ar-C), 127.6 (2 × Ph-C), 126.7 (Ar-C), 126.0 (Ar-C), 124.8 (Ar-C), 43.3 (CH_2), 41.3 (C(H_c)), 26.7 (CH_2), 24.0 (CH_2), 20.1 (C(H_d)), 14.2 (C(H_a)(H_b)). HRMS (ES) *m/z* Calcd for $C_{19}H_{20}NO_3S^+$ $[M+H]^+$: 342.1164; Found: 342.1174.

(*E*)-[2-(Benzenesulfonyl)cyclopropyl](3,4-dihydroquinolin-1(2*H*)-yl)methanone (17d)

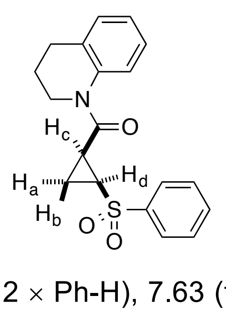

*m*CPBA (78 mg, 0.45 mmol, 3.0 equiv) was added to a 25 °C solution of amide **15d** (47 mg, 0.15 mmol, 1.0 equiv) in CH_2Cl_2 (7.5 mL) and the solution stirred for 6 h. Water (30 mL) was added and the phases separated. The aqueous phase was extracted with Et_2O (5 × 30 mL). The combined organic phases were washed with brine, dried ($MgSO_4$), filtered and concentrated under reduced pressure. Purification by flash column chromatography (Et_2O) gave the sulfone **17d** (44 mg, 86%) as a cream colored gum. R_f = 0.19 (Et_2O). IR (film)/cm^{-1} 3030 (CH), 2934 (CH), 1655 (C=O), 1581, 1491, 1403, 1321 (S=O), 1291, 1150 (S=O), 1086, 727. ^1H NMR (400 MHz, $CDCl_3$) δ 8.00–7.98 (m, 2 H, 2 × Ph-H), 7.63 (t, *J* = 7.5 Hz, 1 H, Ph-H), 7.57 (t, *J* = 7.5 Hz, 2 H, 2 × Ph-H), 7.16–7.14 (m, 4 H, 4 × Ar-H), 4.34 (br s, 1 H, NC(*H*)H), 3.40 (br s, 1 H, NC(*H*)H), 2.80–2.70 (m, 3 H, H_c + 2 × ArC(*H*)H), 2.41–2.39 (m, 1 H, H_d), 2.18–2.09 (m, 1 H, NCH_2C(*H*)H), 2.02 (br s, 1 H, H_b), 1.85 (br s, 1 H, NCH_2C(*H*)H), 1.39–1.34 (m, 1 H, H_a). ^{13}C NMR (101 MHz, $CDCl_3$) δ 164.8 (C=O), 140.4 (Ph-C quat + Ar-C quat), 133.9 (Ar-C quat), 133.6 (Ph-C), 129.0 (2 × Ph-C), 128.4 (Ar-C), 128.1 (2 × Ph-C), 126.0 (Ar-C), 125.5 (Ar-C), 124.2 (Ar-C), 43.6 (CH_2), 42.6 (CH_2), 26.8 (C(H_c)), 25.6 (C(H_d)), 23.8 (CH_2), 13.3 (C(H_a)(H_b)). HRMS (ES) *m/z* Calcd for $C_{19}H_{20}NO_3S^+$ $[M+H]^+$: 342.1164; Found: 342.1163.

Synthesis of 19a–20l through sulfoxide–magnesium exchange, electrophilic trapping

(*E*)-Ethyl-2-iodocyclopropanecarboxylate (19a)

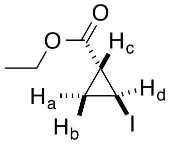

i-PrMgCl (0.32 mL, 1.87 M in THF, 0.60 mmol, 1.5 equiv) was added to a −78 °C solution of (*E*)-ethyl 2-(phenylsulfinyl)cyclopropanecarboxylate **6** (95 mg, 0.40 mmol, 1.0 equiv) in THF (4.0 mL) over 10 s and the solution stirred for 10 min. A solution of I_2 (203 mg, 0.80 mmol, 2.0 equiv) in THF (0.5 mL) was added and the reaction stirred at −78 °C for 1 h. The reaction mixture was warmed to rt, sat. aq NH_4Cl (5 mL) added and the mixture was stirred for 5 min. Water (10 mL) was added and the mixture extracted with CH_2Cl_2 (3 × 30 mL). The combined organic phases were washed with brine, dried ($MgSO_4$), filtered and concentrated under reduced pressure. Purification by flash column chromatography (100% *n*-hexane to 10:1 *n*-hexane:Et_2O) gave cyclopropane **19a** (79 mg, 82%) as a yellow oil. IR (film)/cm^{-1} 2981 (CH), 1721 (C=O), 1396, 1376, 1174, 1033. ^1H NMR (400 MHz, $CDCl_3$) δ 4.15 (q, *J* = 7.1 Hz, 2 H, C$H_2$$CH_3$), 2.77 (ddd, *J* = 8.2, 5.6, 3.7 Hz, 1 H, H_d), 1.97 (ddd, *J* = 9.0, 5.5, 3.7 Hz, 1 H, H_c), 1.63 (ddd, *J* = 8.2, 5.6, 5.6 Hz, 1 H, H_a), 1.32–1.24 (m, 1 H, H_b), 1.27 (t, *J* = 7.1 Hz, 3 H, CH_3). ^{13}C NMR (101 MHz, $CDCl_3$) δ 171.9 (C=O), 61.1 (CH_2CH_3), 24.6 (C(H_c)), 19.6 (C(H_a)(H_b)), 14.2 (CH_3), −17.1 (C(H_d)). HRMS (EI) *m/z* Calcd for $C_6H_9O_2I^+$ $[M]^+$: 239.9647; Found: 239.9651.

(*E*)-Ethyl 2-[cyclopentyl(hydroxyl)methyl]cyclopropane-1-carboxylate (19b)

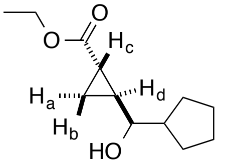

i-PrMgCl (0.32 mL, 1.87 M in THF, 0.60 mmol, 1.5 equiv) was added to a −78 °C solution of (*E*)-ethyl 2-(phenylsulfinyl)cyclopropanecarboxylate **6** (95 mg, 0.40 mmol, 1.0 equiv) in THF (4.0 mL) over 10 s and the solution stirred for 10 min. Cyclopentanecarboxaldehyde (85 µL, 0.80 mmol, 2.0 equiv) was added and the reaction stirred at 0 °C for 1 h. The reaction was warmed to rt, sat. aq NH_4Cl (5 mL) added and the mixture was stirred for 5 min. Water (10 mL) was added and the mixture extracted with CH_2Cl_2 (5 × 30 mL). The combined organic phases were washed with brine, dried ($MgSO_4$), filtered and concentrated under reduced pressure. Purification by flash column chromatography (2:1 pentane:Et_2O) gave cyclopropane **19b** as a

mixture of two diastereoisomers (d.r = 56:44, 60 mg, 71%) as a colourless oil. R_f = 0.16 (2:1 pentane:Et_2O). IR (film)/cm^{-1} 3450 (OH), 2952 (CH), 2911 (CH), 2869 (CH), 1724 (C=O), 1703 (C=O), 1176.

Major diastereoisomer: ^{1}H NMR (400 MHz, $CDCl_3$) δ 4.14–4.08 (m, 2 H, CH_2CH_3), 2.94 (dd, *J* = 7.3, 7.3 Hz, 1 H, C*H*(OH)), 2.06–1.93 (m, 1 H, C*H*$(CH_2)_2$), 1.84–1.73 (m, 2 H, $CHCH_2CH_2$), 1.64–1.54 (m, 4 H, $CH(CH_2)_2$), 1.67–1.55 (m, 1 H, H_d), 1.60–1.54 (m, 1 H, H_c), 1.41–1.30 (m, 4 H, $CH(CH_2CH_2)_2$), 1.24 (t, *J* = 7.2 Hz, 3 H, CH_3), 1.25–1.17 (m, 1 H, H_b), 0.92–0.87 (m, 1 H, H_a). ^{13}C NMR (101 MHz, $CDCl_3$) δ 174.2 (C=O), 77.2 (CH(OH)), 60.5 (CH_2CH_3), 46.7 ($CH(CH_2)_2$), 28.9 (CH_2), 28.6 (CH_2), 28.0 (C(H_c)), 25.5 (CH_2), 25.2 (CH_2), 17.4 (C(H_d)), 14.2 (CH_3), 13.2 (C(H_a)(H_b)).

Minor diastereoisomer: ^{1}H NMR (400 MHz, $CDCl_3$) δ 4.14–4.08 (m, 2 H, CH_2CH_3), 3.07 (dd, *J* = 7.1, 7.1 Hz, 1 H, C*H*(OH)), 2.06–1.93 (m, 1 H, C*H*$(CH_2)_2$), 1.84–1.73 (m, 2 H, $CHCH_2CH_2$), 1.64–1.54 (m, 4 H, $CH(CH_2)_2$), 1.67–1.55 (m, 1 H, H_d), 1.60–1.54 (m, 1 H, H_c), 1.41–1.30 (m, 2 H, $CHCH_2CH_2$), 1.24 (t, *J* = 7.2 Hz, 3 H, CH_3), 1.14–1.08 (m, 1 H, H_b), 0.96–0.92 (m, 1 H, H_a). ^{13}C NMR (101 MHz, $CDCl_3$) δ 174.0 (C=O), 76.4 (CH(OH)), 60.5 (CH_2CH_3), 46.9 ($CH(CH_2)_2$), 28.9 (CH_2), 28.8 (CH_2), 27.2 (C(H_c)), 25.5 (CH_2), 25.2 (CH_2), 18.3 (C(H_d)), 14.2 (CH_3), 11.4 (C(H_a)(H_b)).

HRMS (ES) *m/z* Calcd for $C_{12}H_{19}O_2^+$ $[M–OH]^+$: 195.1385; Found: 195.1386.

(*E*)-Ethyl 2-(hydroxy(phenyl)methyl)cyclopropane-1-carboxylate (19c)

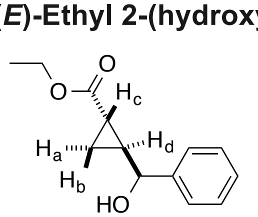

i-PrMgCl (0.32 mL, 1.87 M in THF, 0.60 mmol, 1.5 equiv) was added to a −78 °C solution of (*E*)-ethyl 2-(phenylsulfinyl)cyclopropanecarboxylate **6** (95 mg, 0.40 mmol, 1.0 equiv) in THF (4.0 mL) over 10 s and the solution stirred for 10 min. Benzaldehyde (81 μL, 0.80 mmol, 2.0 equiv) was added and the reaction stirred at 0 °C for 1 h. The reaction was warmed to rt, sat. aq NH_4Cl (5 mL) added and the mixture was stirred for 5 min. H_2O (10 mL) was added and the mixture extracted with CH_2Cl_2 (3 × 30 mL). The combined organic phases were washed with brine, dried ($MgSO_4$), filtered and concentrated under reduced pressure. Purification by flash column chromatography (10:1 *n*-hexane:Et_2O) gave cyclopropane **19c** as a mixture of two diastereomers (d.r = 61:39, 83 mg, 94%) as a colourless oil. R_f = 0.16 (2:1 *n*-hexane:Et_2O). IR (film)/cm^{-1} 3458 (OH), 2982 (CH), 1722 (C=O), 1703 (C=O), 1180.

Major diastereoisomer: ^{1}H NMR (400 MHz, $CDCl_3$) δ 7.40–7.28 (m, 5 H, 5 × Ph-H), 4.21 (d, *J* = 7.3 Hz, 1 H, C*H*(OH)), 4.16–4.02 (m, 2 H, CH_2CH_3), 2.56 (br s, 1 H, OH), 1.86–1.78 (m, 1 H, H_d), 1.81 (ddd, *J* = 8.4, 4.3, 4.3 Hz, 1 H, H_c), 1.25 (t, *J* = 7.1 Hz, 3 H, CH_3), 1.24–1.18 (m, 1 H, H_a), 0.97 (ddd, *J* = 8.5, 6.4, 4.5 Hz, 1H, H_b). ^{13}C NMR (101 MHz, $CDCl_3$) δ 173.7 (C=O), 142.8 (Ph-C quat), 128.5 (2 × Ph-C), 127.83 (Ph-C), 126.00 (2 × Ph-C), 75.6 (CH(OH)), 60.6 (CH_2CH_3), 29.2 (C(H_d)), 18.8 (C(H_c)), 14.1 (CH_3), 12.5 (C(H_a)(H_b)).

Minor diastereoisomer: ^{1}H NMR (400 MHz, $CDCl_3$) δ 7.40–7.28 (m, 5 H, 5 × Ph-H), 4.47 (d, *J* = 6.0 Hz, 1 H, C*H*(OH)), 4.16–4.02 (m, 2 H, CH_2CH_3), 2.45 (br s, 1 H, OH), 1.89–1.78 (m, 1 H, H_d), 1.71 (ddd, *J* = 8.9, 4.6, 4.6 Hz, 1 H, H_c), 1.24–1.18 (m, 1 H, H_a), 1.22 (t, *J* = 7.1 Hz, 3 H, CH_3), 1.10 (ddd, *J* = 8.6, 6.4, 4.2 Hz, 1 H, H_b). ^{13}C NMR (101 MHz, $CDCl_3$) δ 173.8 (C=O), 142.8 (Ph-C quat), 128.4 (2 × Ph-C), 127.78 (Ph-C), 126.03 (2 × Ph-C), 73.9 (CH(OH)), 60.5 (CH_2CH_3), 28.4 (C(H_d)), 17.6 (C(H_c)), 14.1 (CH_3), 12.2 (C(H_a)(H_b)).

HRMS (ES) *m/z* Calcd for $C_{15}H_{20}NO_3^+$ $[M+H+CH_3CN\ adduct]^+$: 262.1443; Found: 262.1447. The observed data (IR, ^{1}H, ^{13}C) was consistent with that previously reported.[10]

(*E*)-Ethyl 2-[hydroxy(pyridine-3-yl)methyl]cyclopropane-1-carboxylate (19d)

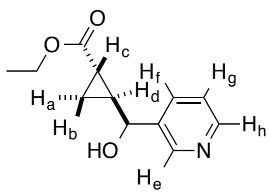

i-PrMgCl (0.32 mL, 1.87 M in THF, 0.60 mmol, 1.5 equiv) was added to a −78 °C solution of (*E*)-ethyl 2-(phenylsulfinyl)cyclopropanecarboxylate **6** (95 mg, 0.40 mmol, 1.0 equiv) in THF (4.0 mL) over 10 s and the solution stirred for 10 min. 3-Pyridinecarboxaldehyde (75 μL, 0.80 mmol, 2.0 equiv) was added and the reaction stirred at 0 °C for 1 h. The reaction was warmed to rt, sat. aq NH_4Cl (5 mL) added and the mixture stirred for 5 min. H_2O (10 mL) was added and the mixture extracted with CH_2Cl_2 (6 × 20 mL) then EtOAc (6 × 20 mL). The combined organic phases were concentrated under reduced pressure, dissolved in CH_2Cl_2, washed with brine, dried ($MgSO_4$), filtered and concentrated under reduced pressure. Purification by flash column chromatography (4:1 EtOAc:toluene) gave cyclopropane **19d** as a mixture of two diastereomers (d.r = 52:48, 80 mg, 90%) as a white oil. R_f = 0.26 (4:1 EtOAc:toluene). IR (film)/cm^{-1} 3169 (OH), 2982 (CH), 1719 (C=O), 1177, 1028, 713.

<u>*Major diastereoisomer:*</u> ^{1}H NMR (400 MHz, $CDCl_3$) δ 8.47 (br s, 1 H, H_e), 8.40–8.38 (m, 1 H, H_h), 7.79–7.73 (m, 1 H, H_f), 7.28–7.24 (m, 1 H, H_g), 4.73 (br s, 1 H, OH), 4.26 (d, *J* = 7.2 Hz, 1 H, C*H*(OH)), 4.13–4.00 (m, 2 H, C$H_2$$CH_3$), 1.85–1.76 (m, 1 H, H_c), 1.83–1.74 (m, 1 H, H_d), 1.23–1.18 (m, 1 H, H_a), 1.21 (t, *J* = 7.1 Hz, 3 H, CH_3), 0.99 (ddd, *J* = 8.4, 6.4, 4.5 Hz, 1 H, H_b). ^{13}C NMR (101 MHz, $CDCl_3$) δ 173.5 (C=O), 148.5 (Ar-C), 147.3 (Ar-C), 139.1 (Ar-C quat), 134.0 (Ar-C), 123.6 (Ar-C), 72.7 (CH(OH)), 60.6 (*C*$H_2$$CH_3$), 29.0 (C($H_d$)), 18.7 (C($H_c$)), 14.1 ($CH_3$), 12.5 (C($H_a$)($H_b$)).
<u>*Minor diastereoisomer:*</u> ^{1}H NMR (400 MHz, $CDCl_3$) δ 8.47 (br s, 1 H, H_e), 8.40–8.38 (m, 1 H, H_h), 7.79–7.73 (m, 1 H, H_f), 7.28–7.24 (m, 1 H, H_g), 4.73 (br s, 1 H, OH), 4.50 (d, *J* = 5.7 Hz, 1 H, C*H*(OH)), 4.13–4.00 (m, 2 H, C$H_2$$CH_3$), 1.81–1.71 (m, 1 H, H_d), 1.78–1.71 (m, 1 H, H_c), 1.23–1.18 (m, 1 H, H_a), 1.20 (t, *J* = 7.1 Hz, 3 H, CH_3), 1.14 (ddd, *J* = 8.5, 6.5, 4.3 Hz, 1 H, H_b). ^{13}C NMR (101 MHz, $CDCl_3$) δ 173.6 (C=O), 148.5 (Ar-C), 147.4 (Ar-C), 139.2 (Ar-C quat), 134.1 (Ar-C), 123.6 (Ar-C), 71.1 (CH(OH)), 60.6 (*C*$H_2$$CH_3$), 28.3 (C($H_d$)), 17.7 (C($H_c$)), 14.1 ($CH_3$), 12.1 (C($H_a$)($H_b$)).
HRMS (ES) *m/z* Calcd for $C_{12}H_{16}NO_3^+$ $[M+H]^+$: 222.1130; Found: 222.1131.

(*E*)-Ethyl 2-(3-hydroxyoxetan-3-yl)cyclopropane-1-carboxylate (19e)

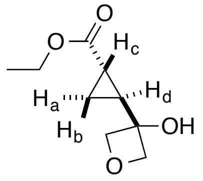

i-PrMgCl (0.32 mL, 1.87 M in THF, 0.60 mmol, 1.5 equiv) was added to a −78 °C solution of (*E*)-ethyl 2-(phenylsulfinyl)cyclopropanecarboxylate **6** (95 mg, 0.40 mmol, 1.0 equiv) in THF (4.0 mL) over 10 s and the solution stirred for 10 min. 3-Oxetanone (52 μL, 0.8 mmol, 2.0 equiv) was added and the reaction stirred at 0 °C for 1 h. The reaction was warmed to rt, sat. aq NH_4Cl (5 mL) added and the mixture stirred for 5 min. Water (10 mL) was added and the mixture extracted with CH_2Cl_2 (3 × 10 mL then 7 × 20 mL) followed by EtOAc (6 × 20 mL). The combined organic phases were concentrated under reduced pressure, dissolved in CH_2Cl_2, washed with brine, dried ($MgSO_4$), filtered and concentrated under reduced pressure. Purification by flash column chromatography (1:39 MeOH:CH_2Cl_2) gave cyclopropane **19e** (49 mg, 66%) as a pale yellow oil. R_f = 0.10 (1:39 MeOH:CH_2Cl_2). IR (film)/cm^{-1} 3400 (OH), 2977 (CH), 2956 (CH), 2876 (CH), 1723 (C=O), 1702 (C=O), 1316, 1178. ^{1}H NMR (400 MHz, $CDCl_3$) δ 4.58 (d, *J* = 6.8 Hz, 1 H, oxetane C(*H*)H), 4.54 (d, *J* = 6.9 Hz, 1 H, oxetane C(*H*)H), 4.50 (d, *J* = 6.8 Hz, 1 H, oxetane C(*H*)H), 4.44 (d, *J* = 6.9 Hz, 1 H, oxetane C(*H*)H), 4.19–4.07 (m, 2 H, C$H_2$$CH_3$), 3.29 (br s, 1 H, OH), 1.94 (ddd, *J* = 9.2, 6.5, 4.4 Hz, 1 H, H_d), 1.72 (ddd, *J* = 8.6, 4.9, 4.4 Hz, 1 H, H_c), 1.26 (t, *J* = 7.1 Hz, 3 H, CH_3), 1.25–1.18 (m, 1 H, H_a), 1.10 (ddd, *J* = 8.6, 6.5, 4.6 Hz, 1 H, H_b). ^{13}C NMR (101 MHz, $CDCl_3$) δ 173.8 (C=O), 83.3 (oxetane-CH_2), 82.5 (oxetane-CH_2), 72.8 (oxetane-C quat), 60.8 (*C*$H_2$$CH_3$), 27.2 (C($H_d$)), 16.4 (C($H_c$)), 14.1 ($CH_3$), 10.8 (C($H_a$)($H_b$)). HRMS (CI+) *m/z* Calcd for $C_9H_{18}NO_4^+$ $[M+NH_4]^+$: 204.1236; Found: 204.1226.

(*E*)-Ethyl-2-[bis(4-chlorophenyl)(hydroxyl)methyl]cyclopropane-1-carboxylate (19f)

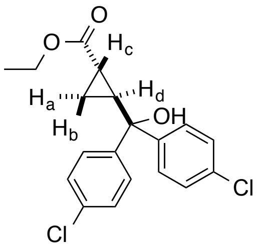

i-PrMgCl (0.32 mL, 1.87 M in THF, 0.60 mmol, 1.5 equiv) was added to a −78 °C solution of (*E*)-ethyl 2-(phenylsulfinyl)cyclopropanecarboxylate **6** (95 mg, 0.40 mmol, 1.0 equiv) in THF (4.0 mL) over 10 s and the solution stirred for 10 min. 4,4-Dichlorobenzophenone (201 mg, 0.80 mmol, 2.0 equiv) in toluene (1.0 mL) was added and the reaction stirred at 0 °C for 3 h. The reaction was warmed to rt, sat. aq. NH_4Cl (5 mL) added and the mixture stirred for 5 min. Water (20 mL) was added and the mixture extracted with EtOAc (5 × 20 mL). The combined organic phases were washed with brine, dried ($MgSO_4$), filtered and concentrated under reduced pressure. Purification by flash column chromatography (3:1 pentane:toluene) gave cyclopropane **19f** (117 mg, 80%) as a pale yellow oil. R_f = 0.59 (5:1 pentane:Et_2O). IR (film)/cm^{-1} 2969 (CH), 2923 (CH), 2877 (CH), 1652 (C=O), 1587, 1490, 1399, 1089, 1013, 906, 728. ^{1}H NMR (400 MHz, $CDCl_3$) δ 7.39–7.28 (m, 8 H, 8 × Ar-H), 4.13 (q, *J* = 7.2 Hz, 2 H, C$H_2$$CH_3$), 2.18 (ddd, *J* = 9.0, 6.7, 4.5 Hz, 1 H, H_c), 1.95 (br s, 1 H, OH), 1.79 (ddd, *J* = 8.7, 4.8, 4.8 Hz, 1 H, H_d), 1.30–1.23 (m, 4 H, H_b + CH_3), 1.13 (ddd, *J* = 8.6, 6.7, 4.3 Hz, 1 H, H_a). ^{13}C NMR (101 MHz, $CDCl_3$) δ 173.8 (C=O), 144.7 (Ar-C-Cl), 144.4 (Ar-C-Cl), 133.5 (2 × Ar-C quat), 128.5 (2 × Ar-C), 128.4 (2 × Ar-C), 128.0 (2 × Ar-C), 127.9 (2 × Ar-C), 75.6 (C(OH)), 60.8 (*C*$H_2$$CH_3$), 31.5 (C($H_c$)), 17.5 (C($H_d$)), 14.2 ($CH_3$), 11.9 (C($H_a$)($H_b$)). FTMS (+ p NSI) *m/z* Calcd for $C_{19}H_{18}O_3Cl_2Na^+$ $[M+Na]^+$: 387.0525; Found: 387.0526.

(*E*)-Ethyl 2-[hydroxydi(pyridin-2-yl)methyl]cyclopropane-1-carboxylate (19g)

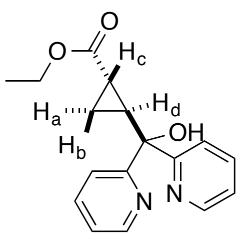

i-PrMgCl (0.32 mL, 1.85 M in THF, 0.60 mmol, 1.5 equiv) was added to a –78 °C solution of (*E*)-ethyl 2-(phenylsulfinyl)cyclopropanecarboxylate **6** (95 mg, 0.40 mmol, 1.0 equiv) in THF (4.0 mL) over 10 s and the solution stirred for 10 min. A solution of di(2-pyridyl)ketone (147 mg, 0.80 mmol, 2.0 equiv) in toluene (1.0 mL) was added and the reaction stirred at 0 °C for 3 h. The reaction was warmed to rt, sat. aq. NH_4Cl (5 mL) added and the mixture stirred for 5 min. Water (10 mL) was added and the mixture extracted with EtOAc (3 × 30 mL). The combined organic phases were washed with brine, dried ($MgSO_4$), filtered and concentrated under reduced pressure. Purification by flash column chromatography (1:1 pentane:Et_2O) gave cyclopropane **19g** (107 mg, 90%) as a colourless oil. R_f = 0.23 (1:1 pentane:Et_2O). IR (film)/cm^{-1} 3319 (OH), 3057 (CH), 2982 (CH), 1718 (C=O), 1587, 1571, 1433, 1177, 749. ^1H NMR (400 MHz, $CDCl_3$) δ 8.52–8.49 (m, 2 H, 2 × Ar-H), 7.85–7.82 (m, 2 H, 2 × Ar-H), 7.69–7.64 (m, 2 H, 2 × Ar-H), 7.18–7.14 (m, 2 H, 2 × Ar-H), 6.28 (br s, 1 H, OH), 4.11–4.00 (m, 2 H, C$H_2$$CH_3$), 2.85 (ddd, *J* = 9.0, 6.5, 4.4 Hz, 1 H, H_c), 1.83–1.79 (m, 1 H, H_d), 1.22–1.16 (m, 4 H, H_a + CH_3), 1.10 (ddd, *J* = 8.8, 4.8, 3.9 Hz, 1 H, H_b). ^{13}C NMR (101 MHz, $CDCl_3$) δ 174.2 (C=O), 163.0 (Ar-C quat), 162.9 (Ar-C quat), 147.53 (Ar-C), 147.47 (Ar-C), 136.8 (2 × Ar-C), 122.2 (2 × Ar-C), 120.9 (2 × Ar-C), 74.8 (C(OH)), 60.2 (CH_2CH_3), 31.3 (C(H_c)), 16.6 (C(H_d)), 14.2 (CH_3), 11.2 (C(H_a)(H_b)). HRMS (EI) *m/z* Calcd for $C_{17}H_{18}N_2O_3^+$ $[M]^+$: 298.1317; Found: 298.1315.

(*E*)-Ethyl 2-benzoylcyclopropane-1-carboxylate (19h)

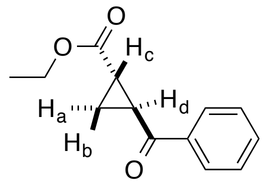

i-PrMgCl (0.32 mL, 1.85 M in THF, 0.60 mmol, 1.5 equiv) was added to a –78 °C solution of (*E*)-ethyl 2-(phenylsulfinyl)cyclopropanecarboxylate **6** (95 mg, 0.40 mmol, 1.0 equiv) in THF (4.0 mL) over 10 s and the solution stirred for 10 min. Benzoyl chloride (119 μL, 0.80 mmol, 2.0 equiv) was added and the reaction stirred at 0 °C for 3 h. The reaction was warmed to rt, sat. aq NH_4Cl (5 mL) added and the mixture stirred for 5 min. Water (10 mL) was added and the mixture extracted with EtOAc (5 × 30 mL). The combined organic phases were washed with brine, dried ($MgSO_4$), filtered and concentrated under reduced pressure. Purification by flash chromatography (9:1–2:1 *n*-hexane:EtOAc) gave cyclopropane **19h** (44 mg, 38%) as a colourless oil. R_f = 0.22 (9:1 *n*-hexane:EtOAc). IR (film)/cm^{-1} 3063 (CH), 2982 (CH), 1724 (C=O), 1672, 1332, 1207, 1004. ^1H NMR (400 MHz, $CDCl_3$) δ 8.04–8.02 (m, 2 H, 2 × Ph-H), 7.62–7.58 (m, 1 H, Ph-H), 7.50 (t, *J* = 7.6 Hz, 2 H, 2 × Ph-H), 4.19 (q, *J* = 7.2 Hz, 2 H, C$H_2$$CH_3$), 3.20 (ddd, *J* = 8.7, 5.8, 3.9 Hz, 1 H, H_c), 2.39 (ddd, *J* = 8.7, 5.9, 3.9 Hz, 1 H, H_d), 1.66–1.58 (m, 2 H, H_a + H_b), 1.30 (t, *J* = 7.1 Hz, 3 H, CH_3). ^{13}C NMR (101 MHz, $CDCl_3$) δ 197.0 (C=O ketone), 172.3 (C=O ester), 137.0 (Ph-C quat), 133.3 (Ph-C), 128.6 (2 × Ph-C), 128.2 (2 × Ph-C), 61.1 (CH_2CH_3), 25.9 (C(H_c)), 24.7 (C(H_d)), 17.9 (C(H_a)(H_b)), 14.2 (CH_3). FTMS (+p APCI) *m/z* Calcd for $C_{13}H_{15}O_3^+$ $[M+H]^+$: 219.1016; Found: 219.1016. The observed data (^1H) was consistent with that previously reported.[11]

(*E*)-Ethyl 2-(phenylcarbamoyl)cyclopropane-1-carboxylate (19i)

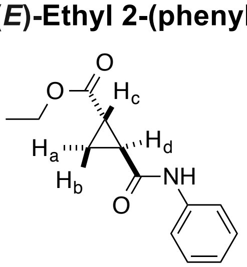

i-PrMgCl (0.32 mL, 1.85 M in THF, 0.60 mmol, 1.5 equiv) was added to a –78 °C solution of (*E*)-ethyl 2-(phenylsulfinyl)cyclopropanecarboxylate **6** (95 mg, 0.40 mmol, 1.0 equiv) in THF (4.0 mL) over 10 s and the solution stirred for 10 min. Phenyl isocyanate (87 μL, 0.80 mmol, 2.0 equiv) was added and the reaction stirred at 0 °C for 6 h. The reaction was warmed to rt, sat. aq. NH_4Cl (5 mL) added and the mixture stirred for 5 min. Water (20 mL) was added and the mixture extracted with Et_2O (5 × 30 mL). The combined organic phases were washed with brine, dried ($MgSO_4$), filtered and concentrated under reduced pressure. Purification by flash column chromatography (1:1 pentane:Et_2O) gave cyclopropane **19i** (70 mg, 75%) as a white solid. R_f = 0.31 (1:1 pentane:Et_2O). mp = 90–91°C. IR (film)/cm^{-1} 3282 (NH), 2977 (CH), 2934 (CH), 1722 (C=O), 1652, 1440, 1365, 1338, 1257, 1206, 1185, 1172, 988, 939, 750, 693. ^1H NMR (400 MHz, $CDCl_3$) δ 7.97 (br s, 1 H, NH), 7.53–7.51 (m, 2 H, 2 × Ph-H), 7.33–7.30 (m, 2 H, 2 × Ph-H), 7.13–7.09 (t, *J* = 7.4 Hz, 1 H, Ph-H), 4.19 (q, *J* = 7.1 Hz, 2 H, C$H_2$$CH_3$), 2.28 (ddd, *J* = 9.1, 5.7, 3.7 Hz, 1 H, H_c), 2.12 (ddd, *J* = 9.0, 5.8, 3.9 Hz, 1 H, H_d), 1.58 (ddd, *J* = 9.0, 5.8, 3.8 Hz, 1 H, H_b), 1.43 (ddd, *J* = 8.9, 5.7, 3.9 Hz, 1 H, H_a), 1.30 (t, *J* = 7.1 Hz, 3 H, CH_3). ^{13}C NMR (101 MHz, $CDCl_3$) δ 173.0 (C=O ester), 168.4 (C=O amide), 137.8 (Ph-C quat), 129.0 (2 × Ph-C), 124.3 (Ph-C), 119.7 (2 × Ph-C), 61.3 (CH_2CH_3), 25.1

(C(H_d)), 22.0 (C(H_c)), 15.3 (C(H_a)(H_b)), 14.2 (CH_3). HRMS (ES) *m/z* Calcd for $C_{13}H_{16}NO_3^+$ $[M+H]^+$: 234.1130; Found: 234.1137.

(*E*)-Ethyl 2-[(4-methoxyphenyl)sulfanyl]cyclopropane-1-carboxylate (19j)

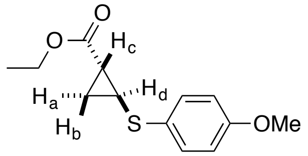

i-PrMgCl (0.32 mL, 1.85 M in THF, 0.60 mmol, 1.5 equiv) was added to a −78 °C solution of (*E*)-ethyl 2-(phenylsulfinyl)cyclopropanecarboxylate **6** (95 mg, 0.40 mmol, 1.0 equiv) in THF (4.0 mL) over 10 s and the solution stirred for 10 min. A solution of bis(4-methoxyphenyl)disulfide (223 mg, 0.80 mmol, 2.0 equiv) in THF (0.5 mL) was added and the reaction stirred at 0 °C for 6 h. The reaction was warmed to rt, sat. aq. NH_4Cl (5 mL) added and the mixture stirred for 5 min. Water (20 mL) was added and the mixture extracted with EtOAc (5 × 30 mL). The combined organic phases were washed with brine, dried ($MgSO_4$), filtered and concentrated under reduced pressure. Purification by flash column chromatography (15:1 pentane:Et_2O) gave cyclopropane **19j** (55 mg, 55%) as a yellow oil. R_f = 0.24 (9:1 pentane:Et_2O). IR (film)/cm^{-1} 2981 (CH), 2961 (CH), 2836 (CH), 1721 (C=O), 1494, 1242, 1173, 1030, 821. ^1H NMR (400 MHz, $CDCl_3$) δ 7.34–7.31 (m, 2 H, 2 × Ar-H), 6.89–6.85 (m, 2 H, 2 × Ar-H), 4.20–4.09 (m, 2 H, CH_2CH_3), 3.81 (s, 3 H, OCH_3), 2.74 (ddd, *J* = 8.3, 5.7, 3.6 Hz, 1 H, H_c), 1.90 (ddd, *J* = 8.8, 5.3, 3.5 Hz, 1 H, H_d), 1.57 (ddd *J* = 8.3, 5.1, 5.1 Hz, 1 H, H_b), 1.27 (t, *J* = 7.2 Hz, 3 H, CH_2CH_3), 1.21 (ddd, *J* = 8.8, 5.6, 4.9 Hz, 1 H, H_a). ^{13}C NMR (101 MHz, $CDCl_3$) δ 172.3 (C=O), 158.8 (Ar-*C*-OCH_3), 131.2 (2 × Ar-C), 126.5 (Ar-C quat), 114.7 (2 × Ar-C), 60.8 (CH_2CH_3), 55.3 (OCH_3), 24.5 (C(H_d)), 24.2 (C(H_c)), 17.2 (C(H_a)(H_b)), 14.2 (CH_2CH_3). FTMS (+p APCI) *m/z* Calcd for $C_{13}H_{17}O_3S^+$ $[M+H]^+$: 253.0893; Found: 253.0891.

(*E*)-Ethyl 2-formylcyclopropane-1-carboxylate (19k)

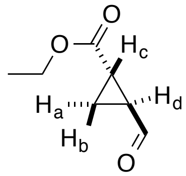

i-PrMgCl (0.32 mL, 1.85 M in THF, 0.60 mmol, 1.5 equiv) was added to a −78 °C solution of (*E*)-ethyl 2-(phenylsulfinyl)cyclopropanecarboxylate **6** (95 mg, 0.40 mmol, 1.0 equiv) in THF (4.0 mL) over 10 s and the solution stirred for 10 min. *N*,*N*-Dimethylformamide (62 μL, 0.80 mmol, 2.0 equiv) was added and the reaction stirred at 0 °C for 6 h. The reaction was warmed to rt, sat. aq. NH_4Cl (5 mL) added and the mixture stirred for 5 min. H_2O (20 mL) was added and the mixture extracted with ether (5 × 30 mL). The combined organic phases were washed with brine, with the aqueous being extracted with Et_2O (30 mL). The combined organic phases were dried ($MgSO_4$), filtered and concentrated under reduced pressure. Purification by flash column chromatography (5:1 pentane:Et_2O) gave cyclopropane **19k** (48 mg, 85%) as a colourless oil. R_f = 0.17 (5:1 pentane:Et_2O). IR (film)/cm^{-1} 2984 (CH), 2849 (CH), 2738 (CH), 1182, 980. ^1H NMR (400 MHz, $CDCl_3$) δ 9.31 (d, *J* = 4.2 Hz, 1 H, C(O)H), 4.18 (q, *J* = 7.1 Hz, 2 H, CH_2CH_3), 2.44 (dddd, *J* = 8.5, 5.8, 4.2, 4.0 Hz, 1 H, H_d), 2.26 (ddd, *J* = 8.9, 6.0, 3.9 Hz, 1 H, H_c), 1.61 (ddd, *J* = 8.5, 5.9, 4.3 Hz, 1 H, H_a), 1.51 (ddd, *J* = 8.9, 5.7, 4.4 Hz, 1 H, H_b), 1.28 (t, *J* = 7.2 Hz, 3 H, CH_3). ^{13}C NMR (101 MHz, $CDCl_3$) δ 198.1 (C=O aldehyde), 171.0 (C=O ester), 61.3 (CH_2CH_3), 30.6 (C(H_d)), 22.2 (C(H_c)), 14.8 (C(H_a)(H_b)), 14.1 (CH_3). The observed data (^1H, ^{13}C) was consistent with that previously reported.[12,13]

(*E*)-Ethyl 2-(4,4,5,5-tetramethyl-1,3,2-dioxaborolan-2-yl)cyclopropane-1-carboxylate (19l)

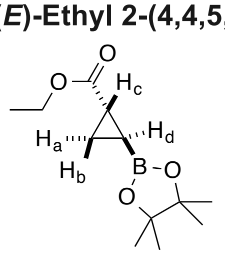

i-PrMgCl (0.32 mL, 1.85 M in THF, 0.60 mmol, 1.5 equiv) was added to a −78 °C solution of (*E*)-ethyl 2-(phenylsulfinyl)cyclopropanecarboxylate **6** (95 mg, 0.40 mmol, 1.0 equiv) in THF (4.0 mL) over 10 s and the solution stirred for 10 min. 2-Isopropoxy-4,4,5,5-tetramethyl-1,3,2-dioxaborolane (163 μL, 0.80 mmol, 2.0 equiv) was added and the reaction stirred at 0 °C for 6 h. The reaction was warmed to rt, MeOH (1 mL) added and stirred for 5 min. Et_2O (10 mL) was added and the mixture filtered, then concentrated under reduced pressure. Purification by flash column chromatograpy (4:1 pentane:Et_2O) gave cyclopropane **19l** (52mg, 54%) as a colourless oil. IR (film)/cm^{-1} 2980 (CH), 2932 (CH), 1727 (C=O), 1424, 1370, 1325, 1177, 1141, 855. ^1H NMR (400 MHz, $CDCl_3$) δ 4.17–4.05 (m, 2 H, CH_2CH_3), 1.75 (ddd, *J* = 7.7, 4.9, 4.9 Hz, 1 H, H_c), 1.26–1.19 (m, 16 H, H_d + 5 × CH_3), 0.98 (ddd, *J* = 7.6, 7.6, 3.0 Hz, 1 H, H_b), 0.57 (ddd, *J* = 10.1, 7.3, 5.3 Hz, 1 H, H_a). ^{13}C NMR (101 MHz, $CDCl_3$) δ 174.2 (C=O), 83.4 (2 × *C*(CH_3)$_2$), 60.5 (CH_2CH_3), 29.7 (C(H_d)), 24.7 (2 × CH_3), 24.6 (2 × CH_3), 18.5 (C(H_c)), 14.2 (CH_2CH_3), 13.0 (C(H_a)(H_b)). ^{11}B NMR (128 MHz, $CDCl_3$) δ 32.58 (B(pin)). HRMS (EI) *m/z* Calcd for $C_{12}H_{21}{}^{11}BO_4^+$ $[M]^+$: 240.1533; Found: 240.1528.

(*E*)-Ethyl 2-(triethoxysilyl)cyclopropane-1-carboxylate (19m)

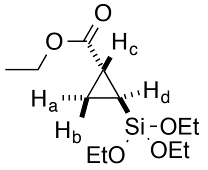

i-PrMgCl (0.32 mL, 1.85 M in THF, 0.60 mmol, 1.5 equiv) was added to a –78 °C solution of (*E*)-ethyl 2-(phenylsulfinyl)cyclopropanecarboxylate **6** (95 mg, 0.40 mmol, 1.0 equiv) in THF (4.0 mL) over 10 s and the solution stirred for 10 min. Chlorotriethoxysilane (157 μL, 0.80 mmol, 2.0 equiv) was added and the reaction stirred at 0 °C for 6 h. The reaction was warmed to rt, sat. aq. NH_4Cl (5 mL) added and the mixture stirred for 5 min. Water (20 mL) was added and the mixture extracted with EtOAc (5 × 30 mL). The combined organic phases were washed with brine, dried ($MgSO_4$), filtered and concentrated under reduced pressure. Purification by flash column chromatography (4:1 pentane:Et_2O) gave cyclopropane **19m** (55 mg, 43%) as a pale yellow oil. IR (film)/cm^{-1} 2976 (CH), 2934 (CH), 1722 (C=O), 1389, 1264, 1166, 1100, 1073, 953, 776. ^{1}H NMR (400 MHz, $CDCl_3$) δ 4.14 (q, *J* = 7.1 Hz, 2 H, (C=O)OCH_2), 3.91–3.81 (m, 6 H, 3 × $SiOCH_2$), 1.74 (ddd, *J* = 7.5, 5.9, 4.3 Hz, 1 H, H_c), 1.28–1.18 (m, 13 H, H_a + 4 × CH_3), 0.96 (ddd, *J* = 7.7, 7.7, 3.1 Hz, 1 H, H_b), 0.36 (ddd, *J* = 10.8, 8.2, 5.9 Hz, 1 H, H_d). ^{13}C NMR (101 MHz, $CDCl_3$) δ 174.6 (C=O), 60.5 (CH_2), 59.1 (CH_2), 58.7 (2 × CH_2), 18.2 (2 × CH_3), 18.0 (CH_3), 16.3 (C(H_c)), 14.2 (CH_3), 11.2 (C(H_a)(H_b)), 2.9 (C(H_d)). HRMS (EI) *m/z* Calcd for $C_{12}H_{24}O_5Si^+$ $[M]^+$: 276.1393; Found: 276.1405.

(*Z*)-Ethyl-2-iodocyclopropanecarboxylate (20a)

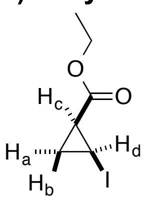

i-PrMgCl (0.32 mL, 1.87 M in THF, 0.60 mmol, 1.5 equiv) was added to a −78 °C solution of (*Z*)-ethyl 2-(phenylsulfinyl)cyclopropanecarboxylate **7** (95 mg, 0.40 mmol, 1.0 equiv) in THF (4.0 mL) over 10 s and the solution stirred for 10 min. A solution of I_2 (203 mg, 0.80 mmol, 2.0 equiv) in THF (0.5 mL) was added and the reaction stirred at −78 °C for 1 h. The reaction was warmed to rt, sat. aq NH_4Cl (5 mL) added and the mixture was stirred for 5 min. Water (10 mL) was added and the mixture extracted with CH_2Cl_2 (3 × 30 mL). The combined organic phases were washed with brine, dried ($MgSO_4$), filtered and concentrated under reduced pressure. Purification by flash column chromatography (100% *n*-hexane to 10:1 *n*-hexane:Et_2O) gave cyclopropane **20a** (56 mg, 58%) as a yellow oil. IR (film)/cm^{-1} 2981 (CH), 1726 (C=O), 1396, 1380, 1248, 1175. ^{1}H NMR (400 MHz, $CDCl_3$) δ 4.30–4.17 (m, 2 H, CH_2CH_3), 2.82 (ddd, *J* = 8.1, 8.1, 6.5 Hz, 1 H, H_d), 1.87 (ddd, *J* = 8.3, 8.3, 6.5 Hz, 1 H, H_c), 1.52 (ddd, *J* = 8.2, 8.2, 6.2 Hz, 1 H, H_a), 1.42 (ddd, *J* = 6.4, 6.4, 6.4 Hz, 1 H, H_b), 1.32 (t, *J* = 7.1 Hz, 3 H, CH_3). ^{13}C NMR (101 MHz, $CDCl_3$) δ 169.9 (C=O), 61.2 (CH_2), 19.2 (C(H_c)), 16.3 (C(H_a)(H_b)), 14.4 (CH_3), −14.6 (C(H_d)). HRMS (ES) *m/z* Calcd for $C_6H_{10}O_2I^+$ $[M+H]^+$: 240.9726; Found: 240.9710. The observed data (IR, ^{1}H, ^{13}C) was consistent with that previously reported.[14]

(*Z*)-4-Cyclopentyl-3-oxabicyclo[3.1.0]hexan-2-one (20b)

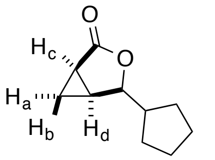

i-PrMgCl (0.32 mL, 1.87 M in THF, 0.60 mmol, 1.5 equiv) was added to a –78 °C solution of (*Z*)-ethyl 2-(phenylsulfinyl)cyclopropanecarboxylate **7** (95 mg, 0.40 mmol, 1.0 equiv) in THF (4.0 mL) over 10 s and the solution stirred for 10 min. Cyclopentanecarboxaldehyde (85 µL, 0.80 mmol, 2.0 equiv) was added and the reaction stirred at 0 °C for 1 h. The reaction was warmed to 25 °C, sat. aq NH_4Cl (5 mL) added and the mixture was stirred for 5 min. Water (10 mL) was added and the mixture extracted with CH_2Cl_2 (5 × 30 mL). The combined organic phases were washed with brine, dried ($MgSO_4$), filtered and concentrated under reduced pressure. Purification by flash column chromatography (2:1 pentane:Et_2O) gave cyclopropane **20b** as a mixture of two diastereoisomers (d.r = 60:40, 57 mg, 86%) as a yellow oil. R_f = 0.24 (2:1 pentane:Et_2O). IR (film)/cm^{-1} 2953 (CH), 2869 (CH), 1760 (C=O), 1452, 1330, 1183, 959.

<u>*Major diastereoisomer:*</u> ^{1}H NMR (400 MHz, $CDCl_3$) δ 4.18 (d, *J* = 7.5 Hz, 1 H, C*H*(CH_d)), 2.12–2.03 (m, 1 H, C*H*(CH_2)$_2$), 2.06–2.03 (m, 2 H, H_c + H_d), 1.88–1.76 (m, 2 H, CH_2), 1.69–1.53 (m, 4 H, 2 × CH_2), 1.45–1.35 (m, 2 H, CH_2), 1.28–1.19 (m, 1 H, H_a), 0.84 (dd, *J* = 4.7, 4.0 Hz, 1 H, H_b). ^{13}C NMR (101 MHz, $CDCl_3$) δ 176.2 (C=O), 84.7 (*C*H(CH_d)), 45.0 (*C*H(CH_2)$_2$), 28.0 (CH_2), 27.7 (CH_2), 25.5 (CH_2), 25.3 (CH_2), 21.5 (C(H_d)), 17.8 (C(H_c)), 12.2 (C(H_a)(H_b)).

<u>*Minor diastereoisomer:*</u> ^{1}H NMR (400 MHz, $CDCl_3$) δ 4.28 (dd, *J* = 9.6, 4.4 Hz, 1 H, C*H*(CH_d)), 2.21–2.16 (m, 1 H, H_c), 2.06–2.03 (m, 1 H, H_d), 1.97–1.88 (m, 1 H, C*H*(CH_2)$_2$), 1.88–1.76 (m, 2 H, CH_2), 1.69–1.53 (m, 4 H, 2 × CH_2), 1.45–1.35 (m, 2 H, CH_2), 1.11–1.06 (m, 1 H, H_a), 0.96 (ddd, *J* = 4.7, 4.7, 3.2 Hz, 1 H, H_b). ^{13}C NMR (101 MHz, $CDCl_3$) δ 176.3 (C=O), 84.1 (*C*H(CH_d)), 42.2 (*C*H(CH_2)$_2$), 30.5 (CH_2), 28.1 (CH_2), 25.4 (CH_2), 25.1 (CH_2), 20.8 (C(H_d)), 18.5 (C(H_c)), 8.8 (C(H_a)(H_b)).

FTMS (APCI) *m/z* Calcd for $C_{10}H_{15}O_2^+$ $[M+H]^+$: 167.1067; Found: 167.1068.

(*Z*)-4-Phenyl-3-oxabicyclo[3.1.0]hexan-2-one (20c)

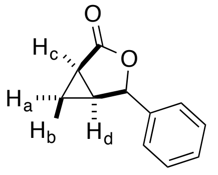

i-PrMgCl (0.32 mL, 1.87 M in THF, 0.60 mmol, 1.5 equiv) was added to a −78 °C solution of (*Z*)-ethyl 2-(phenylsulfinyl)cyclopropanecarboxylate **7** (95 mg, 0.40 mmol, 1.0 equiv) in THF (4.0 mL) over 10 s and the solution stirred for 10 min. Benzaldehyde (81 μL, 0.80 mmol, 2.0 equiv) was added and the reaction stirred at 0 °C for 1 h. The reaction was warmed to rt, sat. aq. NH_4Cl (5 mL) added and the mixture was stirred for 5 min. H_2O (10 mL) was added and the mixture extracted with CH_2Cl_2 (3 × 30 mL). The combined organic phases were washed with brine, dried ($MgSO_4$), filtered and concentrated under reduced pressure. Purification by flash column chromatography (10:1 *n*-hexane:Et_2O) gave cyclopropane **20c** as a mixture of two diastereoisomers (d.r = 56:44, 48 mg, 69%) as a white solid. R_f = 0.59 (1:1 pentane:EtOAc). mp = 33–34 °C. IR (film)/cm^{-1} 3034 (CH), 3008 (CH), 2930 (CH), 1762 (C=O), 1454, 1312, 1180, 969.

<u>*Major diastereoisomer:*</u> ^{1}H NMR (400 MHz, $CDCl_3$) δ 7.44–7.32 (m, 5 H, 5 × Ph-H), 5.34 (s, 1 H, C*H*(CH_d)), 2.31–2.24 (m, 1 H, H_d), 2.29–2.21 (m, 1 H, H_c), 1.37 (ddd, *J* = 8.9, 7.6, 4.9 Hz, 1 H, H_a), 1.10 (ddd, *J* = 4.7, 4.7, 3.5 Hz, 1 H, H_b). ^{13}C NMR (101 MHz, $CDCl_3$) δ 175.9 (C=O), 139.7 (Ph-C quat), 128.9 (2 × Ph-C), 128.8 (Ph-C), 125.5 (2 × Ph-C), 81.6 (*C*H(CH_d)), 24.7 (C(H_d)), 17.8 (C(H_c)), 12.9 (C(H_a)(H_b)).

<u>*Minor diastereoisomer:*</u> ^{1}H NMR (400 MHz, $CDCl_3$) δ 7.44–7.32 (m, 5 H, 5 × Ph-H), 5.75 (d, *J* = 4.9 Hz, 1 H, C*H*(CH_d)), 2.57 (dddd, *J* = 7.5, 4.9, 4.9, 4.9 Hz, 1 H, H_d), 2.31–2.21 (m, 1 H, H_c), 1.14 (ddd, *J* = 9.0, 7.5, 5.1 Hz, 1 H, H_a), 0.89 (ddd, *J* = 5.1, 4.9, 3.3 Hz, 1 H, H_b). ^{13}C NMR (101 MHz, $CDCl_3$) δ 175.6 (C=O), 137.4 (Ph-C quat), 128.5 (2 × Ph-C), 128.1 (Ph-C), 125.4 (2 × Ph-C), 79.2 (*C*H(CH_d)), 22.2 (C(H_d)), 19.1 (C(H_c)), 9.7 (C(H_a)(H_b)).

HRMS (EI) *m/z* Calcd for $C_{11}H_{10}O_2^+$ $[M]^+$: 174.0681; Found: 174.0674. The observed data (IR, ^{1}H, ^{13}C) was consistent with that previously reported.[15]

(*Z*)-Ethyl 2-[hydroxy(pyridine-3-yl)methyl]cyclopropane-1-carboxylate (20d)

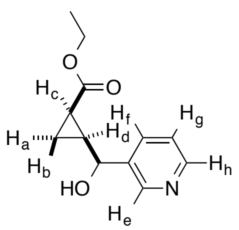

i-PrMgCl (0.32 mL, 1.87 M in THF, 0.60 mmol, 1.5 equiv) was added to a −78 °C solution of (*Z*)-ethyl 2-(phenylsulfinyl)cyclopropanecarboxylate **7** (95 mg, 0.40 mmol, 1.0 equiv) in THF (4.0 mL) over 10 s and the solution stirred for 10 min. 3-Pyridinecarboxaldehyde (75 μL, 0.80 mmol, 2.0 equiv) was added and the reaction stirred at 0 °C for 1 h. The reaction was warmed to rt, sat. aq. NH_4Cl (5 mL) added and the mixture stirred for 5 min. Water (10 mL) was added and the mixture extracted with CH_2Cl_2 (6 × 20 mL) then EtOAc (6 × 20 mL). The combined organic phases were concentrated under reduced pressure, dissolved in CH_2Cl_2, washed with brine, dried ($MgSO_4$), filtered and concentrated under reduced pressure. Purification by flash column chromatography (3:1 EtOAc:toluene) gave cyclopropane **20d** as a mixture of two diastereomers (d.r = 57:43, 76 mg, 86%) as a colourless oil. R_f = 0.23 (3:1 EtOAc:toluene). IR (film)/cm^{-1} 3184 (OH), 2981 (CH), 2929 (CH), 2848 (CH), 1774 (C=O), 1183.

<u>*Major diastereoisomer:*</u> ^{1}H NMR (400 MHz, $CDCl_3$) δ 8.62–8.45 (m, 2 H, 2 × Ar-H), 7.80–7.63 (m, 1 H, Ar-H), 7.34–7.24 (m, 1 H, Ar-H), 5.72 (d, *J* = 4.8 Hz, 1 H, C*H*(OH)), 4.16–4.07 (m, 2 H, C*H*$_2$CH$_3$), 1.90 (ddd, *J* = 8.3, 8.3, 5.8 Hz, 1 H, H_c), 1.70–1.62 (m, 1 H, H_d), 1.26–1.09 (m, 4 H, H_a + CH_3), 0.80–0.79 (m, 1 H, H_b). ^{13}C NMR (101 MHz, $CDCl_3$) δ 173.2 (C=O), 149.4 (Ar-C), 147.5 (Ar-C), 139.8 (Ar-C quat), 133.6 (Ar-C), 123.4 (Ar-C), 77.1 (CH(OH)), 60.8 (*C*H$_2$CH$_3$), 28.8 (C(H_d)), 19.0 (C(H_c)), 12.5 (CH_3), 9.7 (C(H_a)(H_b)).

<u>*Minor diastereoisomer:*</u> ^{1}H NMR (400 MHz, $CDCl_3$) δ 8.62–8.45 (m, 2 H, 2 × Ar-H), 7.80–7.63 (m, 1 H, Ar-H), 7.34–7.24 (m, 1 H, Ar-H), 4.81 (d, *J* = 9.2 Hz, 1 H, C*H*(OH)), 4.16–4.07 (m, 2 H, C*H*$_2$CH$_3$), 2.62–2.57 (m, 1 H, H_d), 2.25 (ddd, *J* = 6.4, 4.1, 3.6 Hz, 1 H, H_c), 1.46–1.36 (m, 1 H, H_a), 1.24 (t, *J* = 6.8 Hz, 3 H, CH_3), 1.17–1.09 (m, 1 H, H_b). ^{13}C NMR (101 MHz, $CDCl_3$) δ 174.9 (C=O), 148.5 (Ar-C), 146.9 (Ar-C), 135.1 (Ar-C quat), 133.4 (Ar-C), 123.7 (Ar-C), 79.1 (CH(OH)), 69.6 (*C*H$_2$CH$_3$), 21.6 (C(H_d)), 18.5 (C(H_c)), 14.1 (CH_3), 12.8 (C(H_a)(H_b)).

HRMS (APCI) *m/z* Calcd for $C_{12}H_{16}NO_3^+$ $[M+H]^+$: 222.1125; Found: 222.1124.

(*Z*)-4,4-Diethyl-3-oxabicyclo[3.1.0]hexan-2-one (20e)

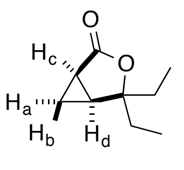

i-PrMgCl (0.32 mL, 1.82 M in THF, 0.60 mmol, 1.5 equiv) was added to a −78 °C solution of (*Z*)-ethyl 2-(phenylsulfinyl)cyclopropanecarboxylate **7** (95 mg, 0.40 mmol, 1.0 equiv) in THF (4.0 mL) over 10 s and the solution stirred for 10 min. Pentan-3-one (85 μL, 0.80 mmol, 2.0 equiv) was added and the reaction stirred at 0 °C for 3 h. The reaction was warmed to rt, sat. aq. NH_4Cl (5 mL) added and the mixture stirred for 5 min. Water (20 mL) was added and the mixture extracted with EtOAc (5 × 20 mL). The combined organic phases were washed with brine, dried ($MgSO_4$), filtered and concentrated under reduced pressure. Purification by flash column chromatography (2:1 pentane:Et_2O) gave cyclopropane **20e** (30 mg, 49%) as a pale yellow oil. R_f = 0.23 (2:1 pentane:Et_2O). IR (film)/cm^{-1} 2973 (CH), 2942 (CH), 2885 (CH), 1766 (C=O), 1463, 1233, 1000, 941. ^{1}H NMR (400 MHz, $CDCl_3$) δ 2.12 (ddd, *J* = 8.9, 5.7, 3.1 Hz, 1 H, H_c), 1.99–1.95 (m, 1 H, H_d), 1.87–1.71 (m, 3 H, 3 × C(*H*)H), 1.61–1.52 (m, 1 H, C(*H*)H), 1.11 (ddd, *J* = 8.9, 7.6, 5.1 Hz, 1 H, H_a), 1.01–0.94 (m, 7 H, H_b + 2 × CH_3). ^{13}C NMR (101 MHz, $CDCl_3$) δ 176.0 (C=O), 87.7 (*C*(CH_2CH_3)$_2$), 30.9 (*C*$H_2$$CH_3$), 28.1 (*C*$H_2$$CH_3$), 24.7 (C($H_d$)), 19.1 (C($H_c$)), 9.7 (C($H_a$)($H_b$)), 8.4 ($CH_3$), 7.1 ($CH_3$). HRMS (CI+) *m/z* Calcd for $C_9H_{15}O_2^+$ [M+H]$^+$: 155.1072; Found: 155.1071.

(*E*)-4,4-Bis(4-chlorophenyl)-3-oxabicyclo[3.1.0]hexan-2-one (20f)

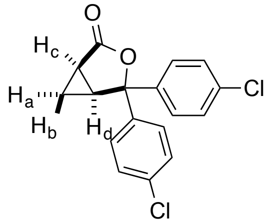

i-PrMgCl (0.32 mL, 1.87 M in THF, 0.60 mmol, 1.5 equiv) was added to a −78 °C solution of (*Z*)-ethyl 2-(phenylsulfinyl)cyclopropanecarboxylate **7** (95 mg, 0.40 mmol, 1.0 equiv) in THF (4.0 mL) over 10 s and the solution stirred for 10 min. 4,4-Dichlorobenzophenone (201 mg, 0.80 mmol, 2.0 equiv) in toluene (1.0 mL) was added and the reaction stirred at 0 °C for 3 h. The reaction was warmed to rt, sat. aq. NH_4Cl (5 mL) added and the mixture stirred for 5 min. Water (20 mL) was added and the mixture extracted with EtOAc (5 × 20 mL). The combined organic phases were washed with brine, dried ($MgSO_4$), filtered and concentrated under reduced pressure. Purification by flash column chromatography (2:1 pentane:Et_2O–100% Et_2O) gave cyclopropane **20f** (83 mg, 65%) as a white solid. mp = 153–155 °C. R_f = 0.17 (1:2 pentane:Et_2O). IR (film)/cm^{-1} 2968 (CH), 2932 (CH), 1644 (C=O), 1583, 1443, 1365, 1087, 1020, 748, 691. ^{1}H NMR (400 MHz, $CDCl_3$) δ 7.42–7.26 (m, 8 H, 8 × Ar-H), 2.75 (ddd, *J* = 7.5, 5.5, 4.6 Hz, 1 H, H_c), 2.35 (ddd, *J* = 9.0, 5.5, 3.4 Hz, 1 H, H_d), 1.43 (ddd, *J* = 9.0, 7.5, 5.3 Hz, 1 H, H_a), 0.94 (ddd, *J* = 5.2, 4.7, 3.4 Hz, 1 H, H_b). ^{13}C NMR (101 MHz, $CDCl_3$) δ 174.5 (C=O), 142.4 (Ar-C quat), 139.4 (Ar-C quat), 134.3 (Ar-C quat), 134.2 (Ar-C quat), 128.9 (2 × Ar-C), 128.8 (2 × Ar-C), 128.0 (2 × Ar-C), 126.7 (2 × Ar-C), 87.6 (*C*(Ar)$_2$), 27.9 (C(H_c)), 19.8 (C(H_d)), 13.1 (C(H_a)(H_b)). HRMS (ASAP+) *m/z* Calcd for $C_{17}H_{13}O_2Cl_2^+$ [M+H]$^+$: 319.0293; Found: 319.0290.

(*Z*)-Ethyl 2-[hydroxydi(pyridin-2-yl)methyl]cyclopropane-1-carboxylate (20g) and (*Z*)-4,4-Di(pyridin-2-yl)-3-oxabicyclo[3.1.0]hexan-2-one (20g')

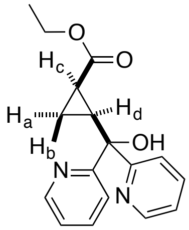

20g

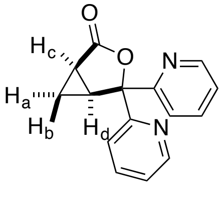

20g'

i-PrMgCl (0.32 mL, 1.85 M in THF, 0.60 mmol, 1.5 equiv) was added to a −78 °C solution of (*Z*)-ethyl 2-(phenylsulfinyl)cyclopropanecarboxylate **7** (95 mg, 0.40 mmol, 1.0 equiv) in THF (4.0 mL) over 10 s and the solution stirred for 10 min. A solution of di(2-pyridyl)ketone (147 mg, 0.80 mmol, 2.0 equiv) in toluene (1.0 mL) was added and the reaction stirred at 0 °C for 3 h. The reaction was warmed to rt, sat. aq. NH_4Cl (5 mL) added and the mixture stirred for 5 min. Water (10 mL) was added and the mixture extracted with EtOAc (3 × 30 mL). The combined organic phases were washed with brine, dried ($MgSO_4$), filtered and concentrated under reduced pressure. Purification by flash column chromatography (EtOAc) gave alcohol **20g** (51 mg, 43%) as a white oil followed by lactone **20g'** (15 mg, 15%) as a yellow gum.

(Z)-Ethyl 2-[hydroxydi(pyridin-2-yl)methyl]cyclopropane-1-carboxylate (20g)

R_f = 0.54 (EtOAc). IR (film)/cm^{-1} 3339 (OH), 3058 (CH), 2982 (CH), 2935 (CH), 1728 (C=O), 1587, 1432, 1187, 1083, 994, 769, 749. ^{1}H NMR (400 MHz, $CDCl_3$) δ 8.54 (dddd, *J* = 8.2, 4.8, 1.7, 0.9 Hz, 2 H, 2 × Ar-H), 7.89 (ddd, *J* = 8.0, 0.9, 0.9 Hz, 1 H, Ar-H), 7.75 (ddd, *J* = 8.0, 0.9, 0.9 Hz, 1 H, Ar-H), 7.65 (m, 2 H, 2 × Ar-H), 7.14–7.10 (m, 2 H, 2 × Ar-H), 6.08 (br s, 1 H, OH), 3.96–3.82 (m, 2 H, C*H*$_2$$CH_3$), 3.00 (ddd, *J* = 9.0, 9.0,

7.6 Hz, 1 H, H_c), 1.91 (ddd, *J* = 8.7, 8.7, 6.2 Hz, 1 H, H_d), 1.69 (ddd, *J* = 7.4, 6.2, 4.9 Hz, 1 H, H_b), 1.15 (ddd, *J* = 8.7, 8.7, 4.9 Hz, 1 H, H_a), 1.02 (t, *J* = 7.1 Hz, 3 H, CH_3). ^{13}C NMR (101 MHz, $CDCl_3$) δ 174.8 (C=O), 164.6 (Ar-C quat), 164.0 (Ar-C quat), 148.2 (Ar-C), 147.9 (Ar-C), 136.7 (Ar-C), 136.2 (Ar-C), 121.9 (Ar-C), 121.8 (Ar-C), 121.1 (Ar-C), 120.3 (Ar-C), 77.2 (C(OH)) 60.7 (CH_2CH_3), 30.9 (C(H_c)), 19.6 (C(H_d)), 13.9 (CH_3), 9.9 (C(H_a)(H_b)). HRMS (ES) *m/z* Calcd for $C_{17}H_{19}N_2O_3^+$ $[M+H]^+$: 299.1396; Found: 299.1397.

(Z)-4,4-Di(pyridin-2-yl)-3-oxabicyclo[3.1.0]hexan-2-one (20g')

R_f = 0.22 (EtOAc). IR (film)/cm^{-1} 3059 (CH), 3005 (CH), 2926 (CH), 2854 (CH), 1773 (C=O), 1587, 1572, 1465, 1433, 1201, 1028, 948, 749, 675. ^1H NMR (400 MHz, $CDCl_3$) δ 8.67–8.65 (m, 2 H, 2 × Ar-H), 7.73–7.64 (m, 2 H, 2 × Ar-H), 7.58 (d, *J* = 7.9 Hz, 1 H, Ar-H), 7.45 (d, *J* = 7.9 Hz, 1 H, Ar-H), 7.24–7.20 (m, 2 H, 2 × Ar-H), 3.57 (ddd, *J* = 7.7, 5.4, 4.7 Hz, 1 H, H_c), 2.27 (ddd, *J* = 8.8, 5.5, 3.3 Hz, 1 H, H_d), 1.36 (ddd, *J* = 8.8, 7.7, 5.2 Hz, 1 H, H_a), 0.82 (ddd, *J* = 4.8, 4.8, 3.3 Hz, 1 H, H_b). ^{13}C NMR (101 MHz, $CDCl_3$) δ 175.2 (C=O), 160.1 (Ar-C quat), 159.3 (Ar-C quat), 149.6 (Ar-C), 149.2 (Ar-C), 136.9 (Ar-C), 136.7 (Ar-C), 123.1 (Ar-C), 122.8 (Ar-C), 120.9 (Ar-C), 120.7 (Ar-C), 88.8 (C$(Ar)_2$), 26.3 (C(H_c)), 19.3 (C(H_d)), 12.3 (C(H_a)(H_b)). HRMS (ES) *m/z* Calcd for $C_{15}H_{13}N_2O_2^+$ $[M+H]^+$: 253.0977; Found: 253.0986.

(*Z*)-Ethyl 2-benzoylcyclopropane-1-carboxylate (20h)

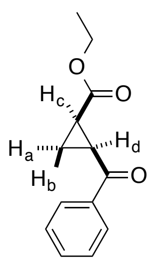

i-PrMgCl (0.32 mL, 1.85 M in THF, 0.60 mmol, 1.5 equiv) was added to a −78 °C solution of (*Z*)-ethyl 2-(phenylsulfinyl)cyclopropanecarboxylate **7** (95 mg, 0.40 mmol, 1.0 equiv) in THF (4.0 mL) over 10 s and the solution stirred for 10 min. Benzoyl chloride (119 μL, 0.80 mmol, 2.0 equiv) was added and the reaction stirred at 0 °C for 3 h. The reaction was warmed to rt, sat. aq. NH_4Cl (5 mL) added and the mixture stirred for 5 min. Water (10 mL) was added and the mixture extracted with EtOAc (5 × 30 mL). The combined organic phases were washed with brine, dried ($MgSO_4$), filtered and concentrated under reduced pressure. Purification by flash column chromatography (9:1 *n*-hexane:EtOAc) gave cyclopropane **20h** (73 mg, 64%) as a golden oil. R_f = 0.07 (9:1 *n*-hexane:EtOAc). IR (film)/cm^{-1} 3063 (CH), 2983 CH), 1727 (C=O), 1681, 1382, 1226, 1185. ^1H NMR (400 MHz, $CDCl_3$) δ 8.05–8.02 (m, 2 H, 2 × Ph-H), 7.58–7.54 (m, 1 H, Ph-H), 7.48–7.44 (m, 2 H, 2 × Ph-H), 3.99 (dddd, *J* = 7.1, 7.1, 7.1, 1.5 Hz, 2 H, CH_2CH_3), 2.79 (ddd, *J* = 9.2, 8.3, 7.0 Hz, 1 H, H_c), 2.31 (ddd, *J* = 9.3, 8.3, 6.4 Hz, 1 H, H_d), 1.92 (ddd, *J* = 6.6, 6.6, 4.8 Hz, 1 H, H_b), 1.37 (ddd, *J* = 8.3, 8.3, 4.8 Hz, 1 H, H_a), 1.05 (t, *J* = 7.1 Hz, 3 H, CH_3). ^{13}C NMR (101 MHz, $CDCl_3$) δ 194.5 (C=O ketone), 169.9 (C=O ester), 137.1 (Ph-C quat), 133.1 (Ph-C), 128.5 (2 × Ph-C), 128.3 (2 × Ph-C), 60.8 (CH_2CH_3), 26.2 (C(H_c)), 23.0 (C(H_d)), 13.9 (CH_3), 11.5 (C(H_a)(H_b)). FTMS (+p APCI) *m/z* Calcd for $C_{13}H_{15}O_3^+$ $[M+H]^+$: 219.1016; Found: 219.1014.The observed data (IR, ^1H, ^{13}C) was consistent with that previously reported.[11,15]

(*Z*)-Ethyl 2-(4,4,5,5-tetramethyl-1,3,2-dioxaborolan-2-yl)cyclopropane-1-carboxylate (20l)

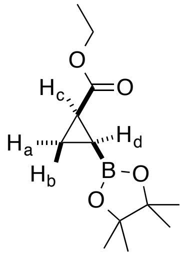

i-PrMgCl (0.32 mL, 1.85 M in THF, 0.60 mmol, 1.5 equiv) was added to a −78 °C solution of (*Z*)-ethyl 2-(phenylsulfinyl)cyclopropanecarboxylate **7** (95 mg, 0.40 mmol, 1.0 equiv) in THF (4.0 mL) over 10 s and the solution stirred for 10 min. 2-Isopropoxy-4,4,5,5-tetramethyl-1,3,2-dioxaborolane (163 μL, 0.80 mmol, 2.0 equiv) was added and the reaction stirred at 0 °C for 6 h. The reaction was warmed to rt, MeOH (1 mL) added and stirred for 5 min. The mixture was concentrated under reduced pressure, dissolved in acetonitrile (10 mL) and extracted with pentane (5 × 10 mL). The combined pentane phases were washed with acetonitrile (10 mL), then concentrated under reduced pressure to give cyclopropane **20l** (79 mg, 82%) as a colourless oil. IR (film)/cm^{-1} 2978 (CH), 2927 (CH), 2852 (CH), 1724 (C=O), 1380, 1319, 1142, 854. ^1H NMR (400 MHz, $CDCl_3$) δ 4.24–4.05 (m, 2 H, CH_2CH_3), 1.83 (ddd, *J* = 9.3, 7.8, 4.8 Hz, 1 H, H_c), 1.35–1.23 (m, 15 H, 5 × CH_3) 1.12 (ddd, *J* = 8.5, 4.7, 3.7 Hz, 1 H, H_b), 1.06 (ddd, *J* = 9.9, 7.9, 3.6 Hz, 1 H, H_a), 0.42 (ddd, *J* = 9.7, 8.8, 8.8 Hz, 1 H, H_d). ^{13}C NMR (101 MHz, $CDCl_3$) δ 174.4 (C=O), 83.5 (2 × $C(CH_3)_2$), 60.5 (CH_2CH_3), 29.7 (C(H_d)), 24.89 (2 × CH_3), 24.87 (2 × CH_3), 17.8 (C(H_c)), 14.3 (CH_2CH_3), 11.2 (C(H_a)(H_b)). ^{11}B NMR (128 MHz, $CDCl_3$) δ 32.06 (B(pin)). HRMS (EI) *m/z* Calcd for $C_{11}H_{18}{}^{11}BO_4^+$ $[M-CH_3]^+$: 225.1298; Found: 225.1302.

Synthesis of 21a–22h through sulfoxide–magnesium exchange, Negishi cross-coupling

(*E*)-Ethyl 2-phenylcyclopropane-1-carboxylate (21a)

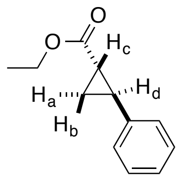

i-PrMgCl (0.32 mL, 1.85 M in THF, 0.60 mmol, 1.5 equiv) was added to a −78 °C solution of (*E*)-ethyl 2-(phenylsulfinyl)cyclopropanecarboxylate **6** (95 mg, 0.40 mmol, 1.0 equiv) in THF (4.0 mL) over 10 s and the solution stirred for 10 min. A mixture of $Pd_2(dba)_3$ (9 mg, 0.01 mmol, 2.5 mol%), $(t\text{-Bu})_3P$ (4.5 mg, 0.022 mmol, 5.5 mol%) and $ZnCl_2$ (82 mg, 0.60 mmol, 1.5 equiv) in THF (1.5 mL) was added and the solution stirred at 0 °C for 1 h. Bromobenzene (84 μL, 0.80 mmol, 2.0 equiv) was added and the reaction stirred at 25 °C for 15 h. MeOH (1 mL) was added and the mixture stirred for 15 min. The mixture was filtered through a pad of silica, washing with CH_2Cl_2 (200 mL). Concentration under reduced pressure followed by purification by flash column chromatography (30:1 *n*-hexane:EtOAc) gave cyclopropane **21a** (56 mg, 74%) as a colourless oil. R_f = 0.21 (19:1 *n*-hexane:EtOAc). IR (film)/cm^{-1} 3086 (CH), 3063 (CH), 3032 (CH), 2981 (CH), 2938 (CH), 2907 (CH), 1721 (C=O), 1178, 755, 698. ^{1}H NMR (400 MHz, $CDCl_3$) δ 7.31–7.28 (m, 2 H, 2 × Ph-H), 7.22 (tt, *J* = 7.4, 1.3 Hz, 1 H, Ph-H), 7.13–7.11 (m, 2 H, 2 × Ph-H), 4.19 (q, *J* = 7.1 Hz, 2 H, CH_2CH$_3$), 2.54 (ddd, *J* = 9.4, 6.5, 4.2 Hz, 1 H, H_c), 1.92 (ddd, *J* = 8.4, 5.2, 4.2 Hz, 1 H, H_d), 1.62 (ddd, *J* = 9.4, 5.2, 4.6 Hz, 1 H, H_b), 1.33 (ddd, *J* = 8.5, 6.5, 4.5 Hz, 1 H, H_a), 1.30 (t, *J* = 7.1 Hz, 3 H, CH_3). ^{13}C NMR (101 MHz, $CDCl_3$) δ 173.4 (C=O), 140.1 (Ph-C quat), 128.4 (2 × Ph-C), 126.4 (Ph-C), 126.1 (2 × Ph-C), 60.7 (CH_2CH_3), 26.2 (C(H_c)), 24.2 (C(H_d)), 17.1 (C(H_a)(H_b)), 14.3 (CH_3). HRMS (ES) *m/z* Calcd for $C_{12}H_{15}O_2^+$ [M+H]$^+$: 191.1072; Found: 191.1071. The observed data (^{1}H, ^{13}C) was consistent with that previously reported.[16]

(*Z*)-Ethyl 2-phenylcyclopropane-1-carboxylate (22a)

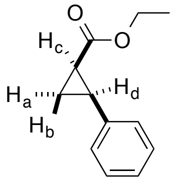

i-PrMgCl (0.32 mL, 1.85 M in THF, 0.60 mmol, 1.5 equiv) was added to a −78 °C solution of (*Z*)-ethyl 2-(phenylsulfinyl)cyclopropanecarboxylate **7** (95 mg, 0.40 mmol, 1.0 equiv) in THF (4.0 mL) over 10 s and the solution stirred for 10 min. A mixture of $Pd_2(dba)_3$ (9 mg, 0.01 mmol, 2.5 mol%), $(t\text{-Bu})_3P$ (4.5 mg, 0.022 mmol, 5.5 mol%) and $ZnCl_2$ (82 mg, 0.60 mmol, 1.5 equiv) in THF (1.5 mL) was added and the solution stirred at 0 °C for 1 h. Bromobenzene (84 μL, 0.80 mmol, 2.0 equiv) was added and the reaction stirred at 25 °C for 15 h. MeOH (1 mL) was added and the mixture stirred for 15 min. The mixture was filtered through a pad of silica, washing with CH_2Cl_2 (200 mL). Concentration under reduced pressure followed by purification by flash column chromatography (9:1 pentane:Et_2O) gave cyclopropane **22a** (58 mg, 76%) as a yellow oil. R_f = 0.27 (9:1 pentane:Et_2O). IR (film)/cm^{-1} 3028 (CH), 2982 (CH), 2934 (CH), 1726 (C=O), 1382, 1179, 1157. ^{1}H NMR (400 MHz, $CDCl_3$) δ 7.29–7.18 (m, 5 H, 5 × Ph-H), 3.88 (q, *J* = 7.1 Hz, 2 H, CH_2CH$_3$), 2.59 (ddd, *J* = 9.0, 9.0, 7.5 Hz, 1 H, H_c), 2.09 (ddd, *J* = 9.4, 7.8, 5.7 Hz, 1 H, H_d), 1.73 (ddd, *J* = 7.5, 5.3, 5.3 Hz, 1 H, H_b), 1.34 (ddd, *J* = 8.7, 7.8, 5.1 Hz, 1 H, H_a), 0.98 (t, *J* = 7.1 Hz, 3 H, CH_3). ^{13}C NMR (101 MHz, $CDCl_3$) δ 171.0 (C=O), 136.6 (Ph-C quat), 129.3 (2 × Ph-C), 127.9 (2 × Ph-C), 126.6 (Ph-C), 60.2 (CH_2CH_3), 25.5 (C(H_c)), 21.8 (C(H_d)), 14.0 (CH_3), 11.1 (C(H_a)(H_b)). HRMS (EI) *m/z* Calcd for $C_{12}H_{14}O_2^+$ [M]$^+$: 190.0994; Found: 190.0988. The observed data (^{1}H, ^{13}C) was consistent with that previously reported.[17]

(*E*)-Ethyl 2-(4-chlorophenyl)cyclopropane-1-carboxylate (21b)

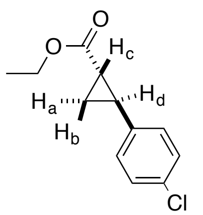

i-PrMgCl (0.32 mL, 1.85 M in THF, 0.60 mmol, 1.5 equiv) was added to a −78 °C solution of (*E*)-ethyl 2-(phenylsulfinyl)cyclopropanecarboxylate **6** (95 mg, 0.40 mmol, 1.0 equiv) in THF (4.0 mL) over 10 s and the solution stirred for 10 min. A mixture of $Pd_2(dba)_3$ (9 mg, 0.01 mmol, 2.5 mol%), $(t\text{-Bu})_3P$ (4.5 mg, 0.022 mmol, 5.5 mol%) and $ZnCl_2$ (82 mg, 0.60 mmol, 1.5 equiv) in THF (1.5 mL) was added and the solution stirred at 0 °C for 1 h. 1-Bromo, 4-chlorobenzene (153 mg, 0.80 mmol, 2.0 equiv) in THF (0.5 mL) was added and the reaction stirred at 25 °C for 15 h. MeOH (1 mL) was added and the mixture stirred for 15 min. The mixture was filtered through a pad of silica, washing with CH_2Cl_2 (200 mL). Concentration under reduced pressure followed by purification by flash chromatography (9:1 pentane:Et_2O) gave cyclopropane **21b** (76 mg, 85%) as a pale yellow oil. R_f = 0.31 (9:1 pentane:Et_2O). IR (film)/cm^{-1} 2983 (CH), 2938 (CH), 1724 (C=O), 1497, 1328, 1186. ^{1}H NMR (400 MHz, $CDCl_3$) δ 7.27–7.23 (m, 2 H, 2 × Ar-H), 7.05–7.02 (m, 2 H, 2 × Ar-H), 4.18 (q, *J* = 7.1 Hz, 2 H, CH_2CH$_3$), 2.50 (ddd, *J* = 9.3, 6.5, 4.2 Hz, 1 H, H_c), 1.87 (ddd, *J* = 8.5, 5.4, 4.2 Hz, 1 H, H_d), 1.64–1.58 (m, 1 H, H_b), 1.29 (t, *J* = 7.1 Hz, 3 H, CH_3), 1.28 (ddd, *J* = 8.5, 6.4, 4.6 Hz,

1 H, H_a). ^{13}C NMR (101 MHz, $CDCl_3$) δ 173.1 (C=O), 138.6 (Ar-C-Cl), 132.2 (Ar-C quat), 128.5 (2 × Ph-C), 127.6 (2 × Ph-C), 60.8 (CH_2CH_3), 25.5 (C(H_c)), 24.2 (C(H_d)), 17.0 (C(H_a)(H_b)), 14.2 (CH_3). HRMS (EI) *m/z* Calcd for $C_{12}H_{13}O_2Cl^+$ $[M]^+$: 224.0604; Found: 224.0608. The observed data (IR, ^1H, ^{13}C) was consistent with that previously reported.[18,19]

(*E*)-Ethyl 2-(4-methoxyphenyl)cyclopropane-1-carboxylate (21c)

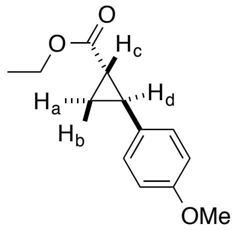

i-PrMgCl (0.32 mL, 1.85 M in THF, 0.60 mmol, 1.5 equiv) was added to a −78 °C solution of (*E*)-ethyl 2-(phenylsulfinyl)cyclopropanecarboxylate **6** (95 mg, 0.40 mmol, 1.0 equiv) in THF (4.0 mL) over 10 s and the solution stirred for 10 min. A mixture of $Pd_2(dba)_3$ (9 mg, 0.01 mmol, 2.5 mol%), $(t\text{-Bu})_3P$ (4.5 mg, 0.022 mmol, 5.5 mol%) and $ZnCl_2$ (82 mg, 0.60 mmol, 1.5 equiv) in THF (1.5 mL) was added and the solution stirred at 0 °C for 1 h. 4-Bromoanisole (100 μL, 0.80 mmol, 2.0 equiv) in THF (0.5 mL) was added and the reaction stirred at 25 °C for 15 h. MeOH (1 mL) was added and the mixture stirred for 15 min. The mixture was filtered through a pad of silica, washing with CH_2Cl_2 (200 mL). Concentration under reduced pressure followed by purification by flash column chromatography (15:1 pentane:Et_2O) gave cyclopropane **21c** (77 mg, 73%) as a cream crystalline solid. R_f = 0.24 (9:1 pentane:Et_2O). IR (film)/cm^{-1} 2988 (CH), 2953 (CH), 2911 (CH), 2837 (CH), 1719 (C=O), 1516, 1252, 1186. ^1H NMR (400 MHz, $CDCl_3$) δ 7.06–7.03 (m, 2 H, 2 × Ar-H), 6.85–6.81 (m, 2 H, 2 × Ar-H), 4.18 (q, *J* = 7.1 Hz, 2 H, CH_2CH_3), 3.79 (s, 3 H, OCH_3), 2.49 (ddd, *J* = 9.3, 6.5, 4.2 Hz, 1 H, H_c), 1.83 (ddd, *J* = 8.4, 5.2, 4.2 Hz, 1 H, H_d), 1.58–1.54 (m, 1 H, H_b), 1.29 (t, *J* = 7.1 Hz, 3 H, CH_3), 1.27–1.24 (m, 1 H, H_a). ^{13}C NMR (101 MHz, $CDCl_3$) δ 173.5 (C=O), 158.3 (Ph-C-OMe), 132.0 (Ph-C quat), 127.3 (2 × Ar-H), 113.9 (2 × Ar-H), 60.6 (CH_2CH_3), 55.3 (OCH_3), 25.6 (C(H_c)), 23.8 (C(H_d)), 16.7 (C(H_a)(H_b)), 14.3 (CH_3). FTMS (+p APCI) *m/z* Calcd for $C_{13}H_{17}O_3^+$ $[M+H]^+$: 221.1172; Found: 221.1182. The observed data (IR, ^1H, ^{13}C) was consistent with that previously reported.[18,20]

(*E*)-Ethyl 2-[(*E*)-2-phenylethenyl]cyclopropane-1-carboxylate (21d)

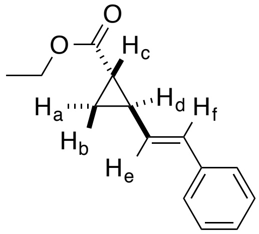

i-PrMgCl (0.32 mL, 1.85 M in THF, 0.60 mmol, 1.5 equiv) was added to a −78 °C solution of (*E*)-ethyl 2-(phenylsulfinyl)cyclopropanecarboxylate **6** (95 mg, 0.40 mmol, 1.0 equiv) in THF (4.0 mL) over 10 s and the solution stirred for 10 min. A mixture of $Pd_2(dba)_3$ (9 mg, 0.01 mmol, 2.5 mol%), $(t\text{-Bu})_3P$ (4.5 mg, 0.022 mmol, 5.5 mol%) and $ZnCl_2$ (82 mg, 0.60 mmol, 1.5 equiv) in THF (1.5 mL) was added and the solution stirred at 0 °C for 1 h. β-Bromostyrene (103 μL, 0.80 mmol, 2.0 equiv) was added and the reaction stirred at 25 °C for 15 h. MeOH (1 mL) was added and the mixture stirred for 15 min. The mixture was filtered through a pad of silica, washing with CH_2Cl_2 (200 mL). Concentration under reduced pressure followed by purification by flash column chromatography (19:1 pentane:Et_2O) gave cyclopropane **21d** (73 mg, 84%) as a golden oil. R_f = 0.46 (9:1 pentane:Et_2O). IR (film)/cm^{-1} 3027 (CH), 3000 (CH), 2983 (CH), 2911 (CH), 1718 (C=O), 1650 (C=C), 1341, 1175, 1036, 964, 754. ^1H NMR (400 MHz, $CDCl_3$) δ 7.33–7.28 (m, 4 H, 4 × Ph-H), 7.25–7.20 (m, 1 H, Ph-H), 6.55 (d, *J* = 15.8 Hz, 1 H, H_f), 5.77 (dd, *J* = 15.8, 8.7 Hz, 1 H, H_e), 4.17 (q, *J* = 7.1 Hz, 2 H, CH_2CH_3), 2.19 (dddd, *J* = 8.9, 8.7, 6.3, 3.9 Hz, 1 H, H_c), 1.77 (ddd, *J* = 8.4, 5.2, 3.9 Hz, 1 H, H_d), 1.49 (ddd, *J* = 8.9, 5.2, 4.5 Hz, 1 H, H_b), 1.29 (t, *J* = 7.1 Hz, 3 H, CH_3), 1.11 (ddd, *J* = 8.4, 6.2, 4.4 Hz, 1 H, H_a). ^{13}C NMR (101 MHz, $CDCl_3$) δ 173.3 (C=O), 137.0 (Ph-C quat), 130.3 (alkene CH), 130.1 (alkene CH), 128.5 (2 × Ph-C), 127.2 (Ph-C), 125.8 (2 × Ph-C), 60.7 (CH_2CH_3), 25.5 (C(H_c)), 22.3 (C(H_d)), 16.0 (C(H_a)(H_b)), 14.3 (CH_3). The observed data (^1H) was consistent with that previously reported.[21]

(*E*)-Ethyl 2-(pyridin-2-yl)cyclopropane-1-carboxylate (21e)

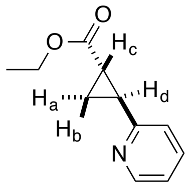

i-PrMgCl (0.32 mL, 1.85 M in THF, 0.60 mmol, 1.5 equiv) was added to a −78 °C solution of (*E*)-ethyl 2-(phenylsulfinyl)cyclopropanecarboxylate **6** (95 mg, 0.40 mmol, 1.0 equiv) in THF (4.0 mL) over 10 s and the solution stirred for 10 min. A mixture of $Pd_2(dba)_3$ (9 mg, 0.01 mmol, 2.5 mol%), $(t\text{-Bu})_3P$ (4.5 mg, 0.022 mmol, 5.5 mol%) and $ZnCl_2$ (82 mg, 0.60 mmol, 1.5 equiv) in THF (1.5 mL) was added and the solution stirred at 0 °C for 1 h. 2-Bromopyridine (76 µL, 0.80 mmol, 2.0 equiv) was added and the reaction stirred at 25 °C for 15 h. MeOH (1 mL) was added and the mixture stirred for 15 min. The mixture was filtered through a pad

of silica, washing with CH_2Cl_2 (200 mL). Concentration under reduced pressure followed by flash column chromatography (4:1 pentane:Et_2O) gave cyclopropane **21e** (46 mg, 60%) as a yellow oil. R_f = 0.17 (4:1 pentane:Et_2O). IR (film)/cm^{-1} 2981 (CH), 2930 (CH), 1723 (C=O), 1595, 1476, 1330, 1179, 1049, 775. ^{1}H NMR (400 MHz, $CDCl_3$) δ 8.46–8.44 (ddd, *J* = 4.9, 1.8, 1.0 Hz, 1 H, Ar-H), 7.59–7.56 (ddd, *J* = 7.6, 7.6, 1.8 Hz, 1 H, Ar-H), 7.25–7.22 (ddd, *J* = 7.6, 1.0, 1.0 Hz, 1 H, Ar-H), 7.11–7.07 (ddd, *J* = 7.6, 4.9, 1.0 Hz, 1 H, Ar-H), 4.17 (q, *J* = 7.2 Hz, 2 H, C*H*$_2$CH$_3$), 2.59 (ddd, *J* = 9.0, 6.1, 3.9 Hz, 1 H, H_c), 2.25 (ddd, *J* = 8.3, 5.6, 3.9 Hz, 1 H, H_d), 1.65–1.57 (m, 2 H, H_a + H_b), 1.28 (t, *J* = 7.2 Hz, 3 H, CH_3). ^{13}C NMR (101 MHz, $CDCl_3$) δ 173.4 (C=O), 158.9 (Ar-C quat), 149.4 (Ar-C), 136.0 (Ar-C), 122.5 (Ar-C), 121.3 (Ar-C), 60.7 (*C*H$_2$CH$_3$), 27.2 (C(H_c)), 24.3 (C(H_d)), 17.3 (C(H_a)(H_b)), 14.2 (CH_3). HRMS (EI) *m/z* Calcd for $C_{11}H_{13}NO_2^+$ [M]$^+$: 191.0946; Found: 191.0941. The observed data (^{1}H, ^{13}C) was consistent with that previously reported.[19]

(*E*)-Ethyl 2-(pyrimidin-2-yl)cyclopropane-1-carboxylate (21f)

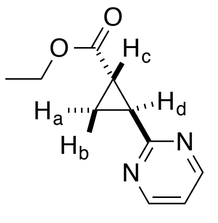

i-PrMgCl (0.32 mL, 1.85 M in THF, 0.60 mmol, 1.5 equiv) was added to a –78 °C solution of (*E*)-ethyl 2-(phenylsulfinyl)cyclopropanecarboxylate **6** (95 mg, 0.40 mmol, 1.0 equiv) in THF (4.0 mL) over 10 s and the solution stirred for 10 min. A mixture of $Pd_2(dba)_3$ (9 mg, 0.01 mmol, 2.5 mol%), (*t*-Bu)$_3$P (4.5 mg, 0.022 mmol, 5.5 mol%) and $ZnCl_2$ (82 mg, 0.60 mmol, 1.5 equiv) in THF (1.5 mL) was added and the solution stirred at 0 °C for 1 h. 2-Bromopyrimidine (127 mg, 0.80 mmol, 2.0 equiv) in toluene (2.0 mL) was added and the reaction stirred at 25 °C for 15 h. MeOH (1 mL) was added and the mixture stirred for 15 min. The mixture was filtered through a pad of silica, washing with CH_2Cl_2 (200 mL). Concentration under reduced pressure followed by flash column chromatography (4:1 pentane:Et_2O) gave cyclopropane **21f** (45 mg, 59%) as a yellow oil. R_f = 0.28 (1:2 pentane:Et_2O). IR (film)/cm^{-1} 2982 (CH), 2938 (CH), 1725 (C=O), 1561, 1425, 1331, 1180. ^{1}H NMR (400 MHz, $CDCl_3$) δ 8.60 (d, *J* = 4.9 Hz, 2 H, 2 × ArNCH), 7.11 (t, *J* = 4.9 Hz, 1 H, ArNCHC*H*), 4.17 (q, *J* = 7.2 Hz, 2 H, C*H*$_2$CH$_3$), 2.82 (ddd, *J* = 8.7, 6.2, 3.9 Hz, 1 H, H_c), 2.31 (ddd, *J* = 8.5, 5.7, 3.8 Hz, 1 H, H_d), 1.71–1.64 (m, 2 H, H_a + H_b), 1.28 (t, *J* = 7.1 Hz, 3 H, CH_3). ^{13}C NMR (101 MHz, $CDCl_3$) δ 172.5 (C=O), 168.8 (Ar-C quat), 156.9 (2 × Ar-C), 118.6 (Ar-C), 60.8 (*C*H$_2$CH$_3$), 28.0 (C(H_c)), 25.0 (C(H_d)), 17.7 (C(H_a)(H_b)), 14.2 (CH_3). HRMS (EI) *m/z* Calcd for $C_{10}H_{12}N_2O_2^+$ [M]$^+$: 192.0899; Found: 192.0908.

(*E*)-*tert*-Butyl 3-[2-(ethoxycarbonyl)cyclopropyl]-1*H*-indole-1-carboxylate (21g)

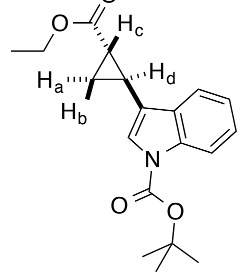

i-PrMgCl (0.32 mL, 1.85 M in THF, 0.60 mmol, 1.5 equiv) was added to a –78 °C solution of (*E*)-ethyl 2-(phenylsulfinyl)cyclopropanecarboxylate **6** (95 mg, 0.40 mmol, 1.0 equiv) in THF (4.0 mL) over 10 s and the solution stirred for 10 min. A mixture of $Pd_2(dba)_3$ (9 mg, 0.01 mmol, 2.5 mol%), (*t*-Bu)$_3$P (4.5 mg, 0.022 mmol, 5.5 mol%) and $ZnCl_2$ (82 mg, 0.60 mmol, 1.5 equiv) in THF (1.5 mL) was added and the solution stirred at 0 °C for 1 h. A solution of 3-Bromoindole-1-carboxylic acid tert-butyl ester (237 mg, 0.80 mmol, 2.0 equiv) in toluene (1.0 mL) was added and the reaction stirred at 25 °C for 15 h. MeOH (1 mL) was added and the mixture stirred for 15 min. The mixture was filtered through a pad of silica, washing with CH_2Cl_2 (200 mL). Concentration under reduced pressure followed by purification by flash column chromatography (19:1 pentane:Et_2O) gave cyclopropane **21g** (119 mg, 94%) as a yellow gum. R_f = 0.15 (19:1 pentane:Et_2O). IR (film)/cm^{-1} 2980 (CH), 2934 (CH), 1722 (C=O), 1451, 1369, 1345, 1254, 1152, 1079, 743. ^{1}H NMR (400 MHz, $CDCl_3$) δ 8.12 (d, *J* = 6.7 Hz, 1 H, ArNCH), 7.62 (d, *J* = 7.6 Hz, 1 H, Ar-H), 7.36–7.25 (m, 3 H, 3 × Ar-H), 4.23 (q, *J* = 7.1 Hz, 2 H, C*H*$_2$CH$_3$), 2.55 (dddd, *J* = 9.0, 6.5, 4.2, 1.1 Hz, 1 H, H_c), 1.92 (ddd, *J* = 8.4, 5.1, 4.4 Hz, 1 H, H_d), 1.68 (s, 9 H, C(CH_3)$_3$), 1.61 (ddd, *J* = 9.1, 5.0, 4.3 Hz, 1 H, H_b), 1.34–1.29 (m, 4 H, H_a + CH$_2$C*H*$_3$). ^{13}C NMR (101 MHz, $CDCl_3$) δ 173.6 (C=O ester), 149.6 (C=O carbamate), 135.4 (Ar-C quat), 130.4 (Ar-C quat), 124.6 (Ar-C), 122.6 (Ar-C), 122.0 (Ar-C), 120.3 (Ar-C quat), 119.0 (Ar-C), 115.3 (Ar-C), 83.7 (*C*(CH$_3$)$_3$), 60.7 (*C*H$_2$CH$_3$), 28.2 (C(*C*H$_3$)$_3$), 21.7 (C(H_d)), 17.1 (C(H_c)), 15.3 (C(H_a)(H_b)), 14.3 (CH$_2$*C*H$_3$). HRMS (EI) *m/z* Calcd for $C_{19}H_{23}NO_4^+$ [M]$^+$: 329.1627; Found: 329.1635. The observed data (^{1}H, ^{13}C) was consistent with that previously reported.[19]

(*Z*)-Ethyl 2-[1-(benzenesulfonyl)-1*H*-indol-3-yl]cyclopropane-1-carboxylate (22h)

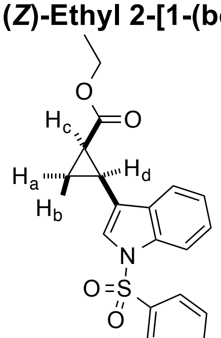

i-PrMgCl (1.20 mL, 1.85 M in THF, 2.25 mmol, 1.5 equiv) was added to a −78 °C solution of (*Z*)-ethyl 2-(phenylsulfinyl)cyclopropanecarboxylate **7** (358 mg, 1.5 mmol, 1.0 equiv) in THF (15 mL) over 10 s and the solution stirred for 10 min. A mixture of $Pd_2(dba)_3$ (35 mg, 0.04 mmol, 2.5 mol%), $(t\text{-Bu})_3P$ (17 mg, 0.08 mmol, 5.5 mol%) and $ZnCl_2$ (308 mg, 2.25 mmol, 1.5 equiv) in THF (1.9 mL) was added and the solution stirred at 0 °C for 1 h. A solution of 3-bromo-(1-phenylsulfonyl)indole (1.00 g, 3.00 mmol, 2.0 equiv) in THF (2.0 mL) was added and the reaction stirred at 25 °C for 15 h. MeOH (5 mL) was added and the mixture stirred for 15 min. The mixture was filtered through a pad of silica, washing with CH_2Cl_2 (200 mL). Concentration under reduced pressure followed by purification by flash column chromatography (5:1 pentane:EtOAc) gave cyclopropane **22h** (414 mg, 75%) as a colourless gum. R_f = 0.24 (5:1 pentane:EtOAc). IR (film)/cm^{-1} 3065 (CH), 2931 (CH), 1721 (C=O), 1447, 1366 (S=O), 1170 (S=O), 1124, 1092, 980, 734, 684. ^{1}H NMR (400 MHz, $CDCl_3$) δ 7.92 (d, *J* = 8.2 Hz, 1 H, Ar-H), 7.87–7.84 (m, 2 H, 2 × Ar-H), 7.59–7.57 (m, 1 H, Ar-H), 7.52–7.48 (m, 1 H, Ar-H), 7.44–7.40 (m, 3 H, 3 × Ar-H), 7.30–7.26 (ddd, *J* = 7.4, 7.4, 1.3 Hz, 1 H, Ar-H), 7.24–7.20 (ddd, *J* = 7.4, 7.4, 1.0 Hz, 1 H, Ar-H), 3.78–3.63 (m, 2 H, CH_2CH_3), 2.43 (dddd, *J* = 8.6, 8.6, 8.6, 1.2 Hz, 1 H, H_c), 2.18 (ddd, *J* = 8.9, 7.9, 5.7 Hz, 1 H, H_d), 1.65 (ddd, *J* = 7.2, 5.6, 5.1 Hz, 1 H, H_b), 1.42 (ddd, *J* = 8.6, 8.1, 4.9 Hz, 1 H, H_a), 0.73 (t, *J* = 7.1 Hz, 3 H, CH_3). ^{13}C NMR (101 MHz, $CDCl_3$) δ 170.4 (C=O), 138.1 (Ar-C quat), 135.0 (Ar-C quat), 133.5 (Ar-C), 131.5 (Ar-C quat), 129.1 (2 × Ar-C), 126.8 (2 × Ar-C), 125.0 (Ar-C), 124.7 (Ar-C), 123.1 (Ar-C), 119.4 (Ar-C), 118.9 (Ar-C quat), 113.6 (Ar-C), 60.1 (CH_2CH_3), 20.7 ($C(H_d)$), 15.5 ($C(H_c)$), 13.7 (CH_3), 11.1 ($C(H_a)(H_b)$). HRMS (ES) *m/z* Calcd for $C_{20}H_{20}NO_4S^+$ $[M+H]^+$: 370.1113; Found: 370.1114. The observed data (^{1}H, ^{13}C) was consistent with that previously reported.[22]

Synthesis of 23, 24 and 25

(*E*)-Sodium 2-(pyridin-2-yl)cyclopropane-1-carboxylate (23)

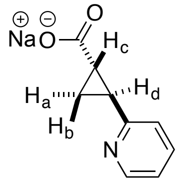

$NaOH_{(aq)}$ (1.0 M, 0.29 mL, 0.29 mmol, 1.2 equiv) was added to a 30 °C solution of (*E*)-ethyl 2-(pyridin-2-yl)cyclopropane-1-carboxylate **21e** (46 mg, 0.24 mmol, 1.0 equiv) in EtOH (1.2 mL) and the solution was stirred at 30 °C for 24 h. The red reaction mixture was concentrated under reduced pressure, then filtered, washing with acetone (50 mL). The filtrate was concentrated under reduced pressure to give cyclopropane **23** (44 mg, quant) as a red gum. IR (film)/cm^{-1} 1644, 1560 (C=O), 1420, 1371. ^{1}H NMR (400 MHz, DMSO-d_6) δ 8.36 (d, *J* = 4.1 Hz, 1 H, Ar-H), 7.59 (ddd, *J* = 7.7, 7.7, 1.6 Hz, 1 H, Ar-H), 7.21 (d, *J* = 7.8 Hz, 1 H, Ar-H), 7.08 (dd, *J* = 7.0, 5.2 Hz, 1 H, Ar-H), 2.25 (ddd, *J* = 8.8, 5.2, 5.2 Hz, 1 H, H_c), 1.70 (ddd, *J* = 8.4, 5.5, 4.1 Hz, 1 H, H_d), 1.21 (ddd, *J* = 8.0, 5.5, 2.4 Hz, 1 H, H_a), 1.16–1.07 (m, 1 H, H_b). ^{13}C NMR (101 MHz, DMSO-d_6) δ 176.1 (C=O), 161.7 (Ar-C quat), 148.9 (Ar-C), 135.9 (Ar-C), 121.4 (Ar-C), 120.4 (Ar-C), 29.2 ($C(H_d)$), 25.2 ($C(H_c)$), 16.2 ($C(H_a)(H_b)$). HRMS (ES) *m/z* Calcd for $C_9H_{10}NO_2^+$ $[M-Na+2H]^+$: 164.0712; Found: 164.0718.

(*E*)-[2-(Pyridin-2-yl)cyclopropyl](pyrrolidin-1-yl)methanone (24)

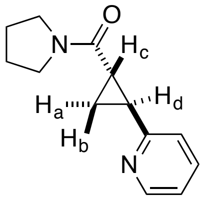

HATU (49 mg, 0.13 mmol, 1.2 equiv) was added to a solution of (*E*)-sodium 2-(pyridin-2-yl)cyclopropane-1-carboxylate **23** (20 mg, 0.11 mmol, 1.0 equiv) in *N*,*N*-dimethylformamide (540 μL) and the resultant red solution was stirred at 40 °C for 10 min. Pyrrolidine (11 μL, 0.13 mmol, 1.2 equiv) was added and the solution stirred for 20 min. Diisopropylethylamine (56 μL, 0.32 mmol, 3.0 equiv) was added and the solution stirred for 24 h. H_2O (10 mL) was added and the mixture extracted with EtOAc (5 × 10 mL). The combined organic phases were washed with brine (10 mL), dried ($MgSO_4$), filtered and concentrated under reduced pressure. Purification by flash column chromatography (1:2 pentane:Et_2O) gave cyclopropane **24** (19 mg, 82%) as a yellow gum. R_f = 0.27 (1:2 pentane:Et_2O). IR (film)/cm^{-} 2954 (CH), 2923 (CH), 2854 (CH), 1741 (C=O), 1596, 1460, 1375, 1220, 964, 803. ^{1}H NMR (400 MHz, $CDCl_3$) δ 8.45 (d, *J* = 4.4 Hz, 1 H, Ar-H), 7.56 (ddd, *J* = 7.7, 7.7, 1.5 Hz, 1 H, Ar-H), 7.29 (d, *J* = 8.1 Hz, 1 H, Ar-H), 7.09–7.06 (m, 1 H, Ar-H), 2.58 (ddd, *J* = 9.1, 5.5, 4.4 Hz, 1 H, H_c), 2.41 (ddd, *J* = 9.0, 5.7, 4.1 Hz, 1 H, H_d), 1.58 (ddd, *J* = 8.8, 5.5, 3.3 Hz, 1 H, H_a), 1.48 (ddd, *J* = 8.9, 5.7, 3.4 Hz, 1 H, H_b), 1.34–1.22 (m, 8 H, 4 × CH_2). ^{13}C NMR (101 MHz, $CDCl_3$) δ 160.2

(C=O), 149.3 (Ar-C), 135.9 (Ar-C), 125.5 (Ar-C quat), 122.9 (Ar-C), 120.9 (Ar-C), 45.6 (NCH_2), 29.7 (NCH_2), 25.8 (C(H_c)), 25.3 (C(H_d)), 21.6 (NCH_2CH_2), 20.7 (NCH_2CH_2), 17.2 (C(H_a)(H_b)). FTMS (+p NSI) *m/z* Calcd for $C_{13}H_{17}N_2O^+$ $[M+H]^+$: 217.1335; Found: 217.1336.

(*Z*)-{2-[1-(Benzenesulfonyl)-1*H*-indol-3-yl]cyclopropyl}methanol (25)

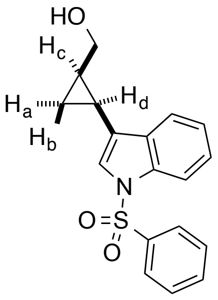

Lithium aluminum tetrahydride (1.0 M in THF, 1.35 mL, 1.35 mmol, 2.5 equiv) was added dropwise over 5 min to a 0 °C solution of (*Z*)-ethyl 2-[1-(benzenesulfonyl)-1*H*-indol-3-yl]cyclopropane-1-carboxylate (**22h**) (200 mg, 0.54 mmol, 1.0 equiv) in THF (0.68 mL) and the solution stirred at 0 °C for 10 min. The solution was warmed to 25 °C and stirred for 3 h. The reaction mixture was cooled to 0 °C, EtOAc (7 ml) was added and the mixture stirred for 15 min. The mixture was warmed to 25 °C, sat. aq. potassium sodium tartrate (15 mL) was added and the mixture stirred for 1 h. The organic phase was separated, and the aqueous phase extracted with Et_2O (5 × 10 mL). The combined organic phases were washed with brine (20 mL), dried ($MgSO_4$), filtered and concentrated under reduced pressure. Purification by flash column chromatography (1:3 pentane:Et_2O) gave cyclopropane **25** (166 mg, 94%) as a yellow gum. R_f (Et_2O) = 0.44. IR (film)/cm^{-1} 3387 (OH), 3117 (CH), 3067 (CH), 2923 (CH), 1447, 1366 (S=O), 1173 (S=O), 1124, 1096, 739, 685. ^1H NMR (400 MHz, $CDCl_3$) δ 8.00 (d, *J* = 8.2 Hz, 1 H, Ar-H), 7.86–7.84 (m, 2 H, 2 × Ar-H), 7.65 (d, *J* = 7.6 Hz, 1 H, Ar-H), 7.54 (t, *J* = 7.4 Hz, 1 H, Ar-H), 7.44 (t, *J* = 7.7 Hz, 2 H, 2 × Ar-H), 7.37–7.33 (m, 1 H, Ar-H), 7.30–7.29 (m, 2 H, Ar-H), 3.51–3.48 (m, 1 H, C*H*(H)OH), 3.12–3.07 (m, 1 H, CH(*H*)OH), 2.12–2.06 (m, 1 H, H_d), 1.64–1.55 (m, 1 H, H_c), 1.15 (ddd, *J* = 8.3, 8.3, 5.1 Hz, 1 H, H_a), 0.88 (br s, 1 H, OH), 0.75 (ddd, *J* = 5.5, 5.5, 5.5 Hz, 1 H, H_b). ^{13}C NMR (101 MHz, $CDCl_3$) δ 138.0 (Ar-C quat), 135.5 (Ar-C quat), 133.8 (Ar-C), 131.7 (Ar-C quat), 129.2 (2 × Ar-C), 126.6 (2 × Ar-C), 125.2 (Ar-C), 124.0 (Ar-C), 123.6 (Ar-C), 121.1 (Ar-C quat), 119.4 (Ar-C), 113.9 (Ar-C), 62.7 (CH_2OH), 19.9 (C(H_c)), 10.8 (C(H_d)), 7.6 (C(H_a)(H_b)). HRMS (ES) *m/z* Calcd for $C_{18}H_{18}NO_3S^+$ $[M+H]^+$: 328.1007; Found: 328.1007.

Optimization of an enantioselective cyclopropanation of phenyl vinyl sulfide

Enantiopure ligands were applied to the cyclopropanation of phenyl vinyl sulfide and ethyl diazoacetate towards an asymmetric cyclopropanation. A range of BOX and PyBOX ligands were investigated in the CuOTf-catalyzed protocol, that give high yields and ee on styrene substrates.[23] However, all gave low yields, dr and ee with PVS. Following the success of the Co-catalyzed reaction, several enantiopure Co^{II}(salen)-type complexes were prepared (see below) to probe a variety of steric and electronic effects (Table **S3**). The best results were achieved using commercial complex **3** (Table **S3**, entry 1), which gave a quantitative yield and showed moderate and good enantioselectivity for the *trans*- and *cis*-cyclopropane products, respectively. Other variations in catalyst structure were not advantageous.

Table S3. Asymmetric induction using Co^{II}(salen)-type complexes

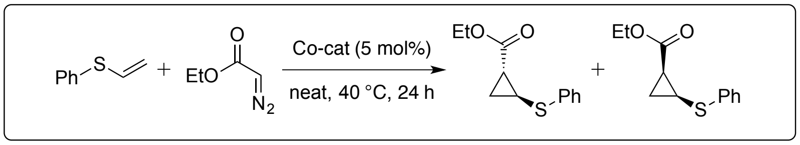

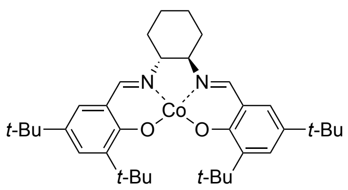

3

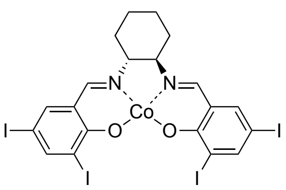

26

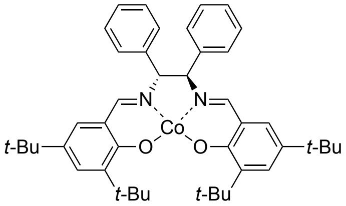

27

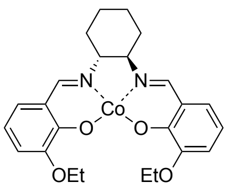

28

Entry	Complex	dr[a] *trans*:*cis*	ee *trans* (%)	ee *cis* (%)	Yield[b] *trans* + *cis* (%)
1	**3**	47:53	40	64	100
2	**26**	50:50	29	23	9
3	**27**	44:56	32	42	6
4	**28**	49:51	51	57	17

[a] Calculated from the crude reaction mixture by ^{1}H NMR. [b] Yields were calculated using ^{1}H NMR by comparison with an internal standard (dibenzyl ether).

A brief optimization of the solvent, reaction temperature and examination of additives, was undertaken (Table **S4**). Conducting the reaction in cyclohexane at 40 °C (Table **S4**, entry 7) or on H_2O at 20 °C (Table **S4**, entry 9) gave the highest levels of ee observed.

Table S4. Asymmetric induction optimization using Co^{II}(salen)-type complex **3**

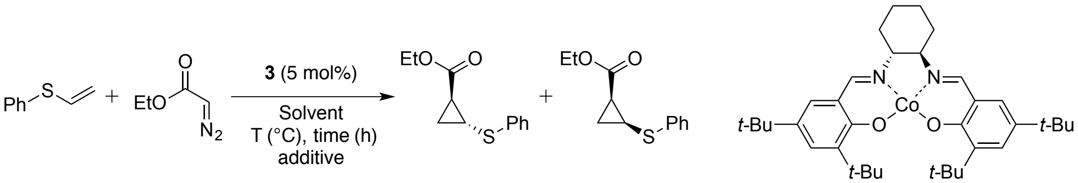

Entry	Additive (5 mol%)	Solvent	Temperature (°C)	dr[a] *trans*:*cis*	ee *trans*	ee *cis*	Yield[b] *trans* + *cis* (%)
1	None	H_2O	40	47:53	40	64	100
2	KSAc	H_2O	40	48:52	48	63	100
3	DMSO	H_2O	40	48:52	46	66	100
4	Br_2	H_2O	40	-	-	-	0
5	None	Benzene	40	48:52	44	60	73
6	None	Toluene	40	45:55	47	63	53
7	None	TBME	40	48:52	49	66	69
7	None	Cyclohexane	40	45:55	53	73	55
8	None	H_2O	30	47:53	48	65	92
9	None	H_2O	20	45:55	52	77	57

[a] Calculated from the crude reaction mixture by ^{1}H NMR. [b] Yields were calculated using ^{1}H NMR by comparison with an internal standard (dibenzyl ether).

Synthesis of enantiopure C_2-symmetric Co^{II}-(salen)-type complexes

General procedure for tetradentate Schiff base synthesis

A solution of the enantiopure chiral diamine (1.0 equiv) in EtOH (0.20 M) was added to a solution of the salicylaldehyde derivative (2.0 equiv) in EtOH (0.17 M) and the solution refluxed for 3–20 h. Filtration and recrystallization from ethanol gave the desired tetradentate Schiff bases. The observed data was consistent with that previously reported for the tetradentate Schiff base ligands for complexes **26**,[24] **27**,[25] and **28**.[26]

***N,N'*-Bis[(*E*)-(3,5-di-iodo)-2-hydroxyphenylmethylene]-[(1*R*,2*R*)-1,2-cyclohexane diamino]cobalt(II) (26)**

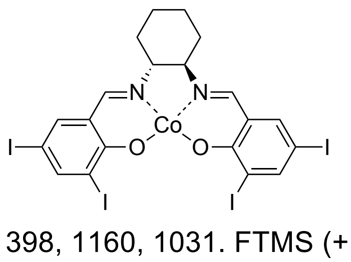

A mixture of $Co^{II}(OAc)_2$ (33 mg, 0.18 mmol, 1.0 equiv), H_2O (1.0 mL) and ethanol (2.5 mL) was added to a solution of *N,N'*-bis[(*E*)-(3,5-di-iodo)-2-hydroxyphenylmethylene]-(1*R*,2*R*)-1,2-cyclohexanediamine (148 mg, 0.18 mmol, 1.0 equiv) in toluene (1.5 mL).The reaction mixture was refluxed for 3 h, cooled to rt, concentrated under reduced pressure, then recrystallized in 3:2 ethanol:$CHCl_3$ (v/v) to give **26** (151 mg, 95%) as a vivid orange solid. mp > 250 °C. IR (film)/cm^{-1} 3003 (CH), 2938 (CH), 2858 (CH), 1738, 1599, 1568, 1490, 1422, 1398, 1160, 1031. FTMS (+ p NSI) *m/z* Calcd for $C_{20}H_{16}CoN_2O_2I_4^+$ $[M]^+$: 882.6717; Found: 882.6714.

***N,N'*-Bis[(*E*)-(3,5-di-*tert*-butyl)-2-hydroxyphenylmethylene]-[(1*R*,2*R*)-1,2-diphenylethylenediamino]cobalt(II) (27)**

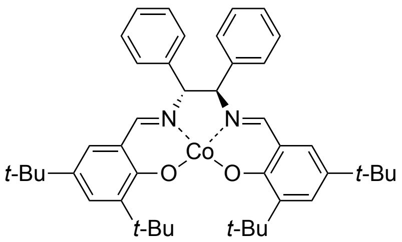

A mixture of $Co(OAc)_2$ (123 mg, 0.70 mmol, 1.0 equiv), H_2O (2.0 mL) and ethanol (4.0 mL) was added to a solution of *N*,*N'*-bis[(*E*)-(3,5-di-*tert*-butyl)-2-hydroxyphenylmethylene]-(1*R*,2*R*)-1,2-diphenylethylenediamine (450 mg, 0.70 mmol, 1.0 equiv) in toluene (4.0 mL, 0.18 M). The reaction mixture was refluxed for 3 h, cooled to rt, concentrated under reduced pressure, then recrystallized from ethanol to obtain **27** (327 mg, 67%) as a vivid red solid. mp > 250 °C. IR (film)/cm^{-1} 2951 (CH), 2904 (CH), 2867 (CH), 1589 (C=N), 1525, 1454, 1319, 1250, 1179, 787, 698. FTMS (+ p NSI) *m/z* Calcd for $C_{44}H_{54}CoN_2O_2^+$ $[M]^+$: 701.3512; Found: 701.3505. The observed data was consistent with that previously reported.[27]

***N,N'*-Bis[(*E*)-3-ethoxy-2-hydroxybenzylidene]-[(1*R*,2*R*)-1,2-cyclohexanediamino] cobalt(II) (28)**

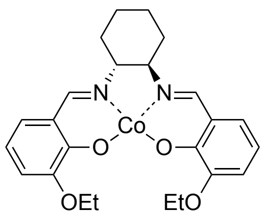

A mixture of $Co^{II}(OAc)_2$ (33 mg, 0.18 mmol), H_2O (1.0 mL) and ethanol (2.5 mL) was added to a solution of *N,N'*-bis[(*E*)-3-ethoxy-2-hydroxybenzylidene]-(1*R*,2*R*)-1,2-cyclohexanediamine (74 mg, 0.18 mmol) in toluene (1.5 mL, 0.12 M). The reaction mixture was refluxed for 3 h, cooled to rt, concentrated under reduced pressure, then recrystallized from $CHCl_3$ to give **28** (74 mg, quant) as a brown solid. mp > 250 °C. IR (film)/cm^{-1} 3250 (CH), 2980 (CH), 2935 (CH), 2864 (CH), 1635 (C=N), 1603, 1561, 1469, 1447, 1390, 1247, 1222. FTMS (+ p NSI) *m/z* Calcd for $C_{20}H_{20}CoN_2O_4^+$ $[M]^+$: 411.0750; Found: 411.0750.

HPLC and SFC traces and conditions

Racemic and enantioselective cyclopropanation

Enantioenriched cyclopropanes **1** and **2** were obtained from an asymmetric cyclopropanation (reaction conditions: Table S1, entry 21). The ee was calculated using chiral HPLC, and compared to the corresponding racemic material. HPLC traces were obtained using apparatus consisting of JASCO AS-1555 Intelligent Sampler, 2 × JASCO PU-1580 HPLC Pump, JASCO HG-1580-32 Dynamic Mixer, JASCO MD-1510 Multiwavelength Detector and JASCO LC-Net II/ADC.

(±)-*E*-Ethyl 2-(phenylsulfanyl)-cyclopropane-1-carboxylate

HPLC Conditions: Chiralpak IB-3 column, 100% *n*-hexane, flow rate: 1.0 mL min^{-1}, 25 °C, UV detection wavelength: 220 nm. Retention times: 19.4 min (1*R*,2*S* enantiomer), 25.0 min (1*S*,2*R* enantiomer).

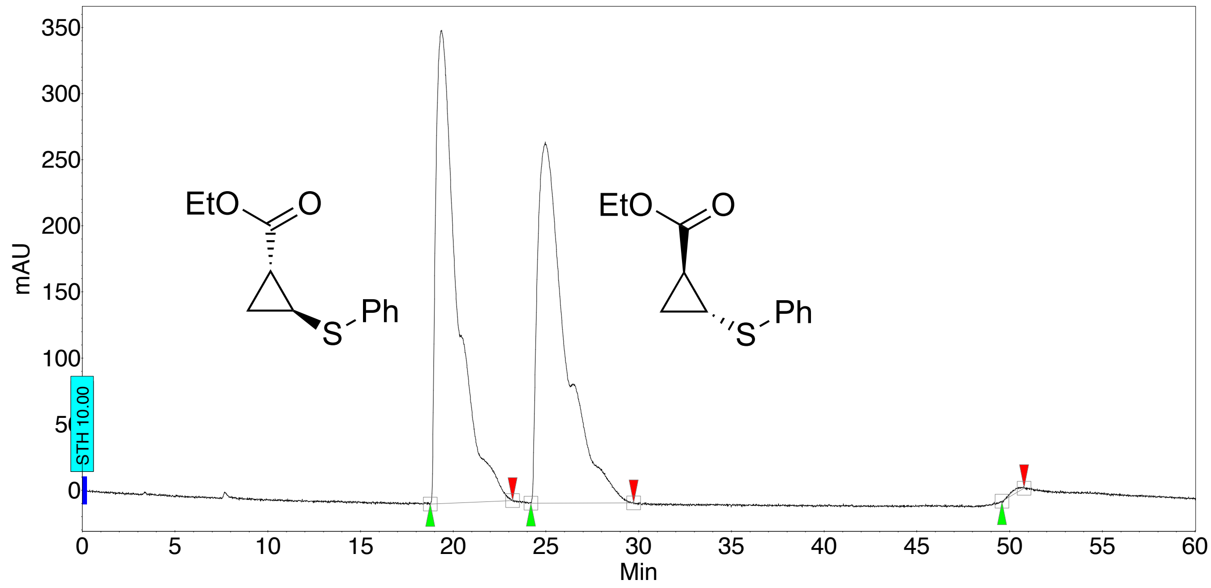

Peak results :

Index	Name	Time [Min]	Quantity [% Area]	Height [mAU]	Area [mAU.Min]	Area % [%]
1	UNKNOWN	19.372	50.21	357.5	482.9	50.214
2	UNKNOWN	24.972	49.57	270.9	476.7	49.569
3	UNKNOWN	50.303	0.22	3.0	2.1	0.217
Total			100.00	631.4	961.6	100.000

Enantioenriched *E*-ethyl 2-(phenylsulfanyl)-cyclopropane-1-carboxylate

HPLC Conditions: Chiralpak IB-3 column, 100% *n*-hexane, flow rate: 1.0 mL min^{-1}, 25 ºC, UV detection wavelength: 220 nm. Retention times: 22.2 min (1*R*,2*S* enantiomer), 27.9 min (1*S*,2*R* enantiomer).

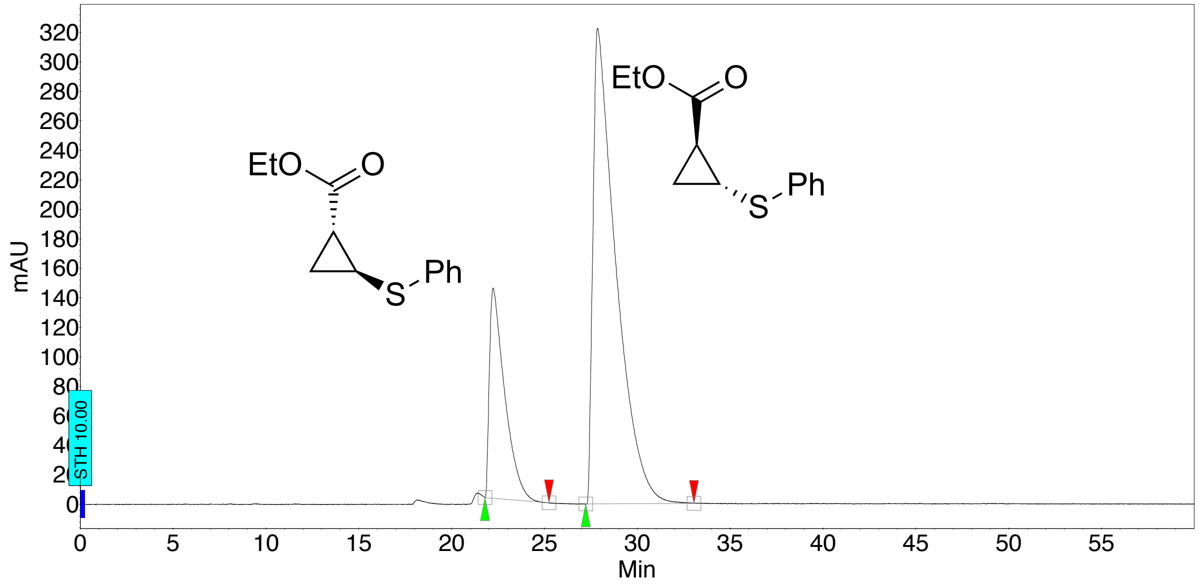

Peak results :

Index	Name	Time [Min]	Quantity [% Area]	Height [mAU]	Area [mAU.Min]	Area % [%]
1	UNKNOWN	22.225	22.77	142.6	136.3	22.770
2	UNKNOWN	27.851	77.23	322.6	462.2	77.230
Total			100.00	465.2	598.4	100.000

ee = <u>54.5%</u>

(±)-*Z*-Ethyl 2-(benzenesulfonyl)cyclopropane-1-carboxylate ((±)-*Z*-Ethyl 2-(phenylsulfanyl)-cyclopropane-1-carboxylate was oxidized to the corresponding sulfone to facilitate separation of the enantiomers.

Conditions: Chiralpak IB-3 column, 90:10 *n*-hexane:IPA, flow rate: 1.0 mL min^{-1}, 25 ºC, UV detection wavelength: 254 nm. Retention times: 23.1 min (1*R*,2*R* enantiomer), 25.9 min (1*S*,2*S* enantiomer).

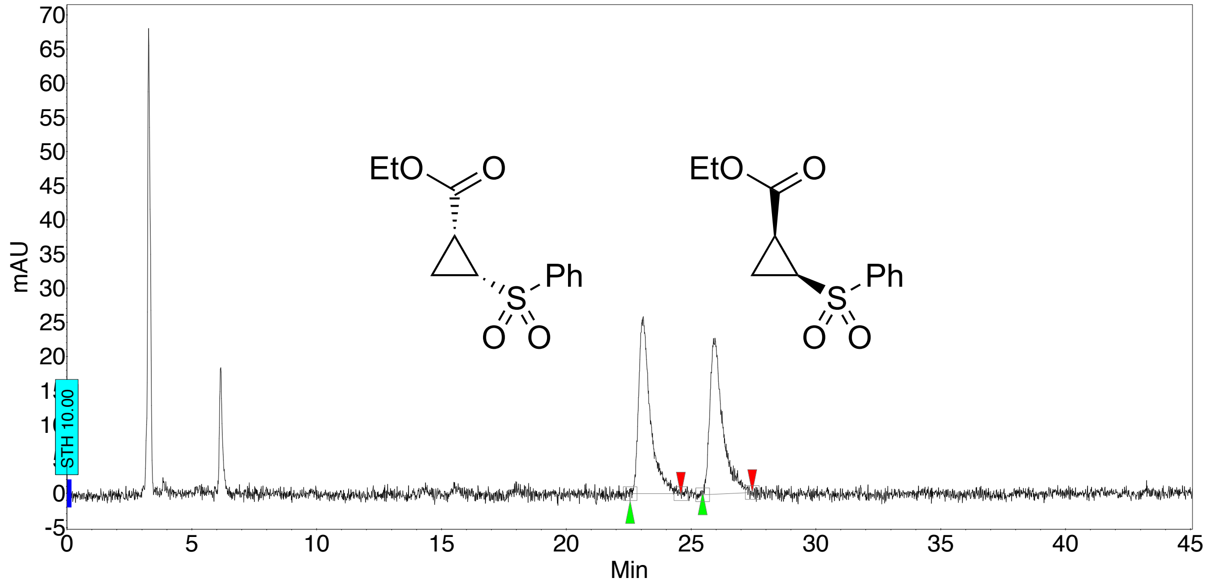

Peak results :

Index	Name	Time [Min]	Quantity [% Area]	Height [mAU]	Area [mAU.Min]	Area % [%]
1	UNKNOWN	23.065	50.78	25.7	14.0	50.777
2	UNKNOWN	25.931	49.22	21.9	13.6	49.223
Total			100.00	47.5	27.5	100.000

Enantioenriched *Z*-Ethyl 2-(benzenesulfonyl)cyclopropane-1-carboxylate (*Z*-Ethyl 2-(phenylsulfanyl)-cyclopropane-1-carboxylate was oxidised to the corresponding sulfone to facilitate separation of the enantiomers.

Conditions: Chiralpak IB-3 column, 95:5 *n*-hexane:IPA, flow rate: 1.0 mL min^{-1}, 25 ºC, UV detection wavelength: 254 nm. Retention times: 23.1 min (1*R*,2*R* enantiomer), 25.9 min (1*S*,2*S* enantiomer).

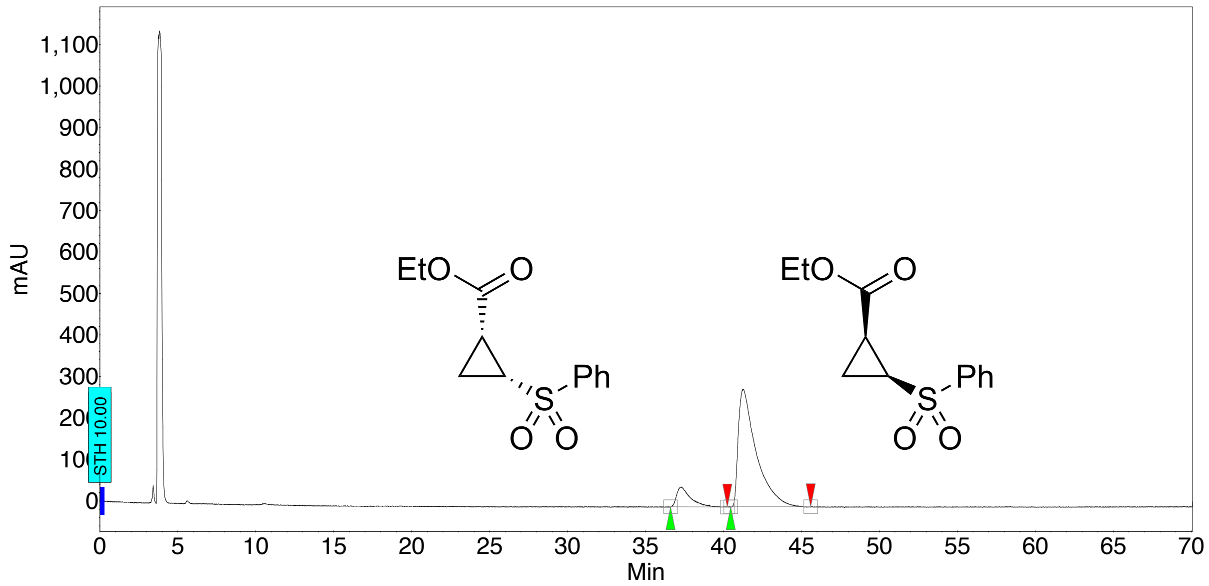

Peak results :

Index	Name	Time [Min]	Quantity [% Area]	Height [mAU]	Area [mAU.Min]	Area % [%]
1	UNKNOWN	37.267	11.40	47.1	48.5	11.397
2	UNKNOWN	41.252	88.60	283.3	377.3	88.603
Total			100.00	330.4	425.8	100.000

ee = <u>77.2%</u>

Preparative chiral supercritical fluid chromatography (SFC) to obtain highly enantioenriched cyclopropanes

Compounds **1** and **2** were obtained in high ee through preparative chiral SFC using an SFC Minigram. Using this technique, approximately 100-300 mg of each enantiomer was obtained.

Preparative chiral SFC of *E*-ethyl 2-(phenylsulfanyl)-cyclopropane-1-carboxylate

Conditions: ADH column, 94:6 CO_2:MeOH, flow = 5.0 mL min^{-1}, pressure = 100 bar, temperature = 35 °C, UV detection at 220 nm.

Ethyl (1*S*,2*R*)-2-(phenylsulfanyl)-cyclopropane-1-carboxylate

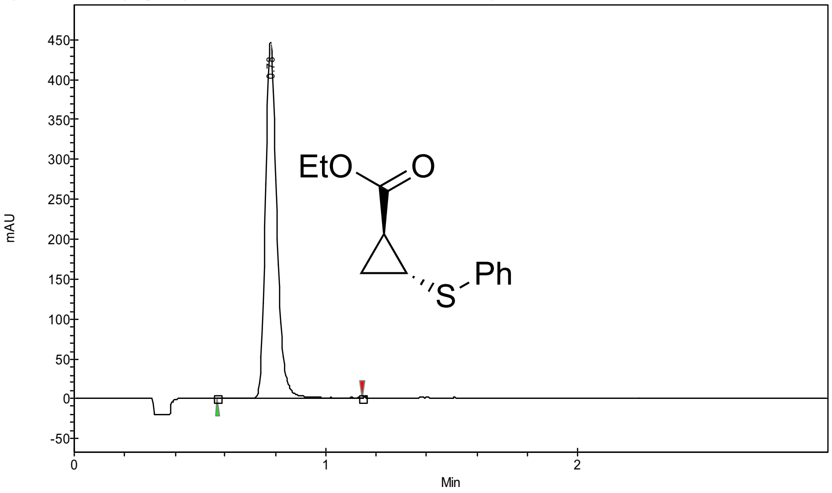

Index	Time	Width 10%	Height	Area	Area
	[Min]	[Min]	[mAU]	[mAU*min]	[%]
1	0.78	0.09	446.59	23.25	100.000
Total				23.25	100.000

Ethyl (*1R*,2*S*)-2-(phenylsulfanyl)-cyclopropane-1-carboxylate

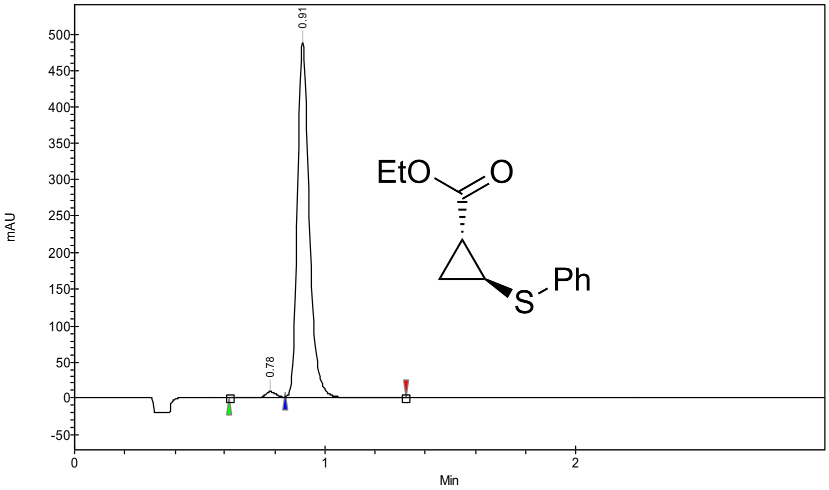

Index	Time	Width 10%	Height	Area	Area
	[Min]	[Min]	[mAU]	[mAU*min]	[%]
1	0.78	0.09	8.30	0.40	1.451
2	0.91	0.10	489.30	27.14	98.549
Total				27.54	100.000

Preparative chiral SFC of *Z*-ethyl 2-(phenylsulfanyl)-cyclopropane-1-carboxylate

Conditions: ADH column, 92:8 CO_2:EtOH + 1% (2M NH_3 in MeOH), flow = 5.0 mL min^{-1}, pressure = 100 bar, temperature = 35 °C, UV detection at 220 nm.

Ethyl (*1S*,2*S*)-2-(phenylsulfanyl)-cyclopropane-1-carboxylate

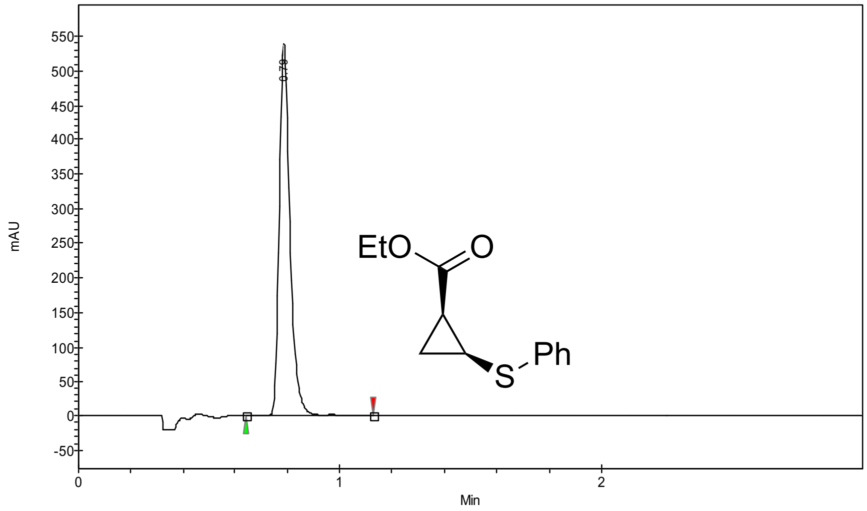

Index	Time	Width 10%	Height	Area	Area
	[Min]	[Min]	[mAU]	[mAU*min]	[%]
1	0.79	0.08	539.19	25.65	100.000
Total				25.65	100.000

Ethyl (*1R*,2*R*)-2-(phenylsulfanyl)-cyclopropane-1-carboxylate

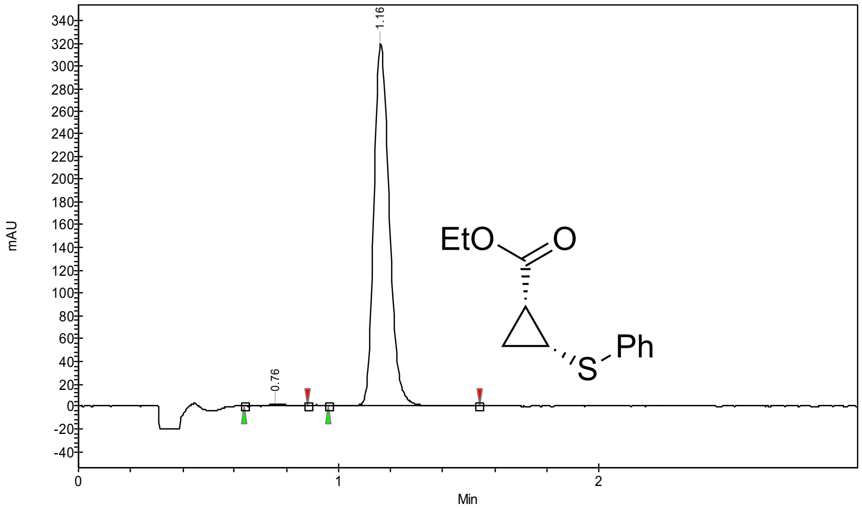

Index	Time	Width 10%	Height	Area	Area
	[Min]	[Min]	[mAU]	[mAU*min]	[%]
1	0.76	0.08	1.60	0.06	0.294
2	1.16	0.12	319.43	21.50	99.706
Total				21.57	100.000

Optical rotation data for enantiopure cyclopropanes 1, 2, 4 and 5

The $[\alpha]_D^{20}$ data for each enantiopure compound generated in this work is reported below (Figure **S3**). Optical rotations (α') were recorded at 20 °C and were converted to the corresponding specific rotations $[\alpha]_D^{20}$.

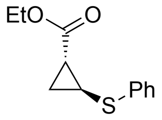	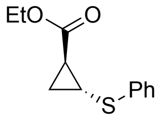	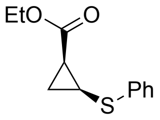	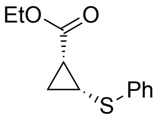
+57° (c = 0.257)	–59° (c = 0.293)	–375° (c = 0.229)	+387° (c = 0.248)
(1*R*,2*S*)	(1*S*,2*R*)	(1*S*,2*S*)	(1*R*,2*R*)
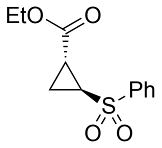	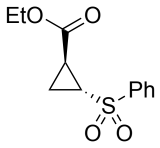	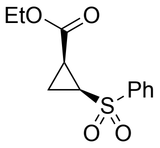	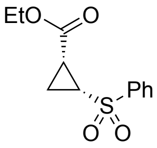
+50° (c = 0.282)	–50° (c = 0.278)	–38° (c = 0.250)	+42° (c = 0.270)
(1*R*,2*S*)	(1*S*,2*R*)	(1*S*,2*S*)	(1*R*,2*R*)

Figure S3: Specific optical rotation data and stereochemical configuration for enantiopure synthesized compounds.

^{1}H, ^{13}C and ^{11}B NMR spectra

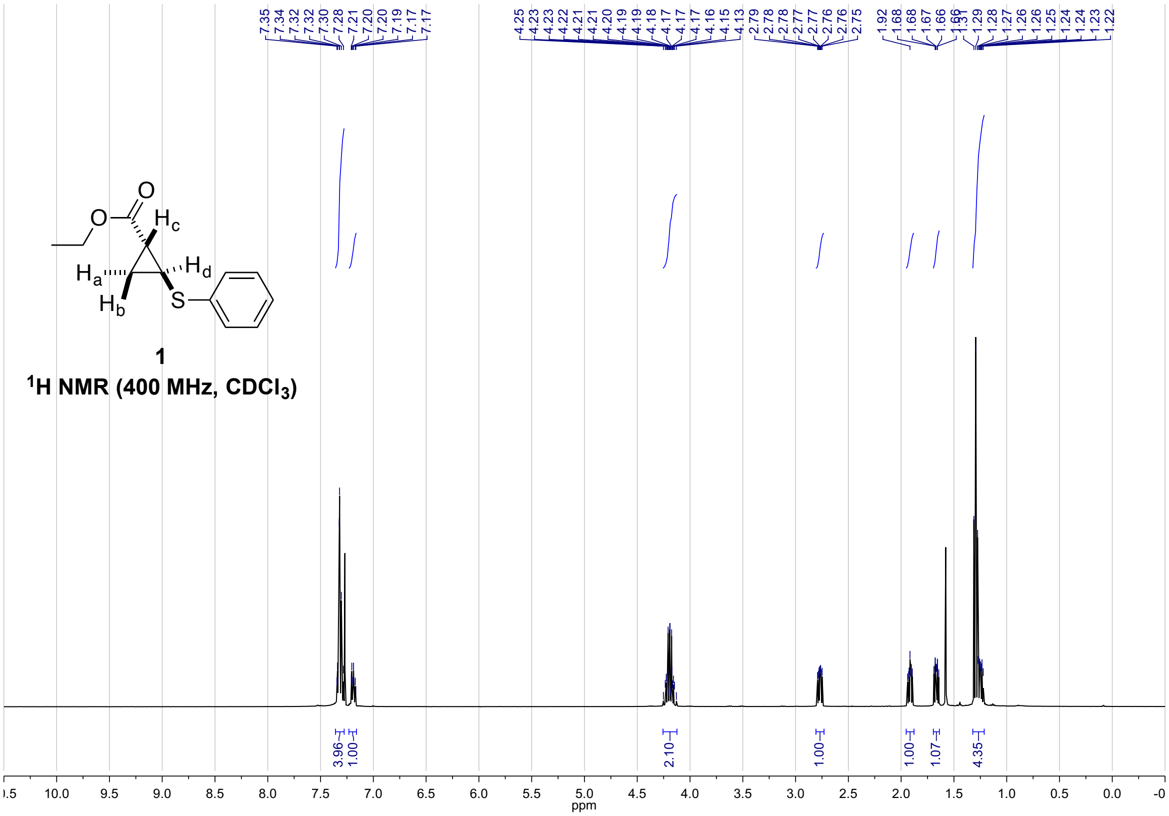
1
^{1}H NMR (400 MHz, $CDCl_3$)
7.35 7.34 7.32 7.32 7.30 7.28 7.21 7.20 7.20 7.19 7.17 7.17
4.25 4.23 4.23 4.22 4.21 4.21 4.20 4.19 4.19 4.18 4.17 4.17 4.17 4.16 4.15 4.13
2.79 2.78 2.78 2.77 2.77 2.76 2.76 2.75
1.92 1.68 1.67 1.66 1.29 1.28 1.27 1.26 1.26 1.25 1.24 1.24 1.23 1.22
3.96 1.00 2.10 1.00 1.00 1.07 4.35
ppm

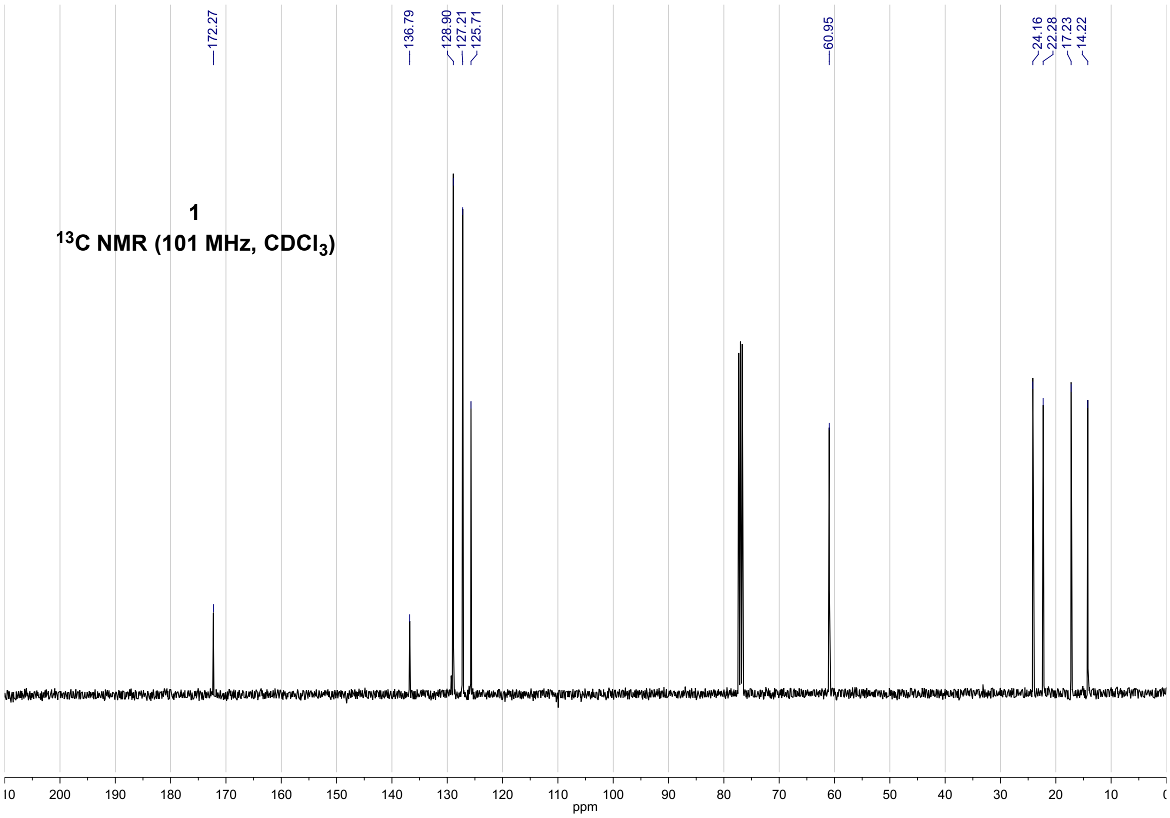
1
^{13}C NMR (101 MHz, $CDCl_3$)
172.27
136.79
128.90
127.21
125.71
60.95
24.16
22.28
17.23
14.22
ppm

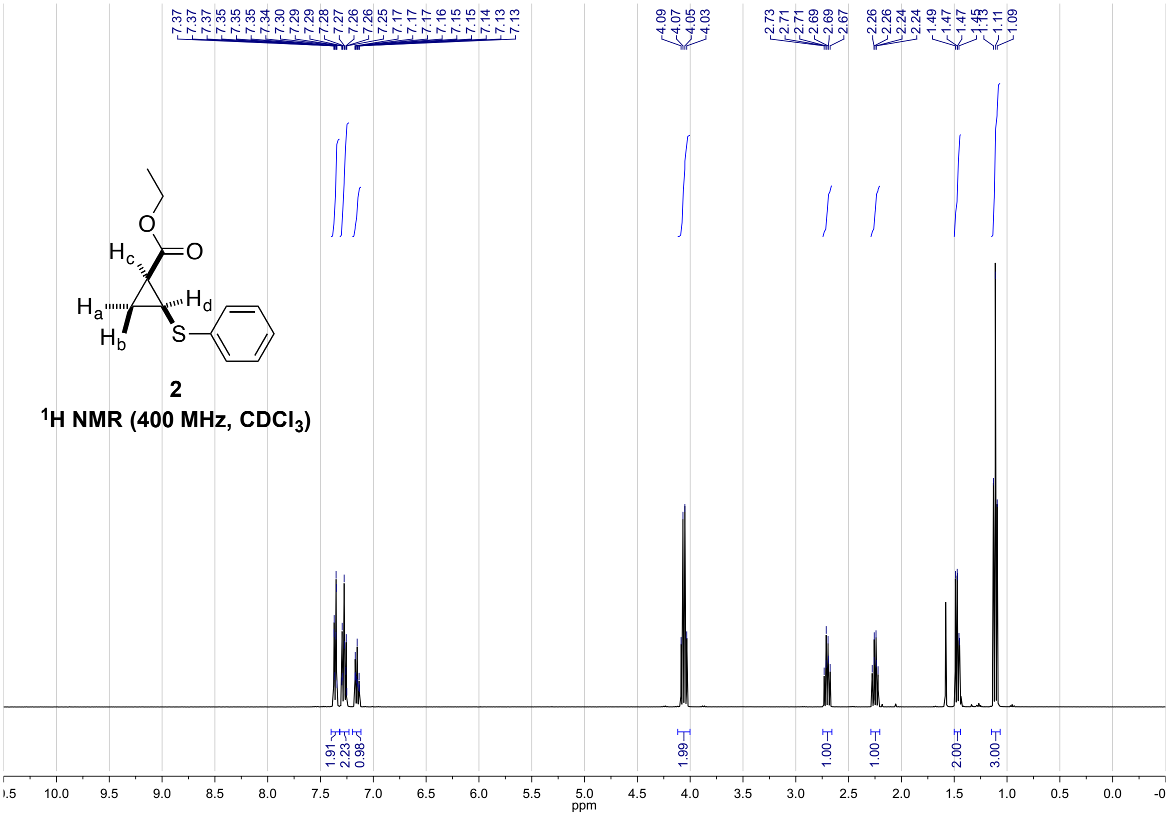

2

^{1}H NMR (400 MHz, $CDCl_3$)

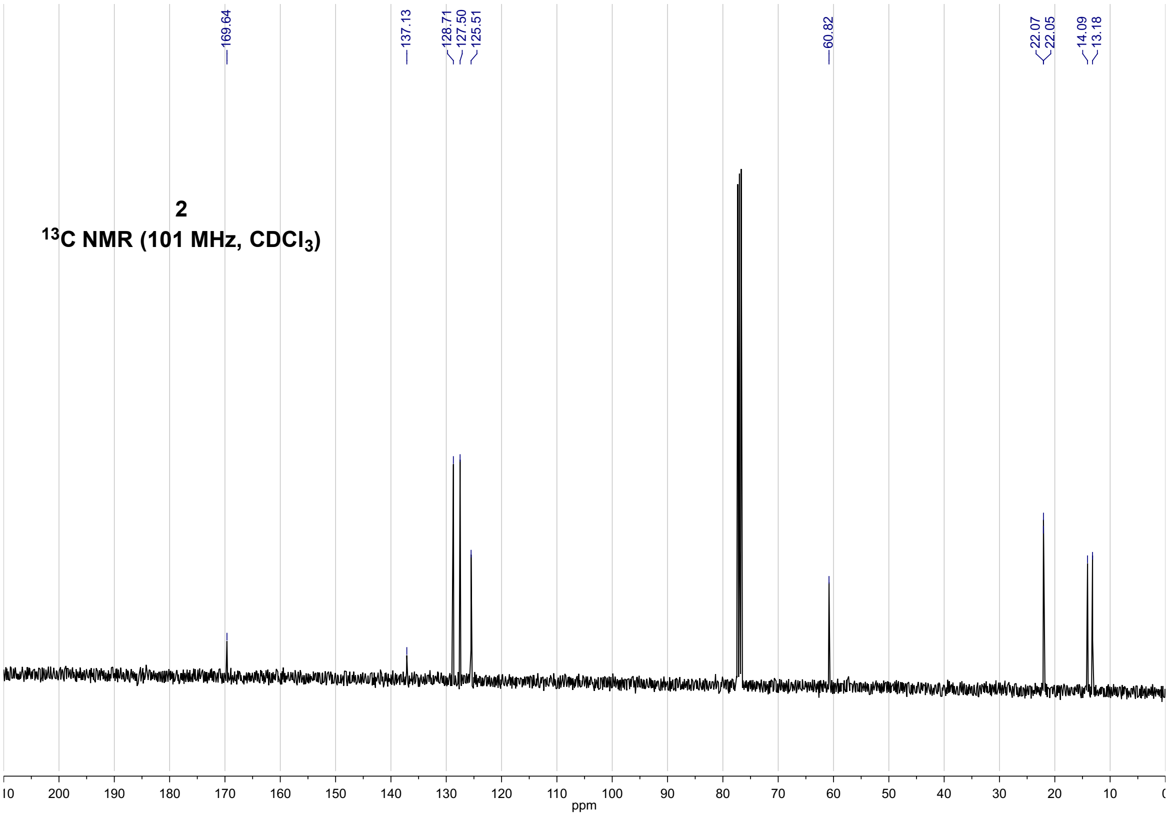

2

^{13}C NMR (101 MHz, $CDCl_3$)

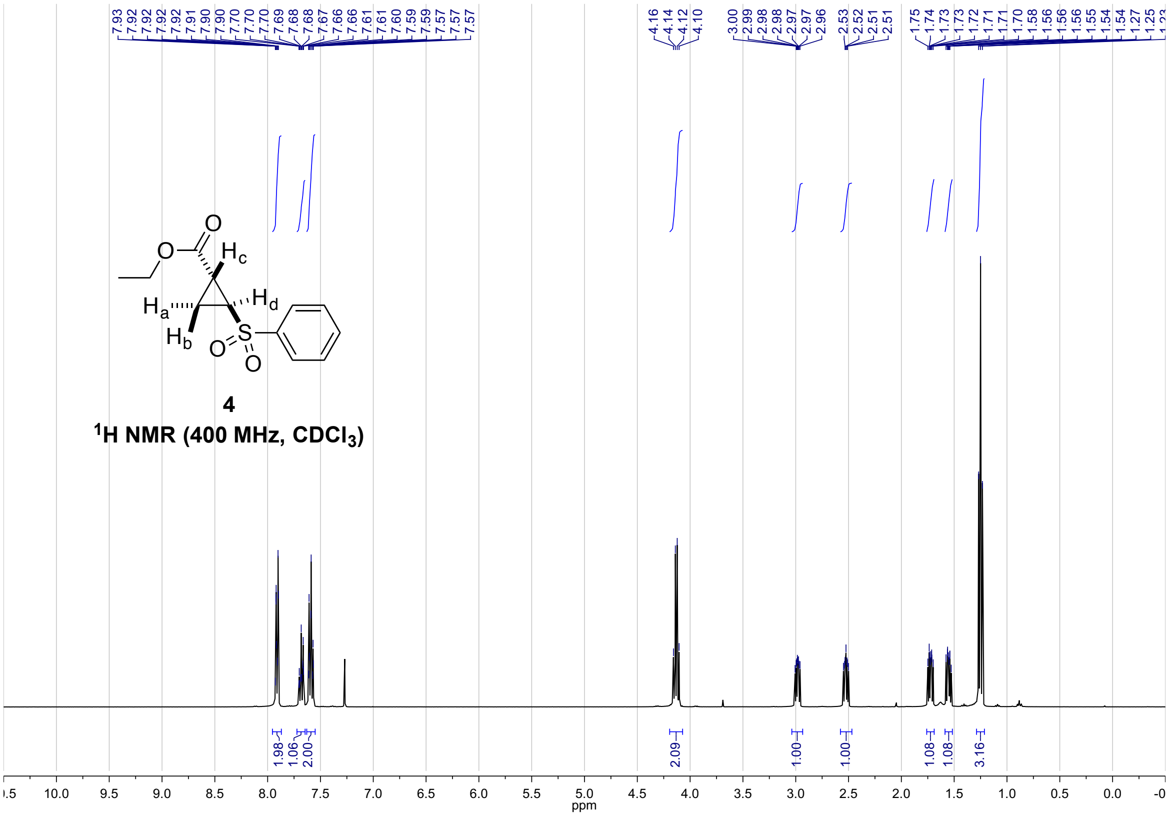

4
^{1}H NMR (400 MHz, CDCl$_3$)

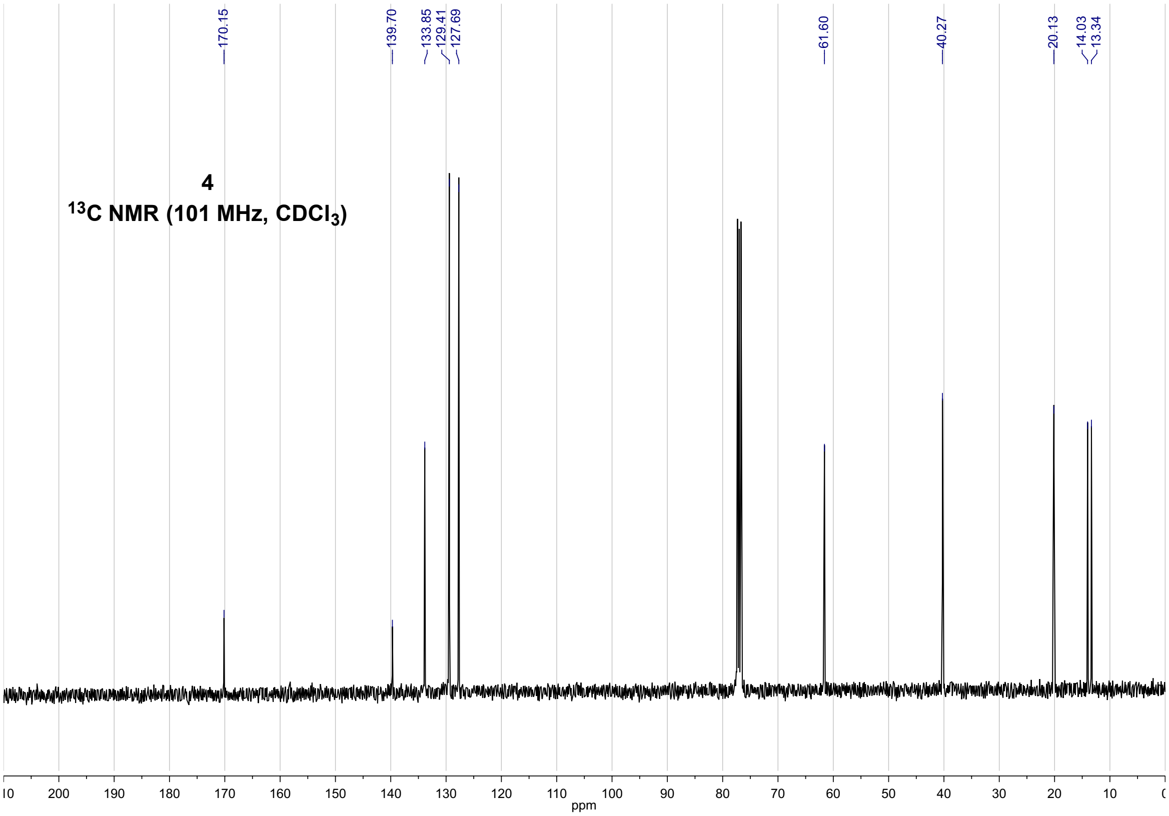

4
^{13}C NMR (101 MHz, CDCl$_3$)

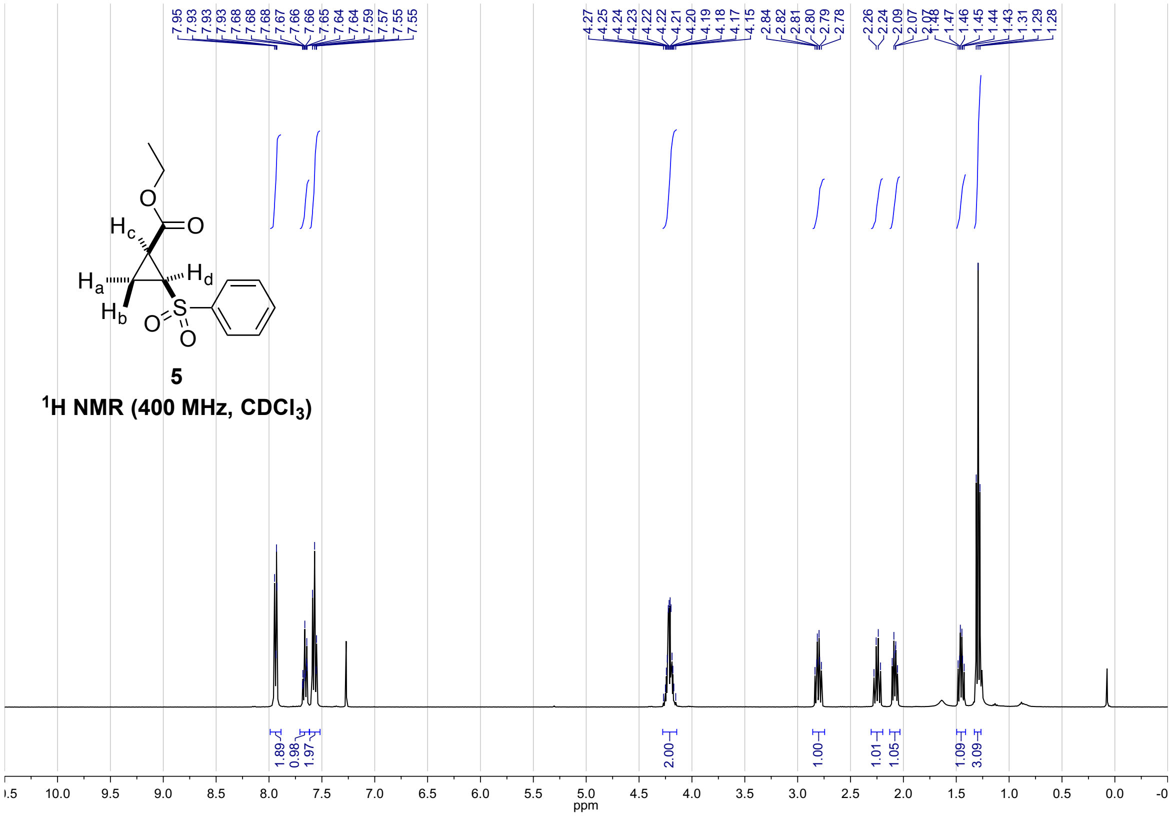
5
^{1}H NMR (400 MHz, CDCl$_3$)
ppm

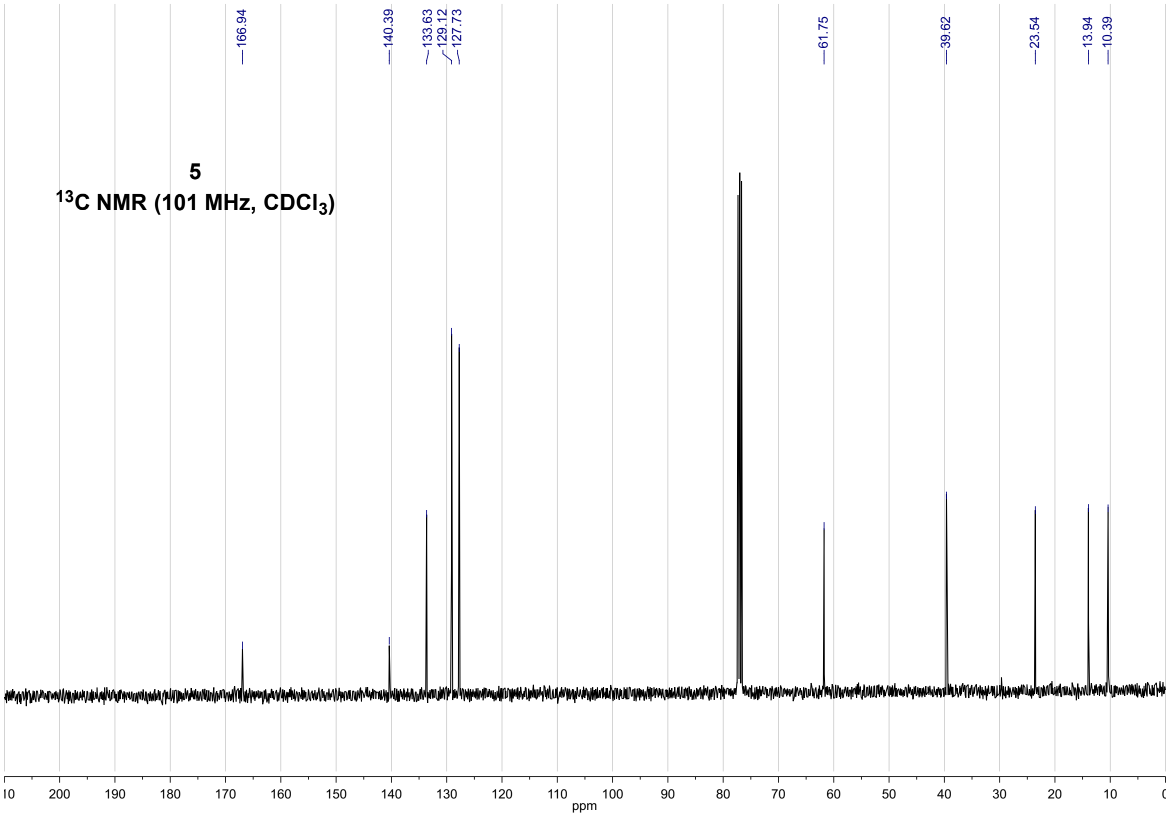
5
^{13}C NMR (101 MHz, CDCl$_3$)
166.94
140.39
133.63
129.12
127.73
61.75
39.62
23.54
13.94
10.39
ppm

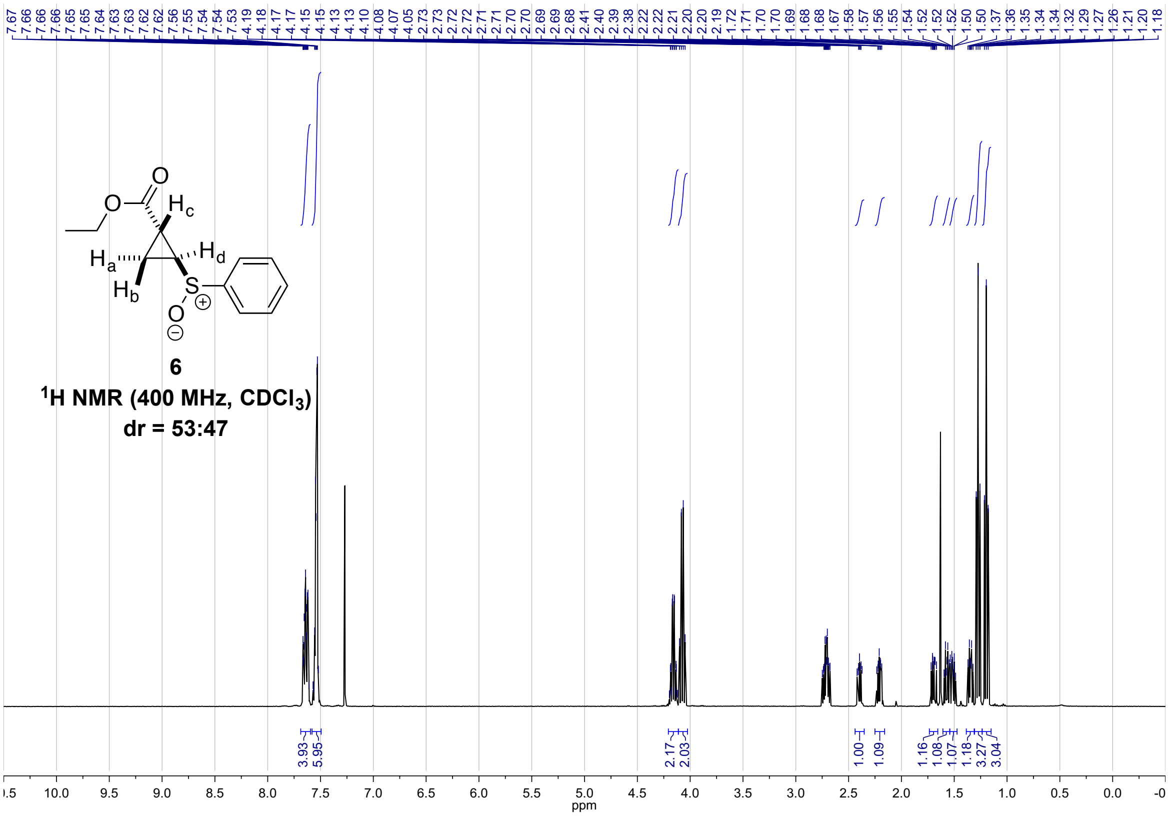
7.67 7.66 7.66 7.65 7.65 7.64 7.63 7.63 7.62 7.62 7.56 7.55 7.54 7.54 7.53 4.19 4.18 4.17 4.17 4.15 4.15 4.13 4.13 4.10 4.08 4.07 4.05 2.73 2.73 2.72 2.72 2.71 2.71 2.70 2.70 2.69 2.69 2.68 2.41 2.40 2.39 2.38 2.22 2.22 2.21 2.20 2.20 2.19 1.72 1.72 1.71 1.70 1.69 1.68 1.67 1.58 1.57 1.56 1.55 1.54 1.52 1.52 1.52 1.50 1.50 1.37 1.36 1.35 1.34 1.32 1.29 1.27 1.26 1.21 1.20 1.18
O
O
Hc
Ha
Hd
Hb
S
6
1H NMR (400 MHz, CDCl3)
dr = 53:47
3.93 5.95 2.17 2.03 1.00 1.09 1.16 1.08 1.07 1.18 3.27 3.04
ppm

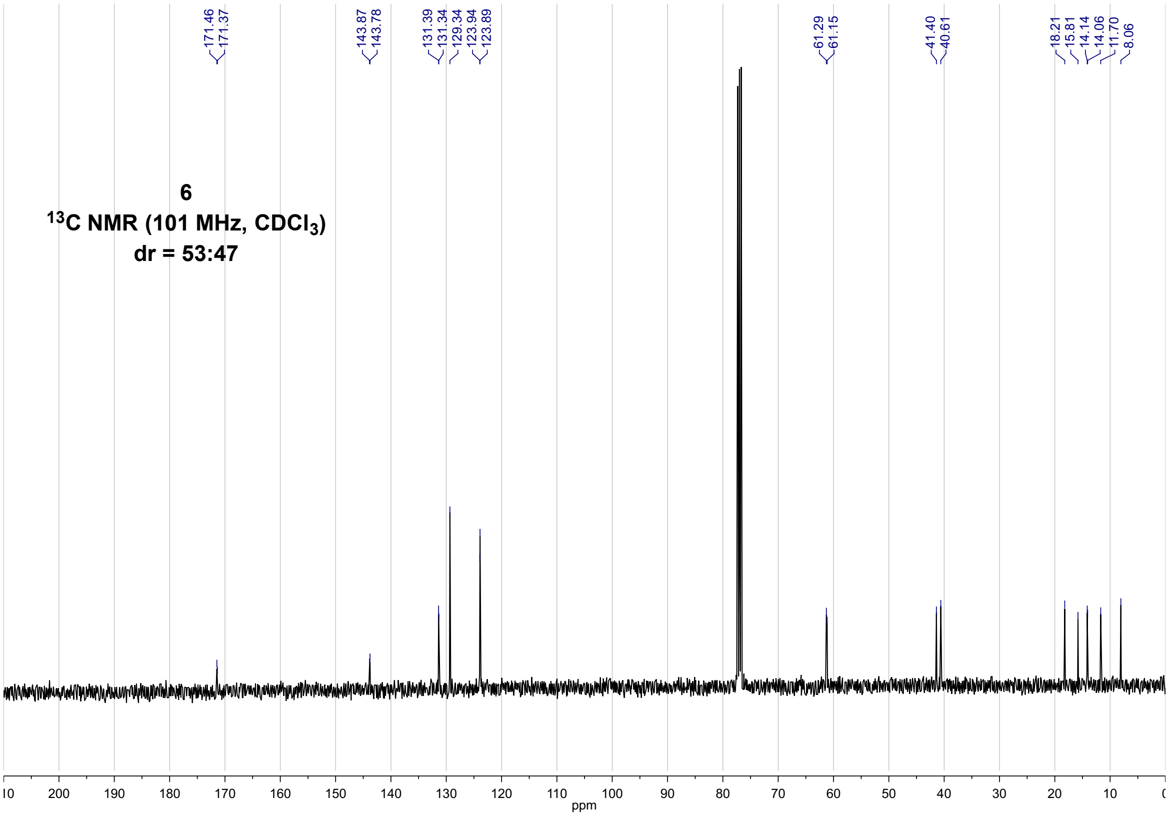
171.46 171.37 143.87 143.78 131.39 131.34 129.34 123.94 123.89 61.29 61.15 41.40 40.61 18.21 15.81 14.14 14.06 11.70 8.06
6
13C NMR (101 MHz, CDCl3)
dr = 53:47
ppm

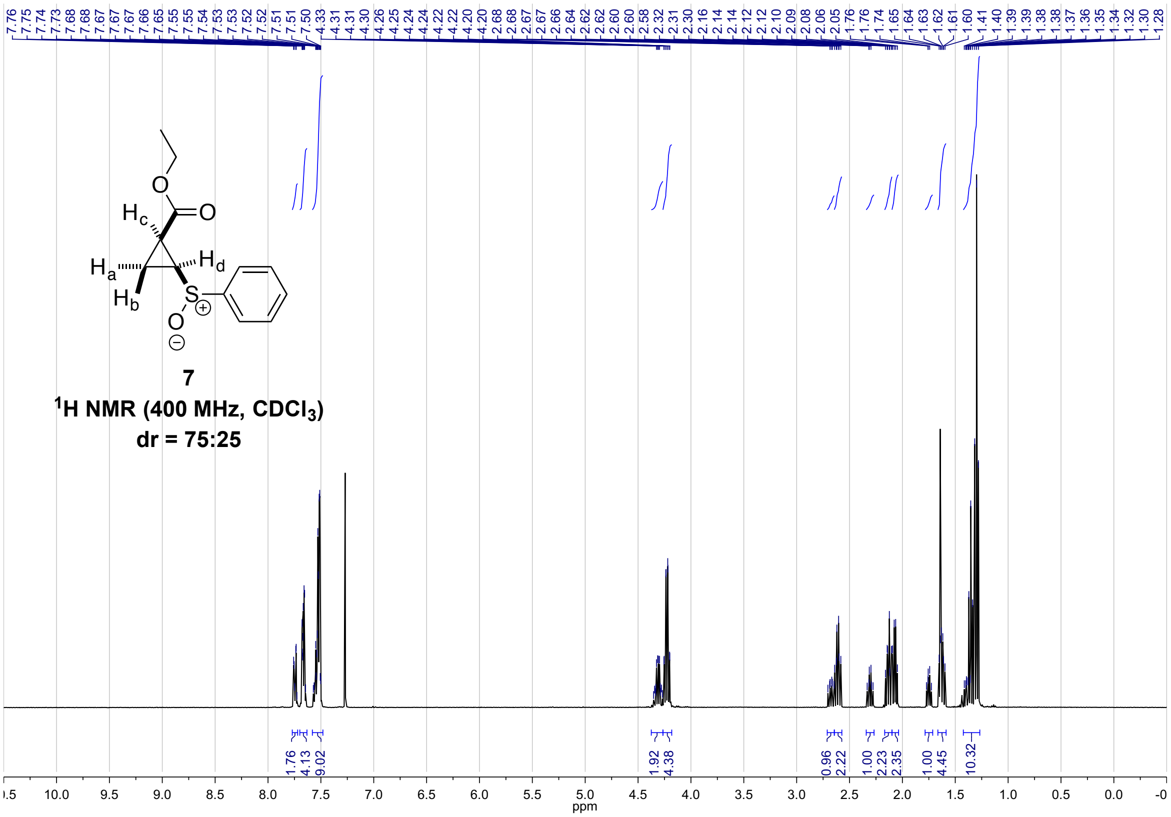

7
^{1}H NMR (400 MHz, CDCl$_3$)
dr = 75:25
ppm

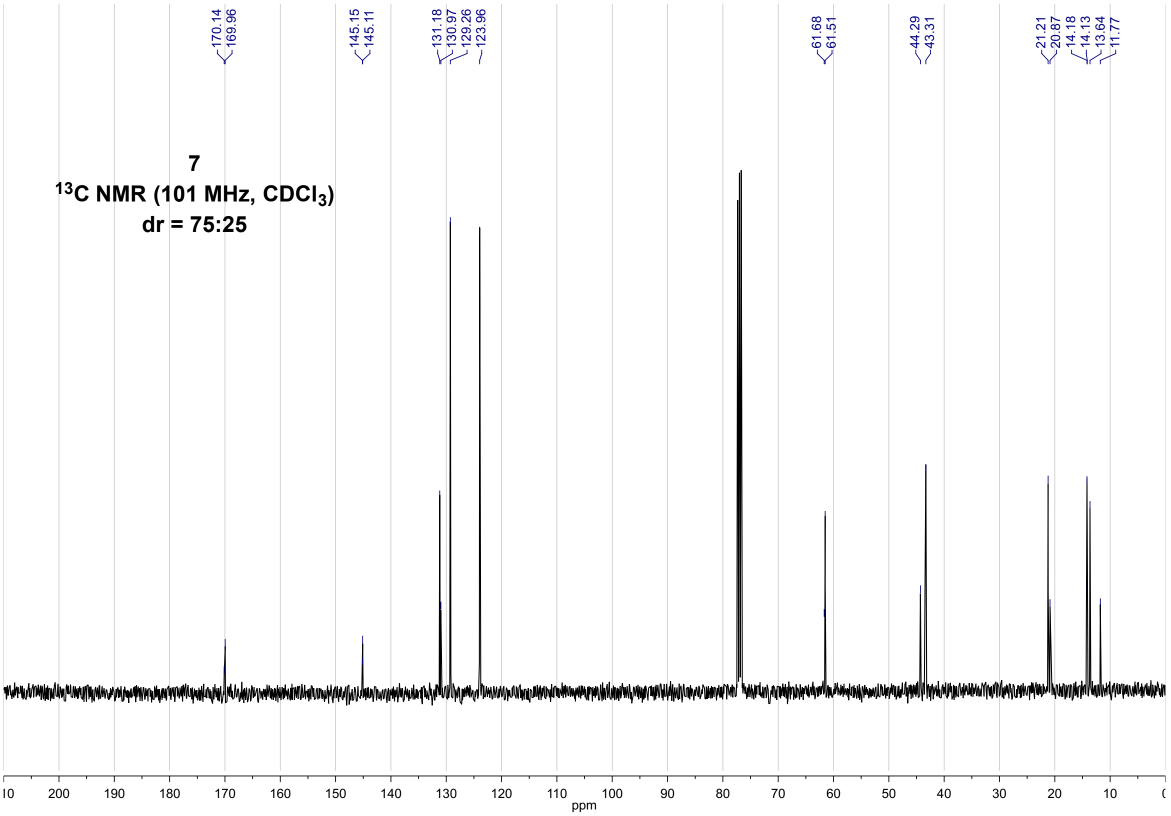

7
^{13}C NMR (101 MHz, CDCl$_3$)
dr = 75:25
170.14
169.96
145.15
145.11
131.18
130.97
129.26
123.96
61.68
61.51
44.29
43.31
21.21
20.87
14.18
14.13
13.64
11.77
ppm

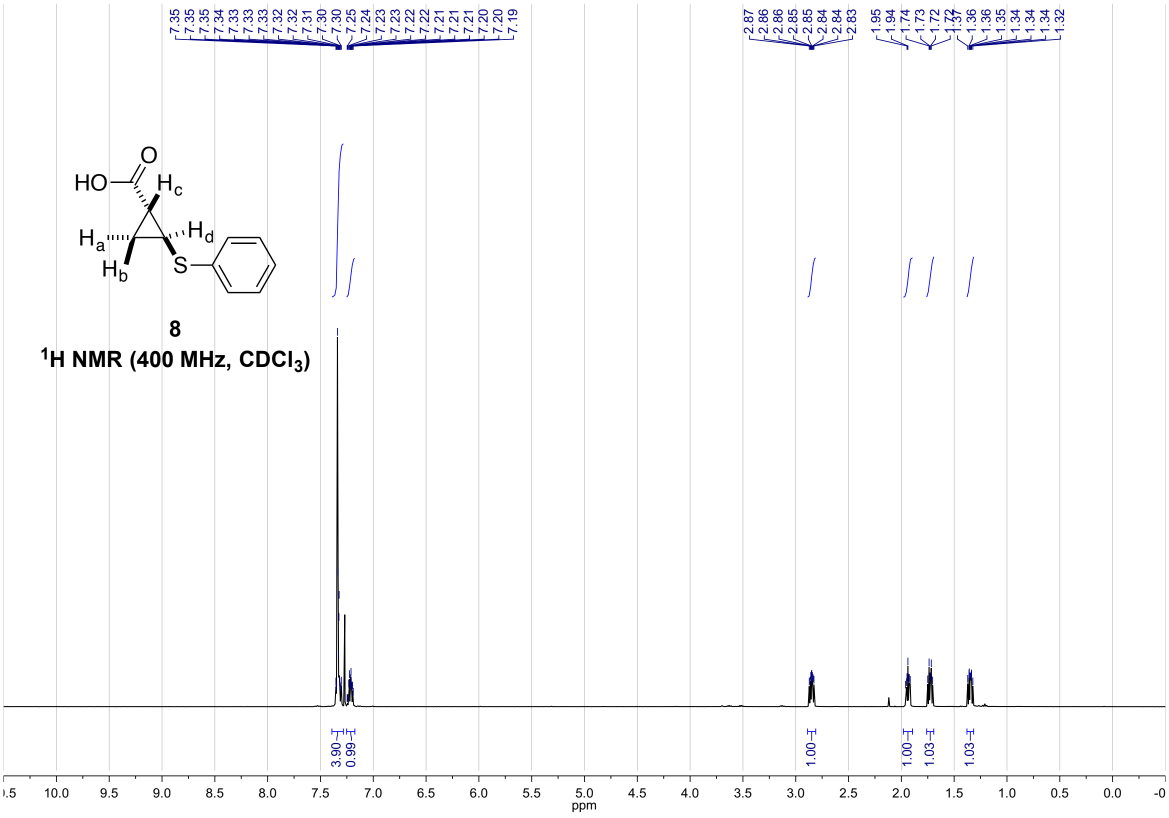
HO
O
Hc
Ha
Hd
Hb
S
8
1H NMR (400 MHz, CDCl3)
ppm

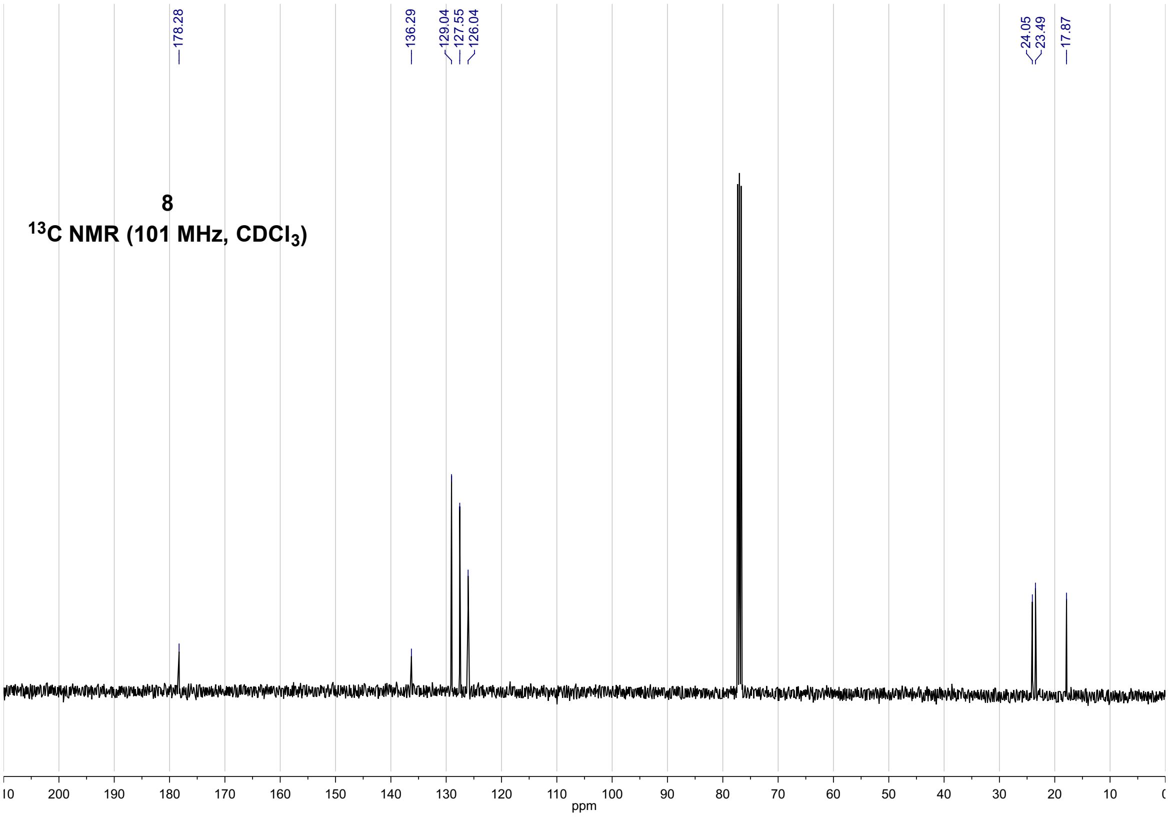
178.28
136.29
129.04
127.55
126.04
24.05
23.49
17.87
8
13C NMR (101 MHz, CDCl3)
ppm

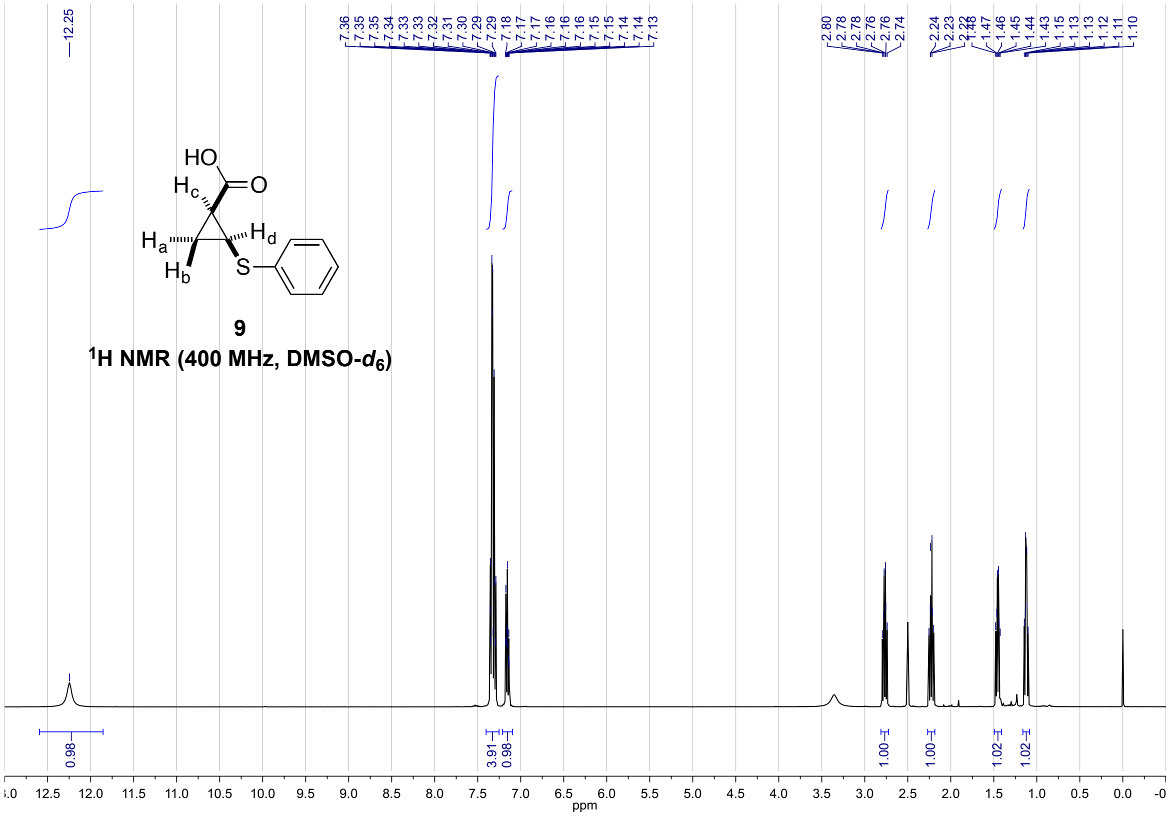

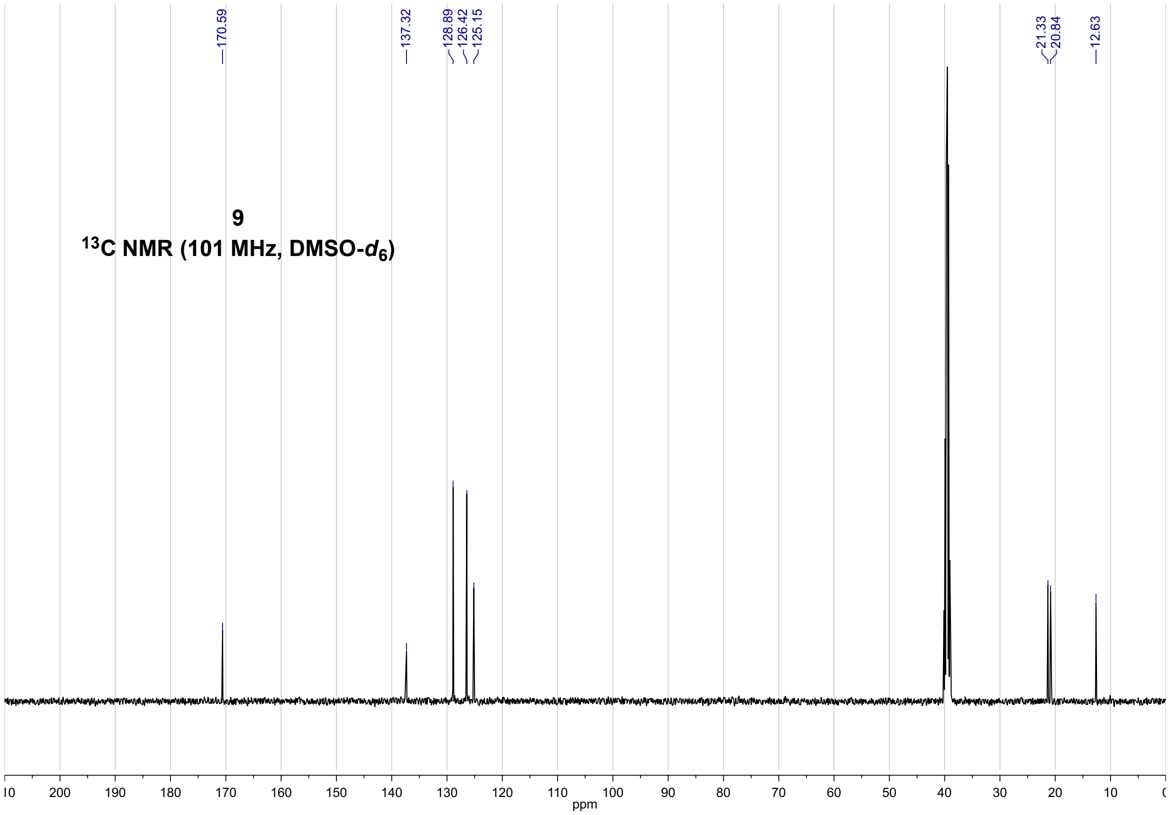

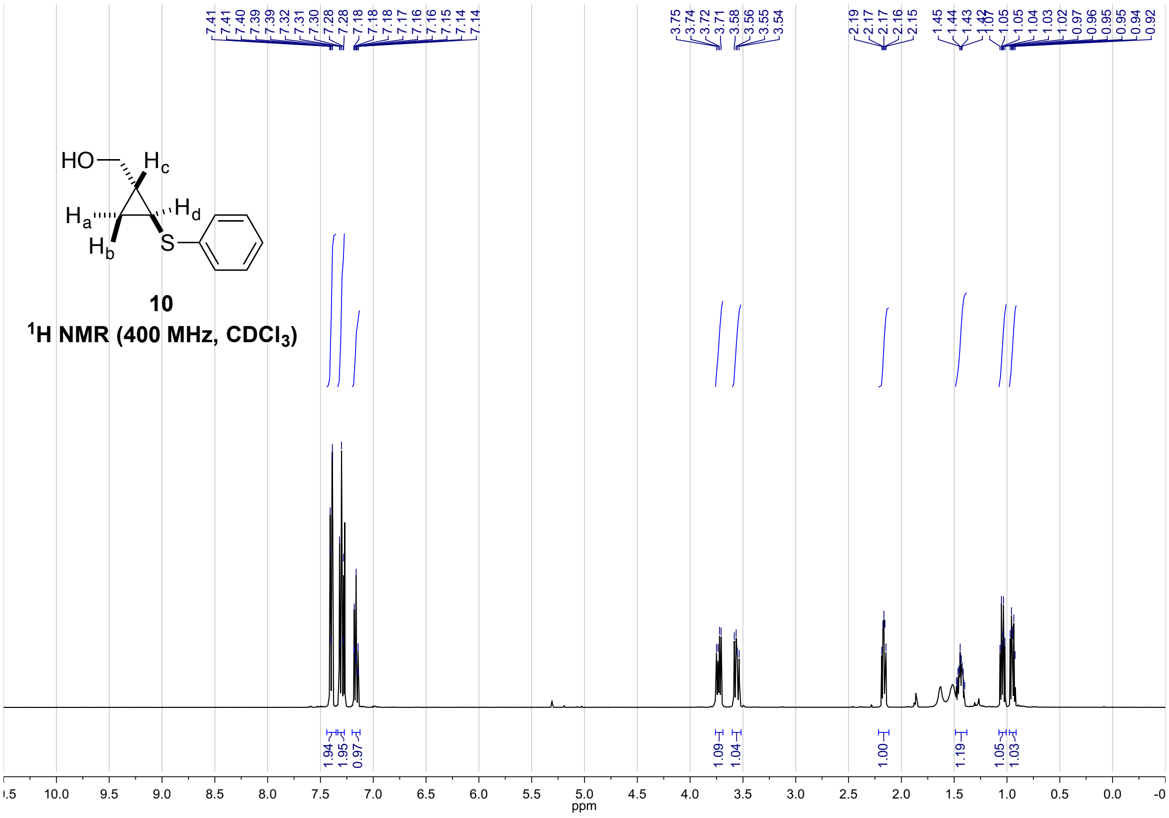

10

^{1}H NMR (400 MHz, CDCl$_3$)

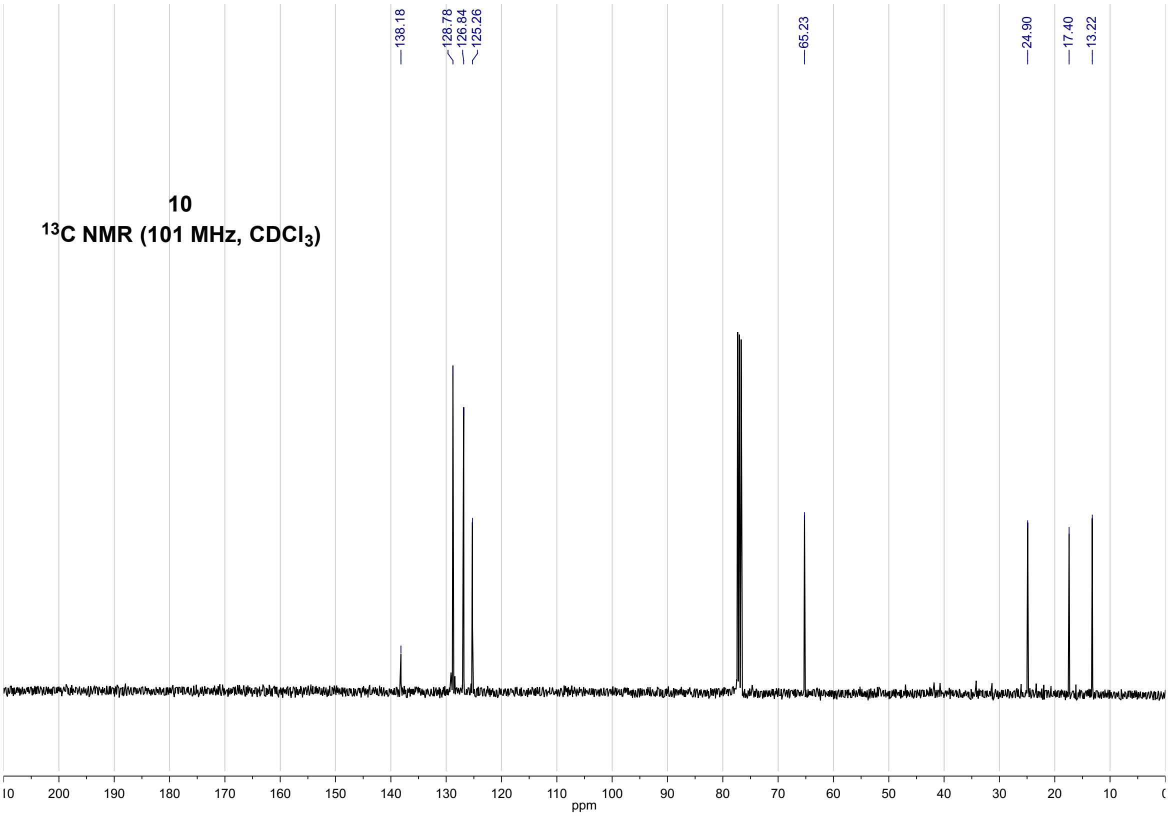

10

^{13}C NMR (101 MHz, CDCl$_3$)

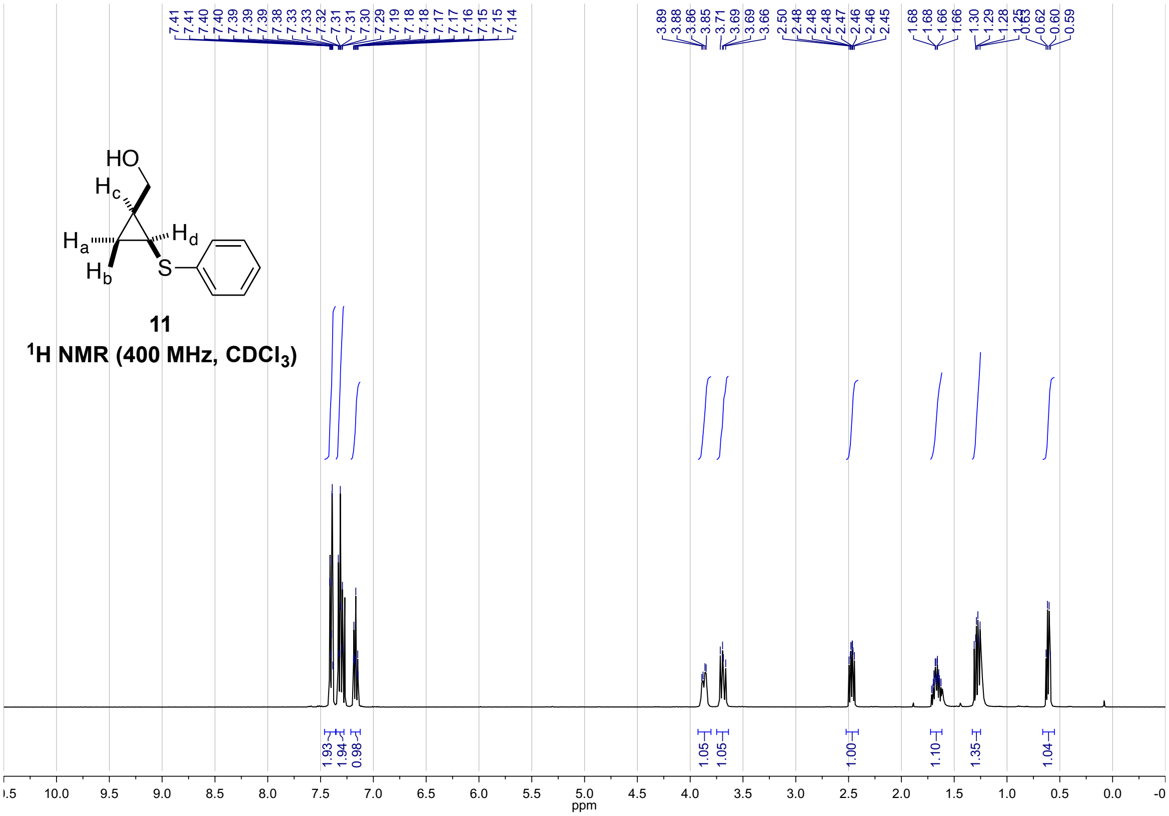
7.41
7.41
7.40
7.39
7.39
7.39
7.38
7.33
7.32
7.31
7.31
7.30
7.29
7.19
7.18
7.18
7.17
7.16
7.15
7.14
3.89
3.88
3.86
3.85
3.71
3.69
3.69
3.66
2.50
2.48
2.48
2.48
2.47
2.46
2.46
2.45
1.68
1.68
1.66
1.66
1.30
1.29
1.28
1.25
0.63
0.62
0.60
0.59
HO
H_c
H_a
H_d
H_b
S
11
^{1}H NMR (400 MHz, CDCl$_3$)
1.93
1.94
0.98
1.05
1.05
1.00
1.10
1.35
1.04
ppm

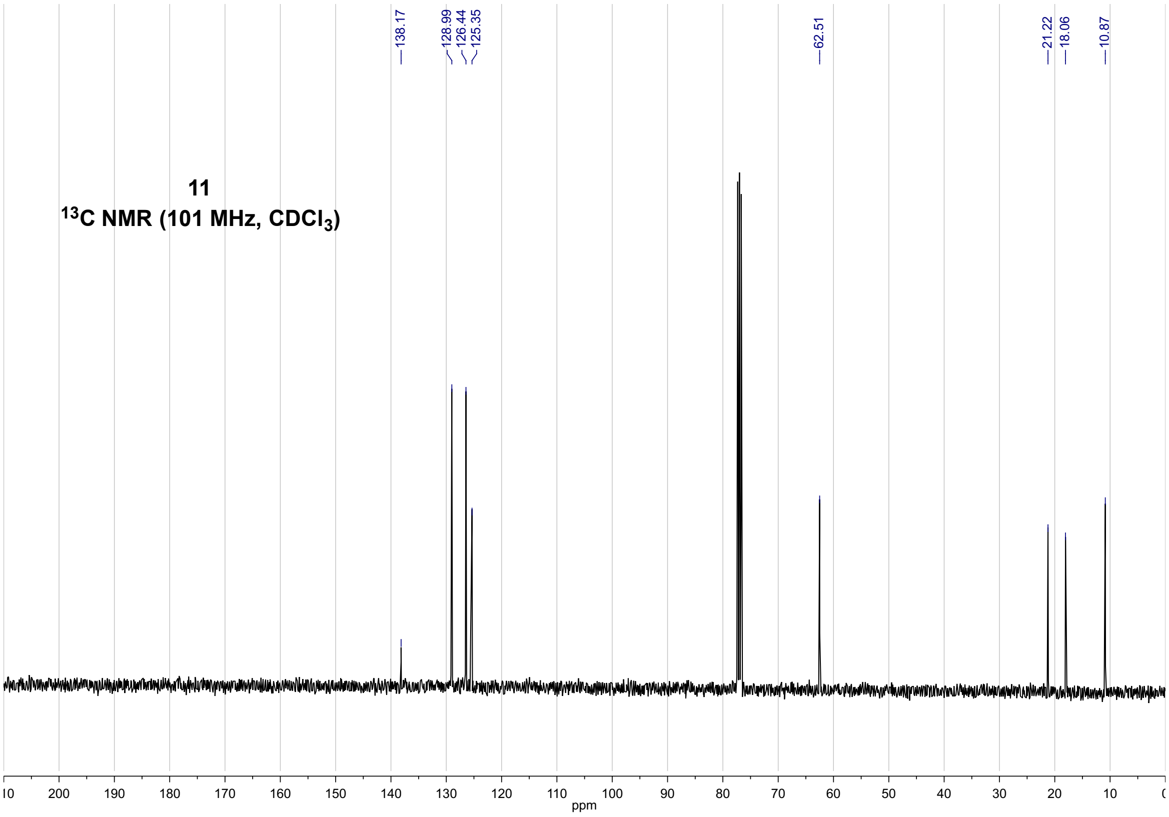
138.17
128.99
126.44
125.35
62.51
21.22
18.06
10.87
11
^{13}C NMR (101 MHz, CDCl$_3$)
ppm

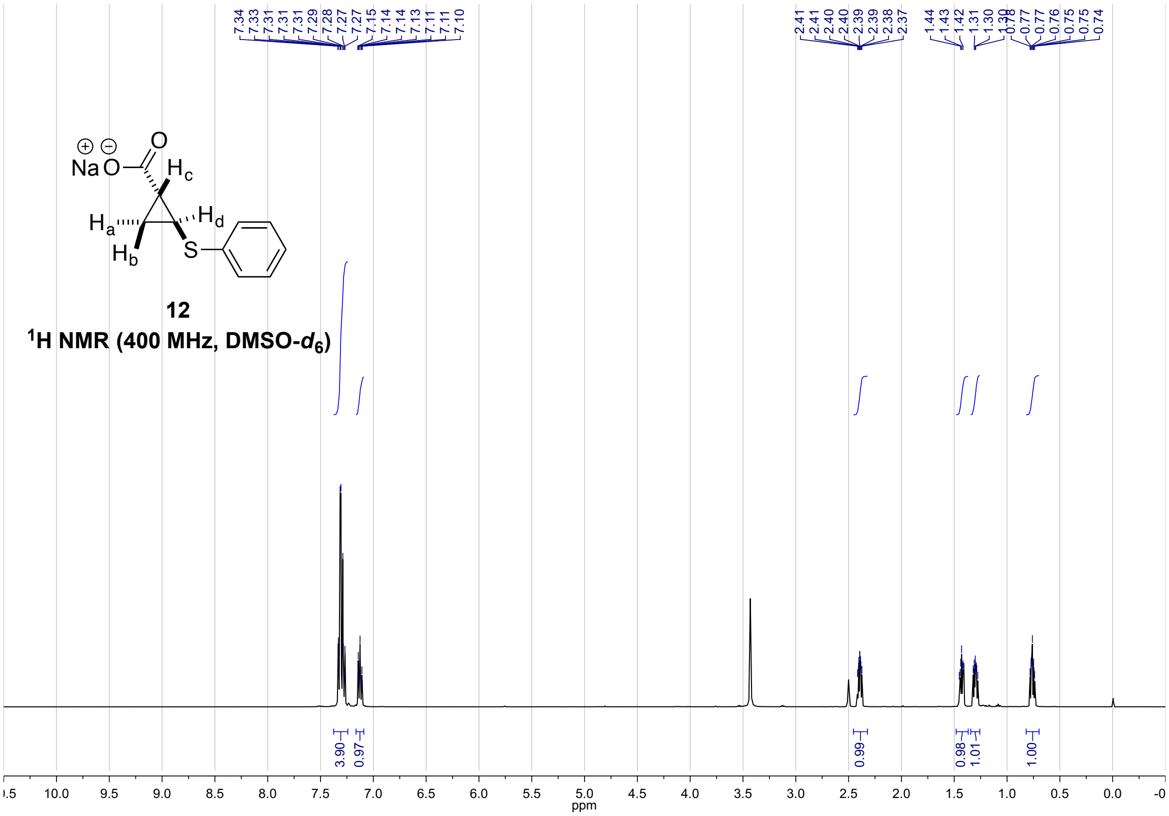

12
^{1}H NMR (400 MHz, DMSO-d_6)

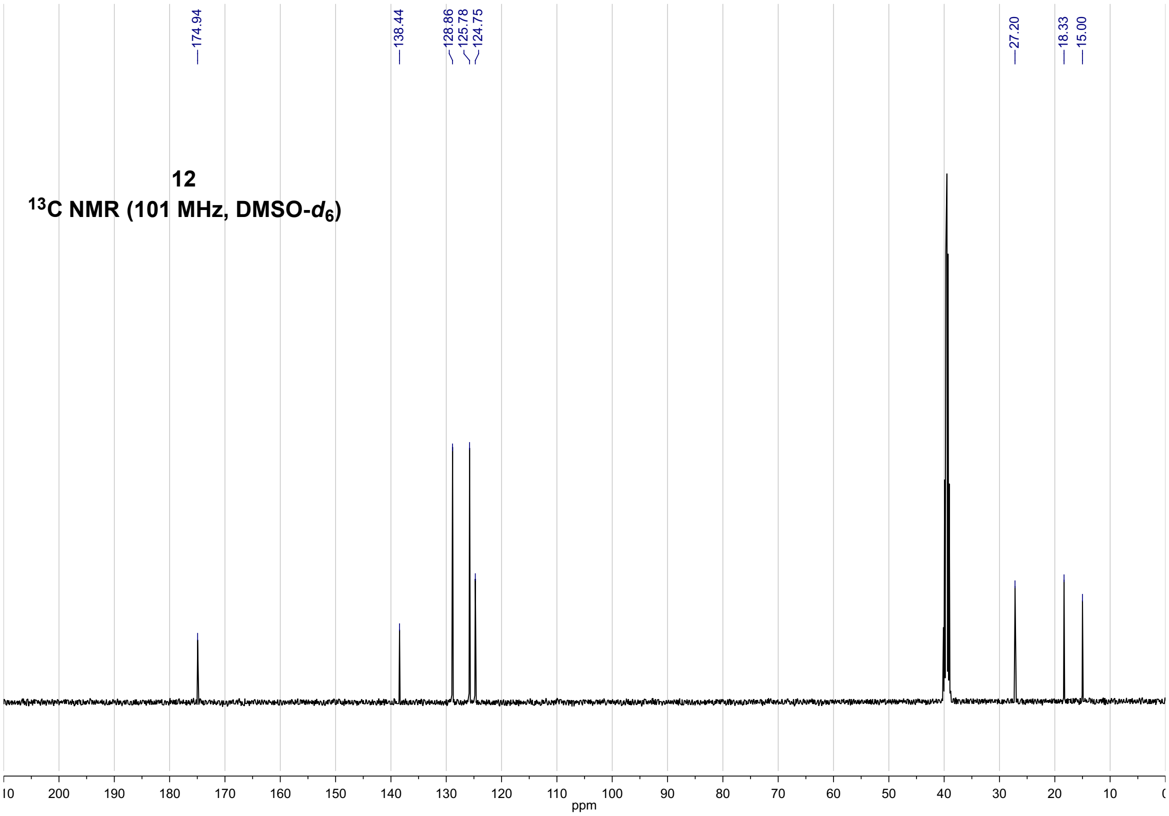

12
^{13}C NMR (101 MHz, DMSO-d_6)

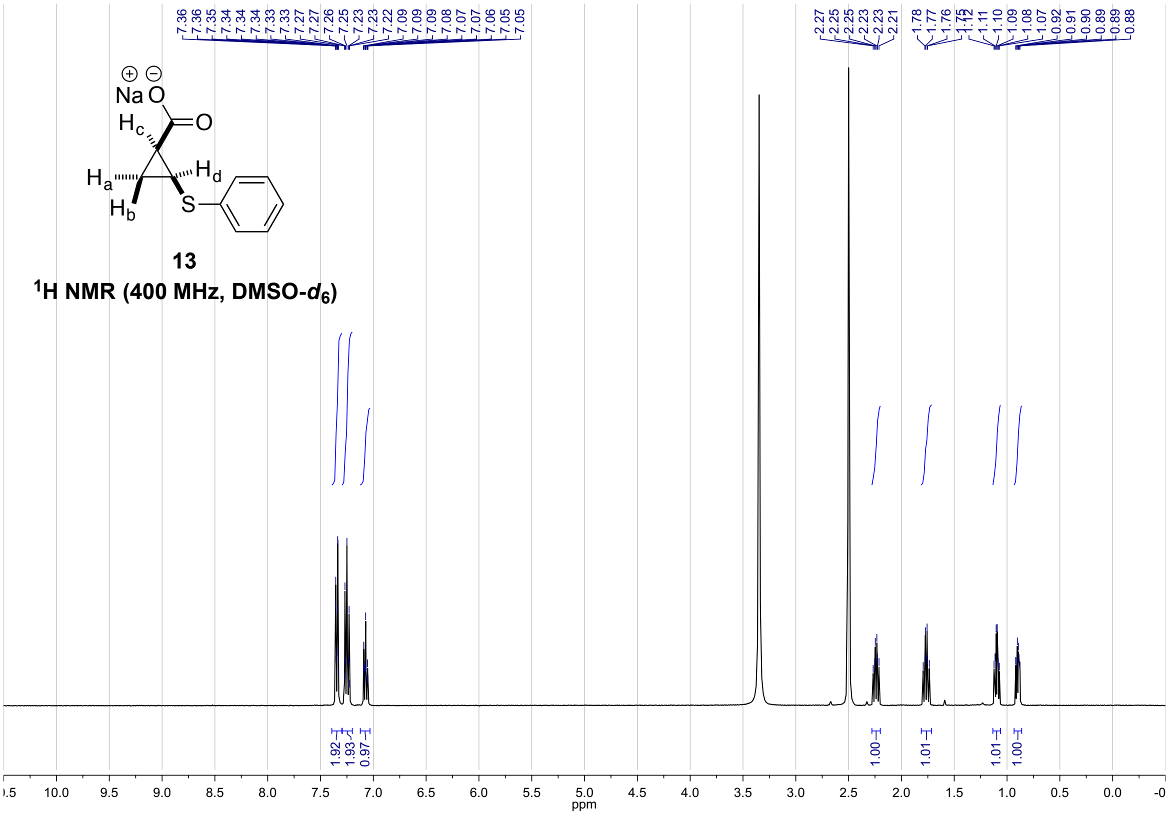
Na O
H$_c$
O
H$_a$
H$_d$
H$_b$
S
13
^{1}H NMR (400 MHz, DMSO-d_6)
1.92
1.93
0.97
1.00
1.01
1.01
1.00
ppm

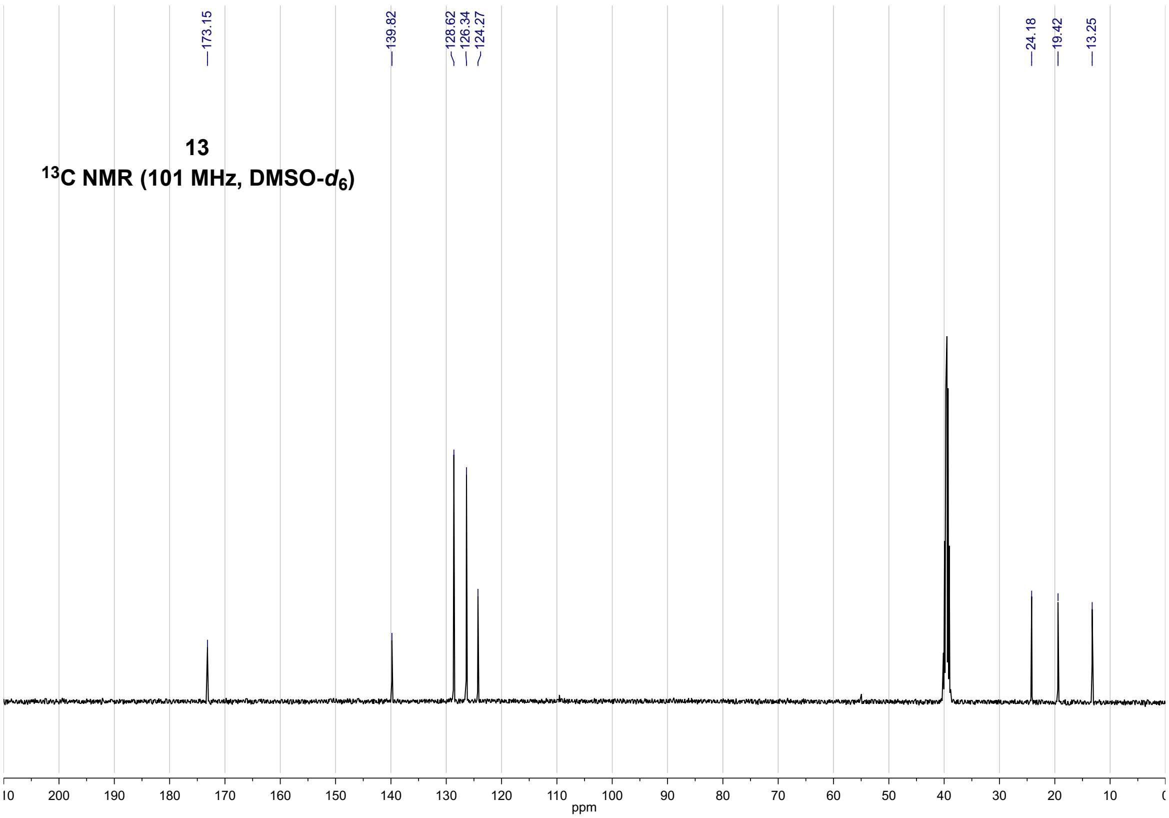
173.15
139.82
128.62
126.34
124.27
24.18
19.42
13.25
13
^{13}C NMR (101 MHz, DMSO-d_6)
ppm

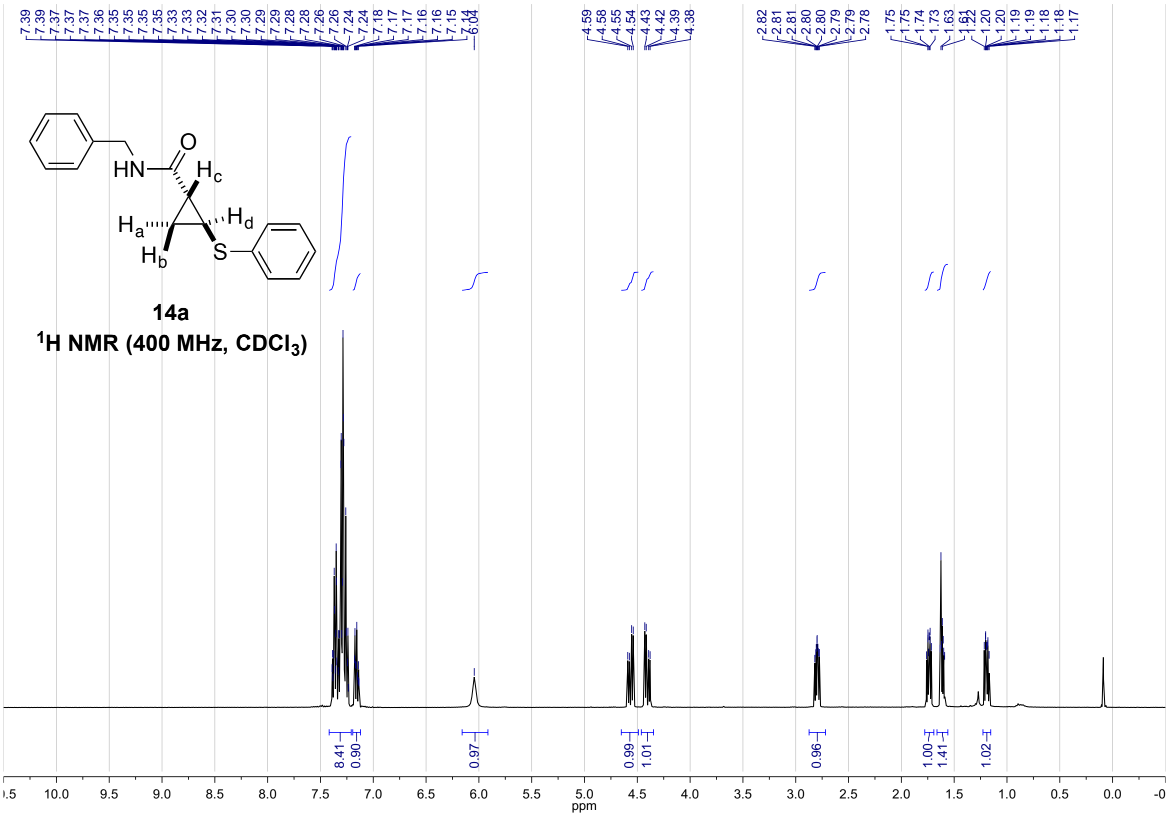
14a
^{1}H NMR (400 MHz, $CDCl_3$)
ppm

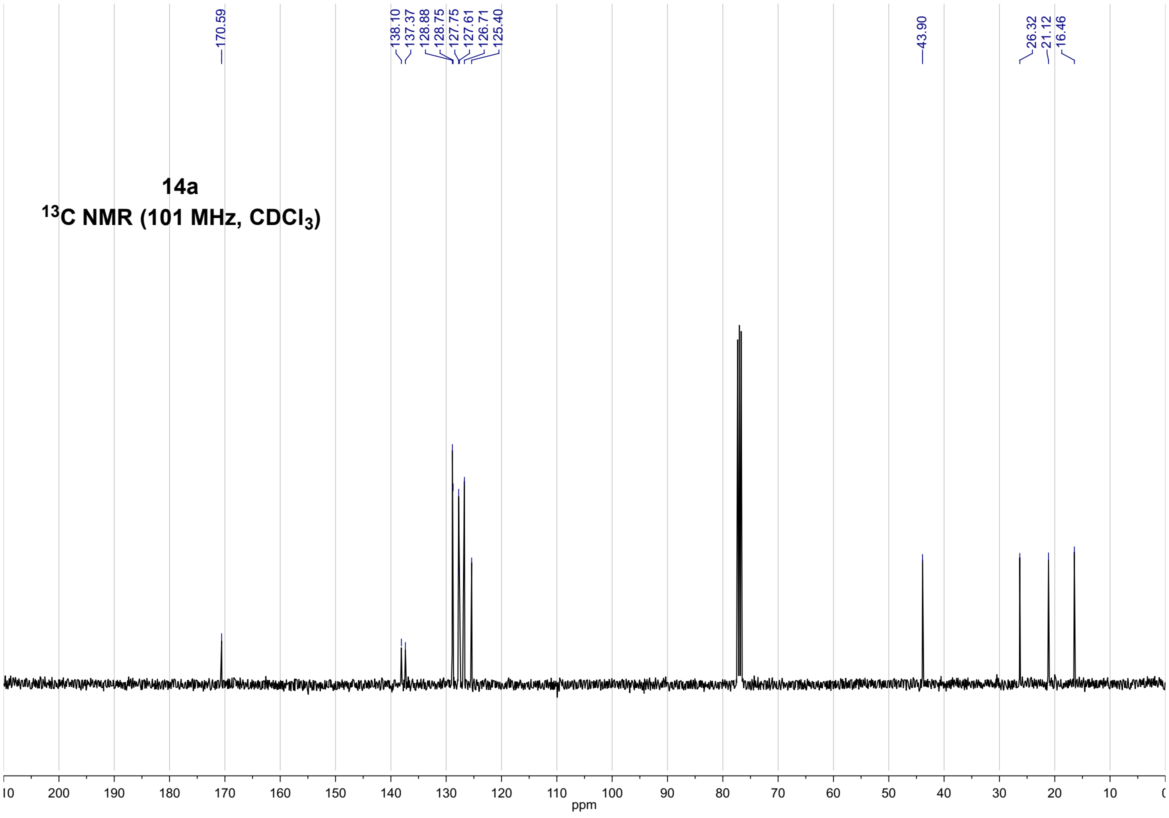
14a
^{13}C NMR (101 MHz, $CDCl_3$)
ppm

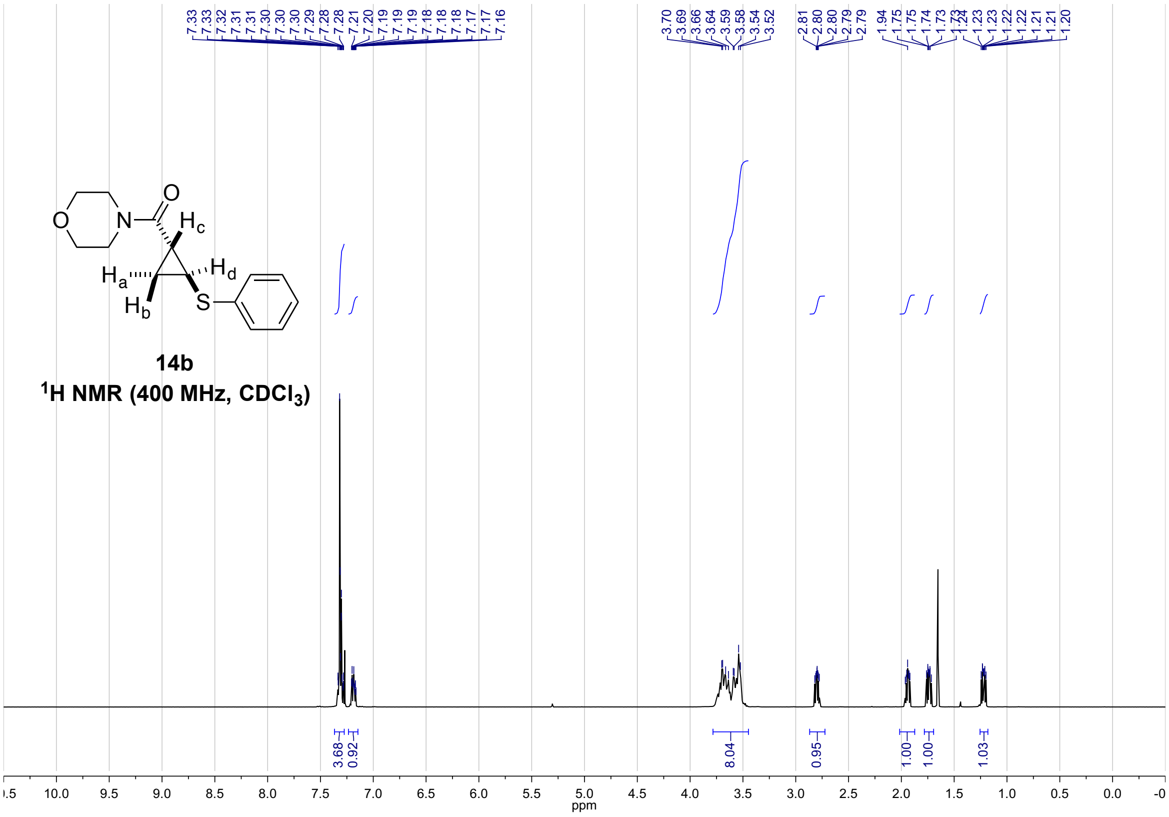

7.33 7.33 7.32 7.31 7.31 7.30 7.30 7.30 7.29 7.28 7.28 7.21 7.20 7.19 7.19 7.19 7.18 7.18 7.18 7.17 7.17 7.16
3.70 3.69 3.66 3.64 3.59 3.58 3.54 3.52
2.81 2.80 2.80 2.79 2.79
1.94 1.75 1.75 1.74 1.73 1.73 1.24 1.23 1.23 1.22 1.22 1.21 1.21 1.20
O
N
H_c
H_a
H_d
H_b
S
14b
1H NMR (400 MHz, CDCl3)
3.68
0.92
8.04
0.95
1.00
1.00
1.03
ppm

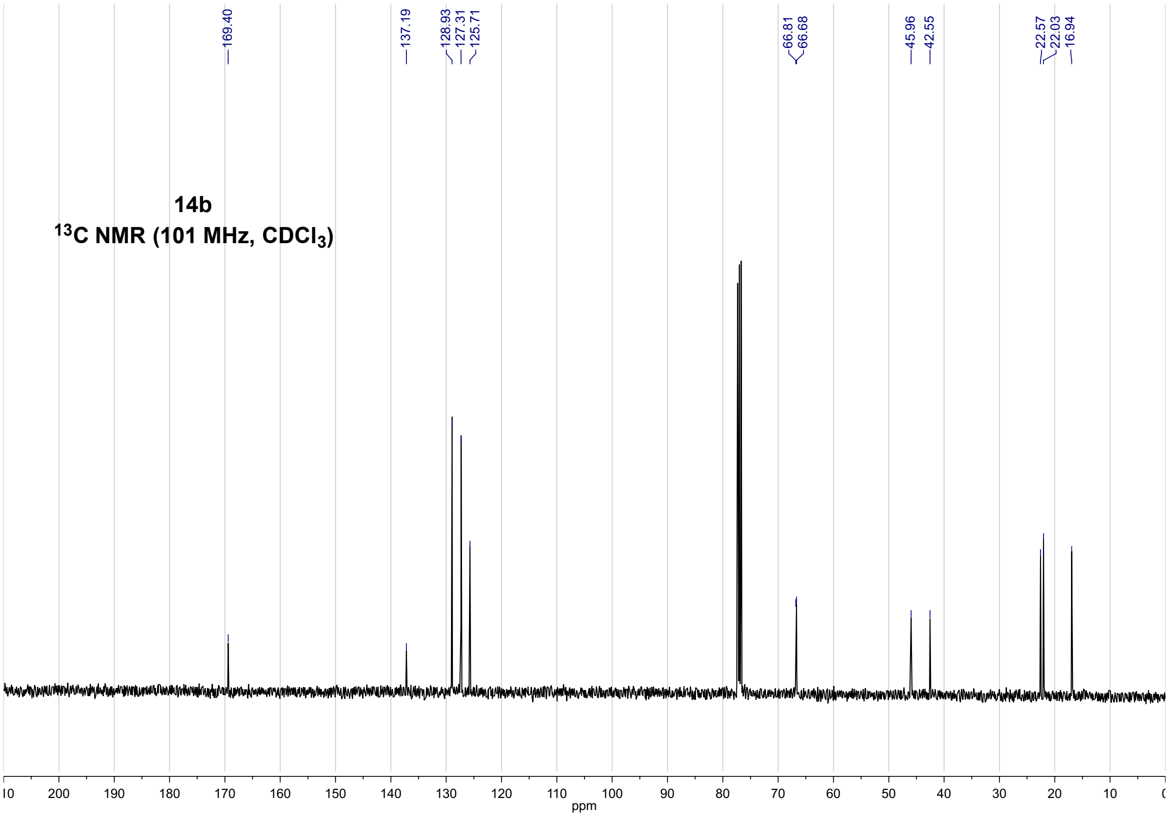

169.40
137.19
128.93 127.31 125.71
66.81 66.68
45.96 42.55
22.57 22.03 16.94
14b
13C NMR (101 MHz, CDCl3)
ppm

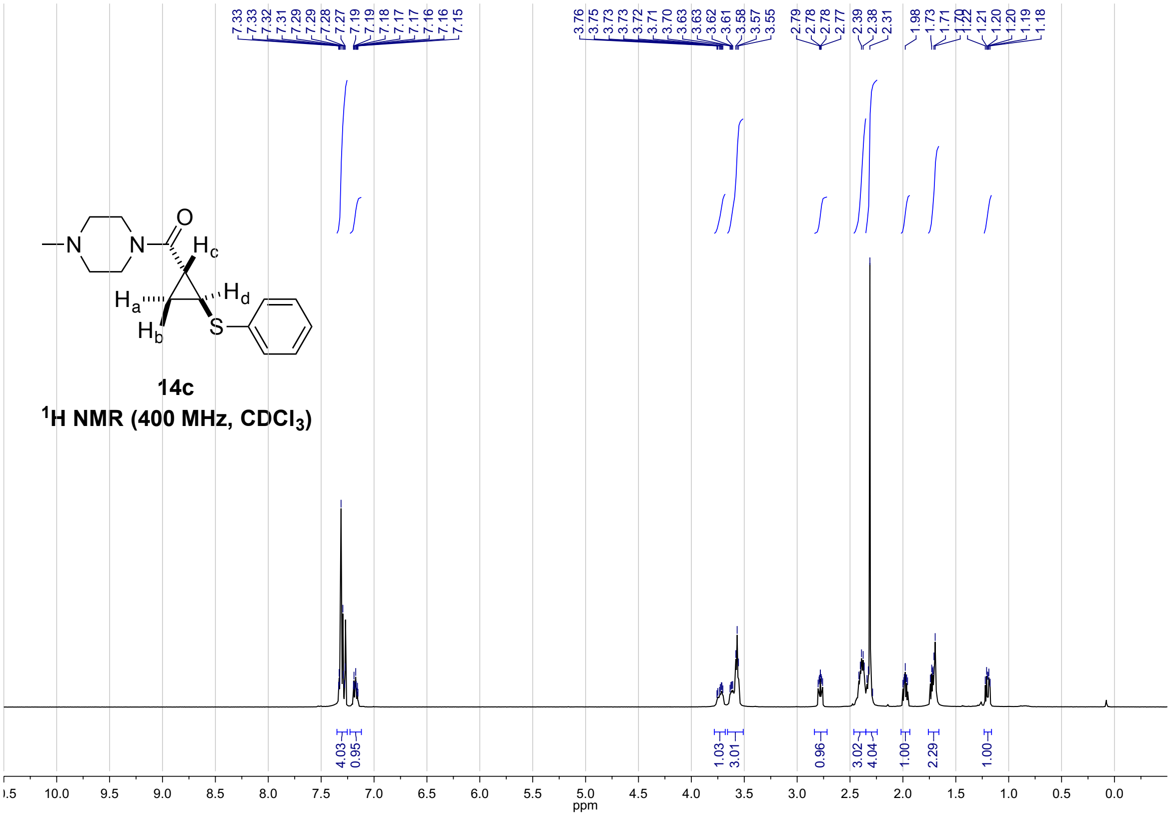
7.33
7.33
7.32
7.31
7.29
7.29
7.28
7.27
7.19
7.19
7.18
7.17
7.17
7.16
7.16
7.15
3.76
3.75
3.73
3.73
3.72
3.71
3.70
3.63
3.62
3.61
3.58
3.57
3.55
2.79
2.78
2.78
2.77
2.39
2.38
2.31
1.98
1.73
1.71
1.21
1.20
1.20
1.19
1.18
O
N
N
H_c
H_a
H_d
H_b
S
14c
^{1}H NMR (400 MHz, $CDCl_3$)
4.03
0.95
1.03
3.01
0.96
3.02
4.04
1.00
2.29
1.00
ppm

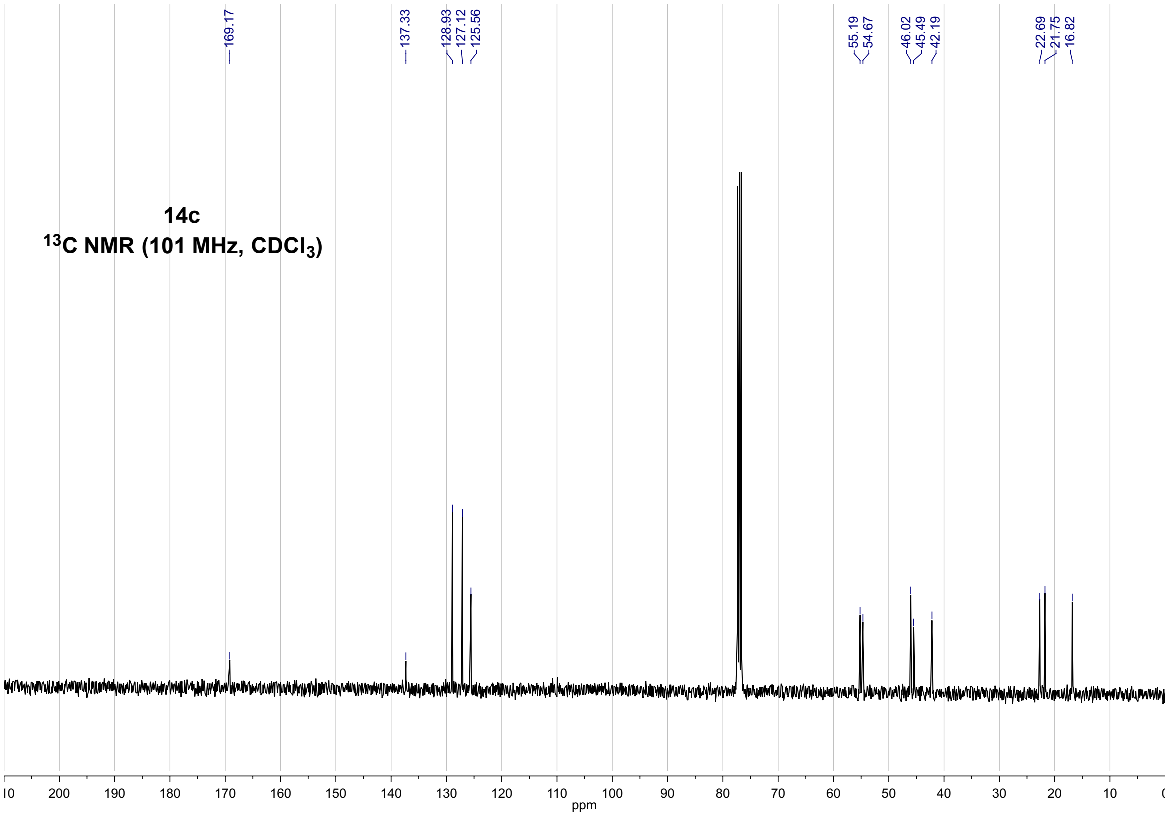
169.17
137.33
128.93
127.12
125.56
55.19
54.67
46.02
45.49
42.19
22.69
21.75
16.82
14c
^{13}C NMR (101 MHz, $CDCl_3$)
ppm

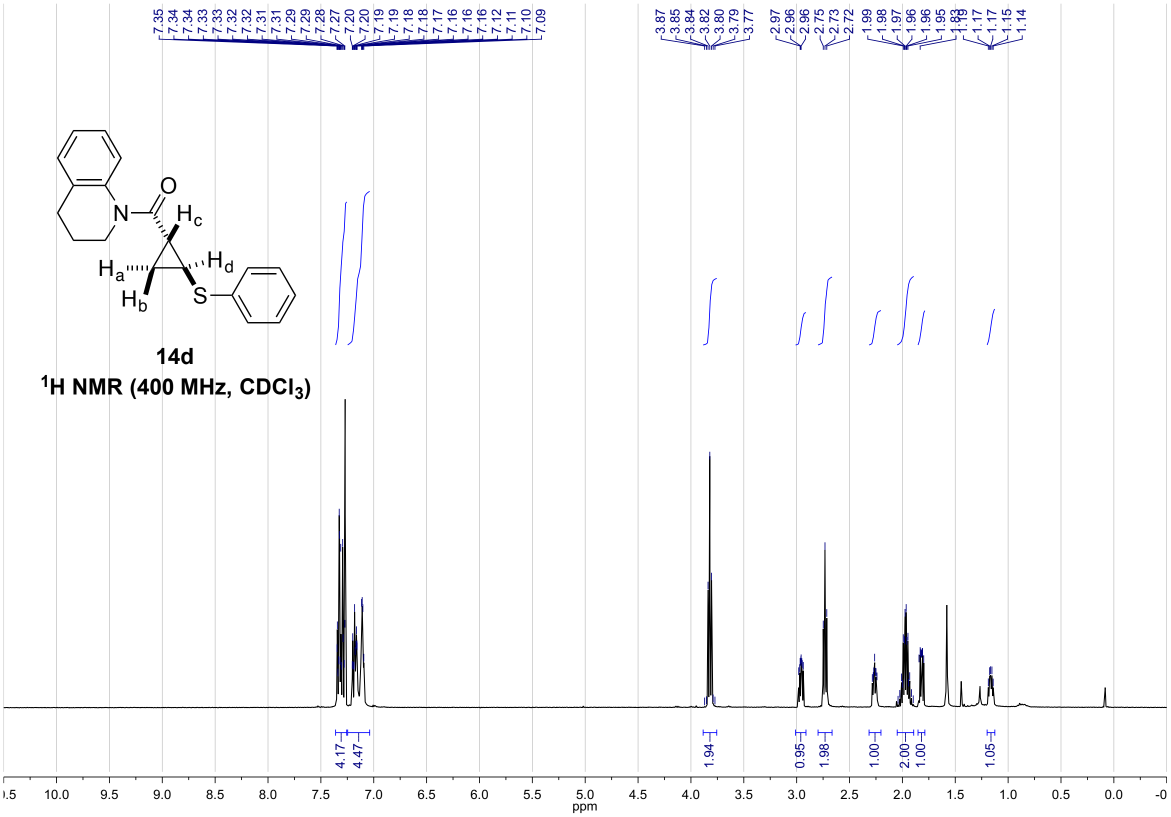
7.35 7.34 7.34 7.33 7.33 7.32 7.32 7.31 7.29 7.29 7.28 7.27 7.20 7.20 7.19 7.18 7.18 7.17 7.16 7.16 7.12 7.11 7.10 7.09
3.87 3.85 3.84 3.82 3.80 3.79 3.77
2.97 2.96 2.96 2.75 2.73 2.72
1.99 1.98 1.97 1.96 1.96 1.95 1.83 1.19 1.17 1.15 1.14
O
N
Hc
Ha
Hd
Hb
S
14d
1H NMR (400 MHz, CDCl3)
4.17 4.47 1.94 0.95 1.98 1.00 2.00 1.00 1.05
ppm

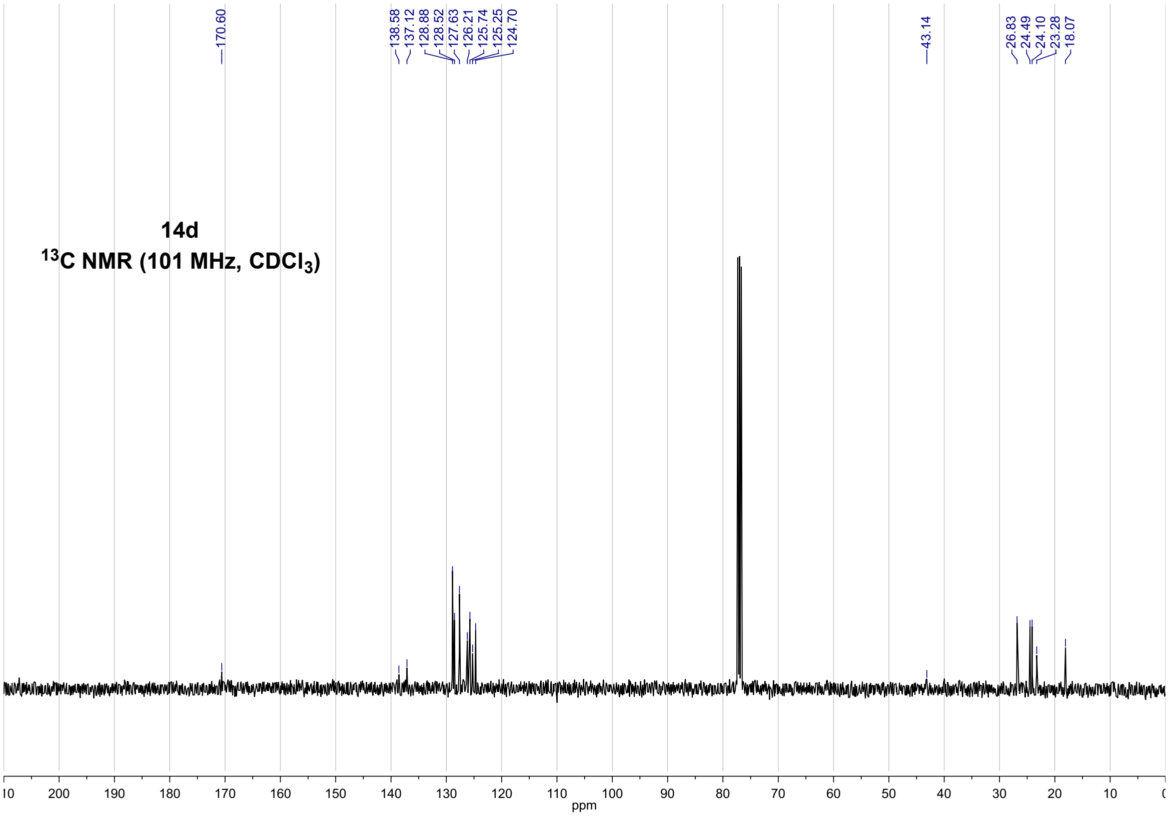
170.60
138.58 137.12 128.88 128.52 127.63 126.21 125.74 125.25 124.70
43.14
26.83 24.49 24.10 23.28 18.07
14d
13C NMR (101 MHz, CDCl3)
ppm

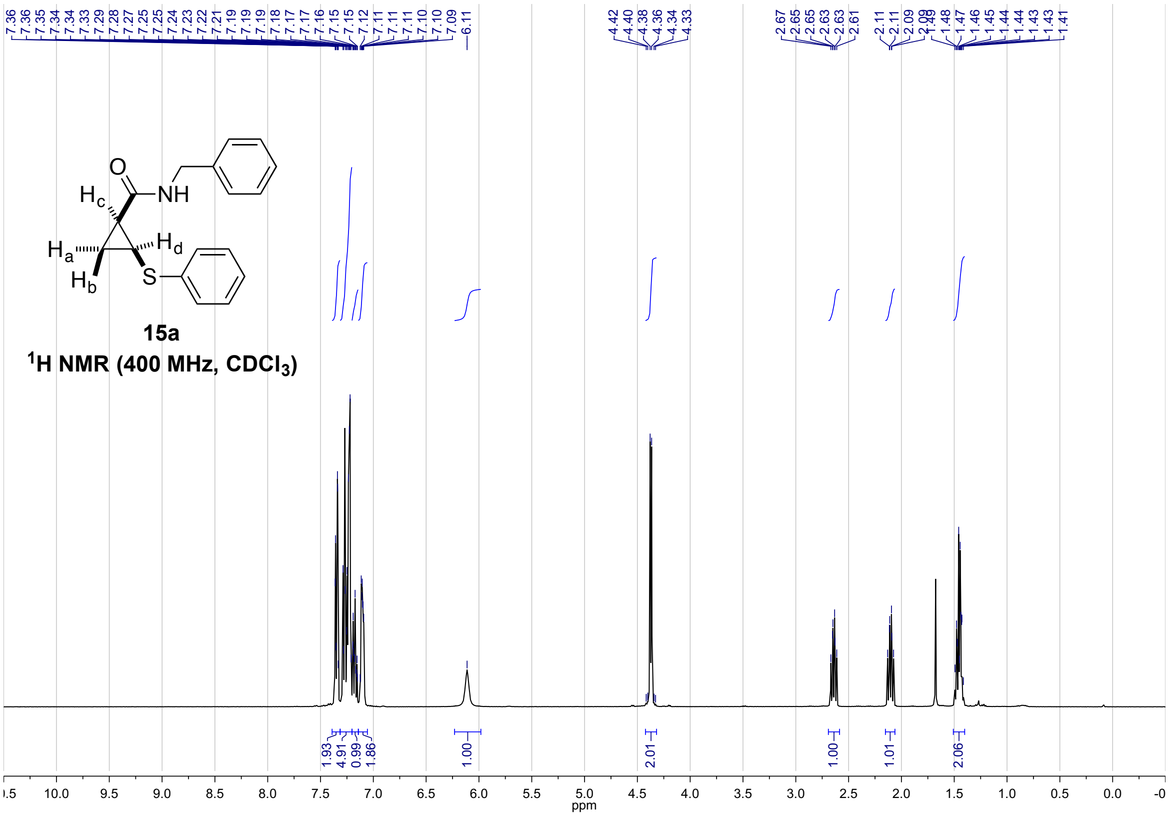

15a
^{1}H NMR (400 MHz, $CDCl_3$)

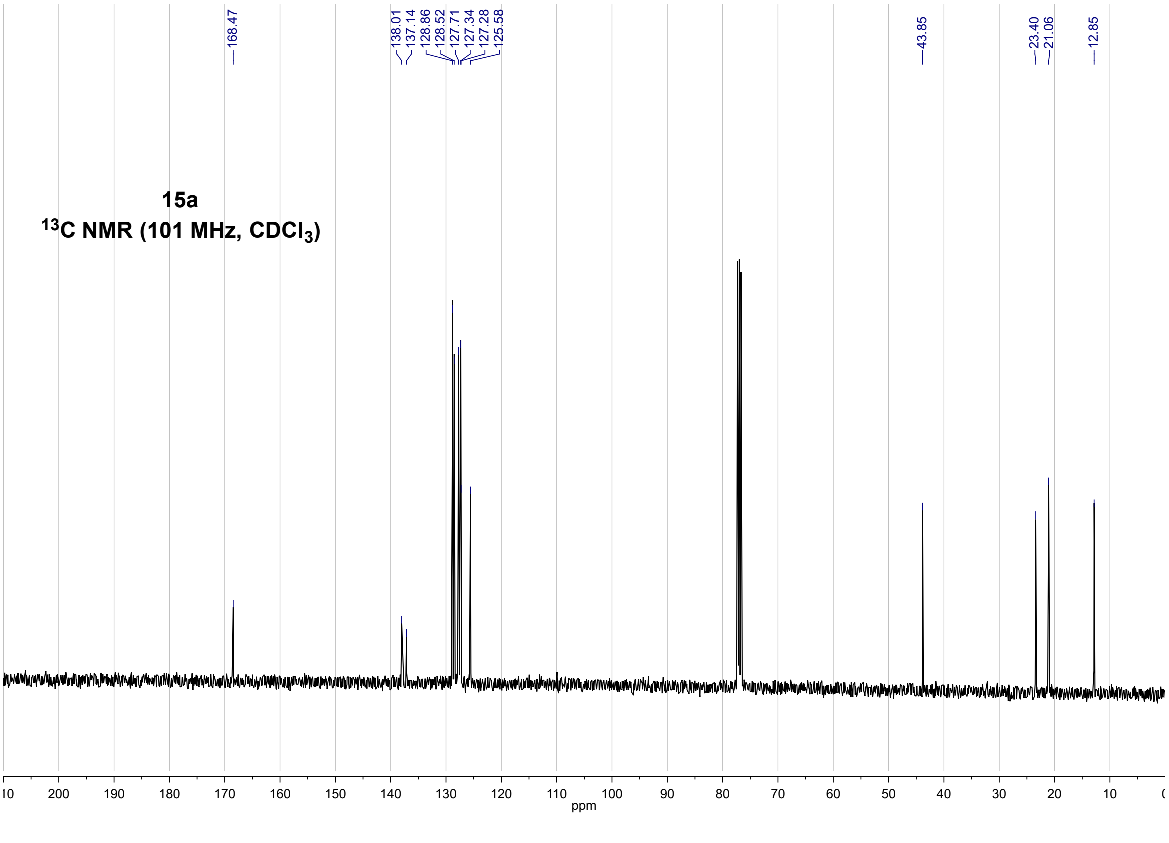

15a
^{13}C NMR (101 MHz, $CDCl_3$)

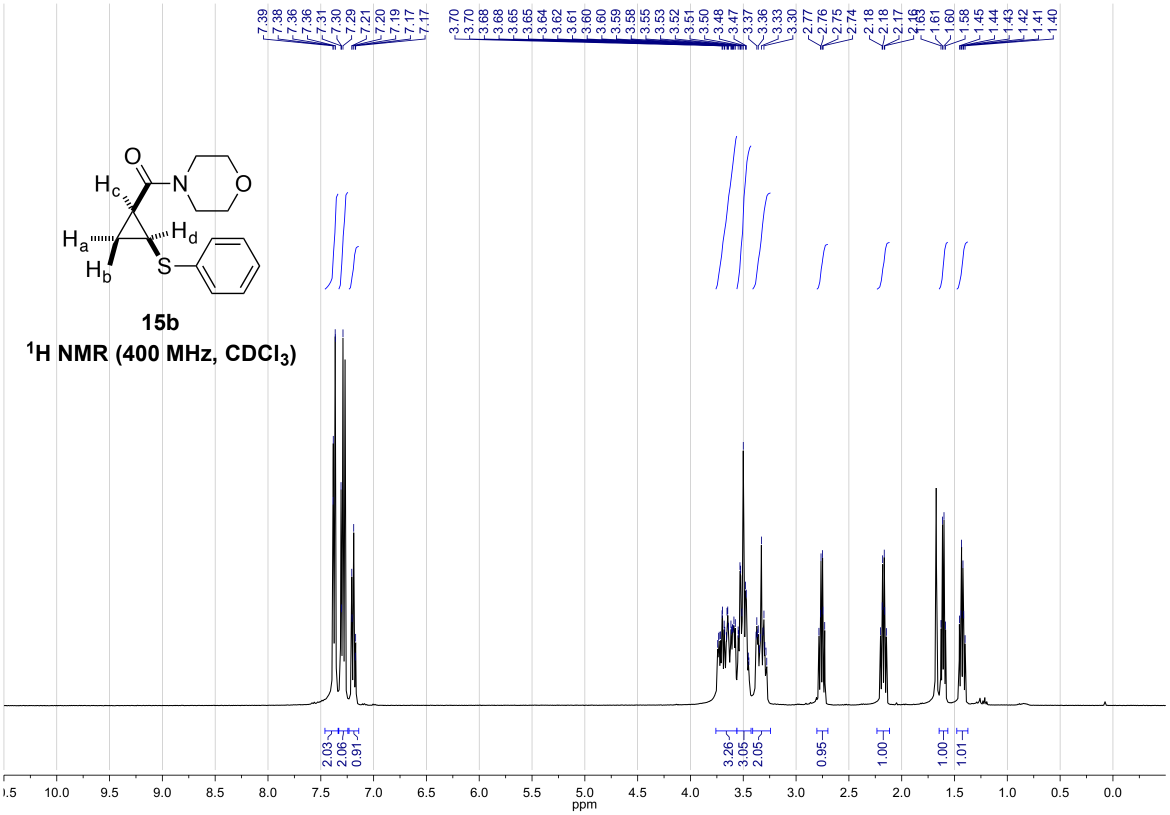
15b
^{1}H NMR (400 MHz, CDCl$_3$)
7.39 7.38 7.36 7.36 7.31 7.30 7.29 7.21 7.20 7.19 7.17 7.17
3.70 3.70 3.68 3.68 3.65 3.64 3.62 3.61 3.60 3.60 3.59 3.58 3.55 3.53 3.52 3.51 3.50 3.48 3.47 3.37 3.36 3.33 3.30
2.77 2.76 2.75 2.74 2.18 2.18 2.17 2.16 1.63 1.61 1.60 1.58 1.45 1.44 1.43 1.42 1.41 1.40
2.03 2.06 0.91 3.26 3.05 2.05 0.95 1.00 1.00 1.01
ppm

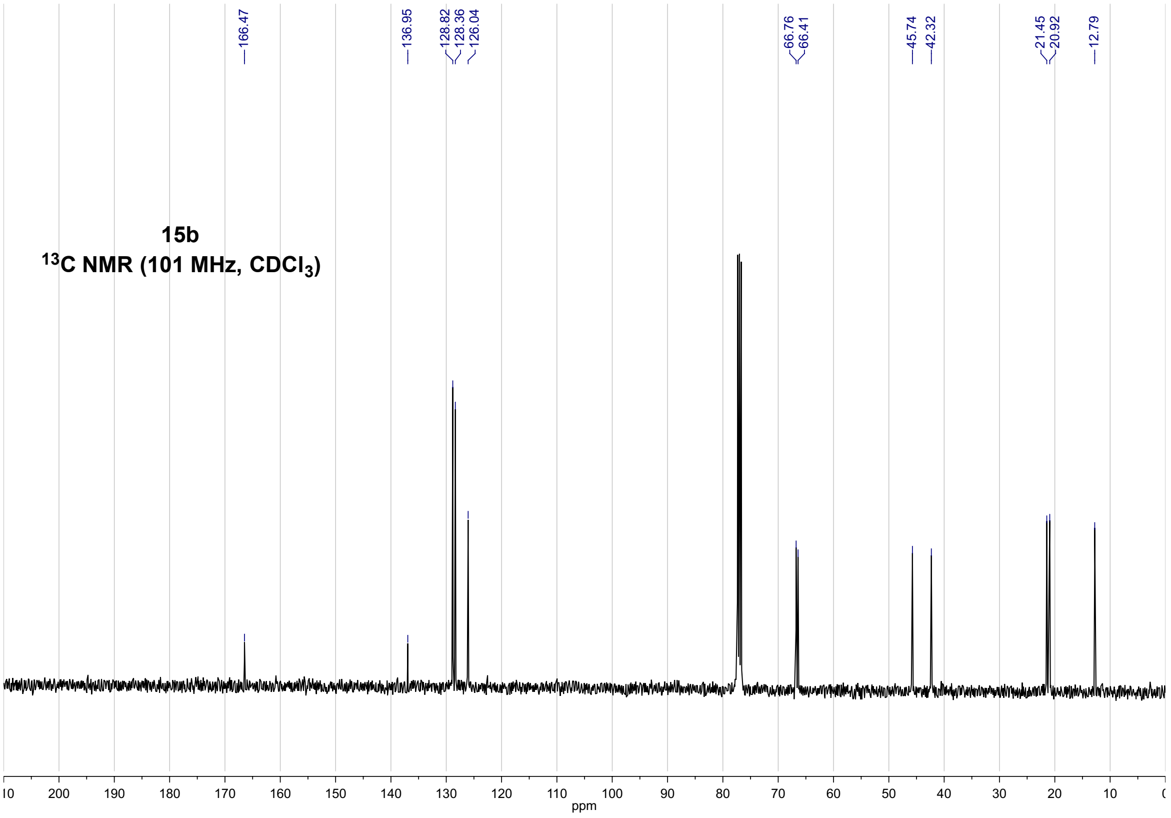
15b
^{13}C NMR (101 MHz, CDCl$_3$)
166.47
136.95
128.82 128.36 126.04
66.76 66.41
45.74
42.32
21.45 20.92
12.79
ppm

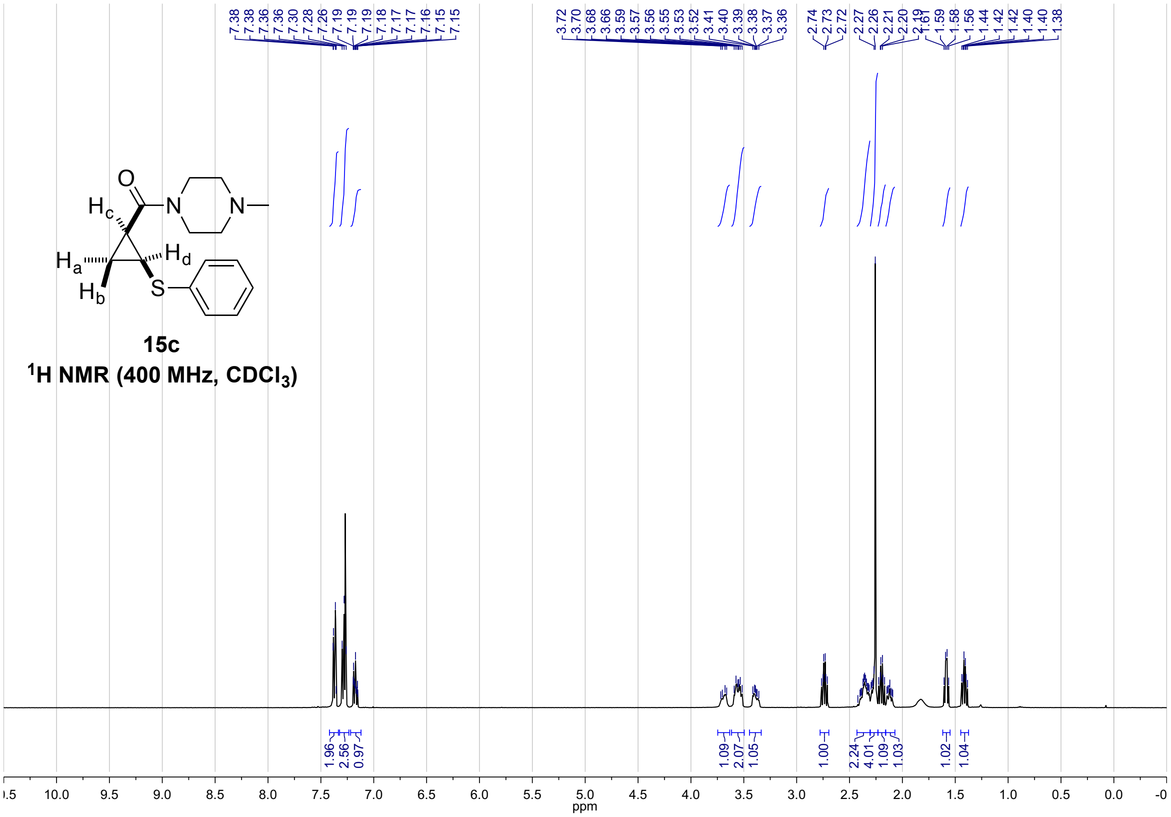
7.38 7.38 7.36 7.30 7.28 7.26 7.19 7.19 7.19 7.18 7.17 7.17 7.16 7.15 7.15
3.72 3.70 3.68 3.66 3.59 3.57 3.56 3.55 3.53 3.52 3.41 3.40 3.39 3.38 3.37 3.36
2.74 2.73 2.72 2.27 2.26 2.21 2.20 2.19 1.61 1.59 1.58 1.56 1.44 1.42 1.42 1.40 1.40 1.38
O
N
N
H_c
H_a
H_d
H_b
S
15c
^{1}H NMR (400 MHz, $CDCl_3$)
1.96 2.56 0.97 1.09 2.07 1.05 1.00 2.24 4.01 1.09 1.03 1.02 1.04
ppm

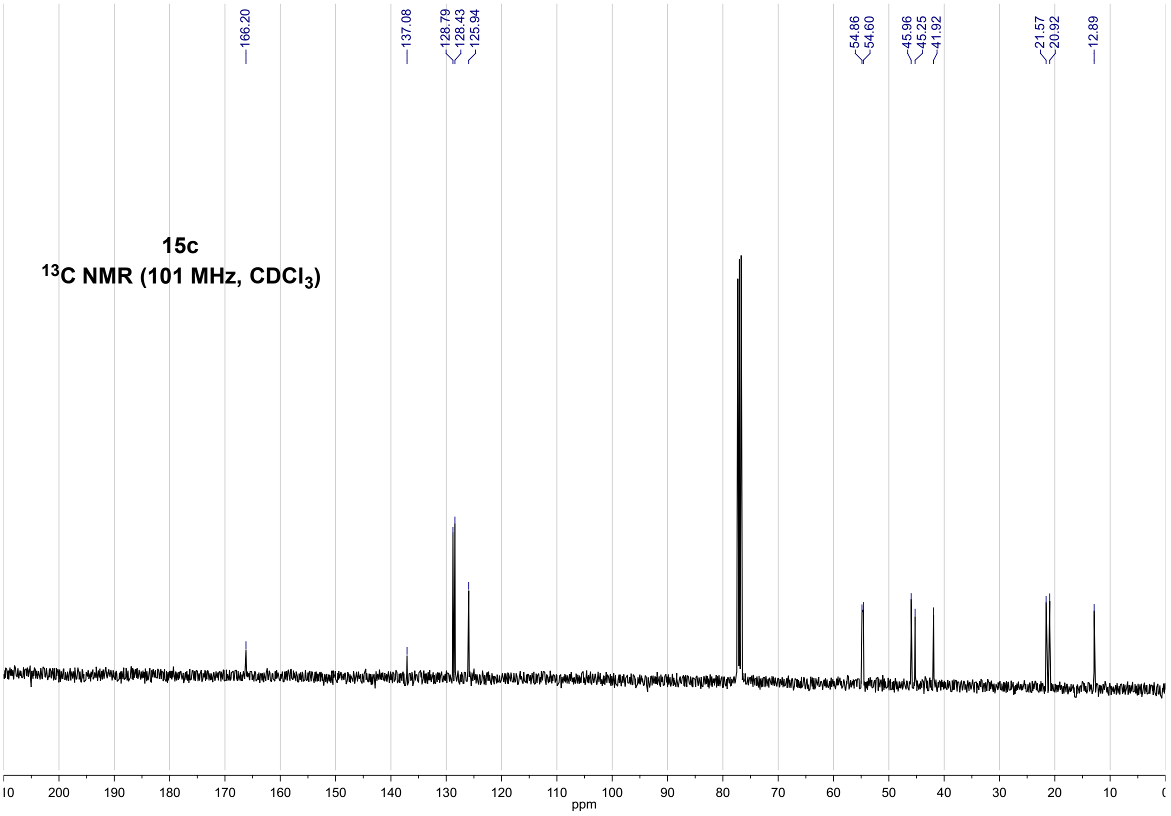
166.20
137.08
128.79 128.43 125.94
54.86 54.60
45.96 45.25 41.92
21.57 20.92
12.89
15c
^{13}C NMR (101 MHz, $CDCl_3$)
ppm

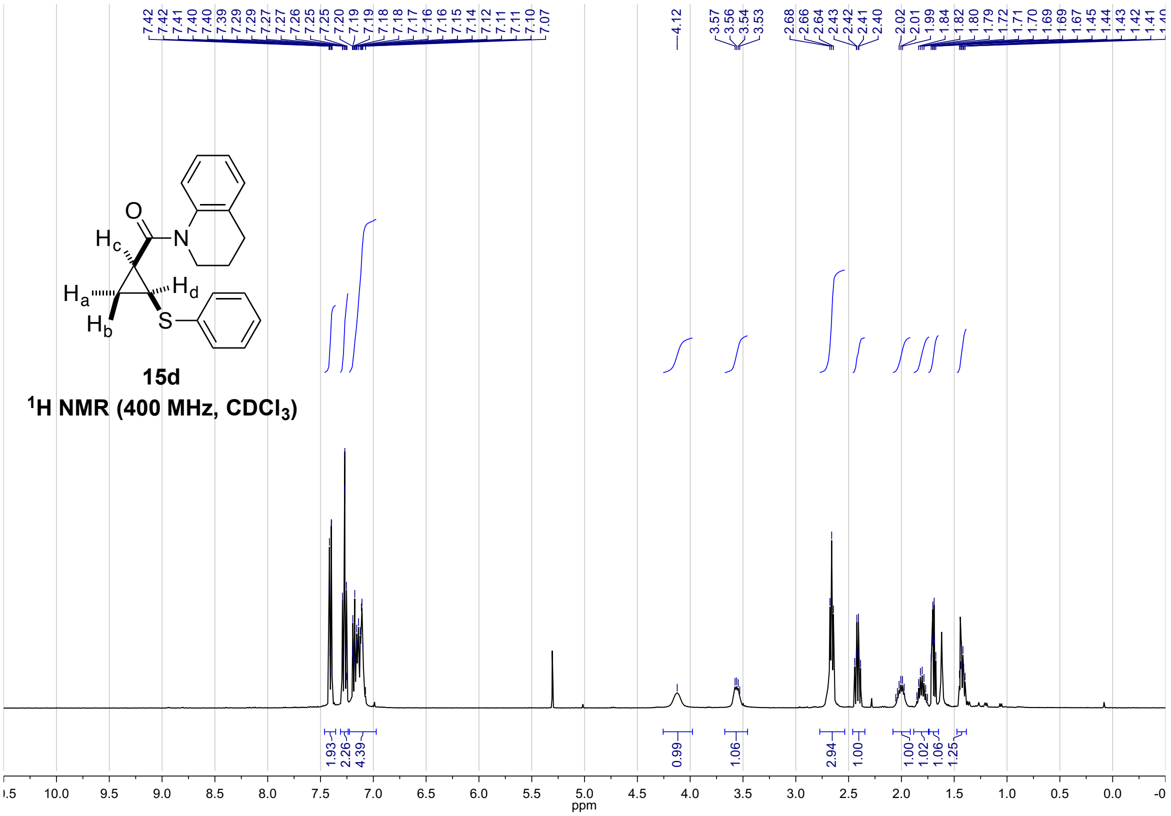

15d

^{1}H NMR (400 MHz, $CDCl_3$)

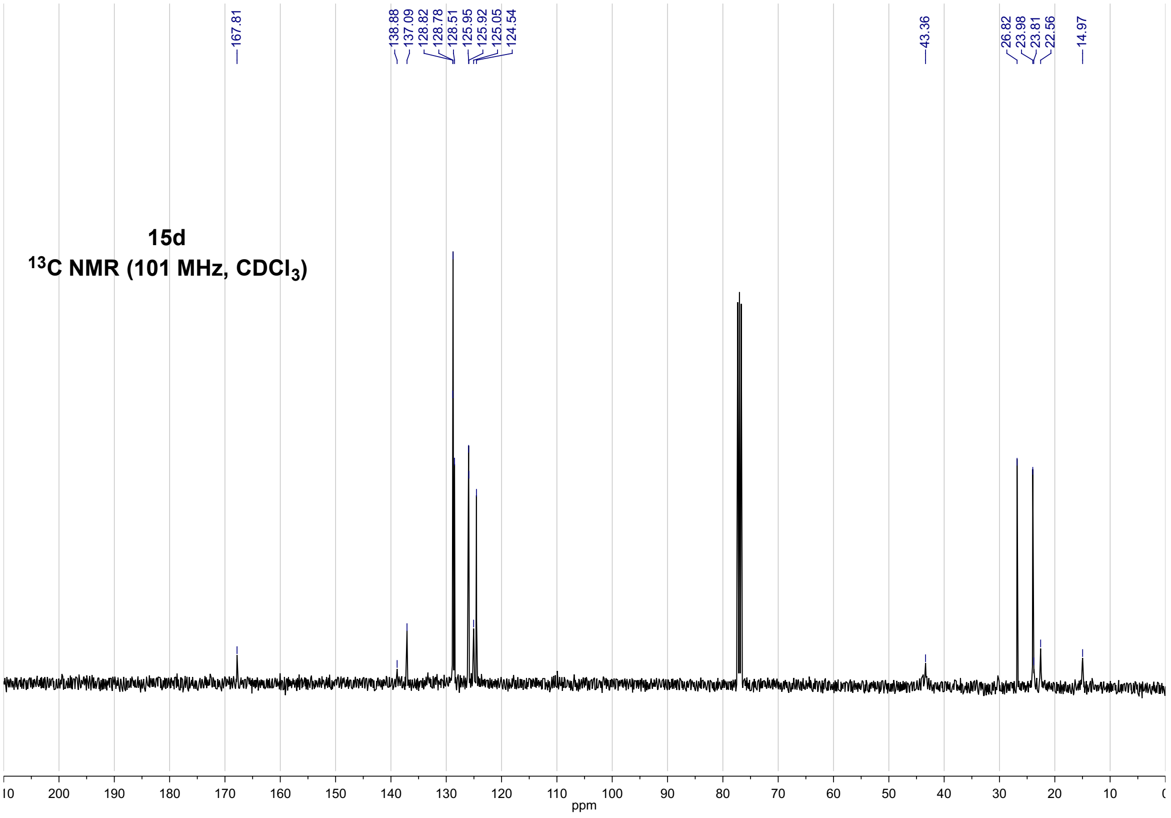

15d

^{13}C NMR (101 MHz, $CDCl_3$)

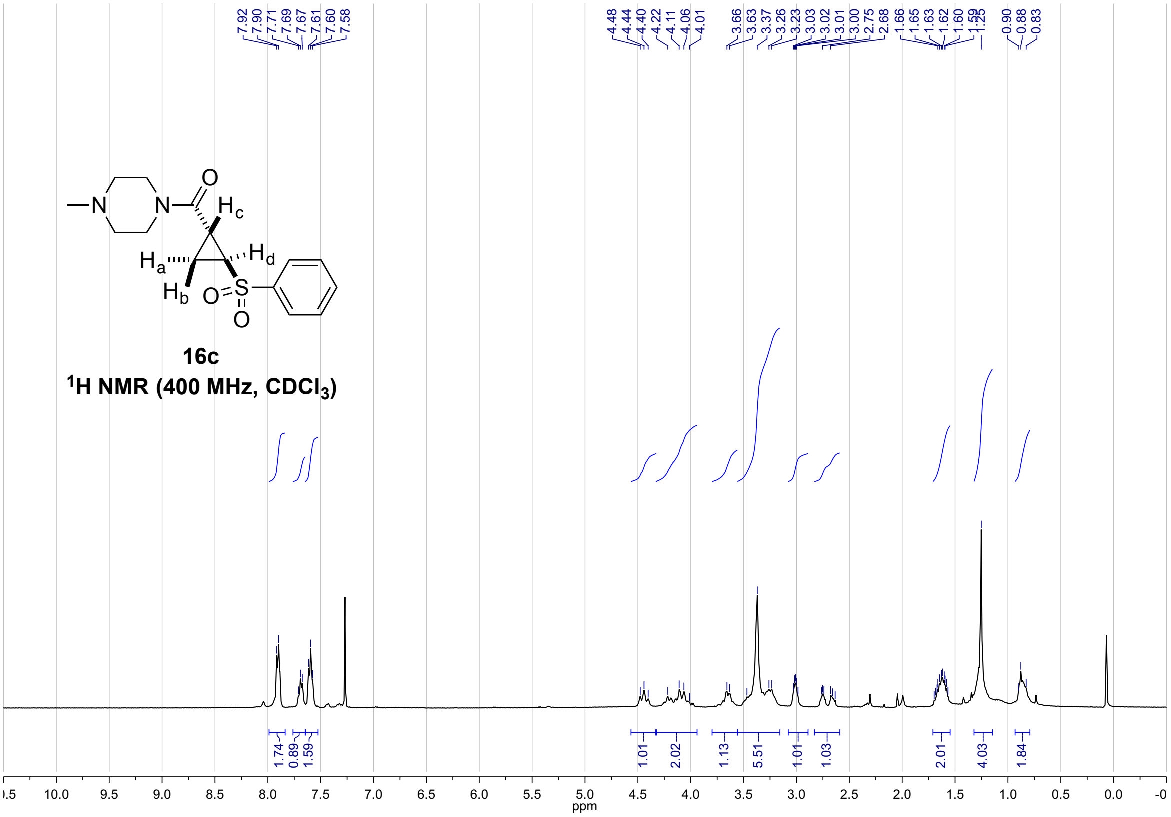
16c
1H NMR (400 MHz, CDCl3)
7.92 7.90 7.71 7.69 7.67 7.61 7.60 7.58
4.48 4.44 4.40 4.22 4.11 4.06 4.01
3.66 3.63 3.37 3.26 3.23 3.03 3.02 3.01 3.00 2.75 2.68
1.66 1.65 1.63 1.62 1.60 1.59 1.25 0.90 0.88 0.83
1.74 0.89 1.59 1.01 2.02 1.13 5.51 1.01 1.03 2.01 4.03 1.84
ppm

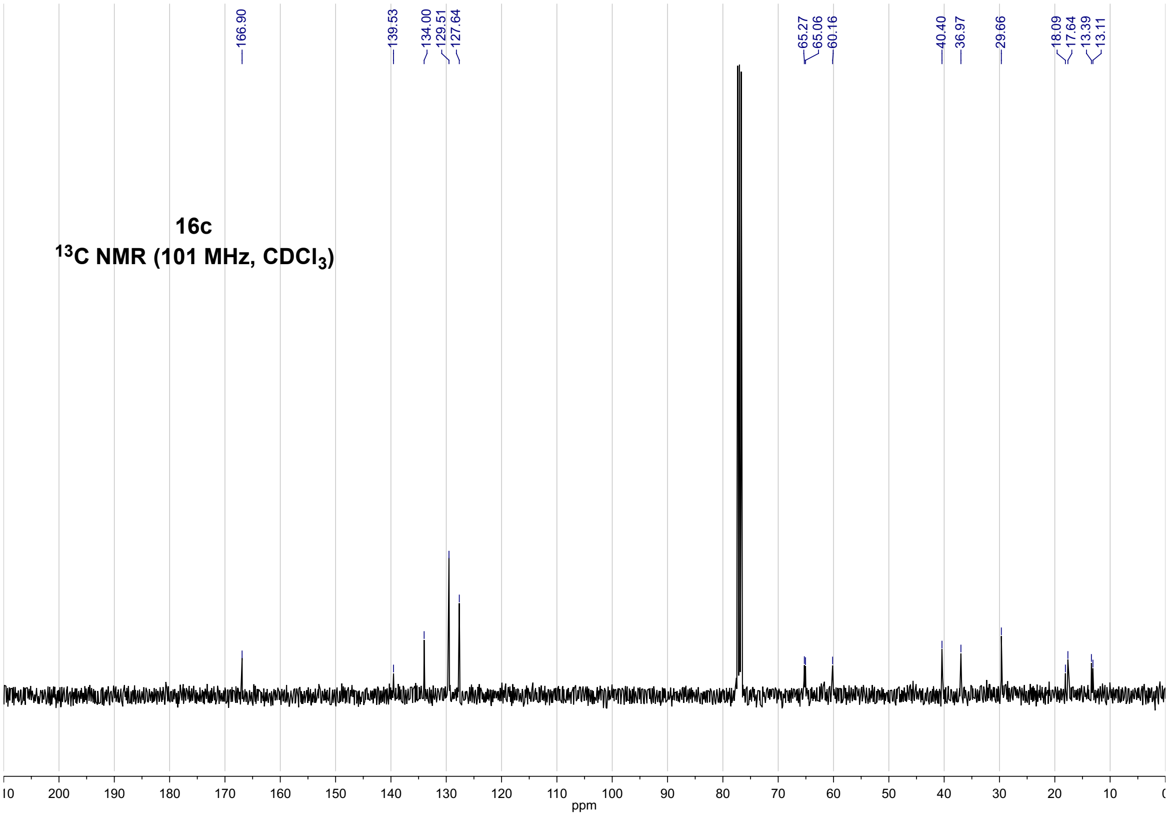
16c
13C NMR (101 MHz, CDCl3)
166.90
139.53 134.00 129.51 127.64
65.27 65.06 60.16
40.40 36.97 29.66
18.09 17.64 13.39 13.11
ppm

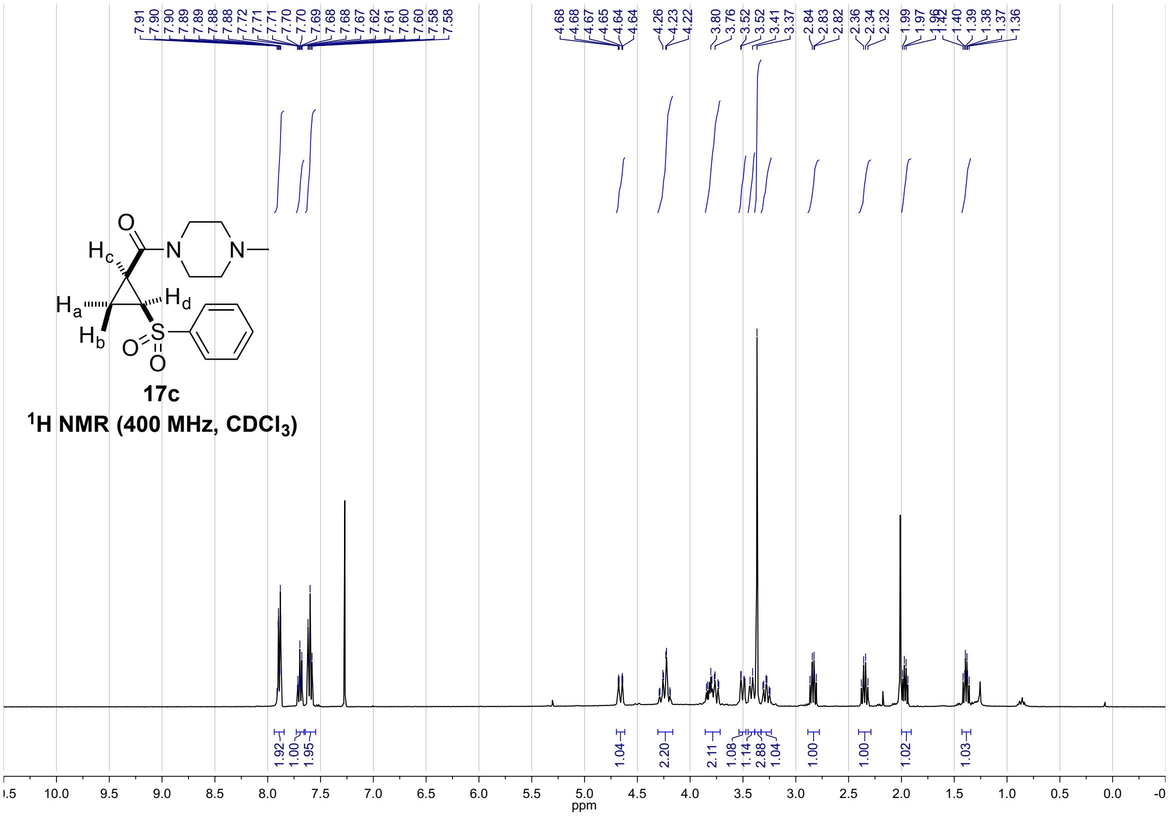

17c
1H NMR (400 MHz, CDCl3)
7.91 7.90 7.90 7.89 7.89 7.88 7.88 7.72 7.71 7.71 7.70 7.70 7.69 7.68 7.68 7.67 7.62 7.61 7.60 7.60 7.58 7.58
4.68 4.68 4.67 4.65 4.64 4.64 4.26 4.23 4.22 3.80 3.76 3.52 3.52 3.41 3.37 2.84 2.83 2.82 2.36 2.34 2.32 1.99 1.97 1.42 1.40 1.39 1.38 1.37 1.36
1.92 1.00 1.95 1.04 2.20 2.11 1.08 1.14 2.88 1.04 1.00 1.00 1.02 1.03
ppm

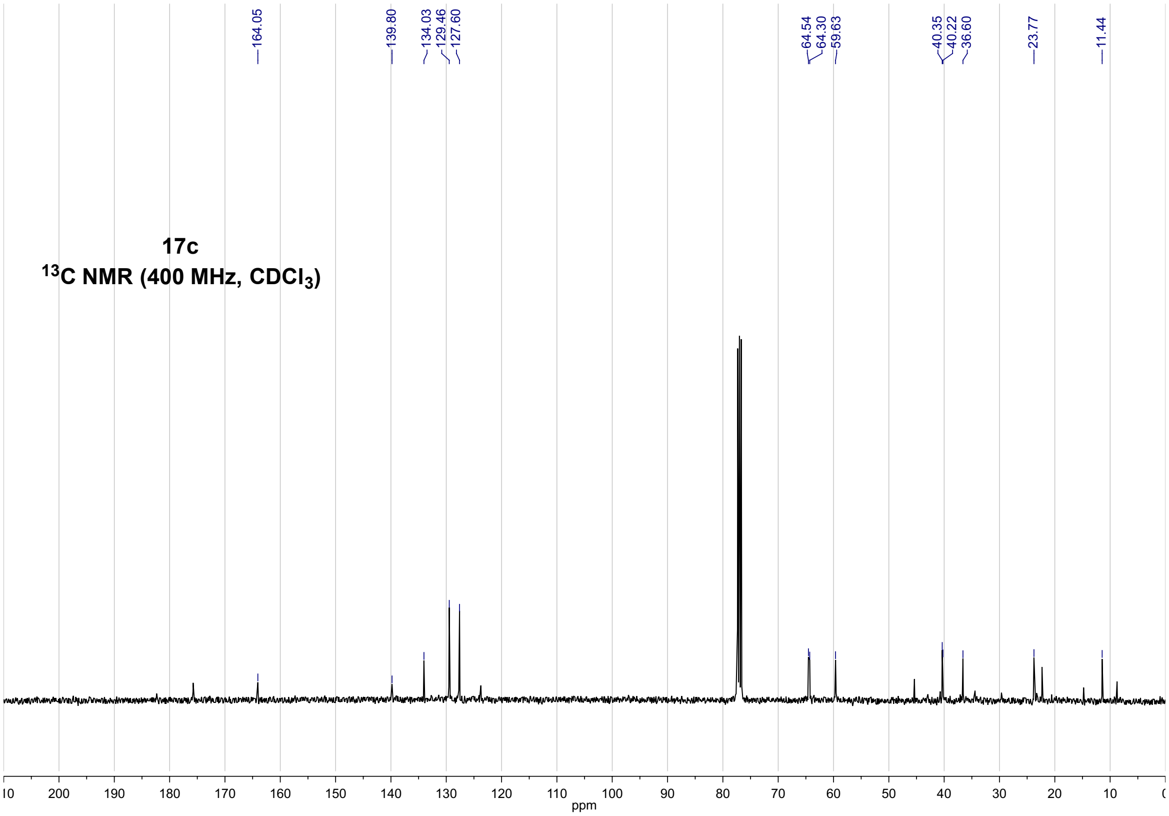

17c
13C NMR (400 MHz, CDCl3)
164.05 139.80 134.03 129.46 127.60 64.54 64.30 59.63 40.35 40.22 36.60 23.77 11.44
ppm

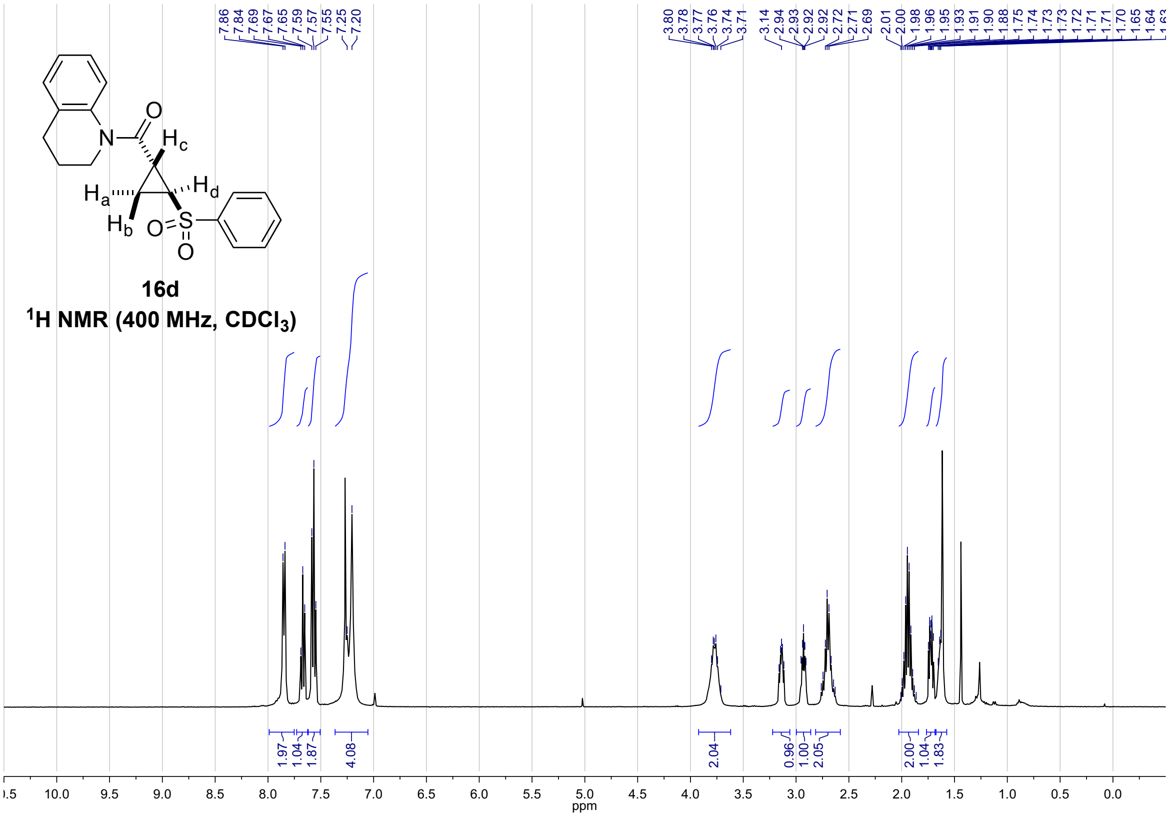

16d

^{1}H NMR (400 MHz, $CDCl_3$)

16d

^{13}C NMR (101 MHz, $CDCl_3$)

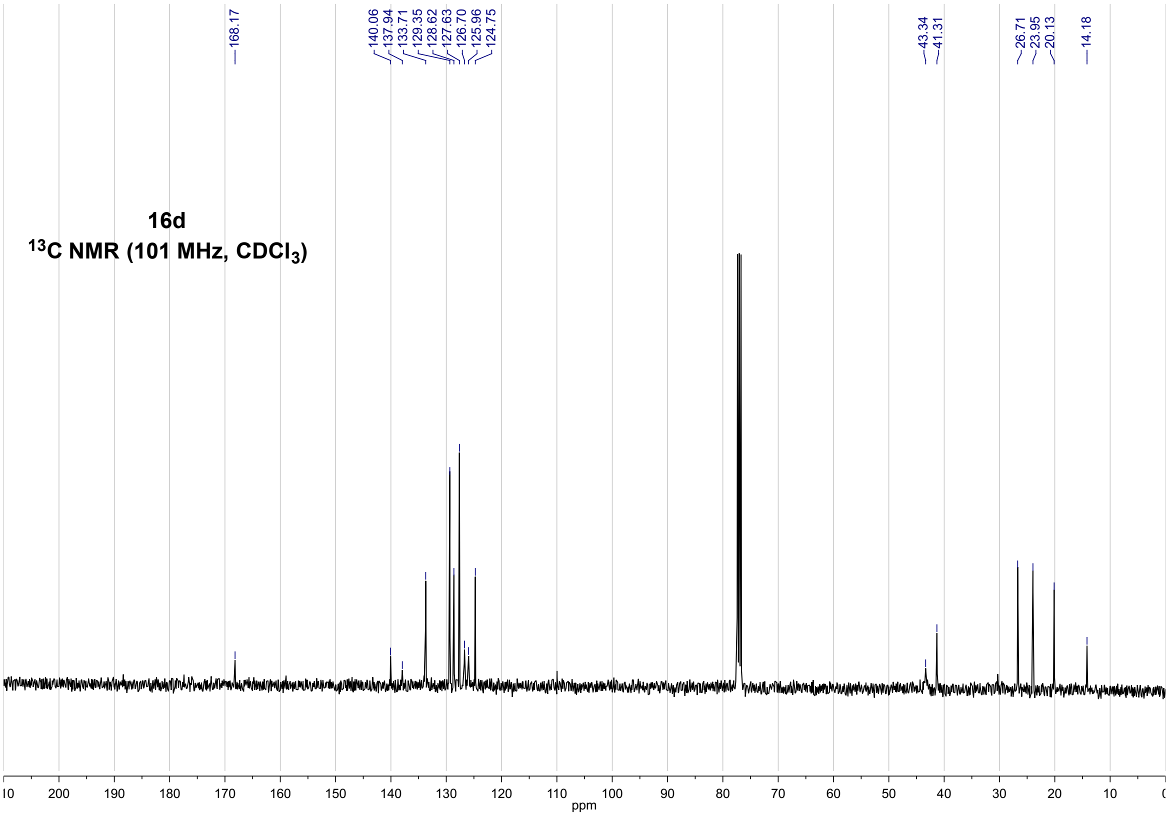

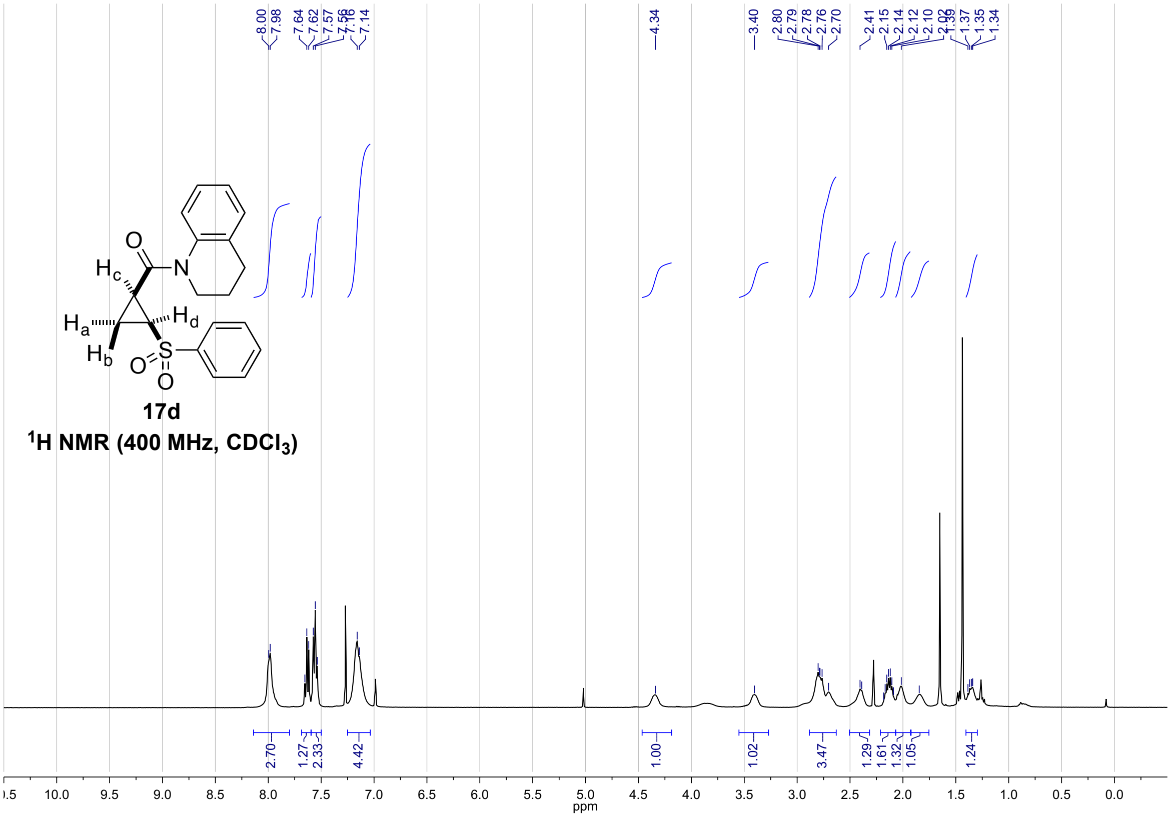
17d
¹H NMR (400 MHz, CDCl3)
ppm

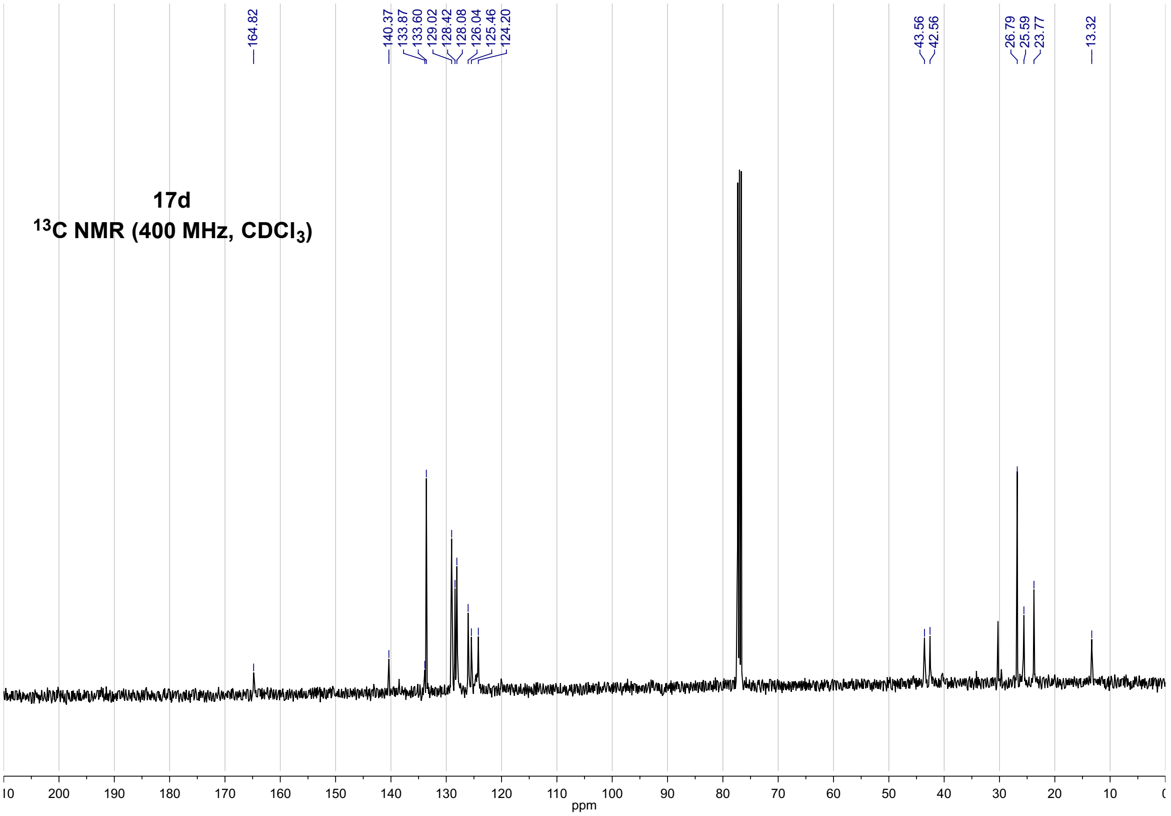
17d
¹³C NMR (400 MHz, CDCl3)
164.82
140.37
133.87
133.60
129.02
128.42
128.08
126.04
125.46
124.20
43.56
42.56
26.79
25.59
23.77
13.32
ppm

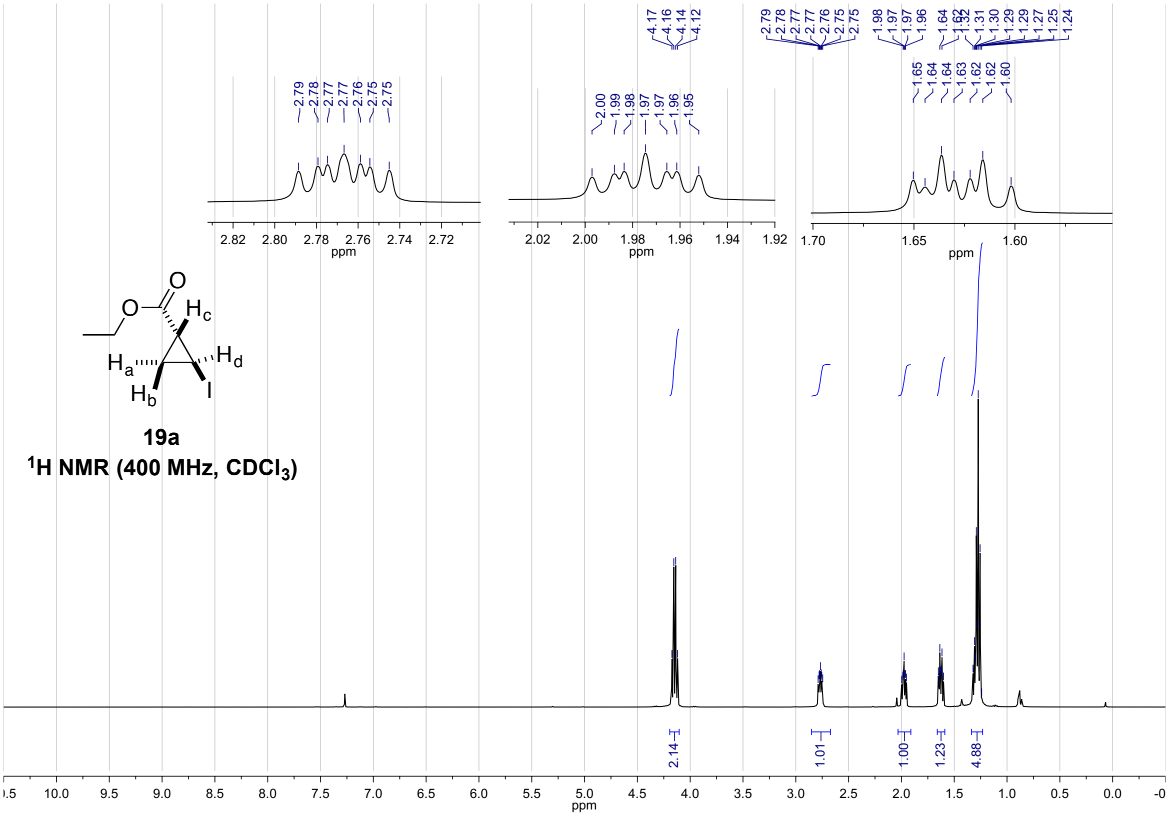

19a

^{1}H NMR (400 MHz, $CDCl_3$)

19a

^{13}C NMR (101 MHz, $CDCl_3$)

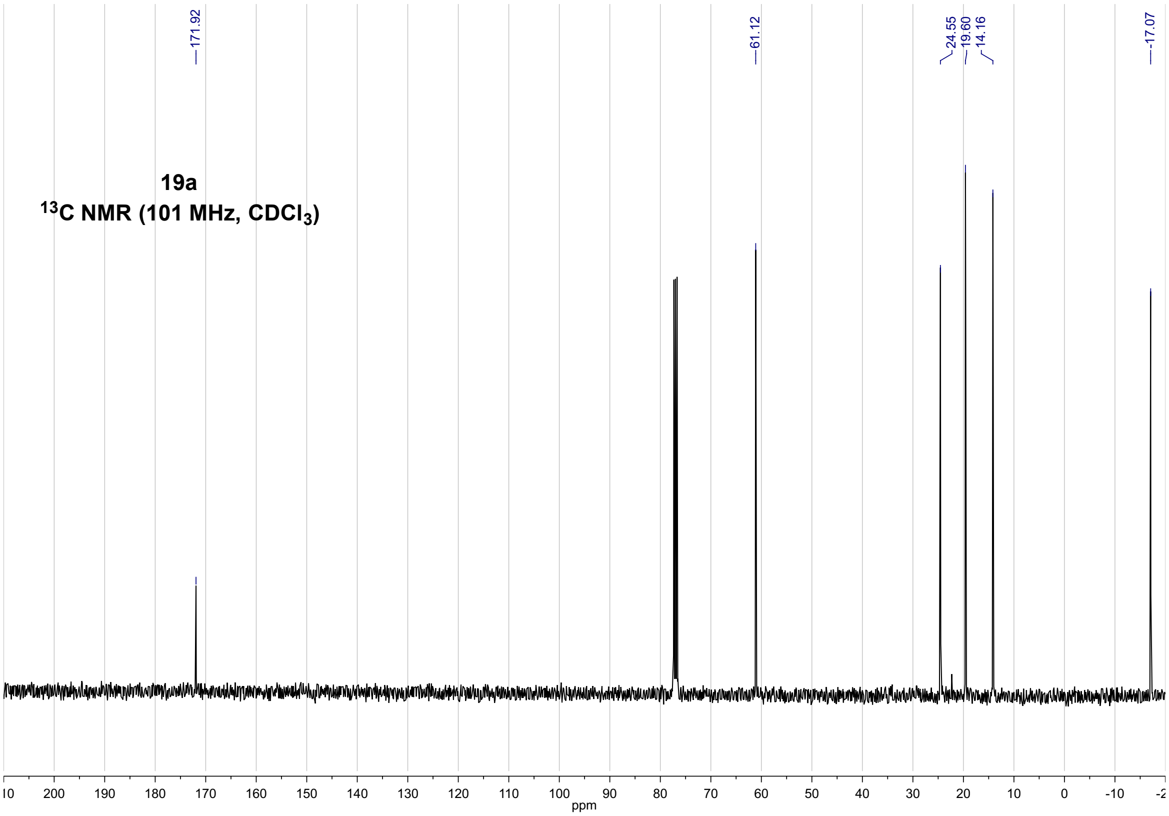

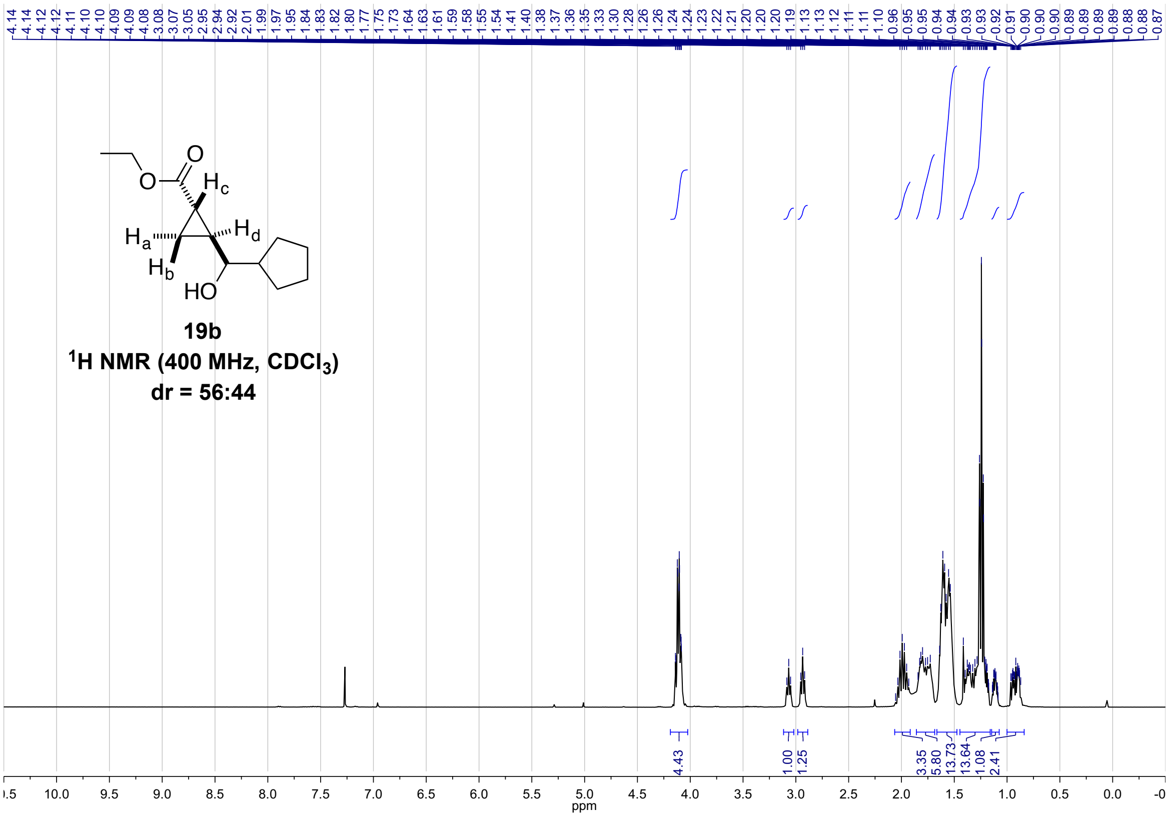
19b
^{1}H NMR (400 MHz, CDCl$_3$)
dr = 56:44
ppm

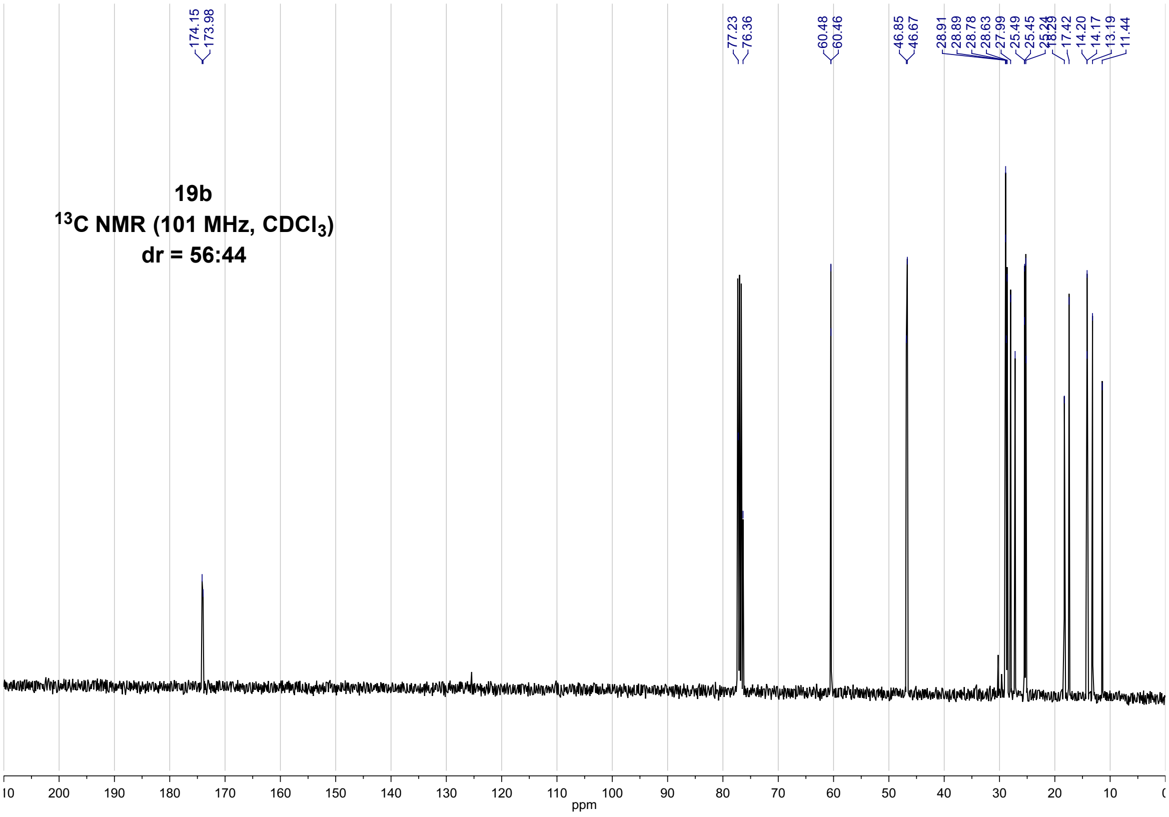
19b
^{13}C NMR (101 MHz, CDCl$_3$)
dr = 56:44
ppm

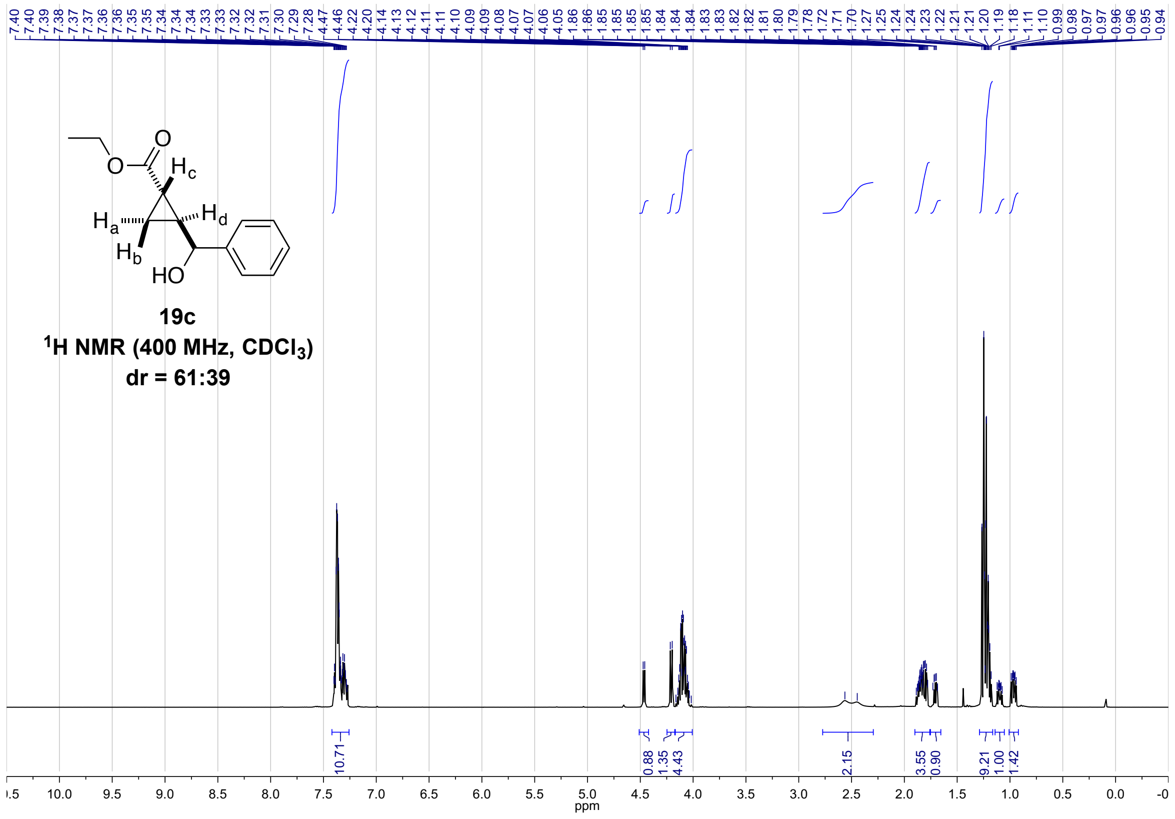

19c
^{1}H NMR (400 MHz, $CDCl_3$)
dr = 61:39

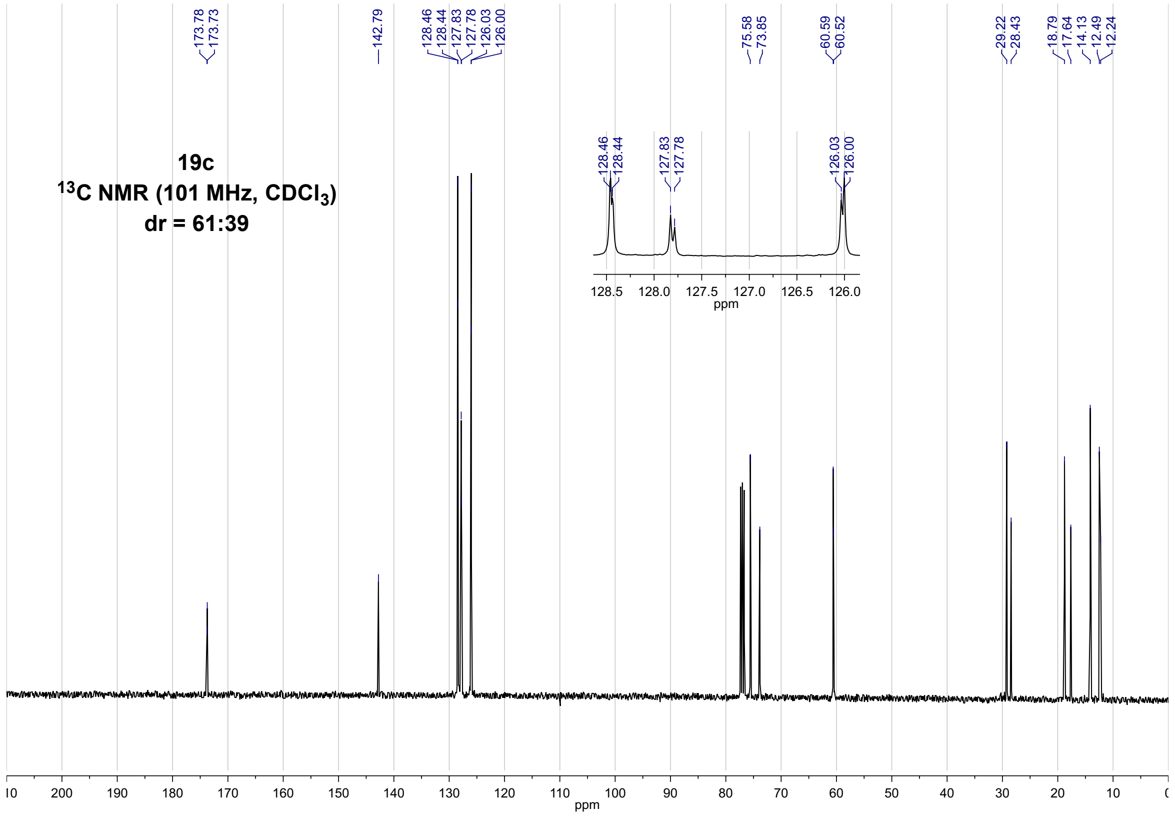

19c
^{13}C NMR (101 MHz, $CDCl_3$)
dr = 61:39

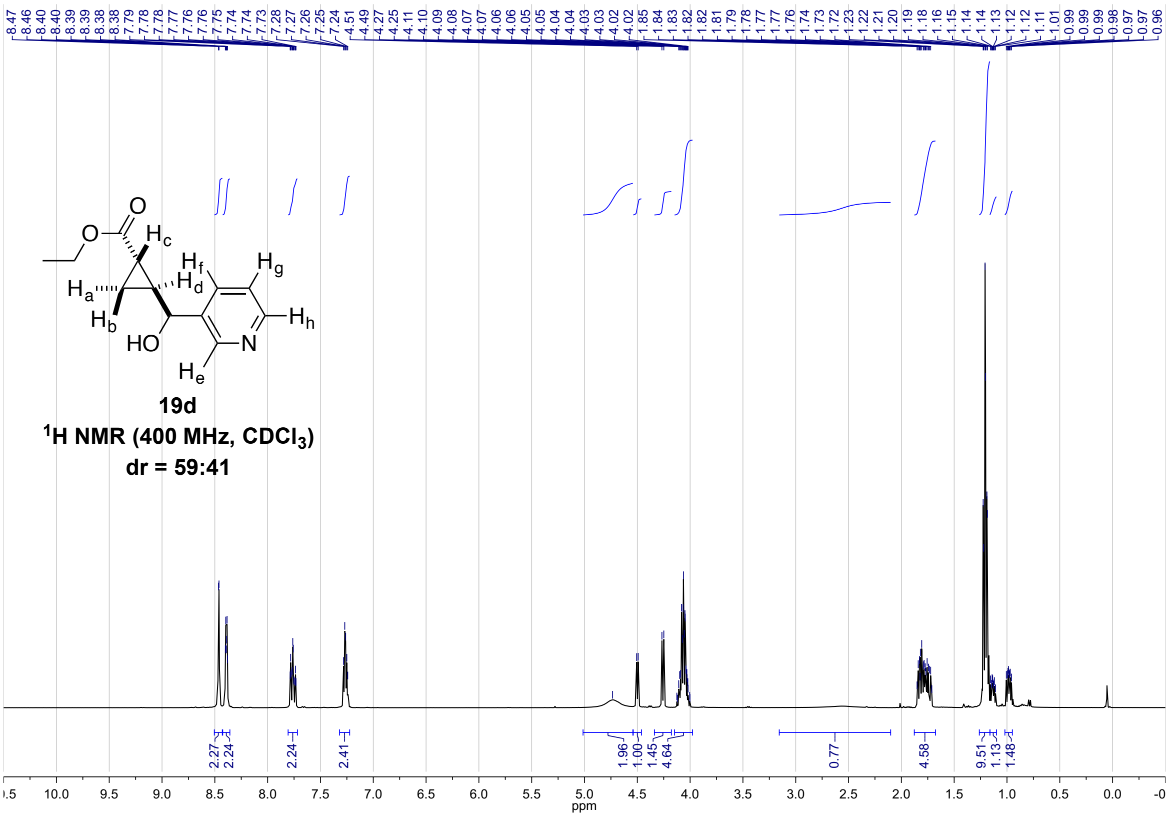

19d
^{1}H NMR (400 MHz, $CDCl_3$)
dr = 59:41

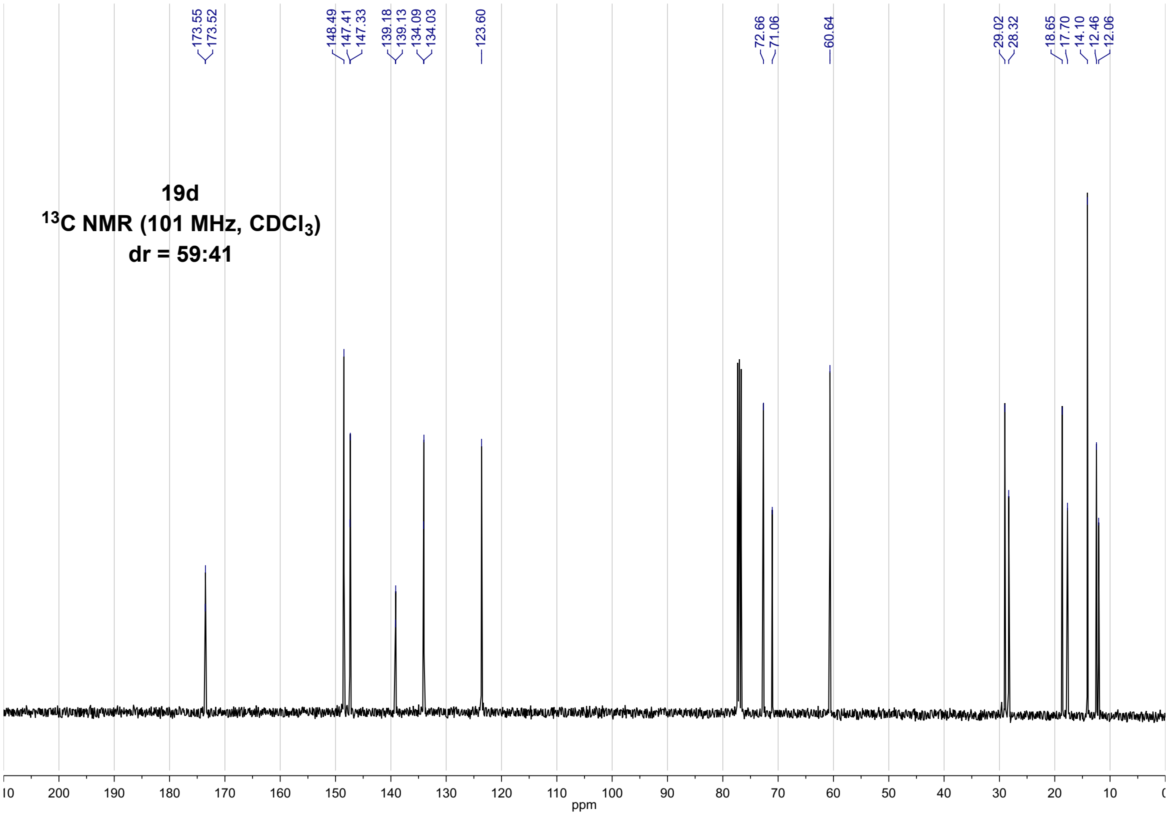

19d
^{13}C NMR (101 MHz, $CDCl_3$)
dr = 59:41

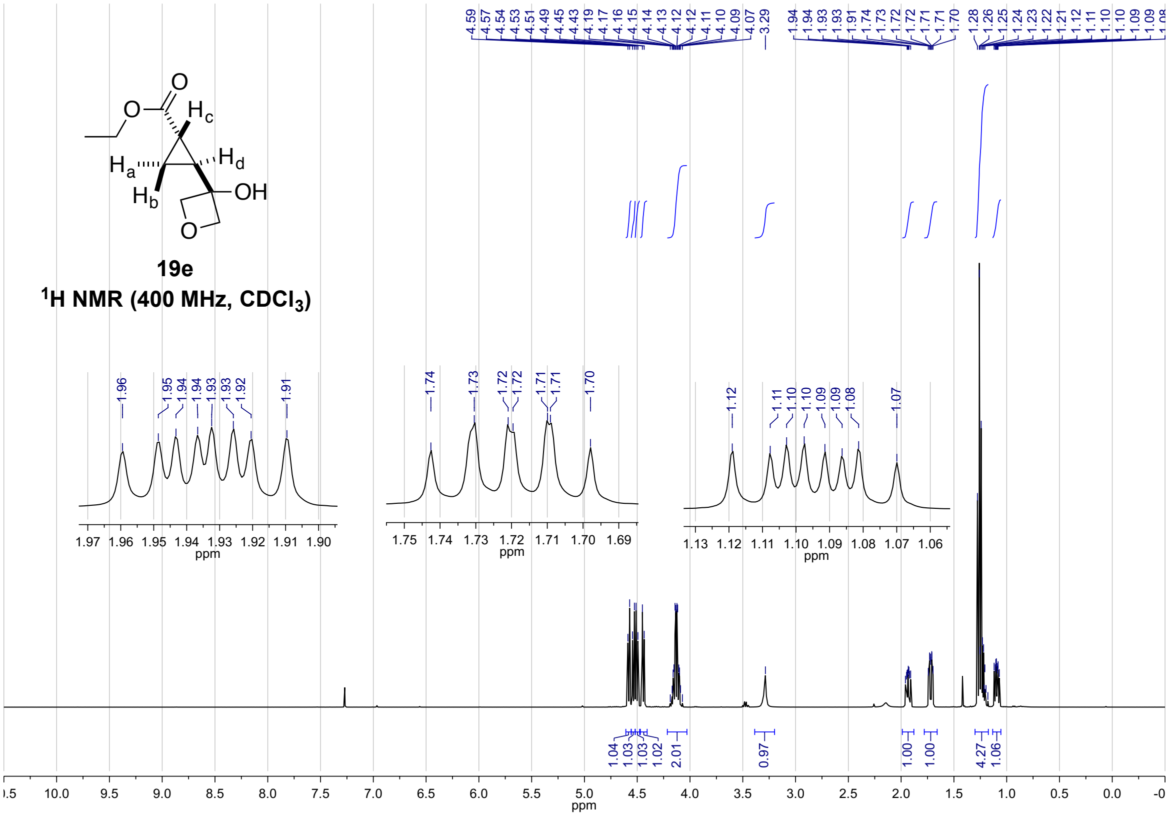

19e
^{1}H NMR (400 MHz, CDCl$_3$)

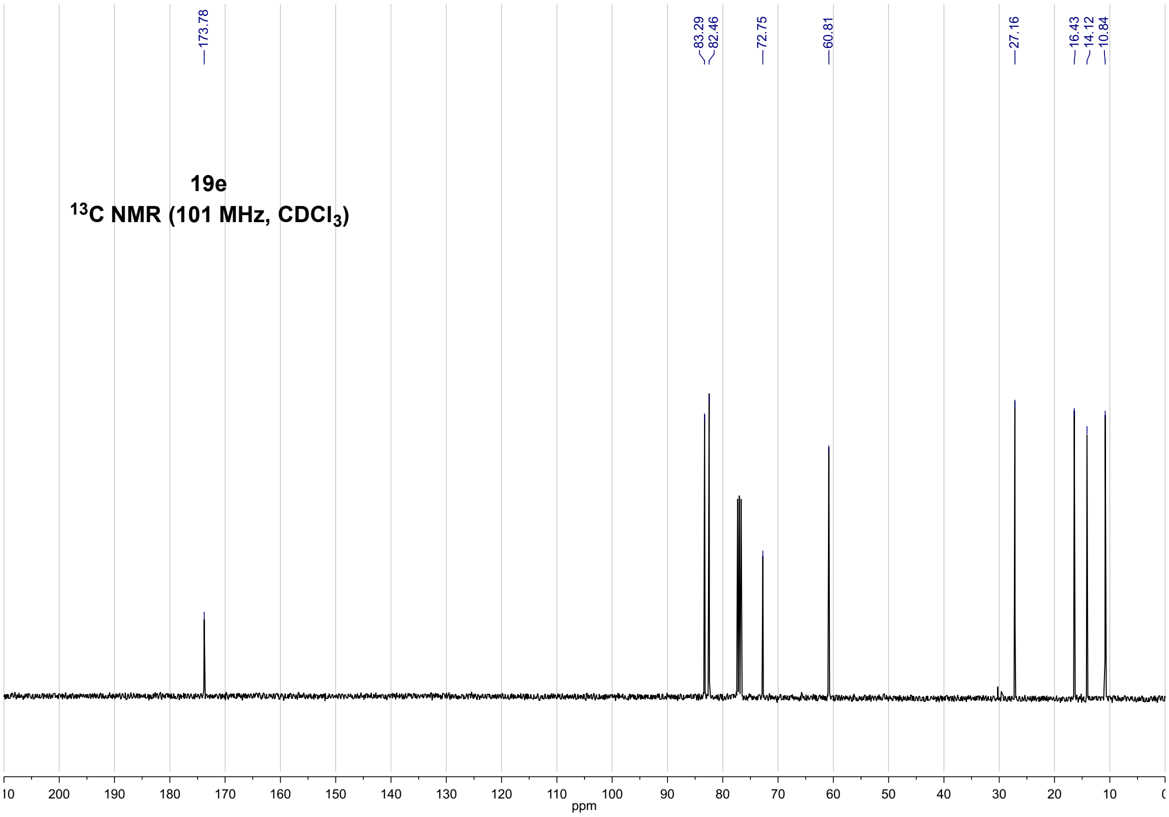

19e
^{13}C NMR (101 MHz, CDCl$_3$)

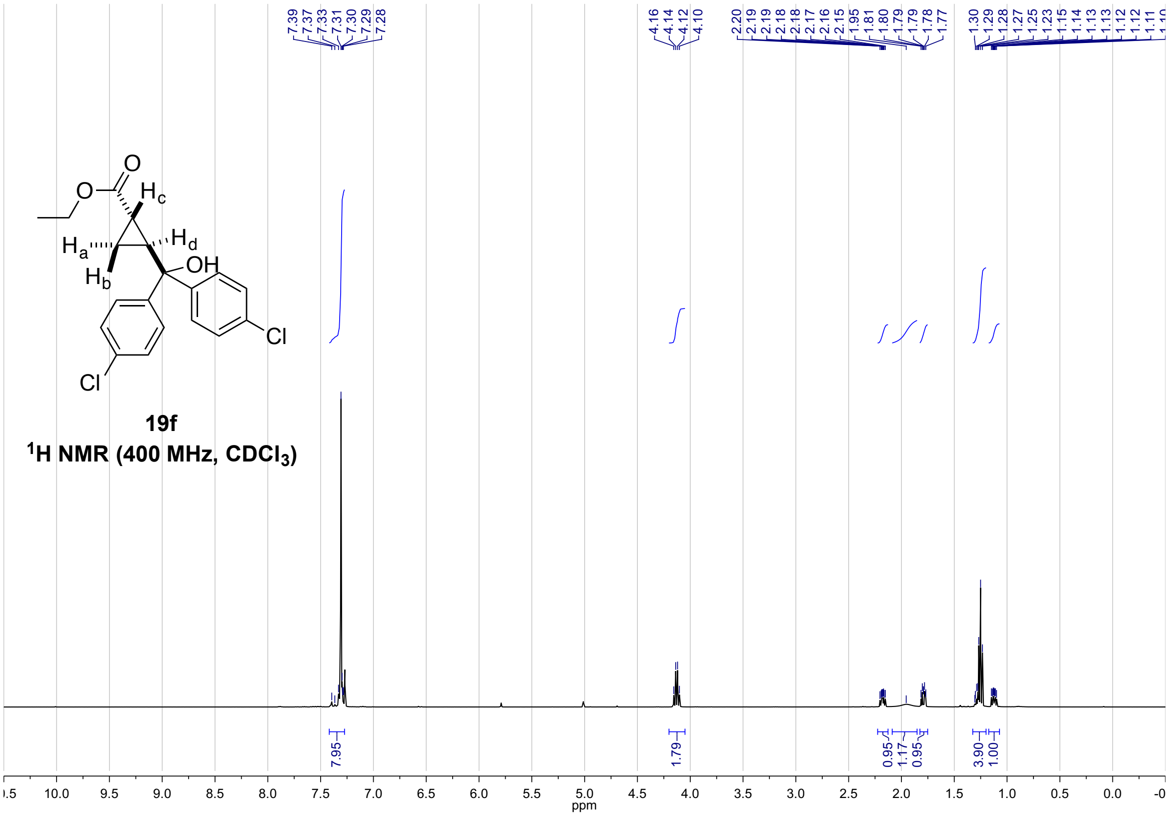

19f
^{1}H NMR (400 MHz, CDCl$_3$)
ppm

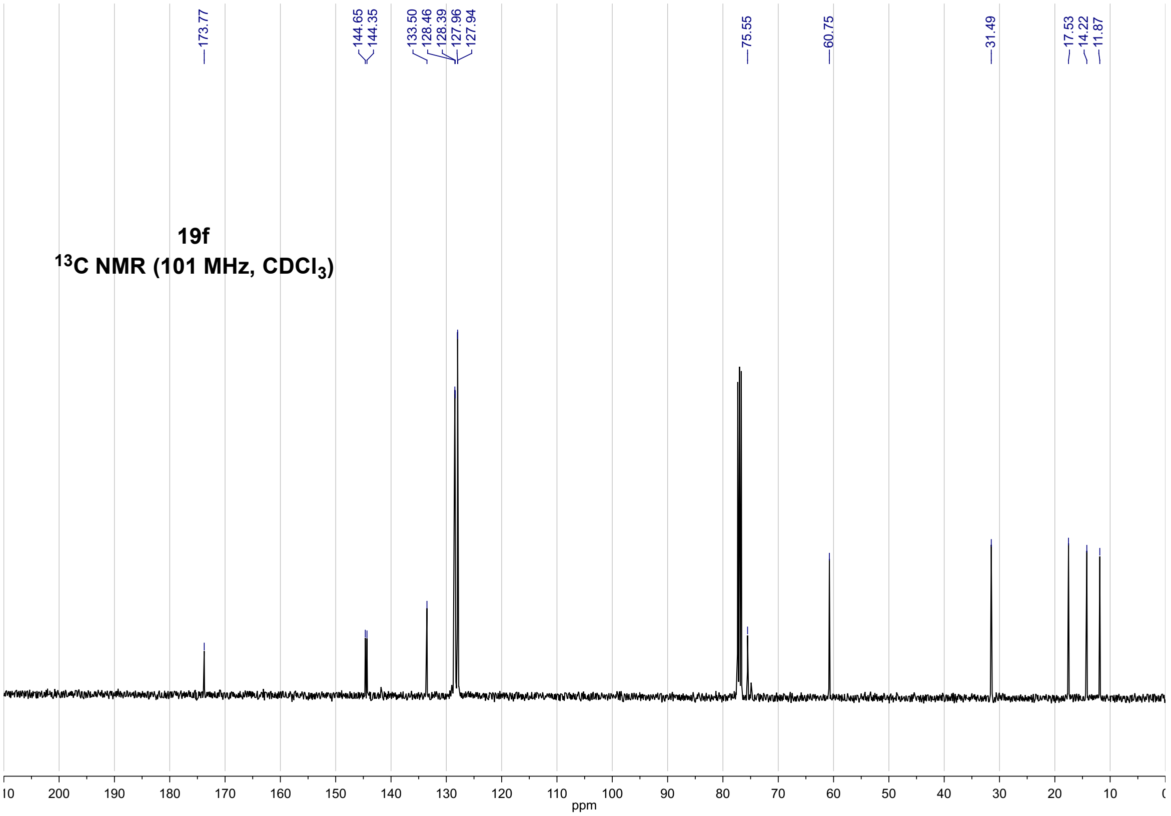

19f
^{13}C NMR (101 MHz, CDCl$_3$)
ppm

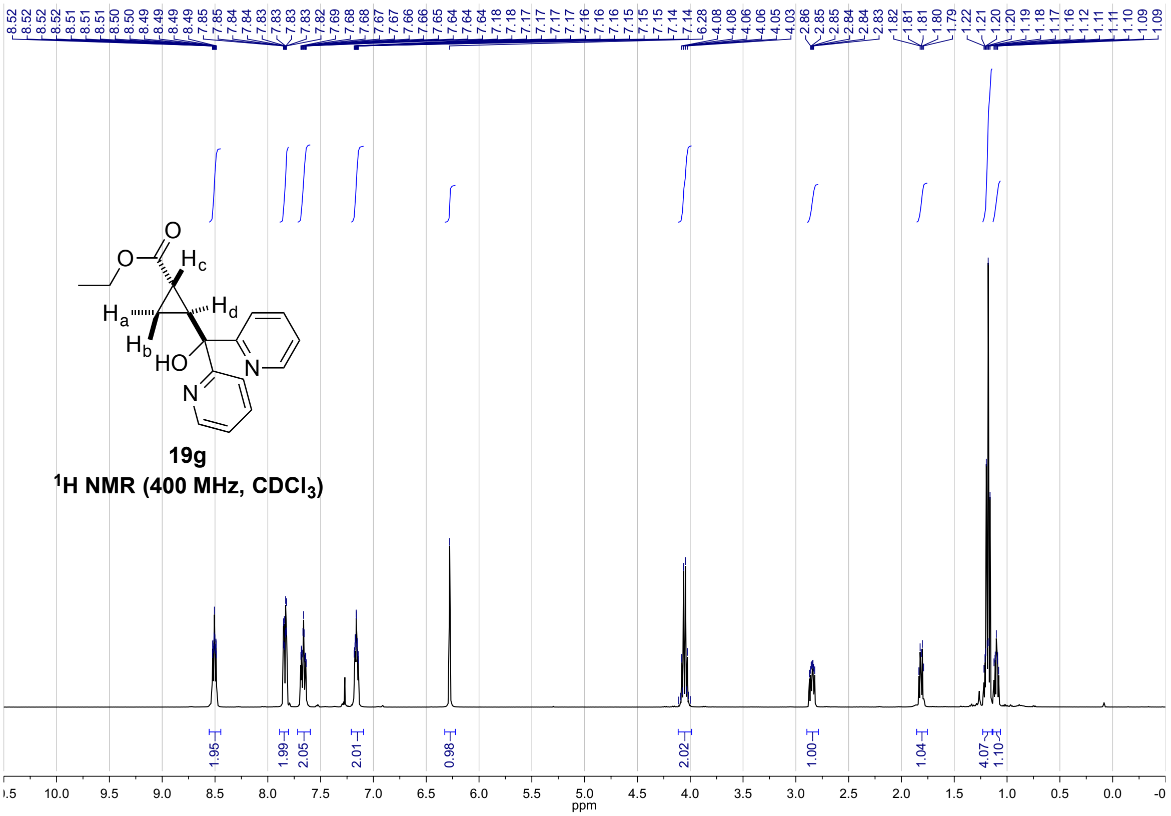
19g
1H NMR (400 MHz, CDCl3)
O
O
Hc
Ha
Hd
Hb
HO
N
N
ppm

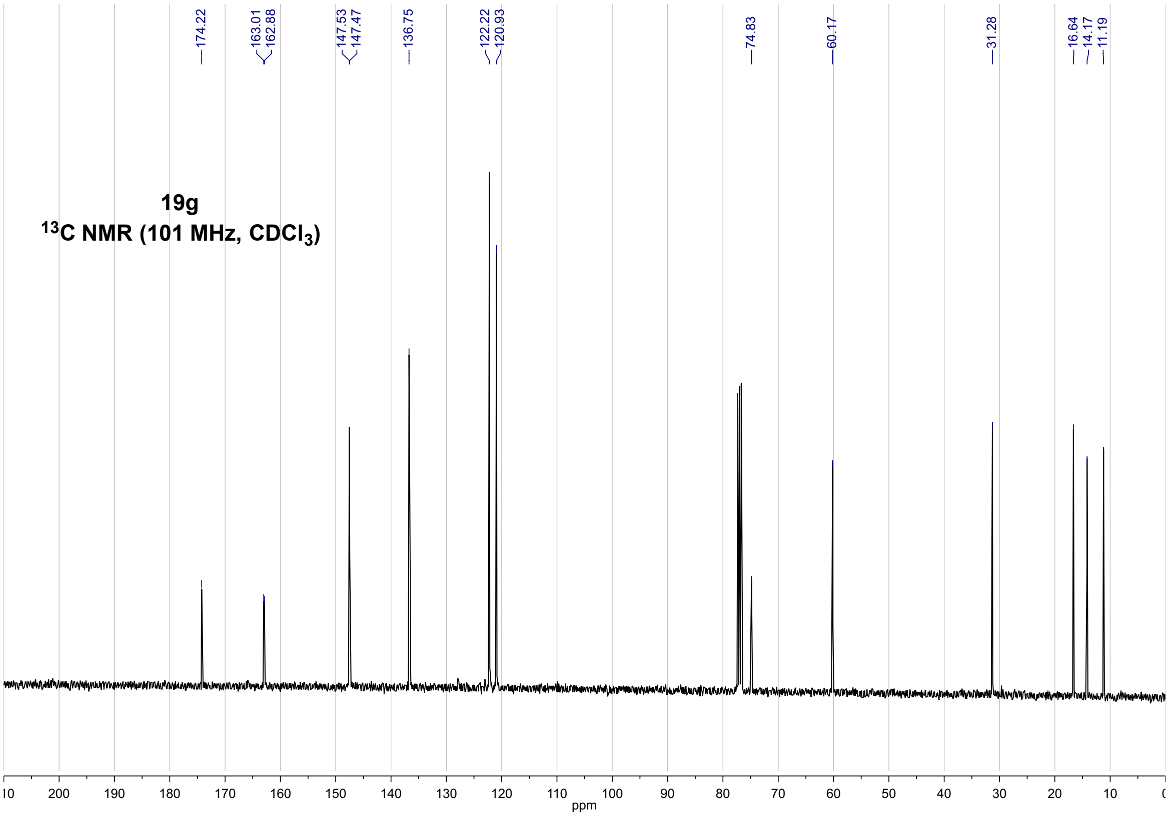
19g
13C NMR (101 MHz, CDCl3)
174.22
163.01
162.88
147.53
147.47
136.75
122.22
120.93
74.83
60.17
31.28
16.64
14.17
11.19
ppm

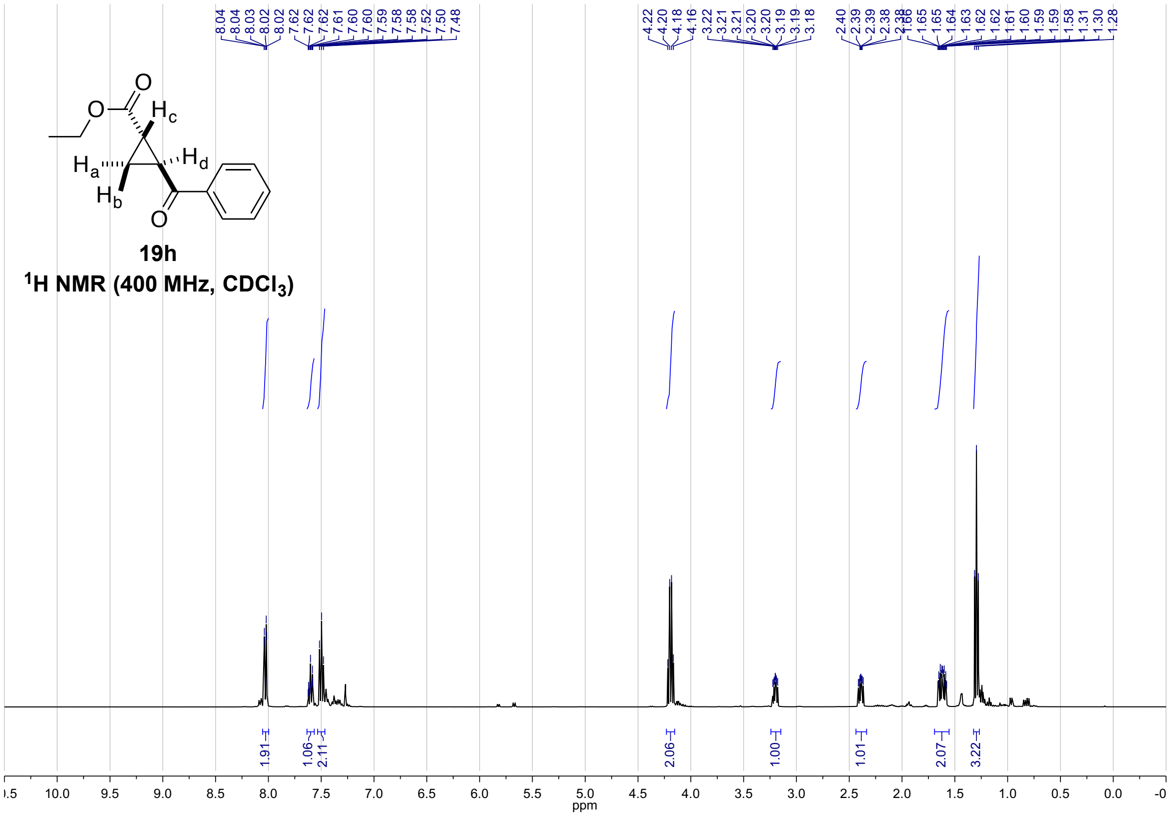
19h
¹H NMR (400 MHz, CDCl₃)
8.04 8.04 8.03 8.02 8.02 7.62 7.62 7.61 7.60 7.60 7.59 7.58 7.58 7.52 7.50 7.48
4.22 4.20 4.18 4.16 3.22 3.21 3.21 3.20 3.20 3.19 3.19 3.18
2.40 2.39 2.39 2.38 2.38 1.66 1.65 1.65 1.64 1.63 1.62 1.62 1.61 1.60 1.59 1.59 1.58 1.31 1.30 1.28
1.91 1.06 2.11 2.06 1.00 1.01 2.07 3.22
ppm

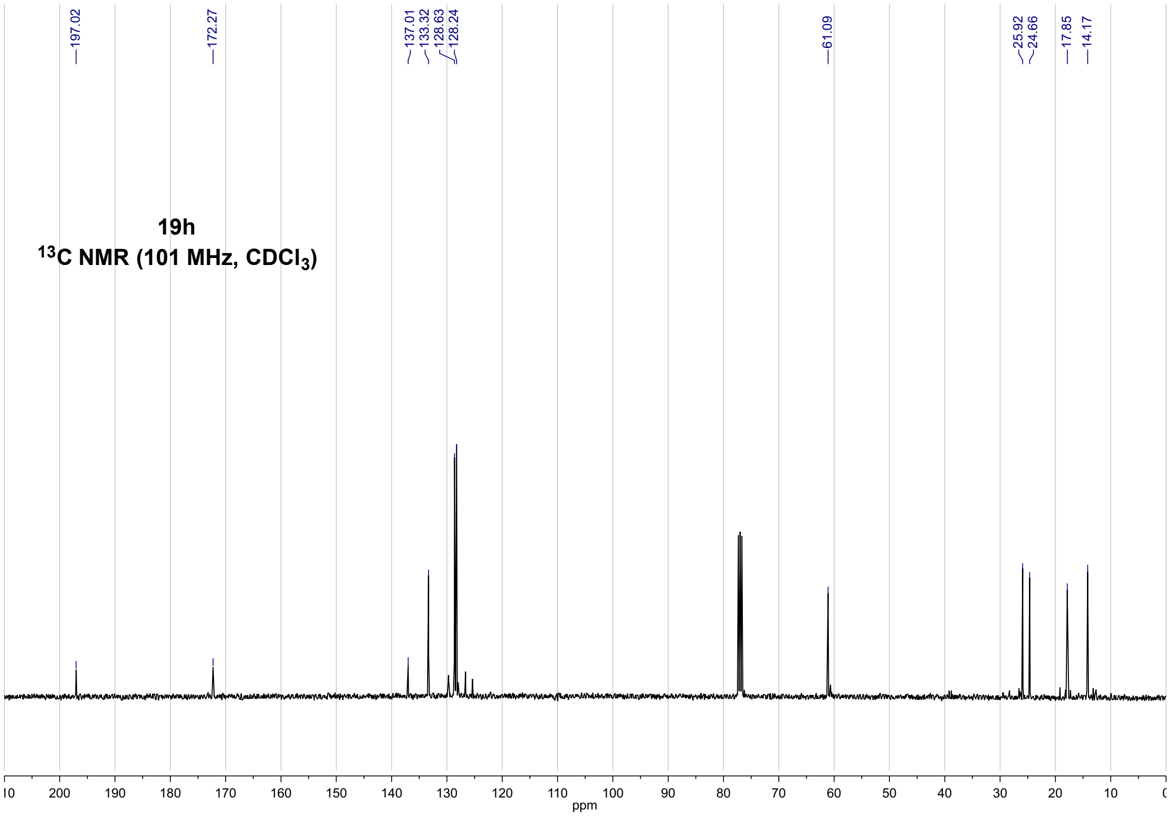
19h
¹³C NMR (101 MHz, CDCl₃)
197.02
172.27
137.01 133.32 128.63 128.24
61.09
25.92 24.66 17.85 14.17
ppm

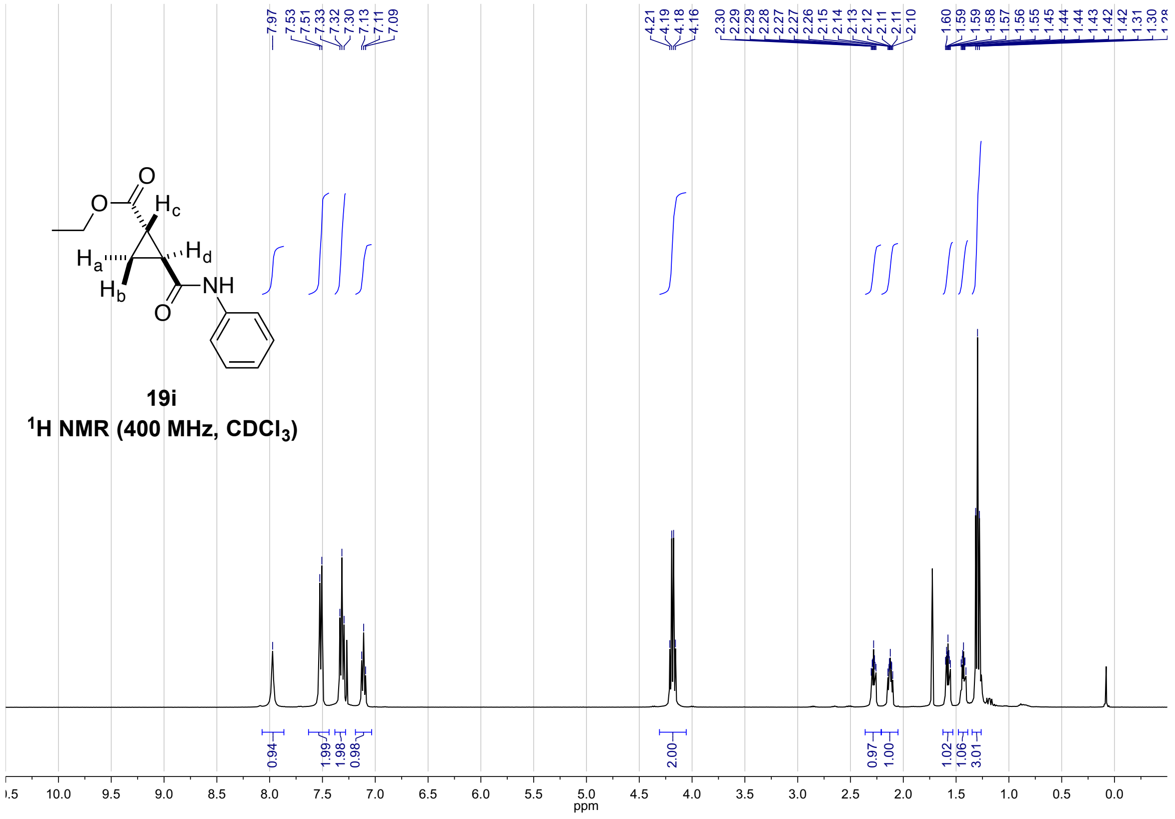

19i
^{1}H NMR (400 MHz, CDCl$_3$)

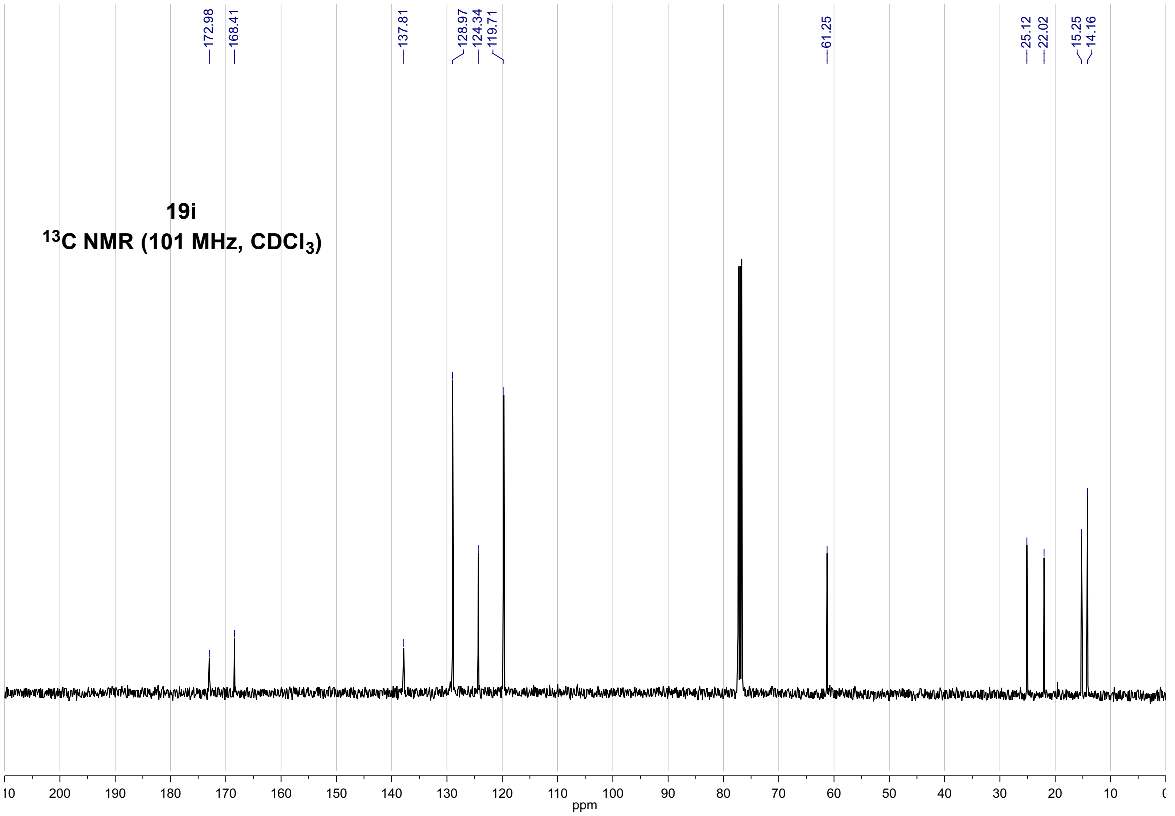

19i
^{13}C NMR (101 MHz, CDCl$_3$)
172.98
168.41
137.81
128.97
124.34
119.71
61.25
25.12
22.02
15.25
14.16

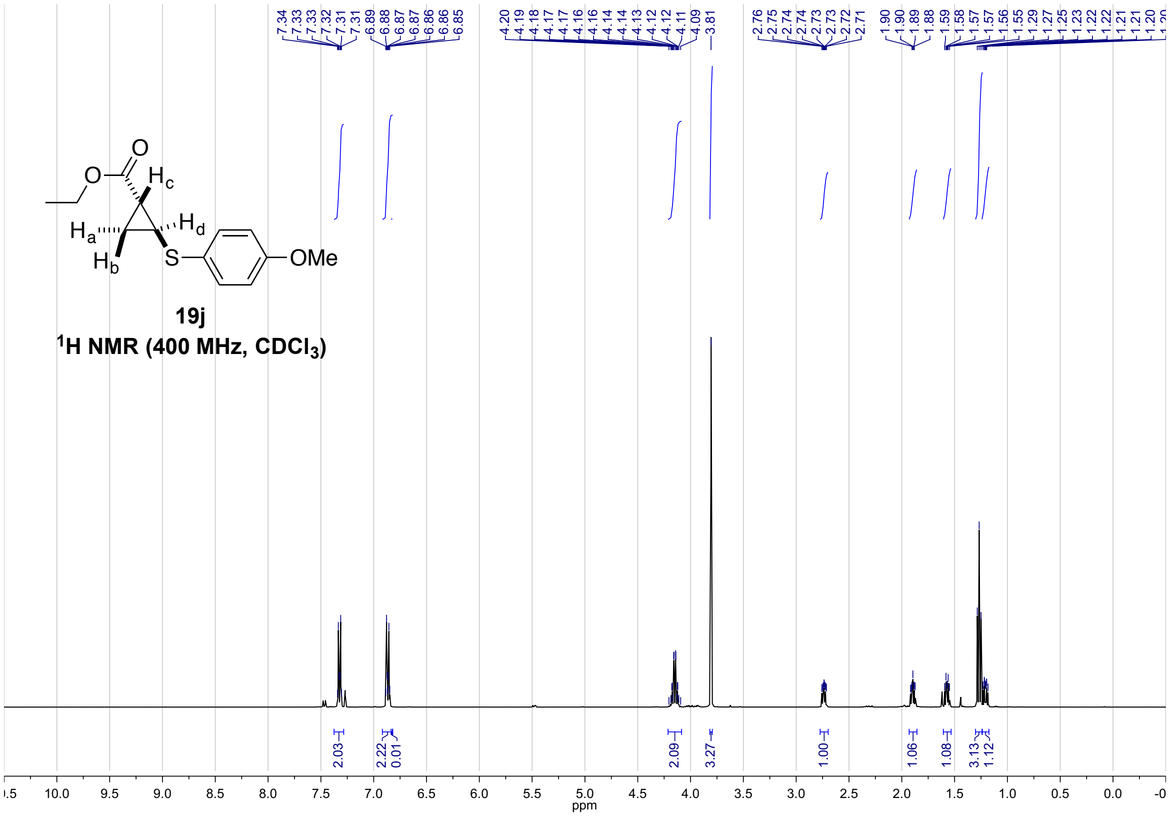
19j
^{1}H NMR (400 MHz, CDCl$_3$)
ppm

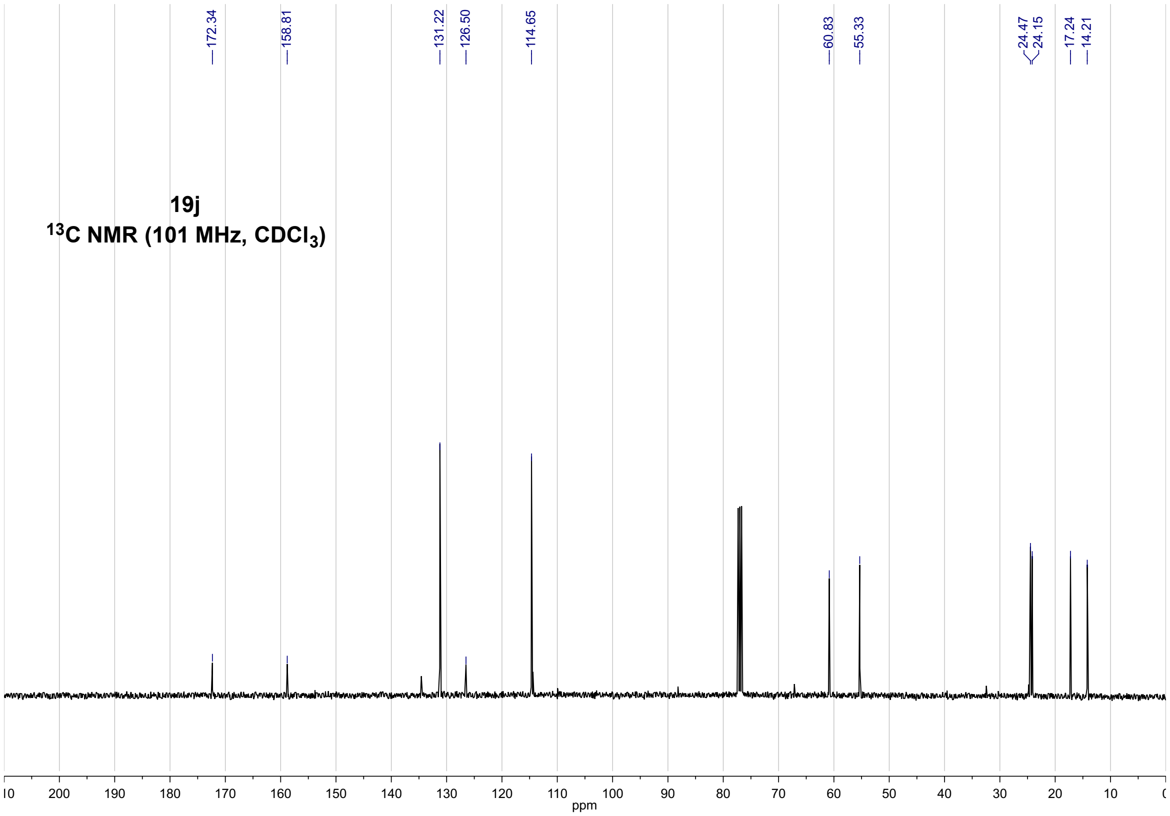
19j
^{13}C NMR (101 MHz, CDCl$_3$)
172.34
158.81
131.22
126.50
114.65
60.83
55.33
24.47
24.15
17.24
14.21
ppm

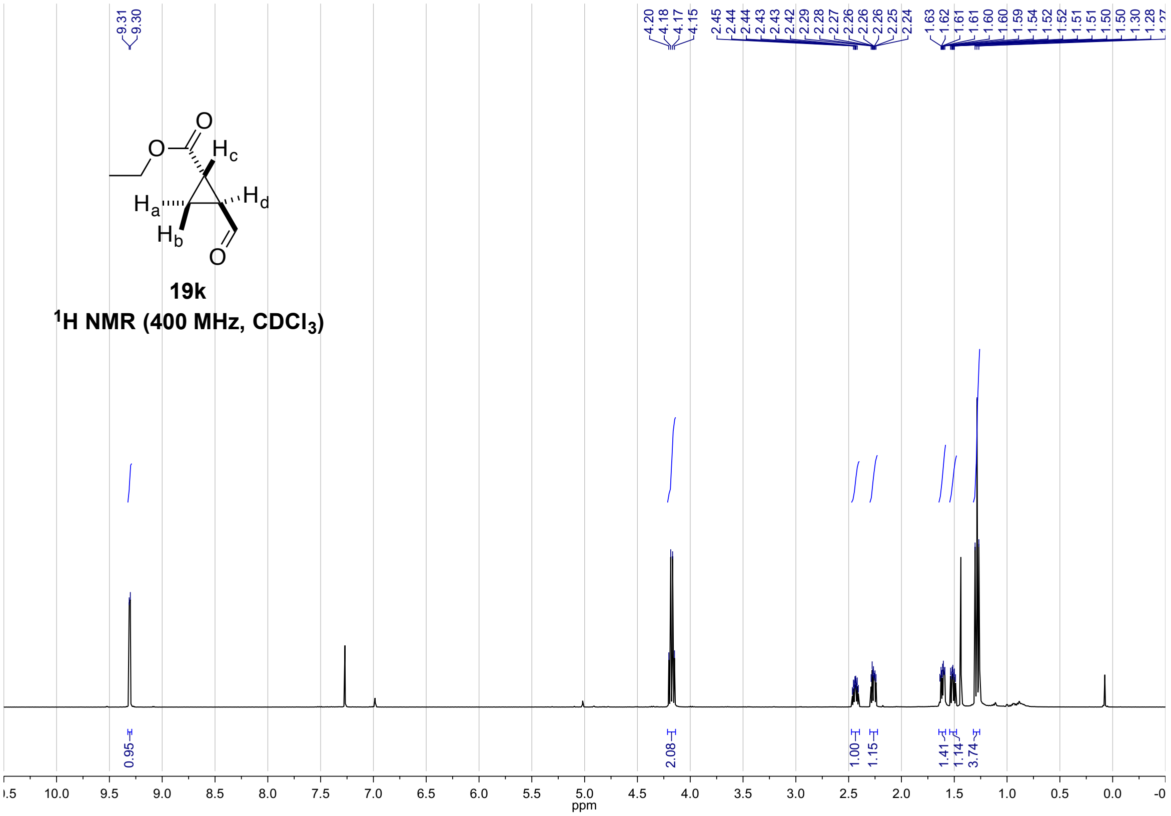
19k
^{1}H NMR (400 MHz, CDCl$_3$)

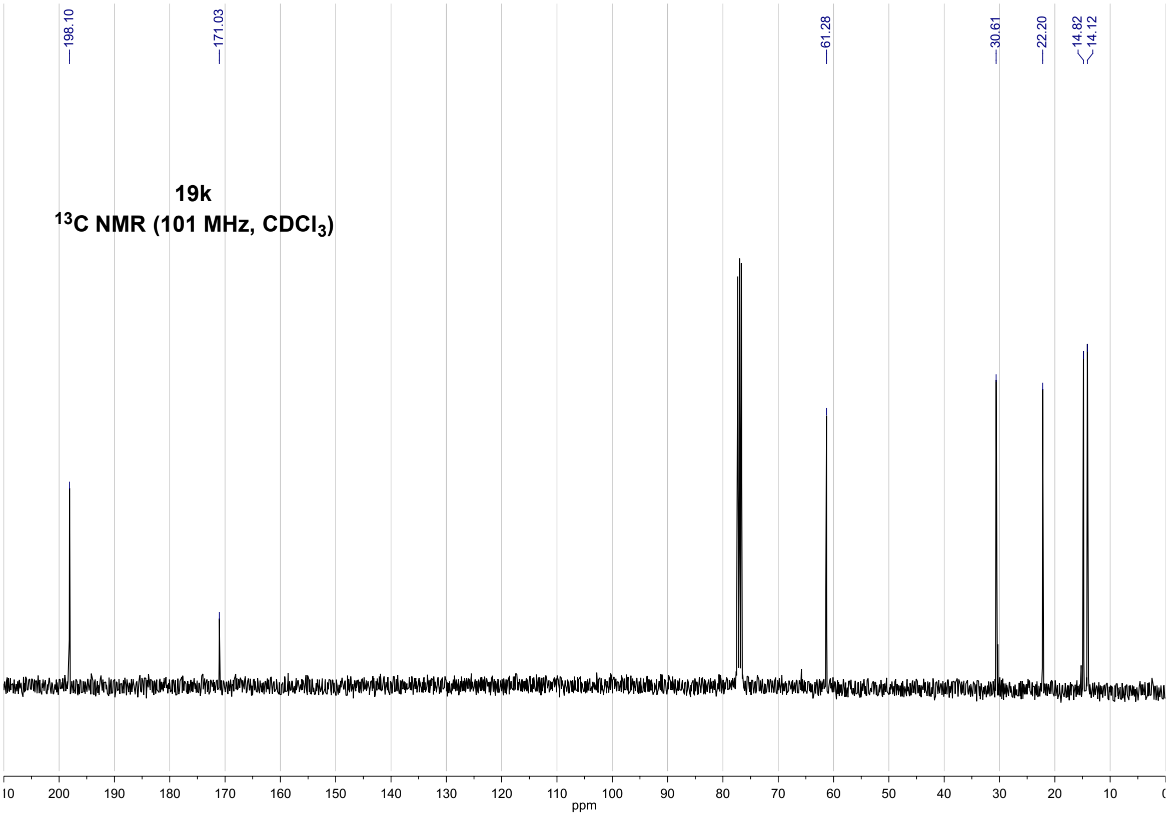
19k
^{13}C NMR (101 MHz, CDCl$_3$)

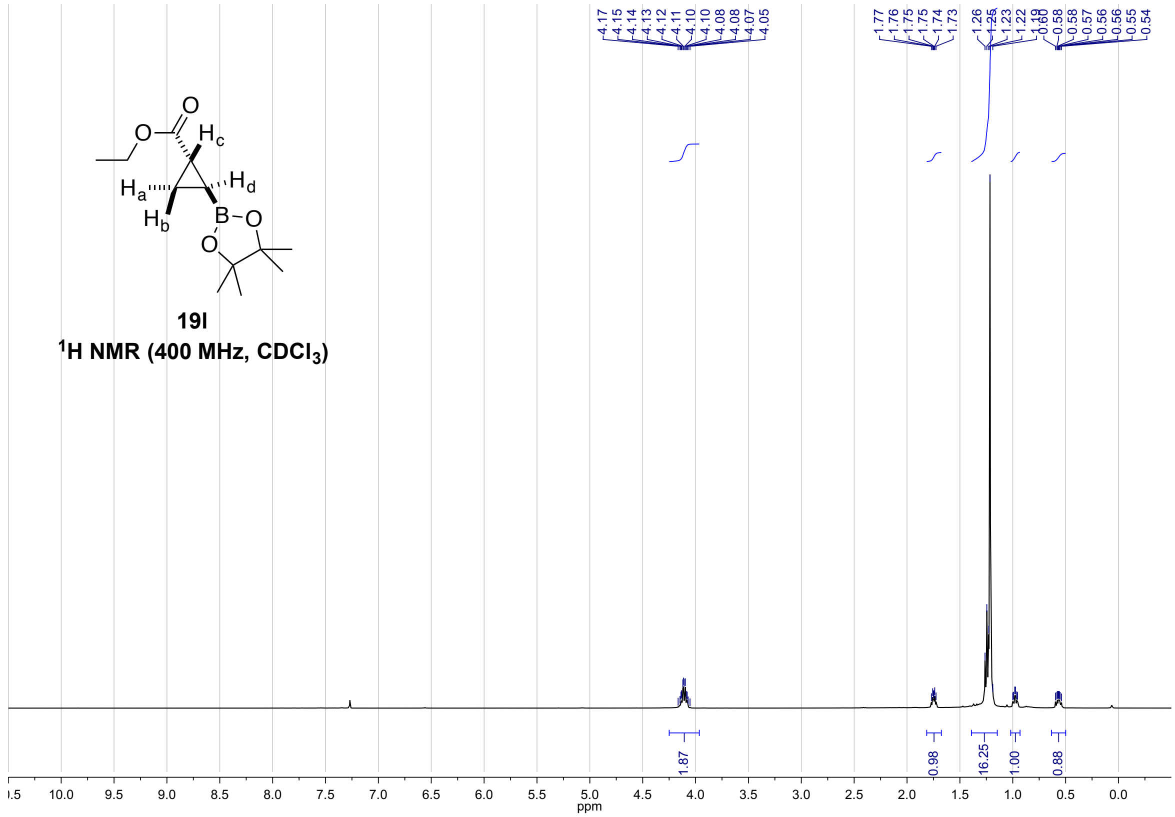

19l
^1H NMR (400 MHz, $CDCl_3$)

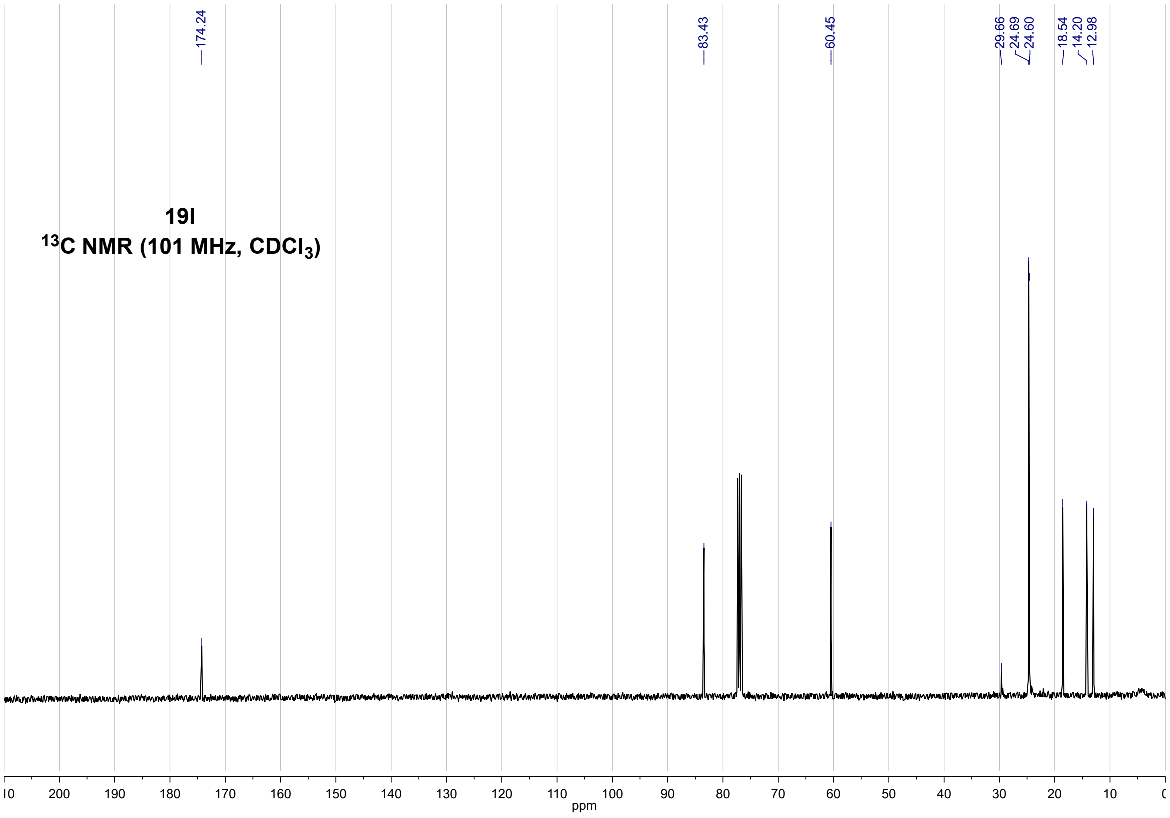

19l
^{13}C NMR (101 MHz, $CDCl_3$)

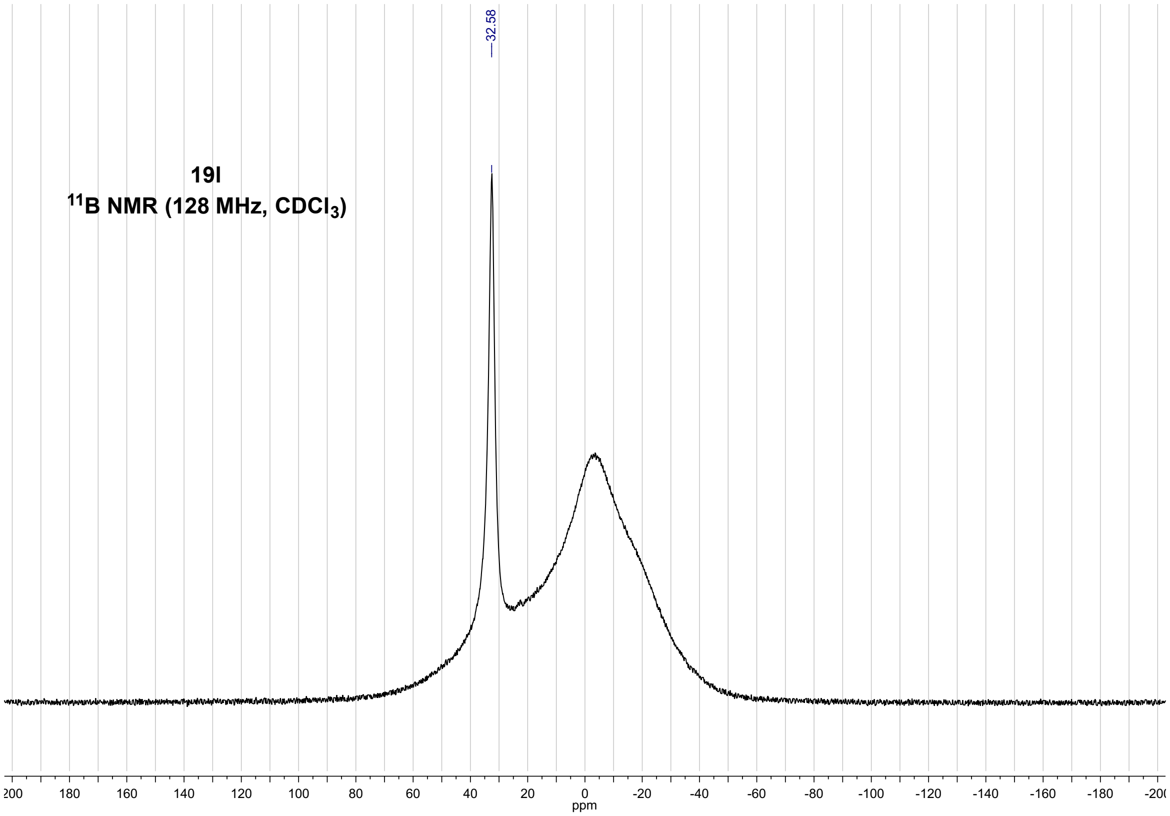
32.58
19l
11B NMR (128 MHz, CDCl3)
200
180
160
140
120
100
80
60
40
20
0
-20
-40
-60
-80
-100
-120
-140
-160
-180
ppm

19m

^{1}H NMR (400 MHz, $CDCl_3$)

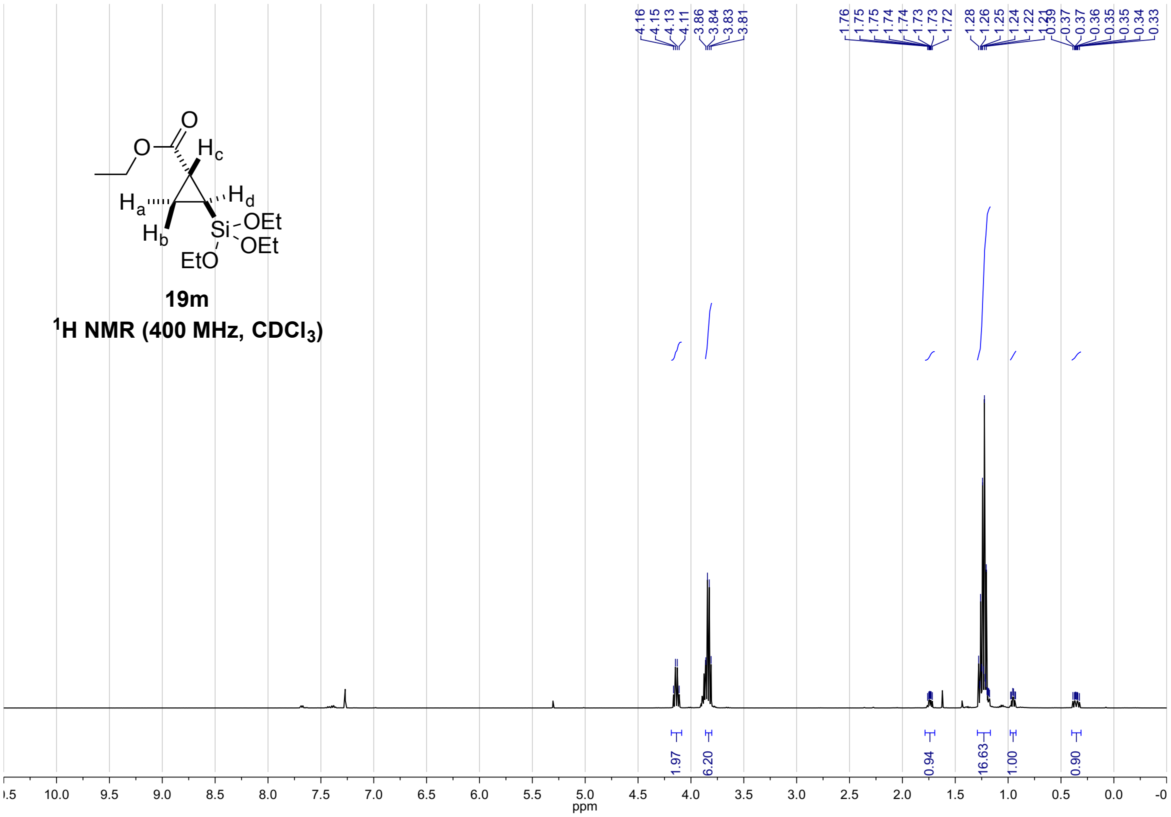

19m

^{13}C NMR (101 MHz, $CDCl_3$)

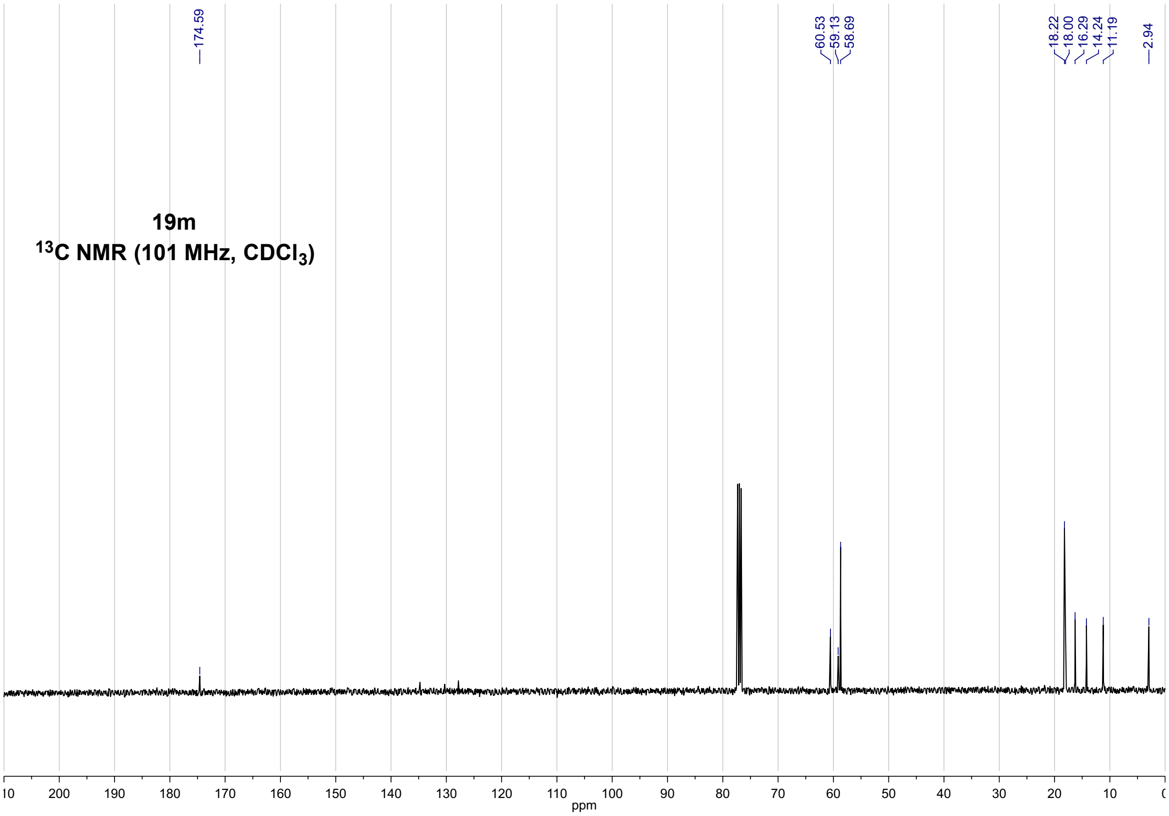

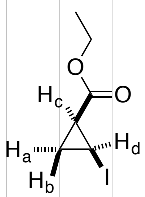

20a
^{1}H NMR (400 MHz, CDCl$_3$)

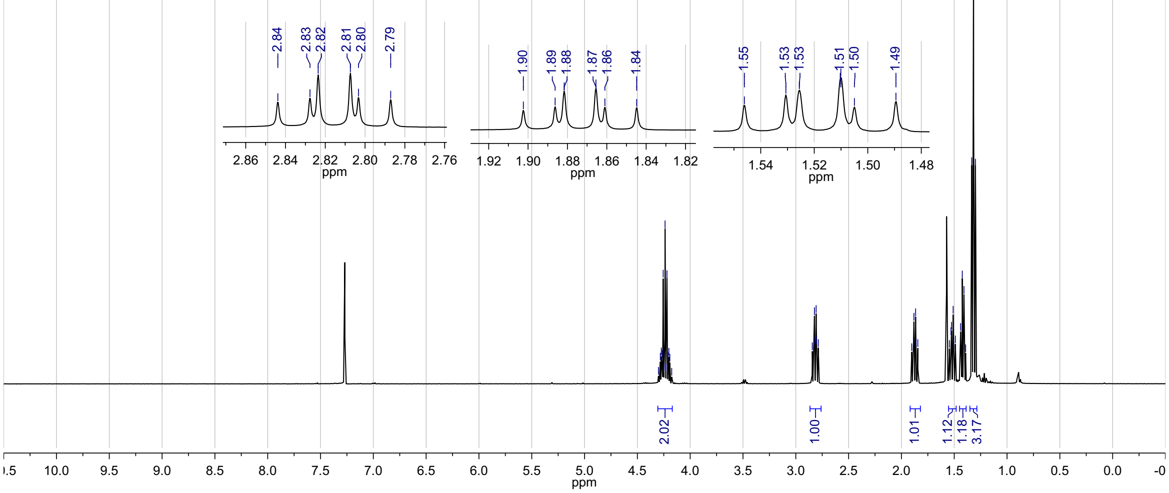

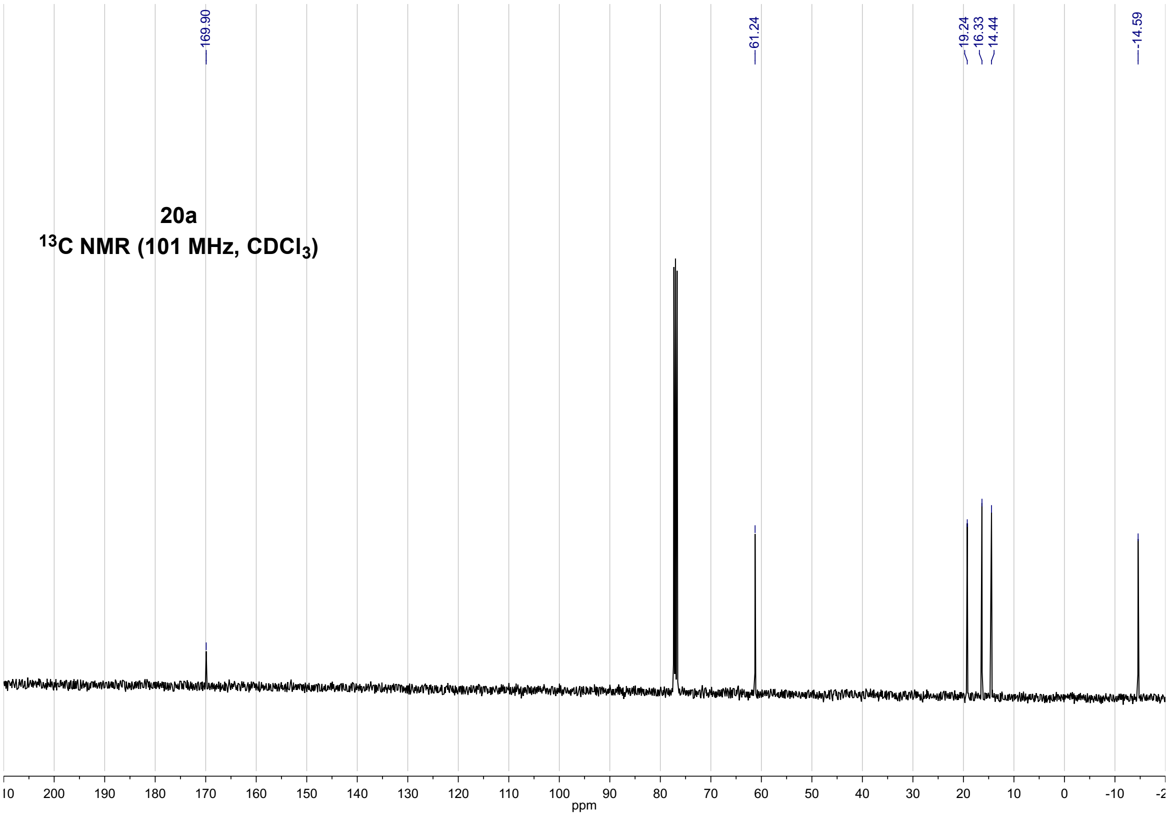

20a
^{13}C NMR (101 MHz, CDCl$_3$)

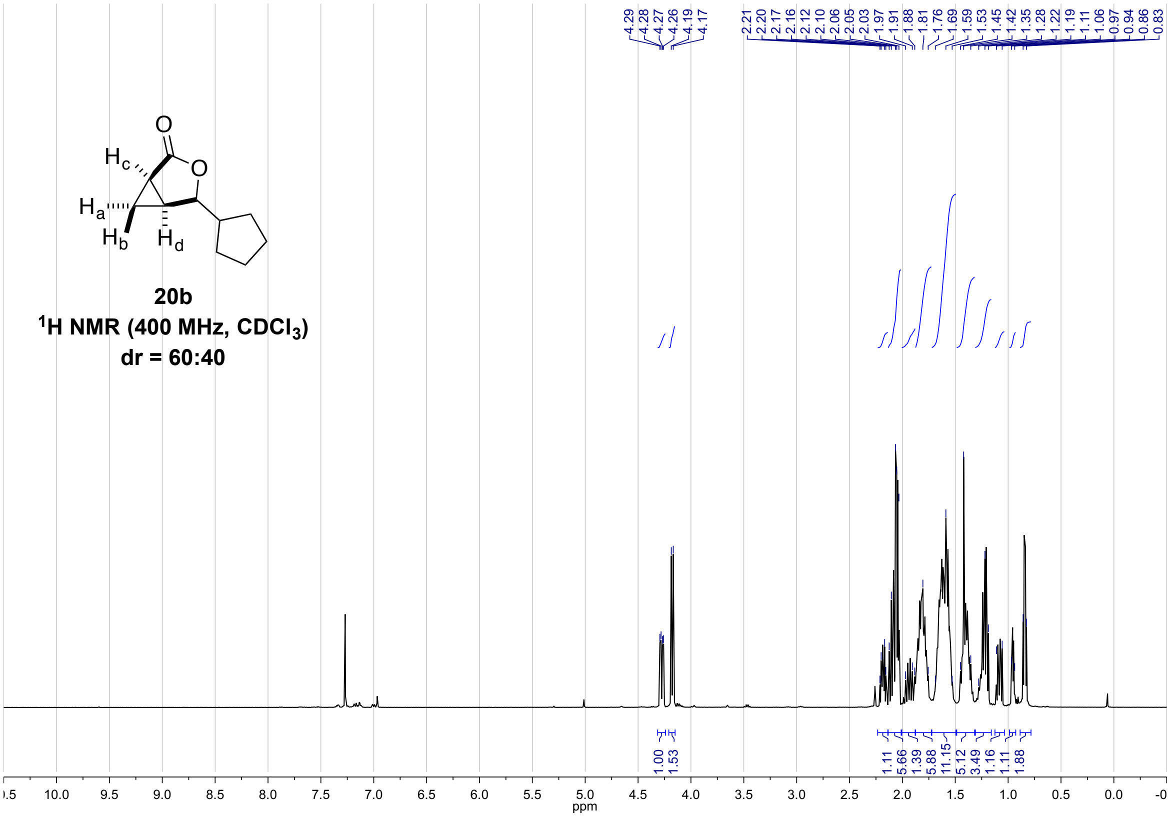
4.29
4.28
4.27
4.26
4.19
4.17
2.21
2.20
2.17
2.16
2.12
2.10
2.06
2.05
2.03
1.97
1.91
1.88
1.81
1.76
1.69
1.59
1.53
1.45
1.42
1.35
1.28
1.22
1.19
1.11
1.06
0.97
0.94
0.86
0.83
O
O
Hc
Ha
Hb
Hd
20b
1H NMR (400 MHz, CDCl3)
dr = 60:40
1.00
1.53
1.11
5.66
1.39
5.88
11.15
5.12
3.49
1.16
1.11
1.88
ppm

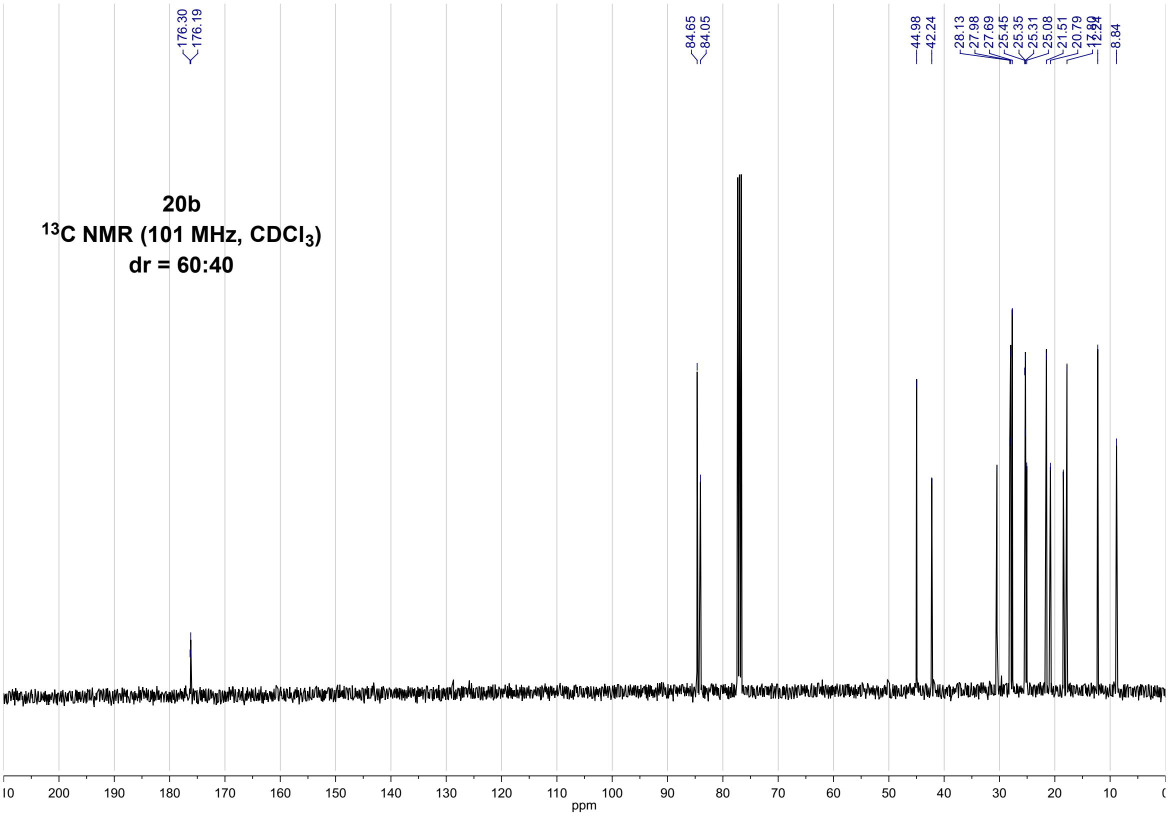
176.30
176.19
84.65
84.05
44.98
42.24
28.13
27.98
27.69
25.45
25.35
25.31
25.08
21.51
20.79
8.84
20b
13C NMR (101 MHz, CDCl3)
dr = 60:40
ppm

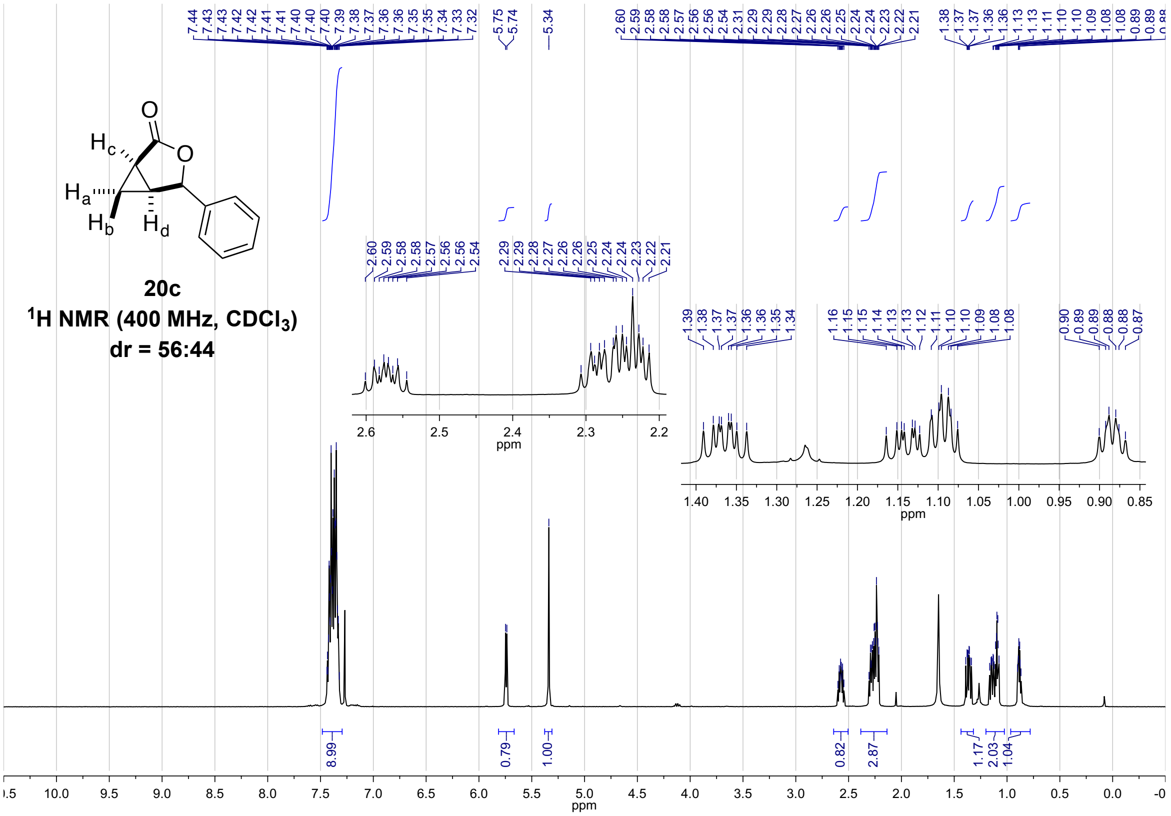
20c
^{1}H NMR (400 MHz, CDCl$_3$)
dr = 56:44
7.44 7.43 7.43 7.42 7.42 7.41 7.41 7.40 7.40 7.39 7.38 7.37 7.36 7.36 7.35 7.35 7.34 7.33 7.32
5.75 5.74
5.34
2.60 2.59 2.58 2.58 2.57 2.56 2.56 2.54 2.31 2.29 2.29 2.28 2.27 2.26 2.26 2.25 2.24 2.24 2.23 2.22 2.21
1.38 1.37 1.37 1.36 1.36 1.13 1.13 1.11 1.10 1.09 1.08 1.08 0.89 0.89
8.99
0.79
1.00
0.82
2.87
1.17
2.03
1.04
ppm

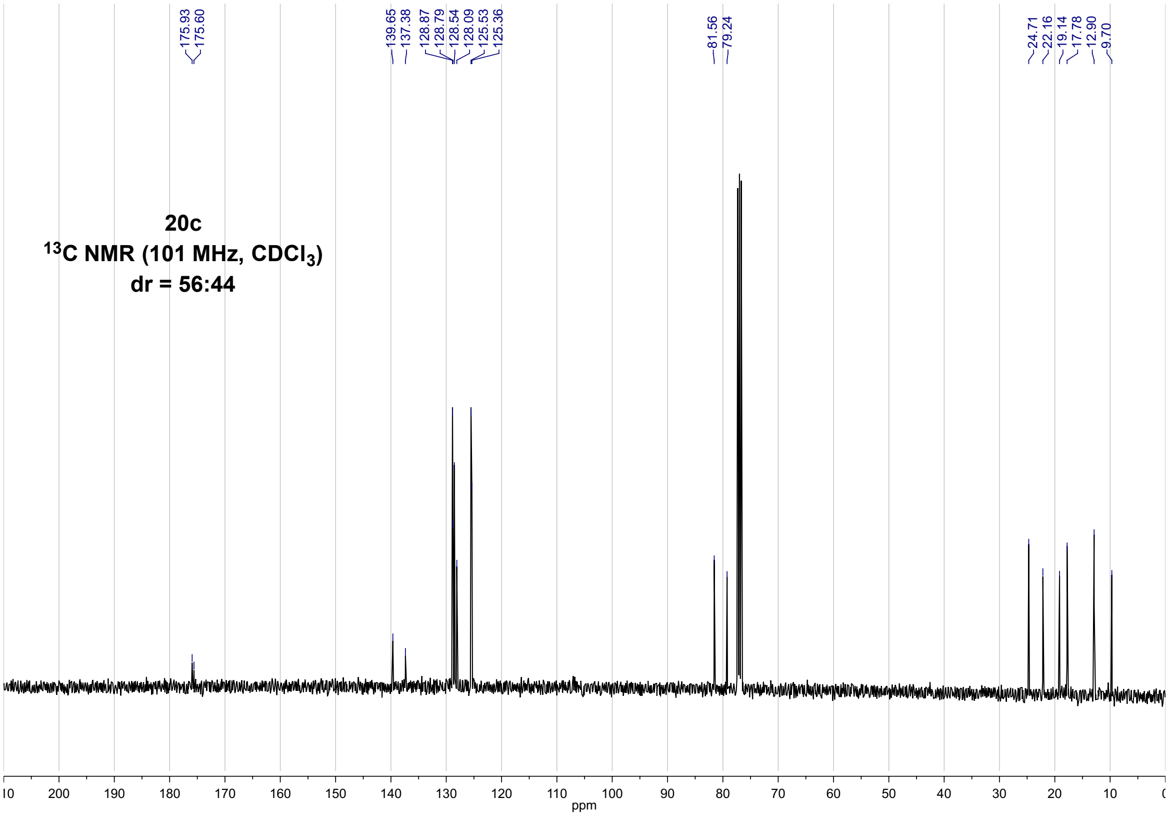
20c
^{13}C NMR (101 MHz, CDCl$_3$)
dr = 56:44
175.93 175.60
139.65 137.38 128.87 128.79 128.54 128.09 125.53 125.36
81.56 79.24
24.71 22.16 19.14 17.78 12.90 9.70
ppm

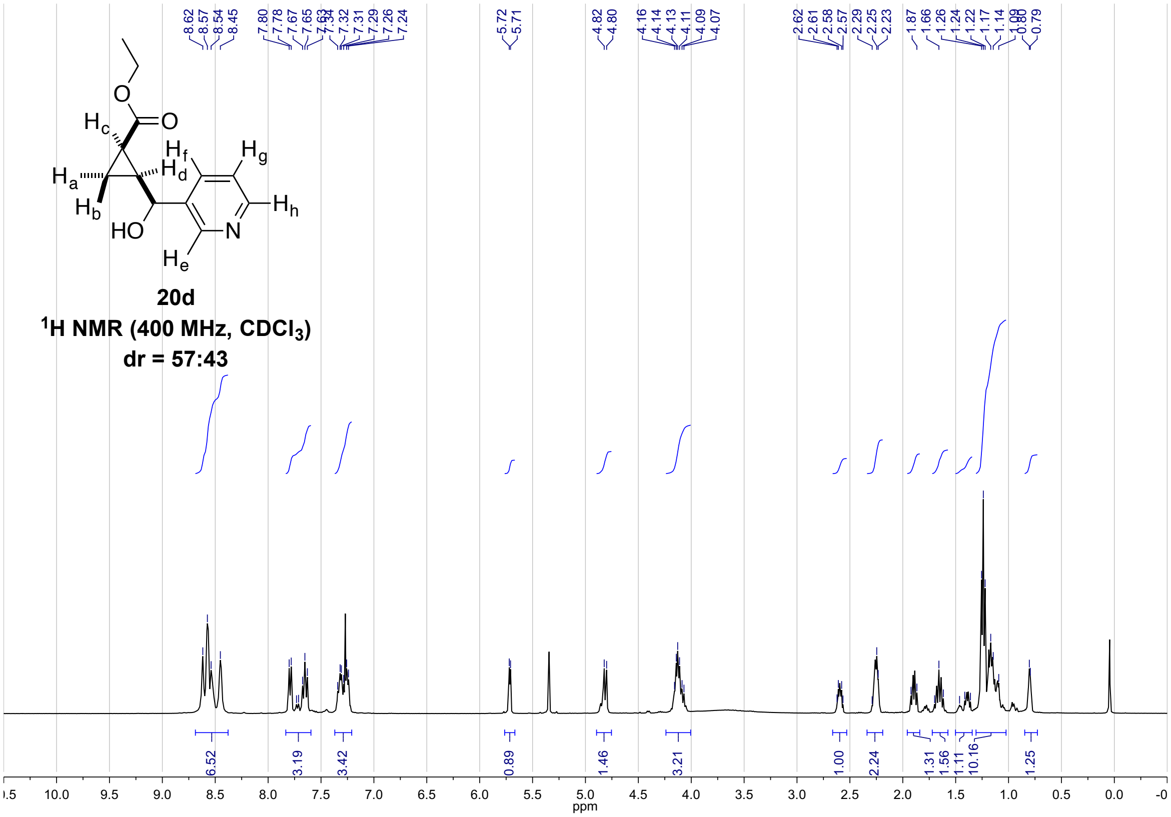
20d
^{1}H NMR (400 MHz, CDCl$_3$)
dr = 57:43
ppm

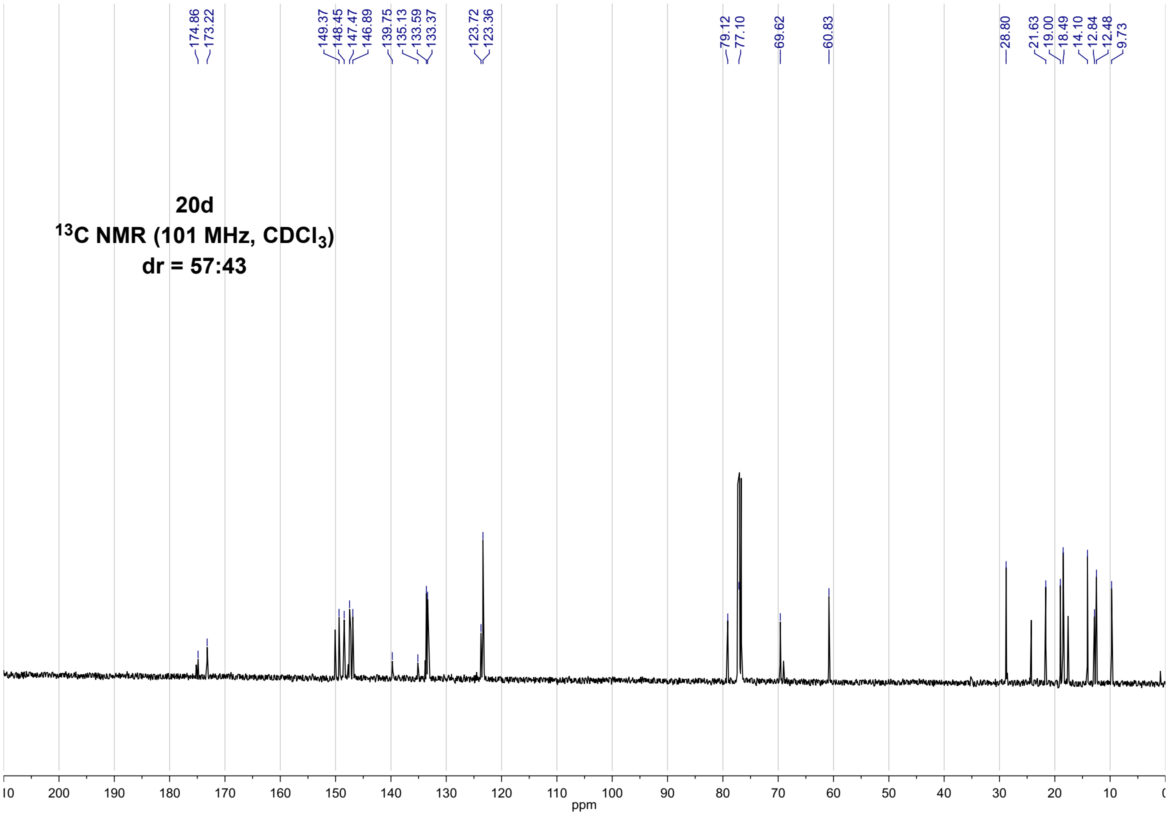
20d
^{13}C NMR (101 MHz, CDCl$_3$)
dr = 57:43
ppm

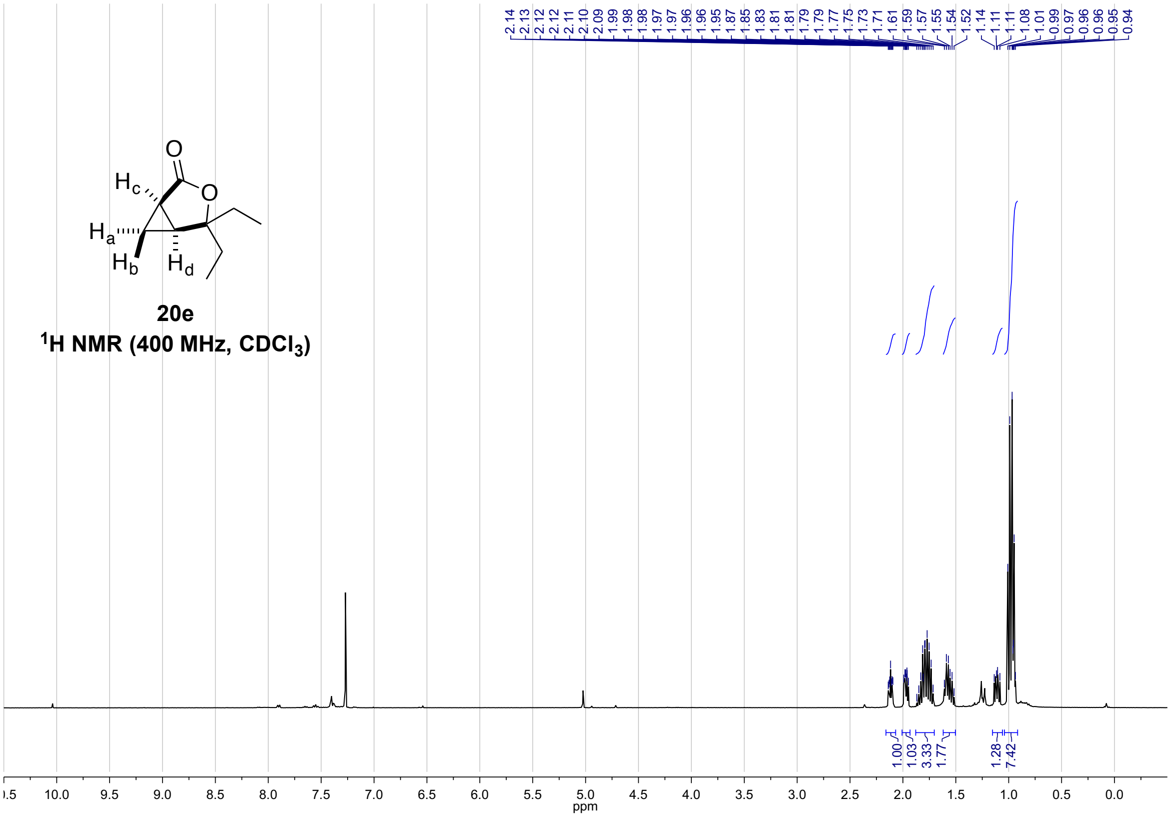

20e
^{1}H NMR (400 MHz, $CDCl_3$)

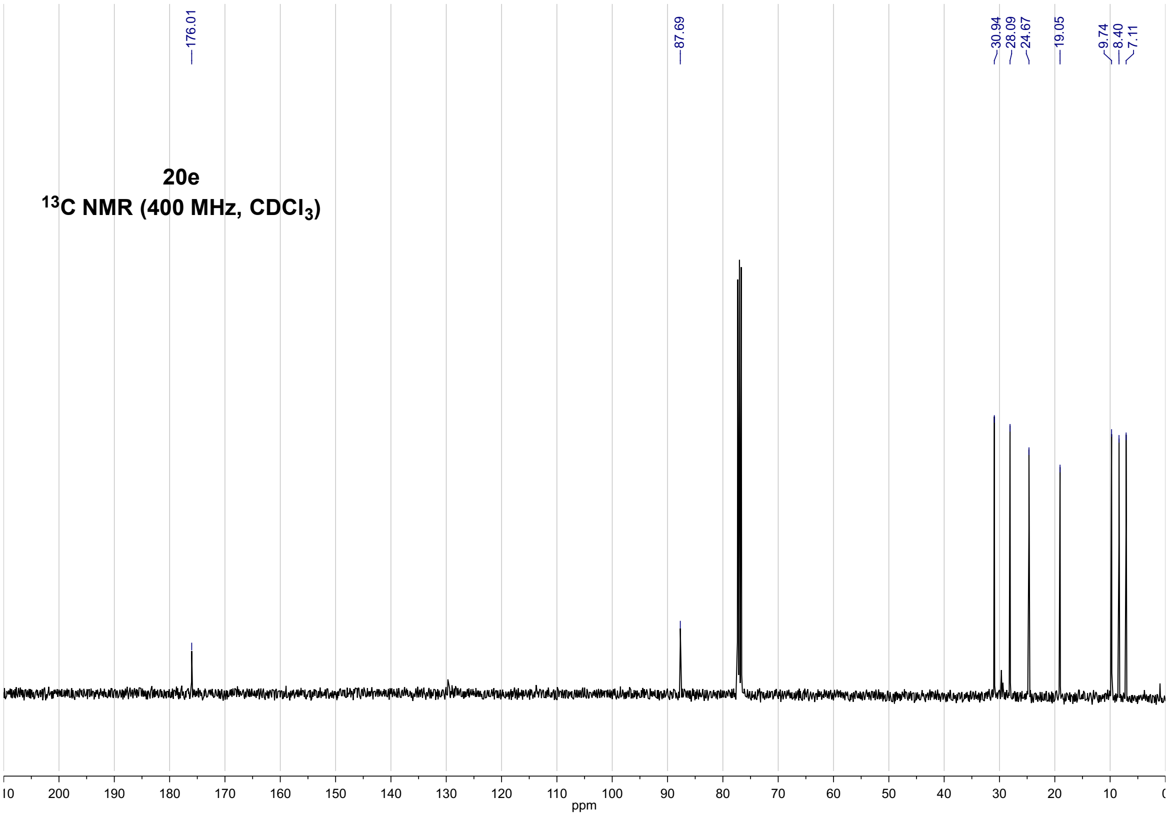

20e
^{13}C NMR (400 MHz, $CDCl_3$)

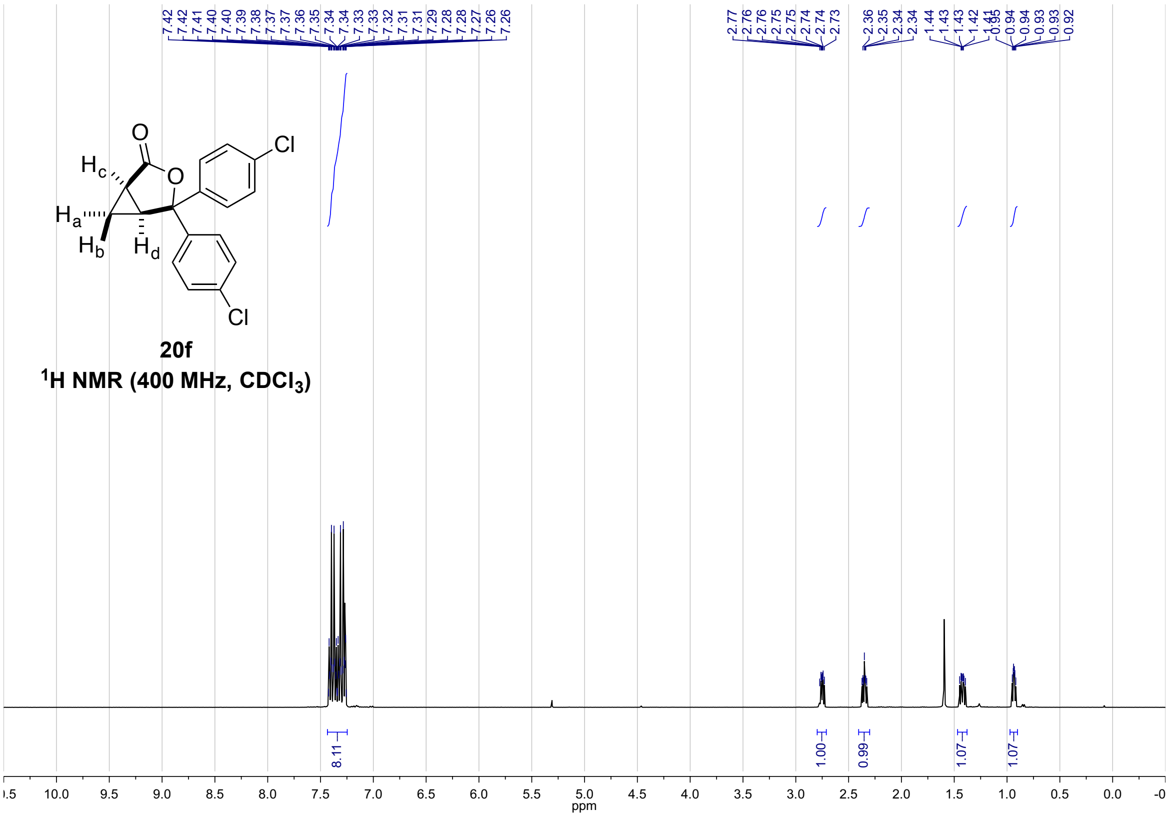
7.42
7.42
7.41
7.40
7.40
7.39
7.38
7.37
7.37
7.36
7.35
7.34
7.34
7.33
7.33
7.32
7.31
7.31
7.29
7.28
7.28
7.27
7.26
7.26
2.77
2.76
2.75
2.75
2.74
2.74
2.73
2.36
2.35
2.34
2.34
1.44
1.43
1.43
1.42
1.41
0.95
0.94
0.94
0.93
0.93
0.92
O
O
Cl
Cl
H_c
H_a
H_b
H_d
20f
^{1}H NMR (400 MHz, $CDCl_3$)
8.11
1.00
0.99
1.07
1.07
ppm

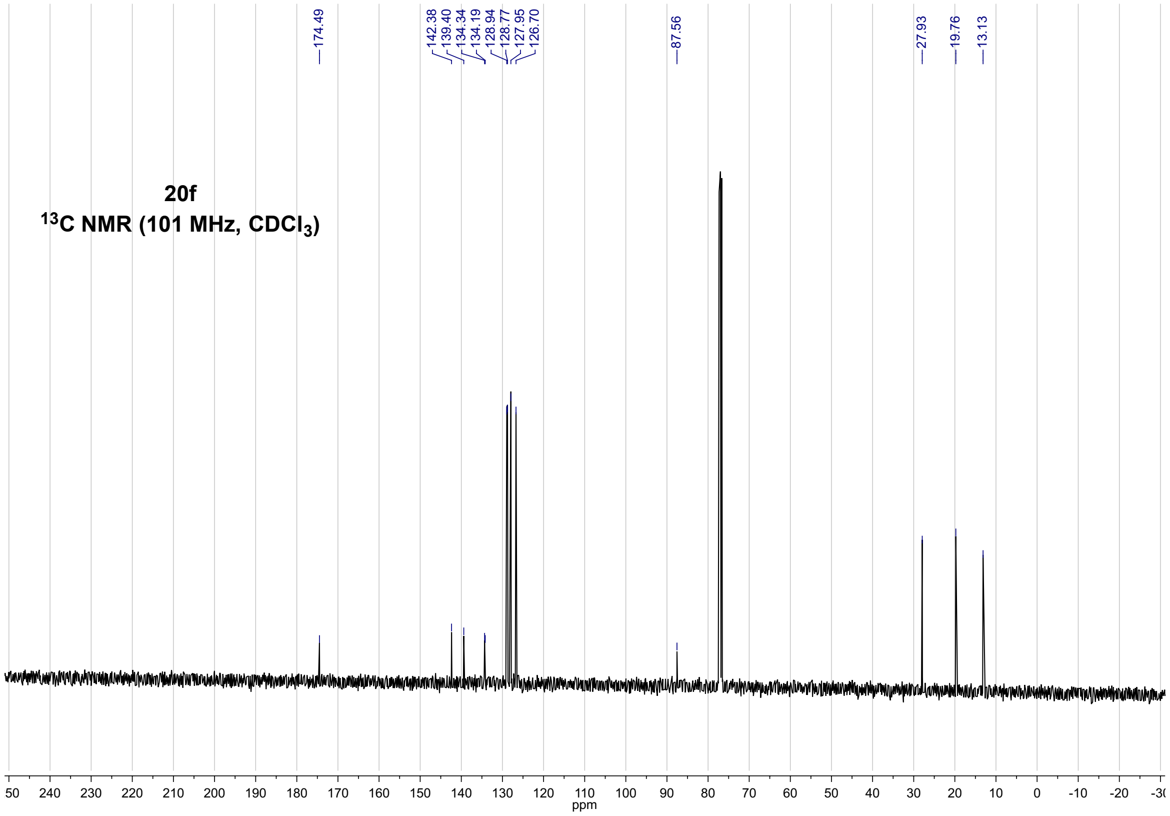
174.49
142.38
139.40
134.34
134.19
128.94
128.77
127.95
126.70
87.56
27.93
19.76
13.13
20f
^{13}C NMR (101 MHz, $CDCl_3$)
ppm

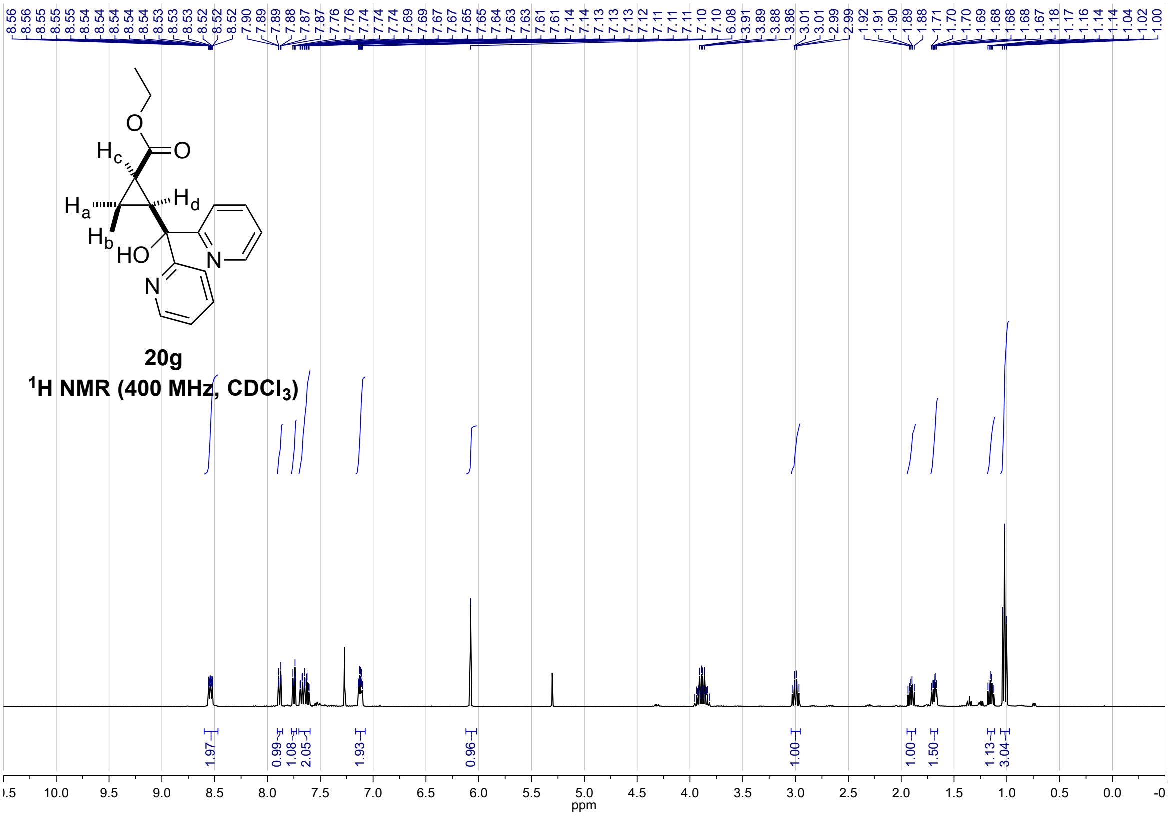
20g
^{1}H NMR (400 MHz, $CDCl_3$)
ppm

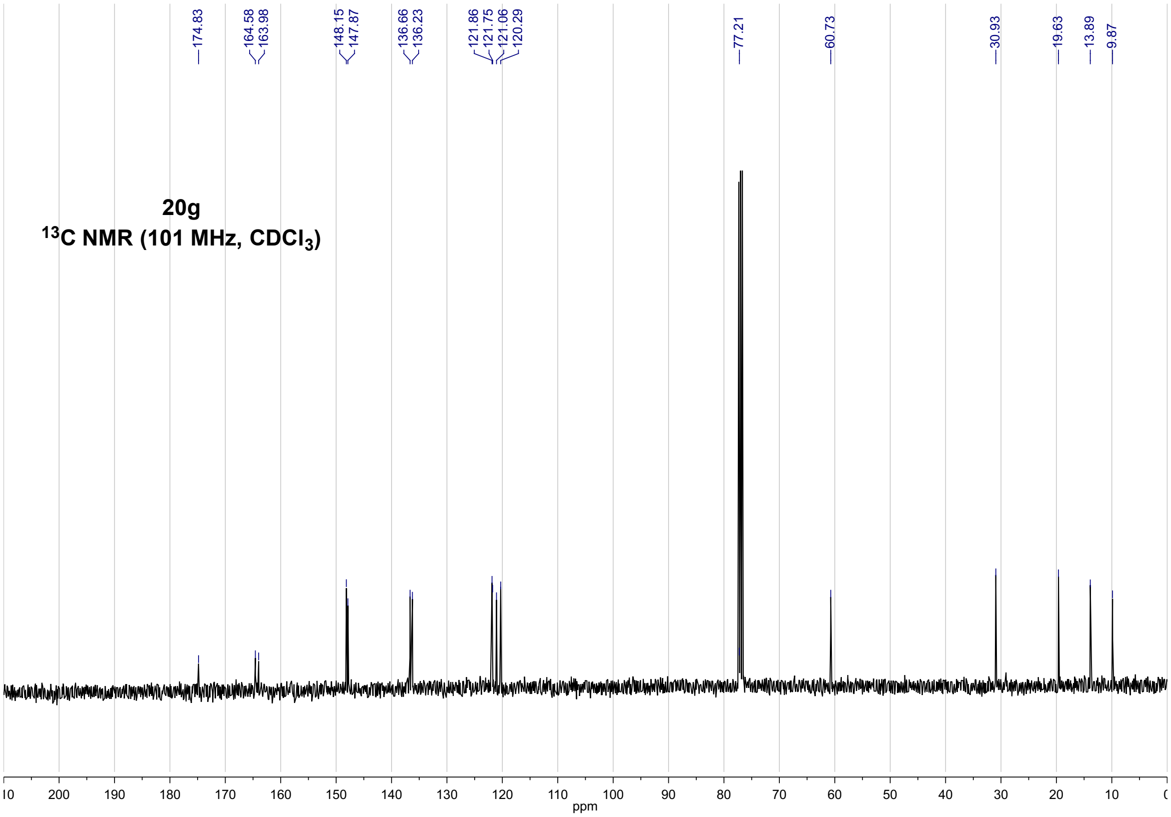
20g
^{13}C NMR (101 MHz, $CDCl_3$)
174.83
164.58
163.98
148.15
147.87
136.66
136.23
121.86
121.75
121.06
120.29
77.21
60.73
30.93
19.63
13.89
9.87
ppm

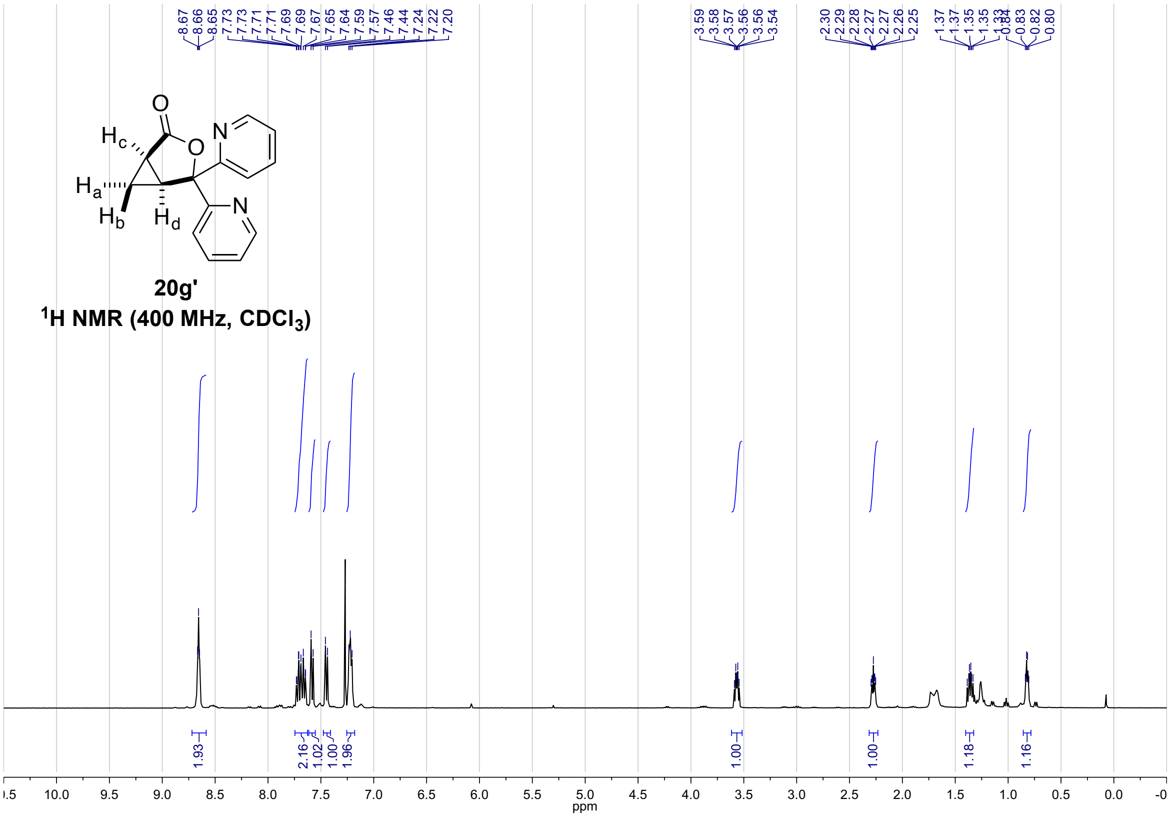

20g'
^{1}H NMR (400 MHz, $CDCl_3$)

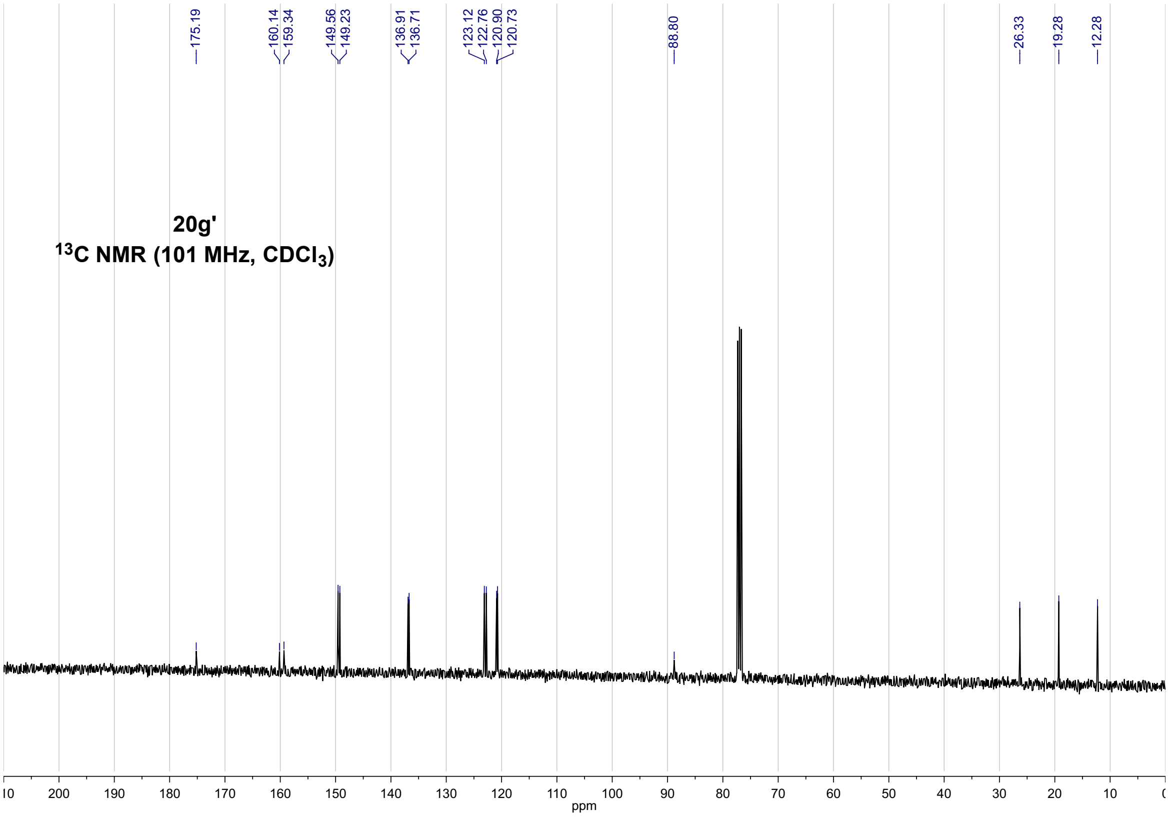

20g'
^{13}C NMR (101 MHz, $CDCl_3$)

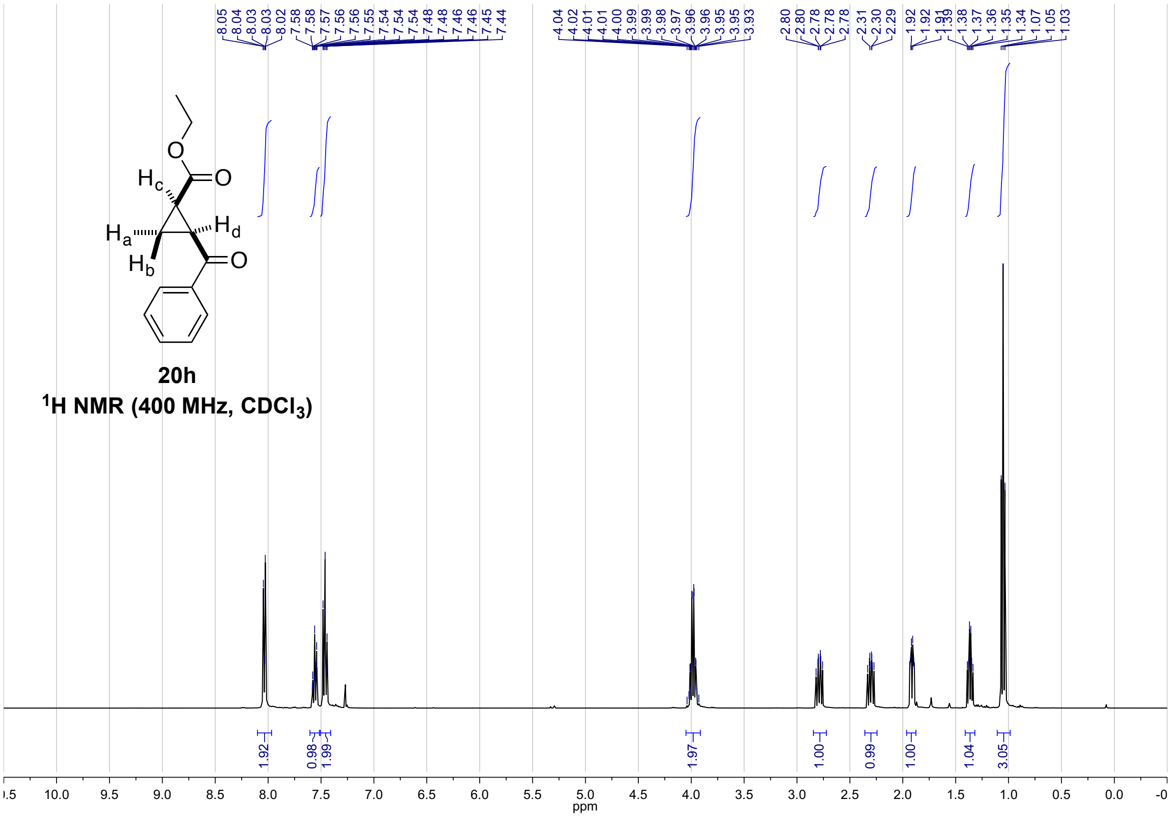

20h
^{1}H NMR (400 MHz, $CDCl_3$)

20h
^{13}C NMR (101 MHz, $CDCl_3$)

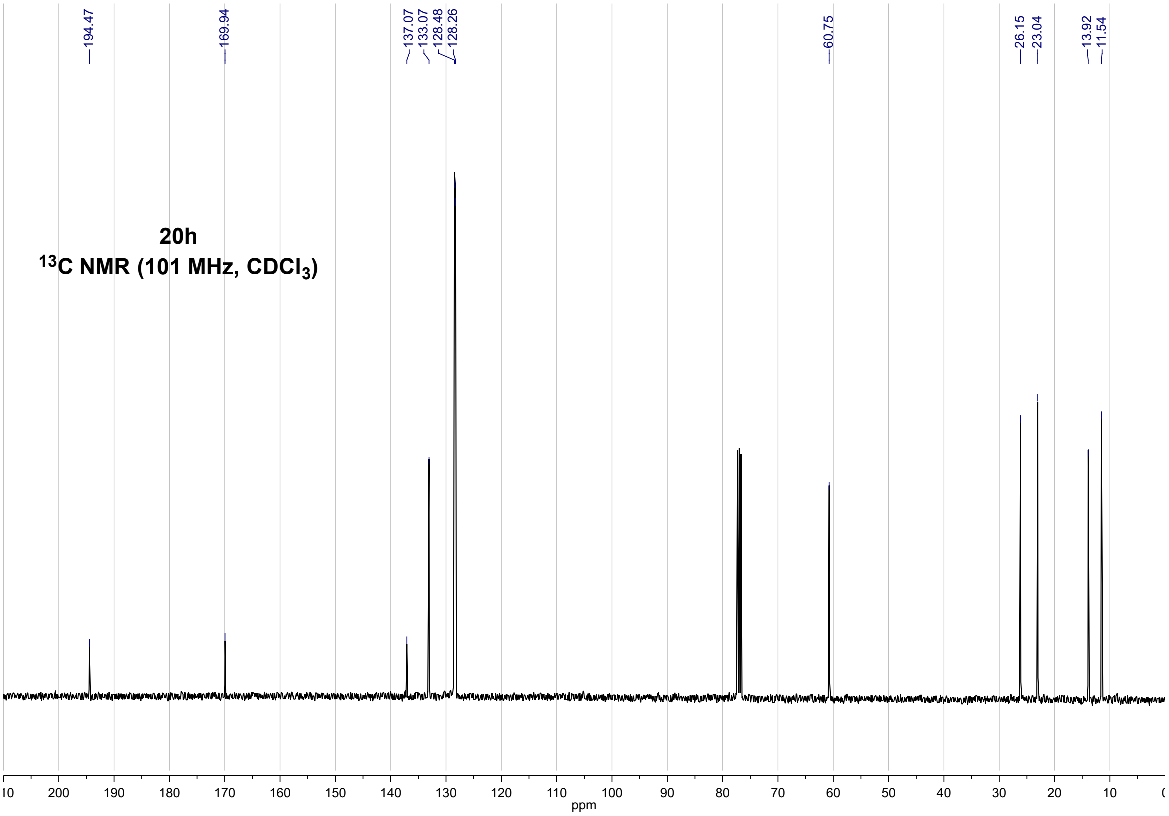

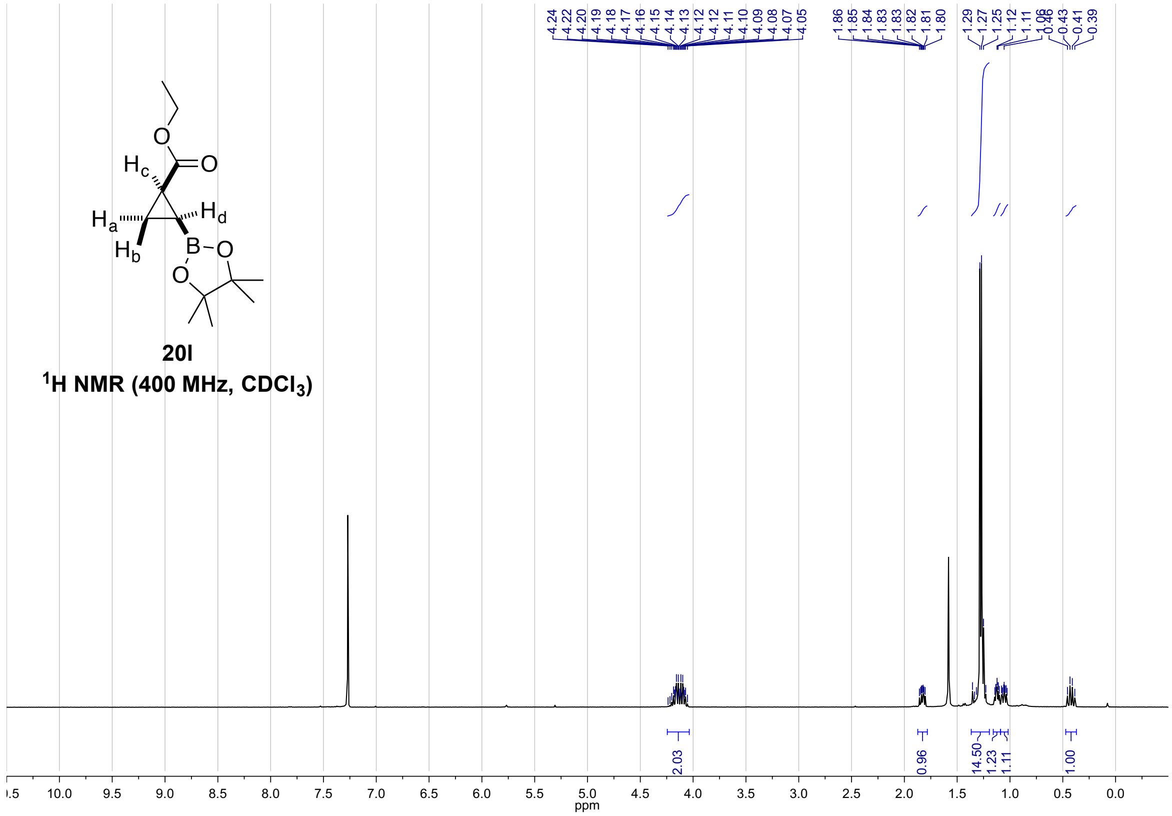

20l

^{1}H NMR (400 MHz, $CDCl_3$)

20l

^{13}C NMR (101 MHz, $CDCl_3$)

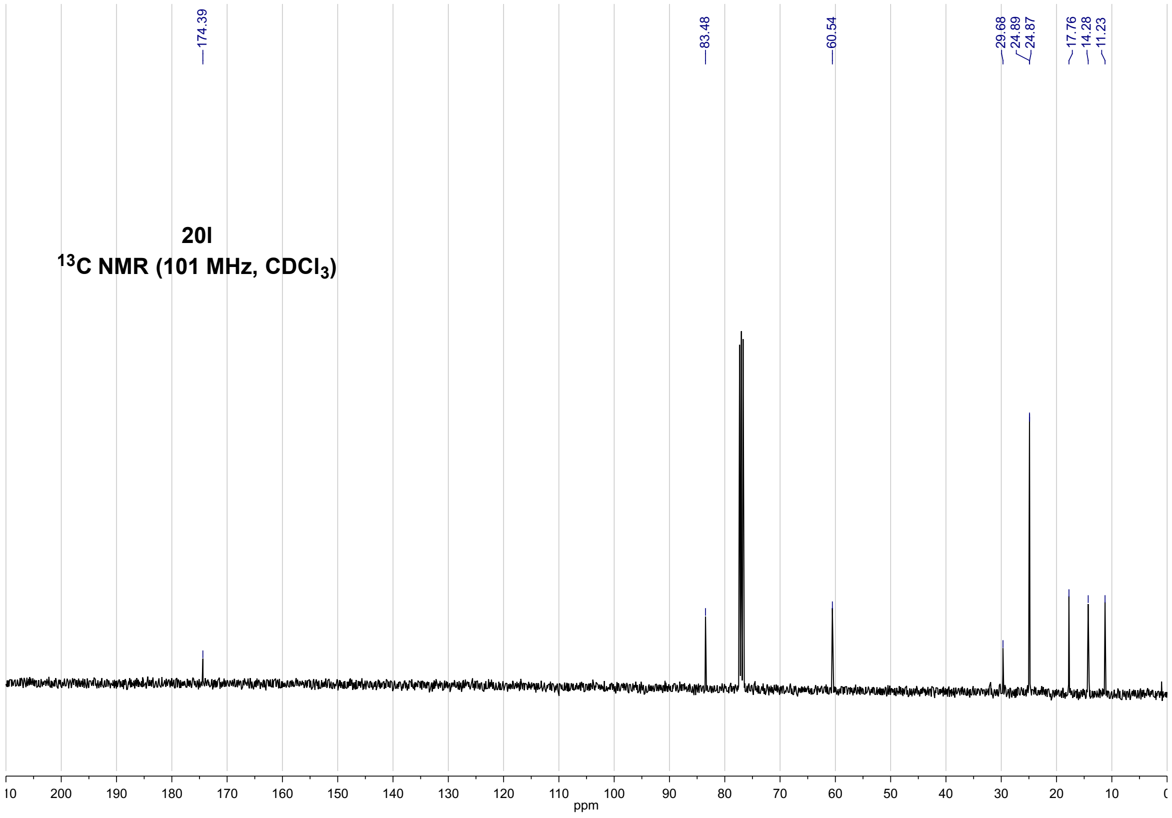

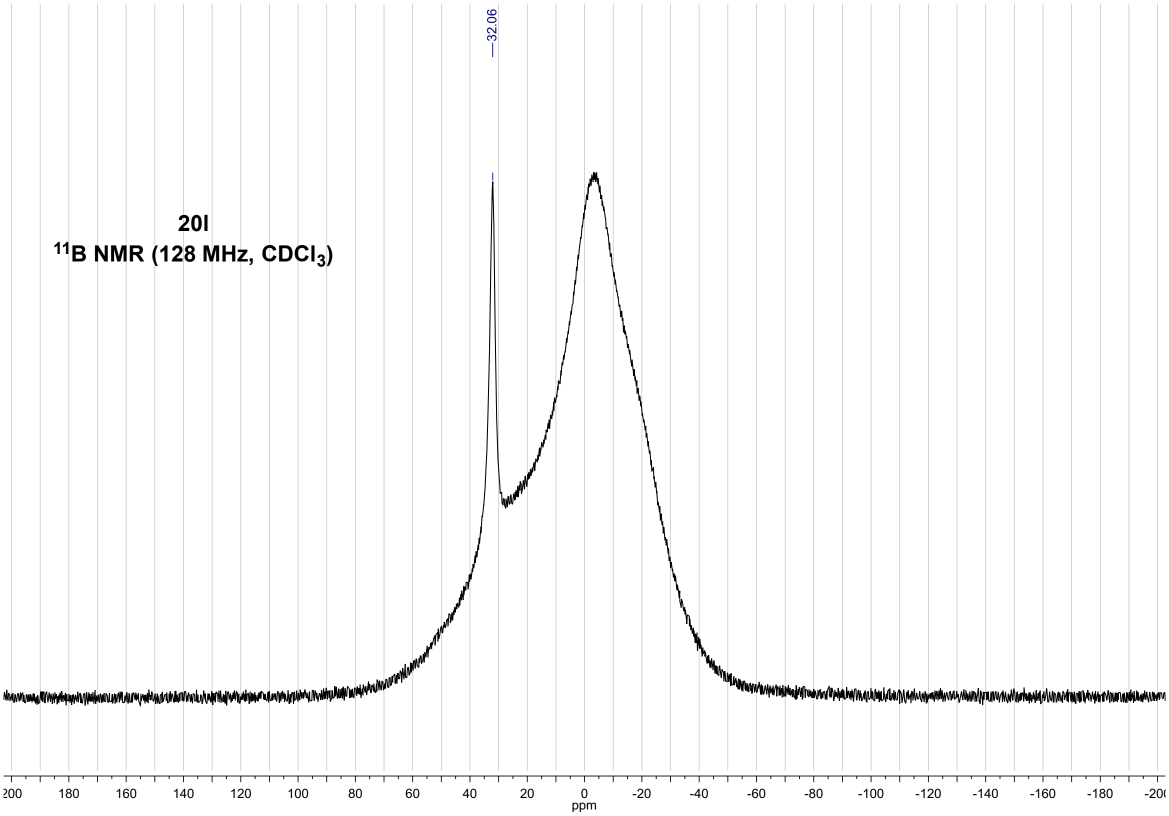
32.06
20l
11B NMR (128 MHz, CDCl3)
200
180
160
140
120
100
80
60
40
20
0
-20
-40
-60
-80
-100
-120
-140
-160
-180
-200
ppm

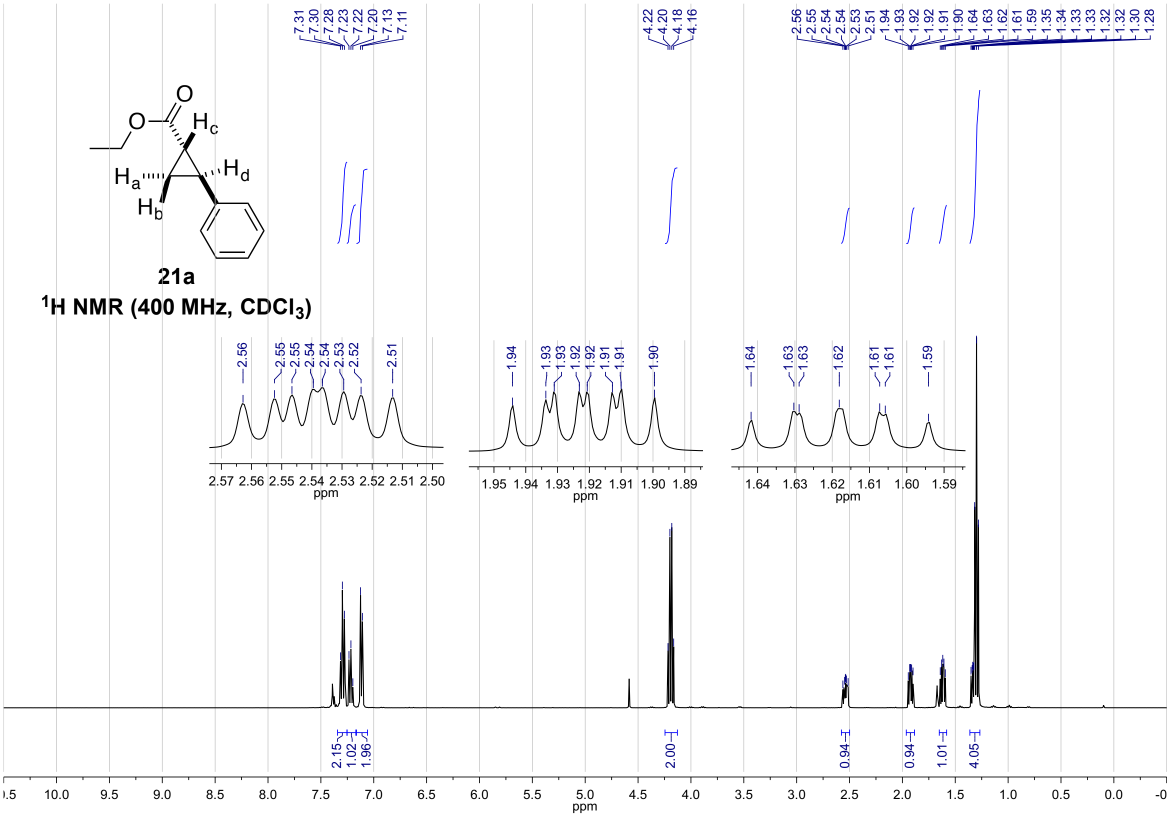
21a
^{1}H NMR (400 MHz, $CDCl_3$)
7.31 7.30 7.28 7.23 7.22 7.20 7.13 7.11
4.22 4.20 4.18 4.16
2.56 2.55 2.54 2.54 2.53 2.51 1.94 1.93 1.92 1.92 1.91 1.90 1.64 1.63 1.62 1.61 1.59 1.35 1.34 1.33 1.33 1.32 1.32 1.30 1.28
2.15 1.02 1.96 2.00 0.94 0.94 1.01 4.05
ppm

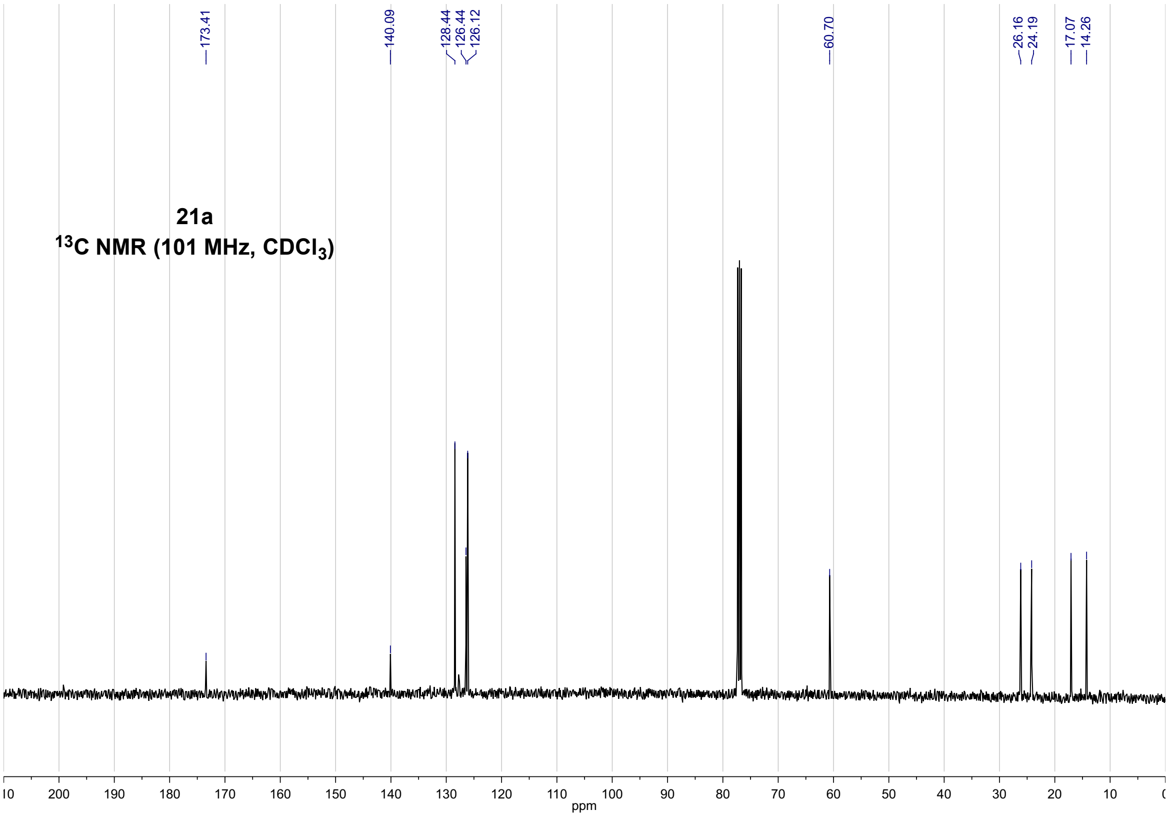
21a
^{13}C NMR (101 MHz, $CDCl_3$)
173.41
140.09
128.44 126.44 126.12
60.70
26.16 24.19
17.07 14.26
ppm

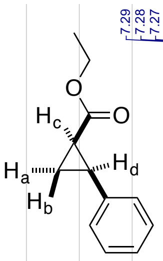

22a

^{1}H NMR (400 MHz, CDCl$_3$)

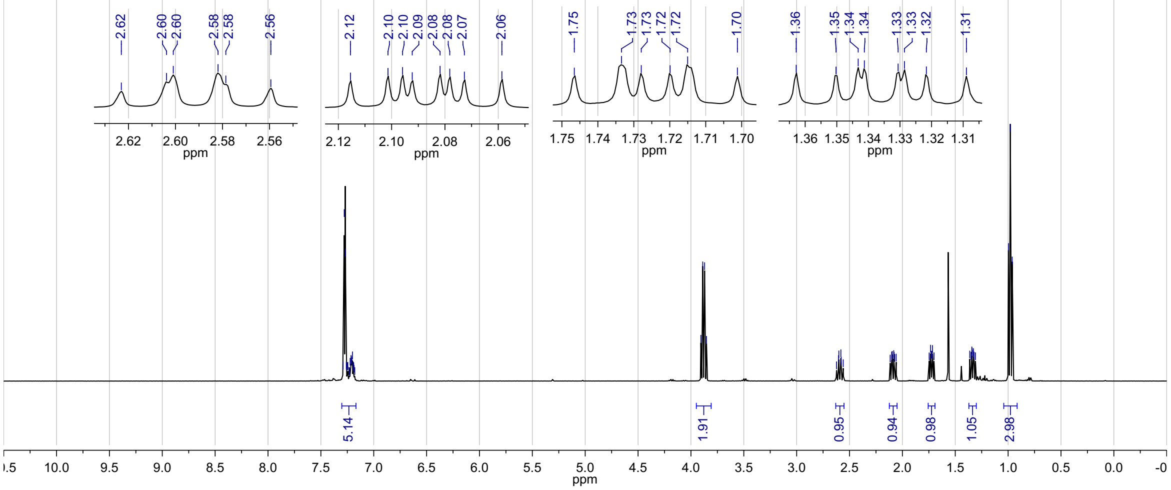

22a

^{13}C NMR (101 MHz, CDCl$_3$)

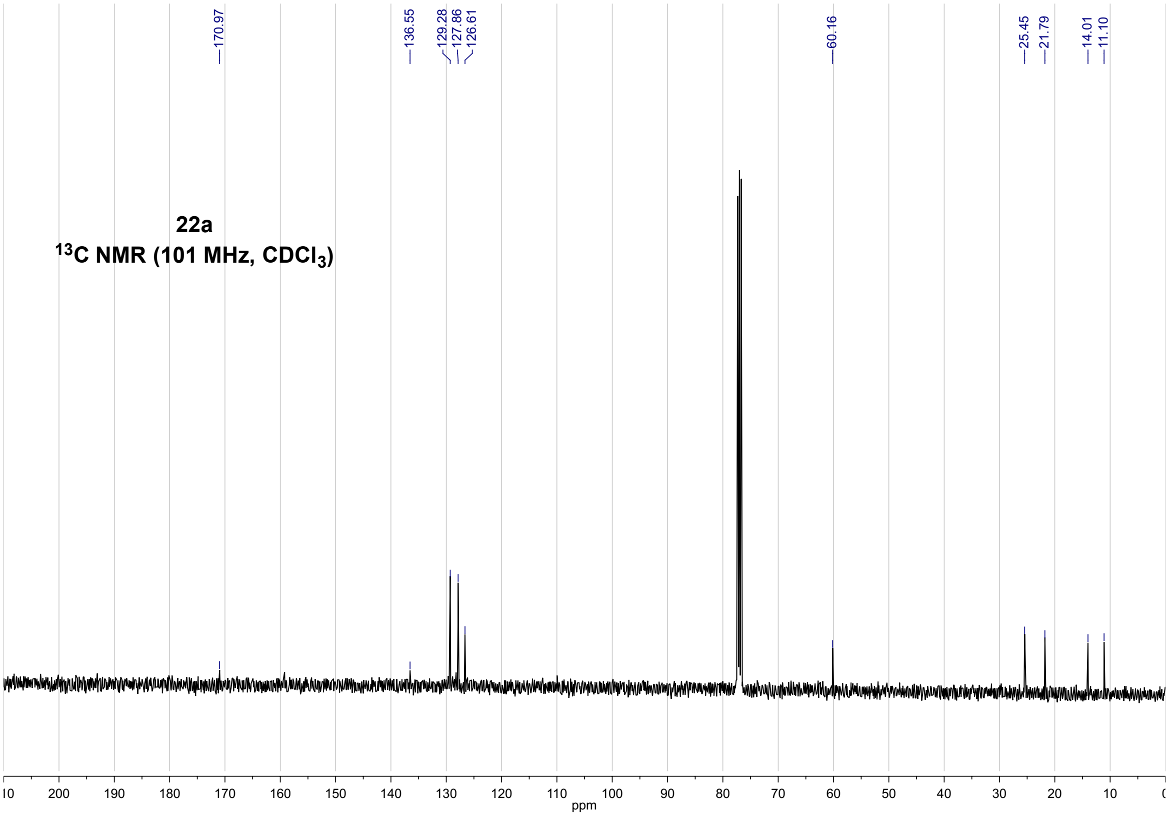

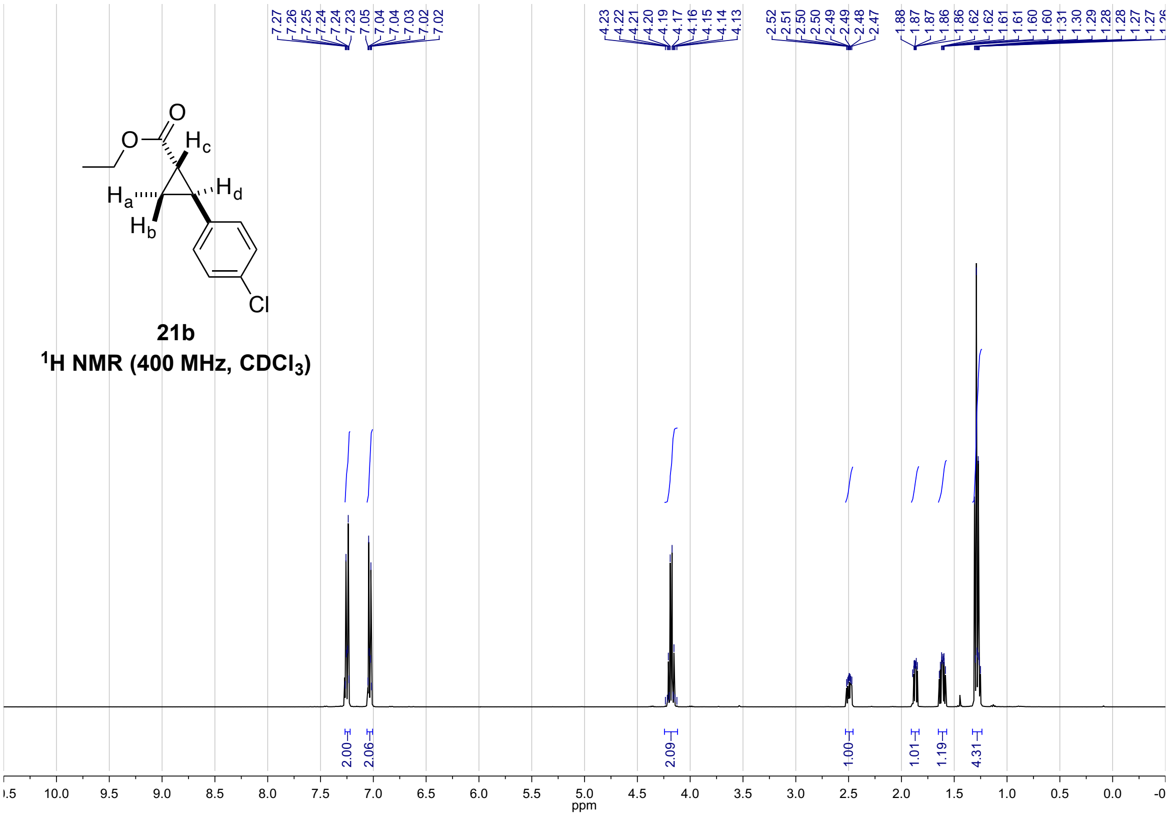
21b
^{1}H NMR (400 MHz, CDCl$_3$)
ppm

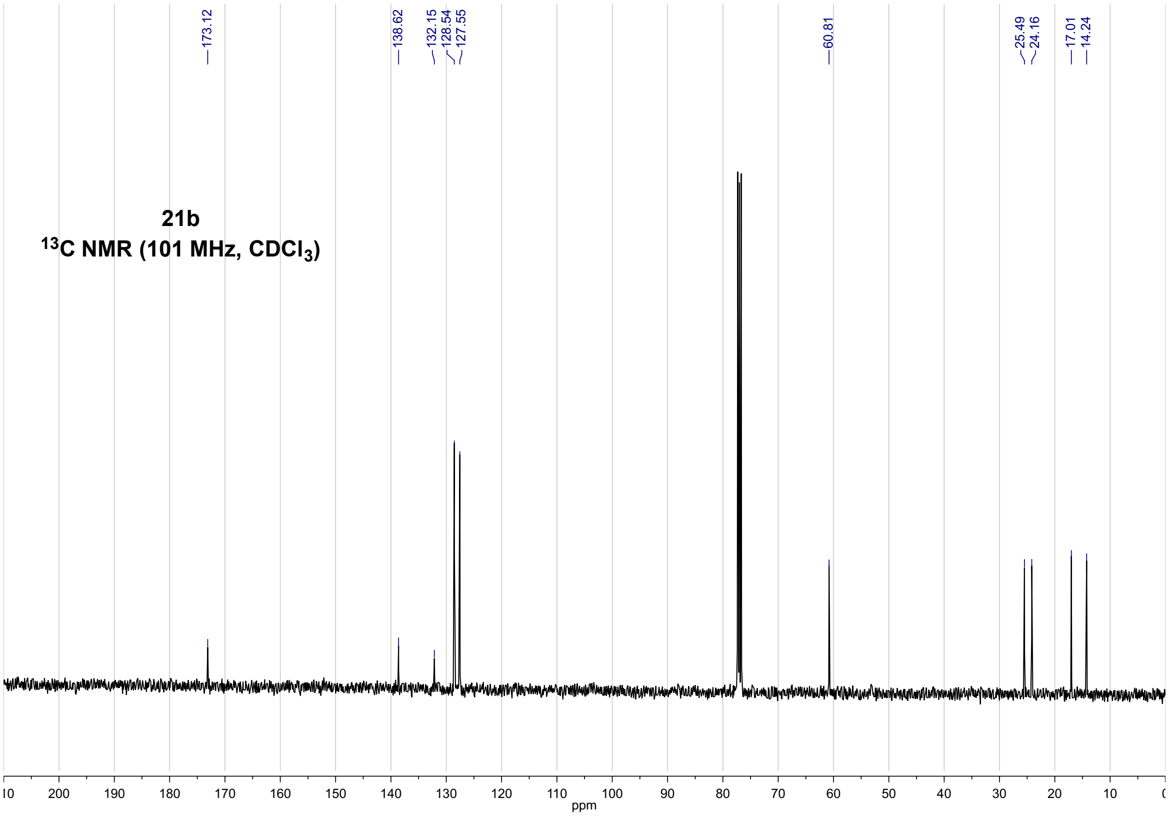
21b
^{13}C NMR (101 MHz, CDCl$_3$)
173.12
138.62
132.15
128.54
127.55
60.81
25.49
24.16
17.01
14.24
ppm

21c
^{1}H NMR (400 MHz, CDCl$_3$)

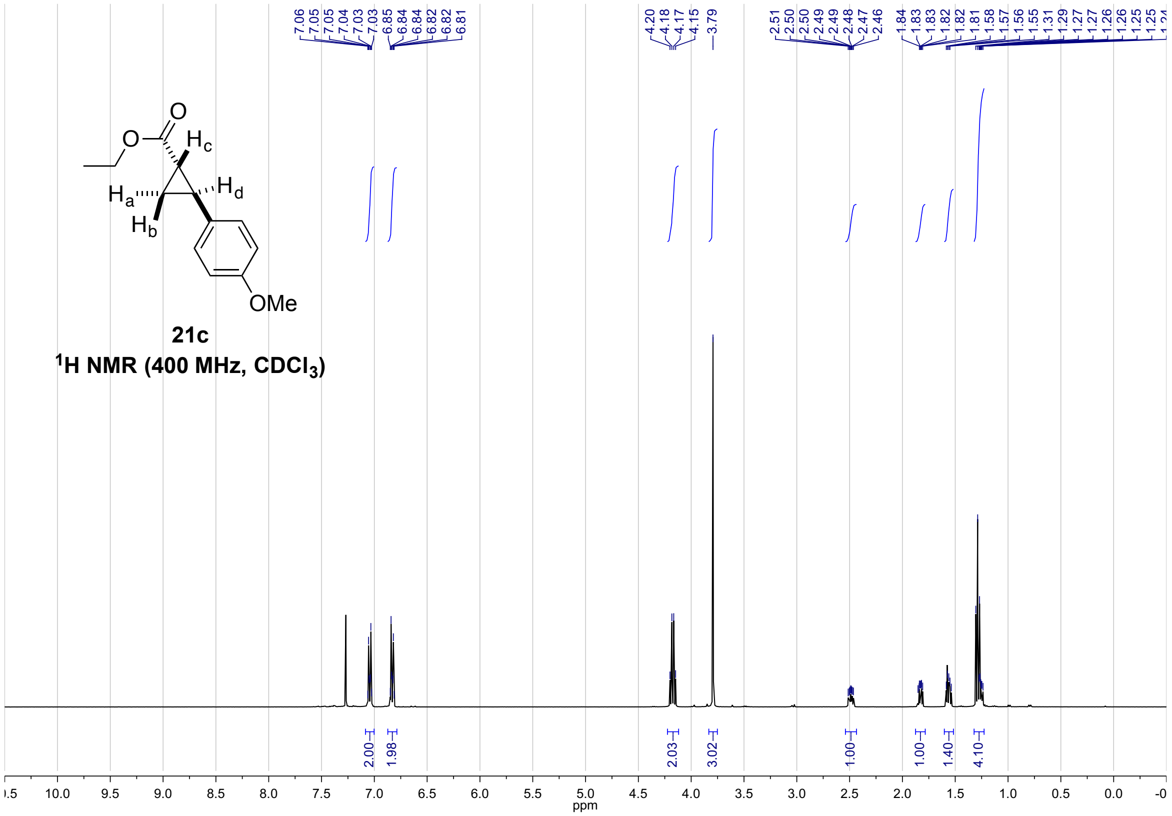

21c
^{13}C NMR (101 MHz, CDCl$_3$)

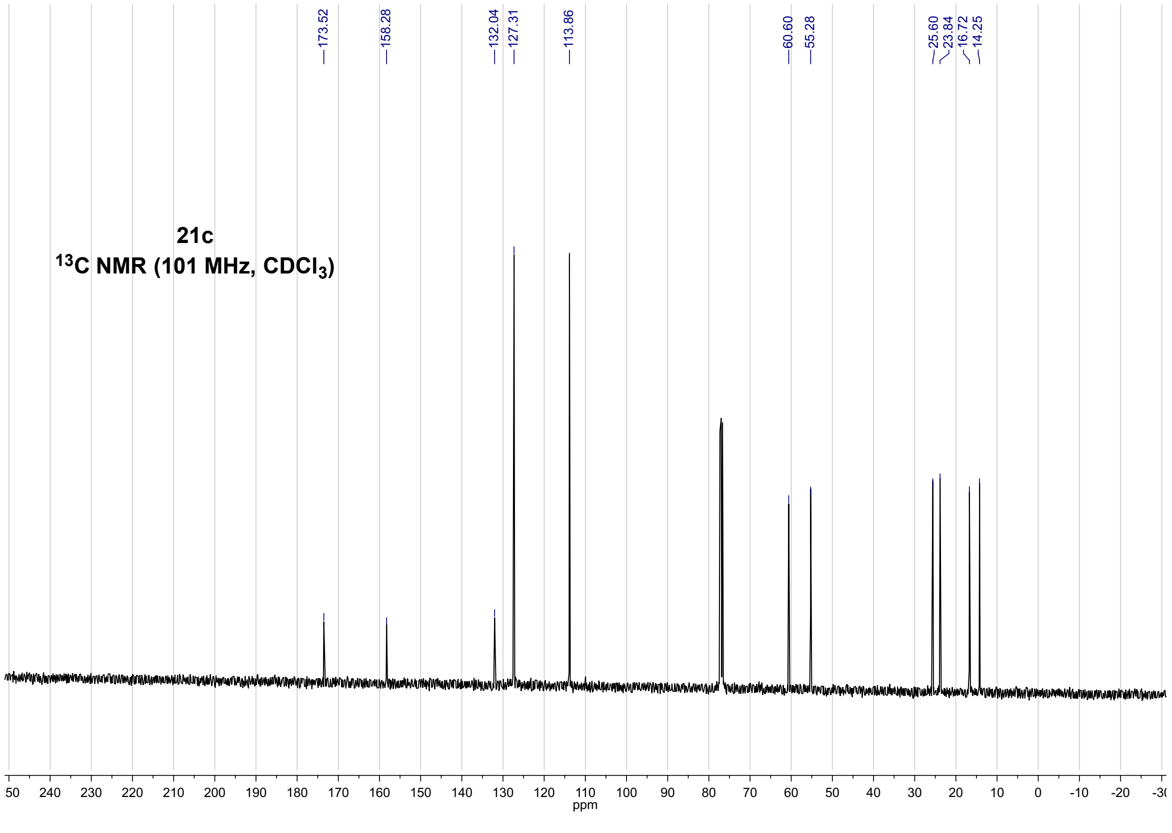

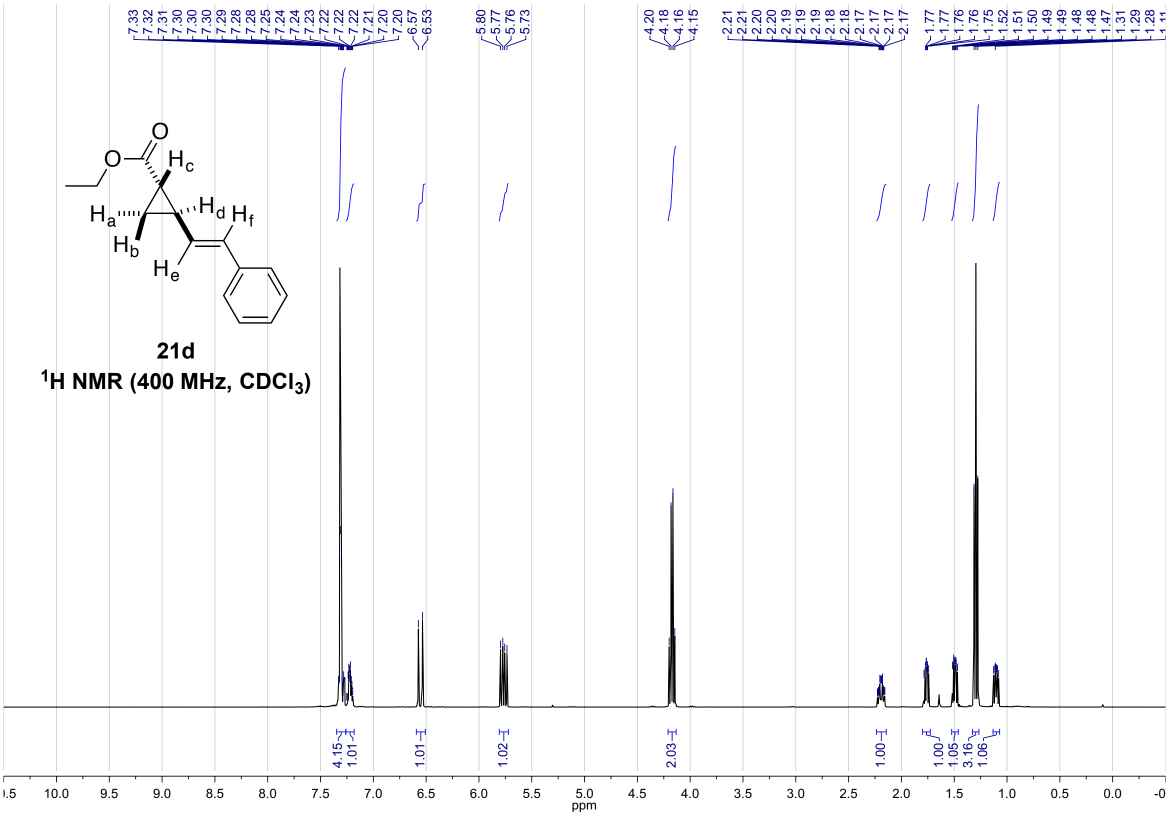

21d
^{1}H NMR (400 MHz, $CDCl_3$)

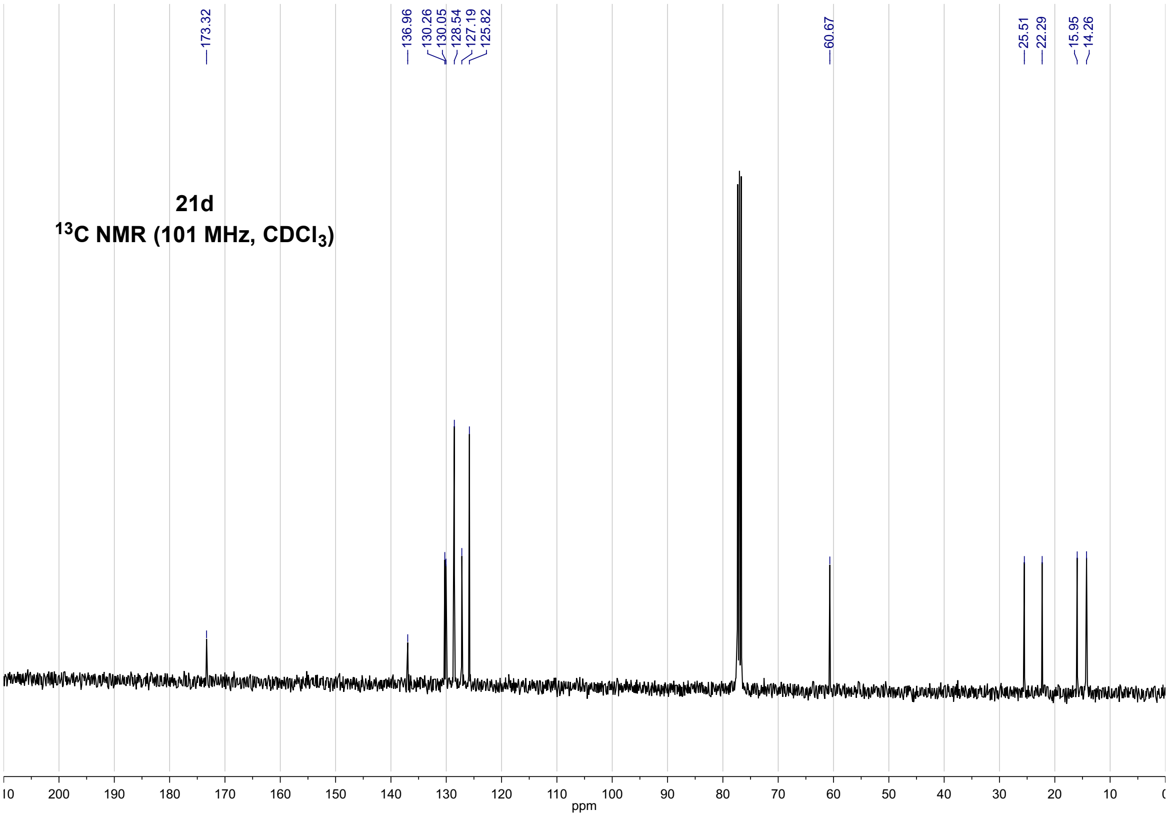

21d
^{13}C NMR (101 MHz, $CDCl_3$)

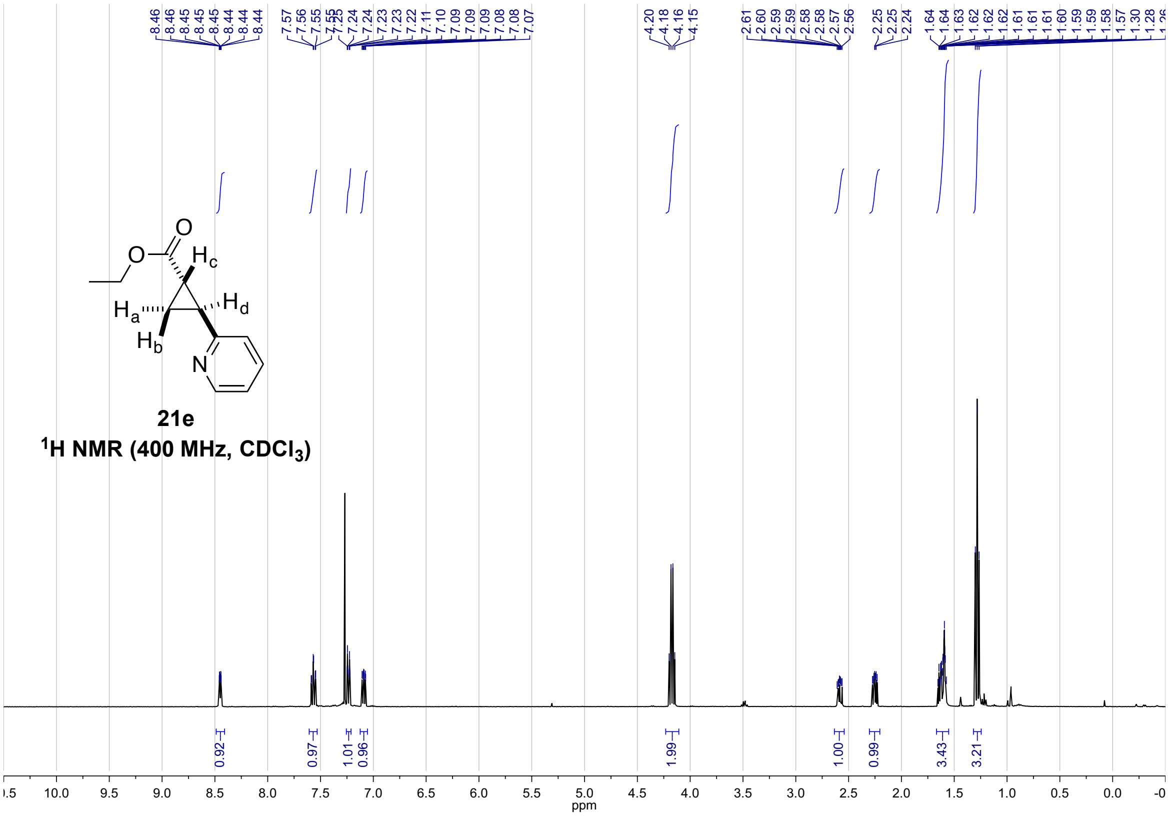
21e
1H NMR (400 MHz, CDCl3)
Ha
Hb
Hc
Hd
O
N
ppm

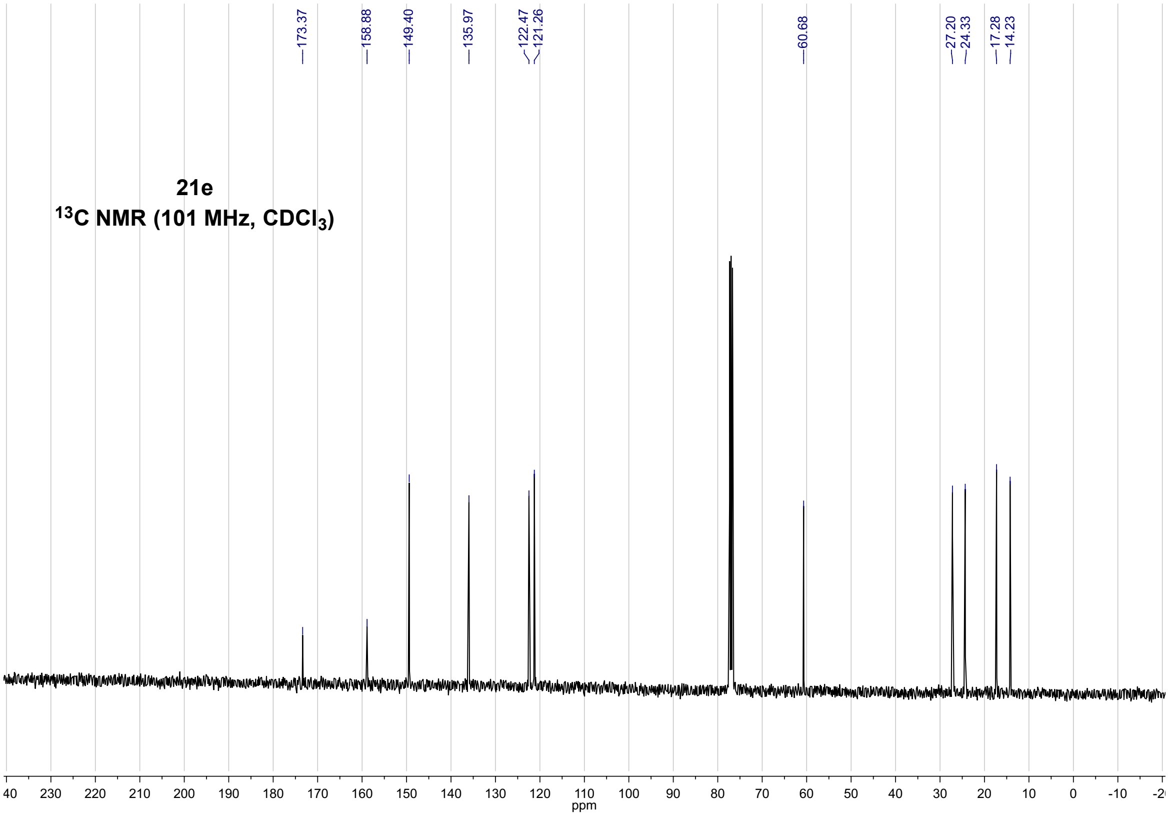
21e
13C NMR (101 MHz, CDCl3)
173.37
158.88
149.40
135.97
122.47
121.26
60.68
27.20
24.33
17.28
14.23
ppm

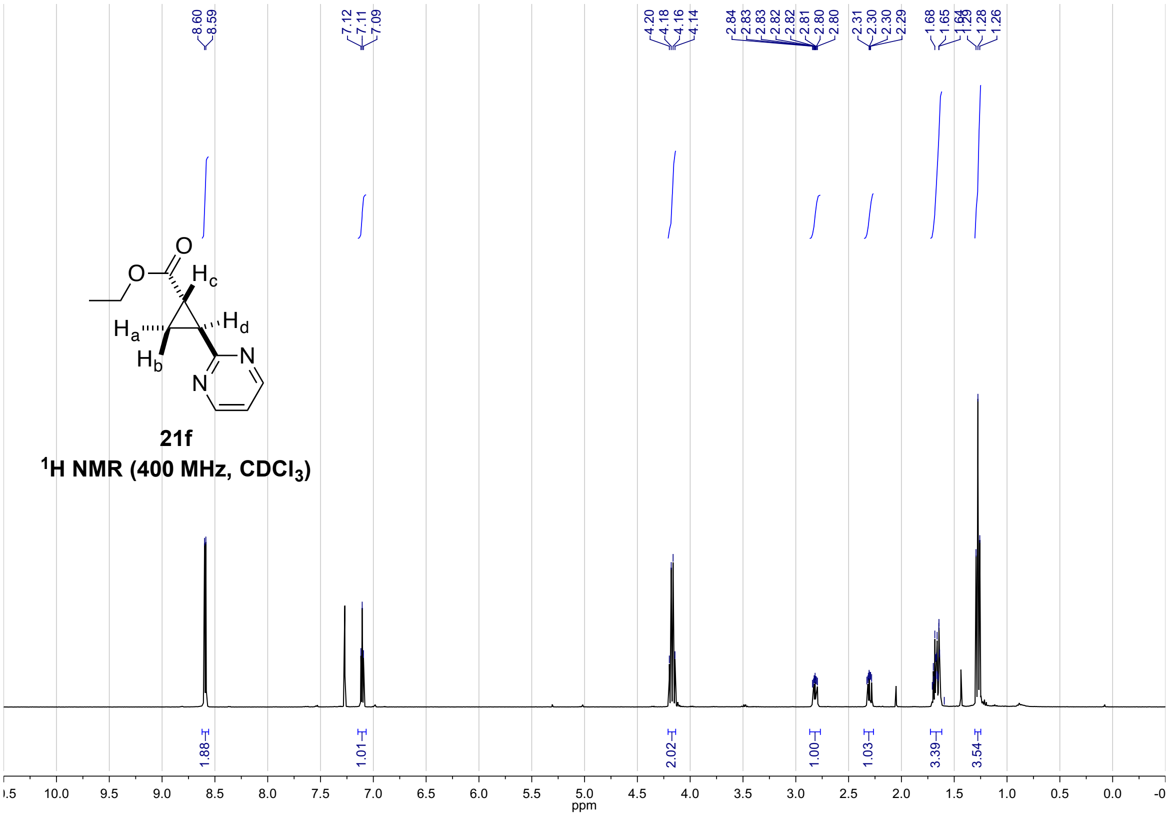

21f

^{1}H NMR (400 MHz, $CDCl_3$)

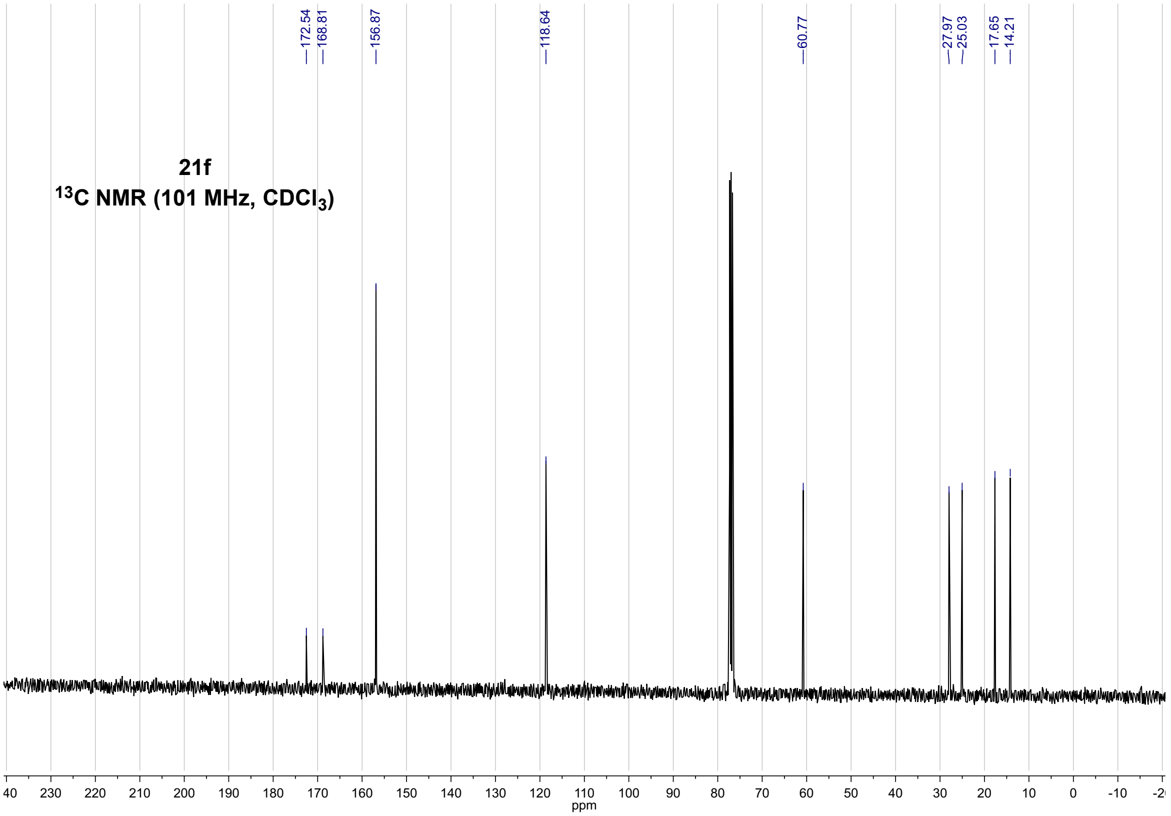

21f

^{13}C NMR (101 MHz, $CDCl_3$)

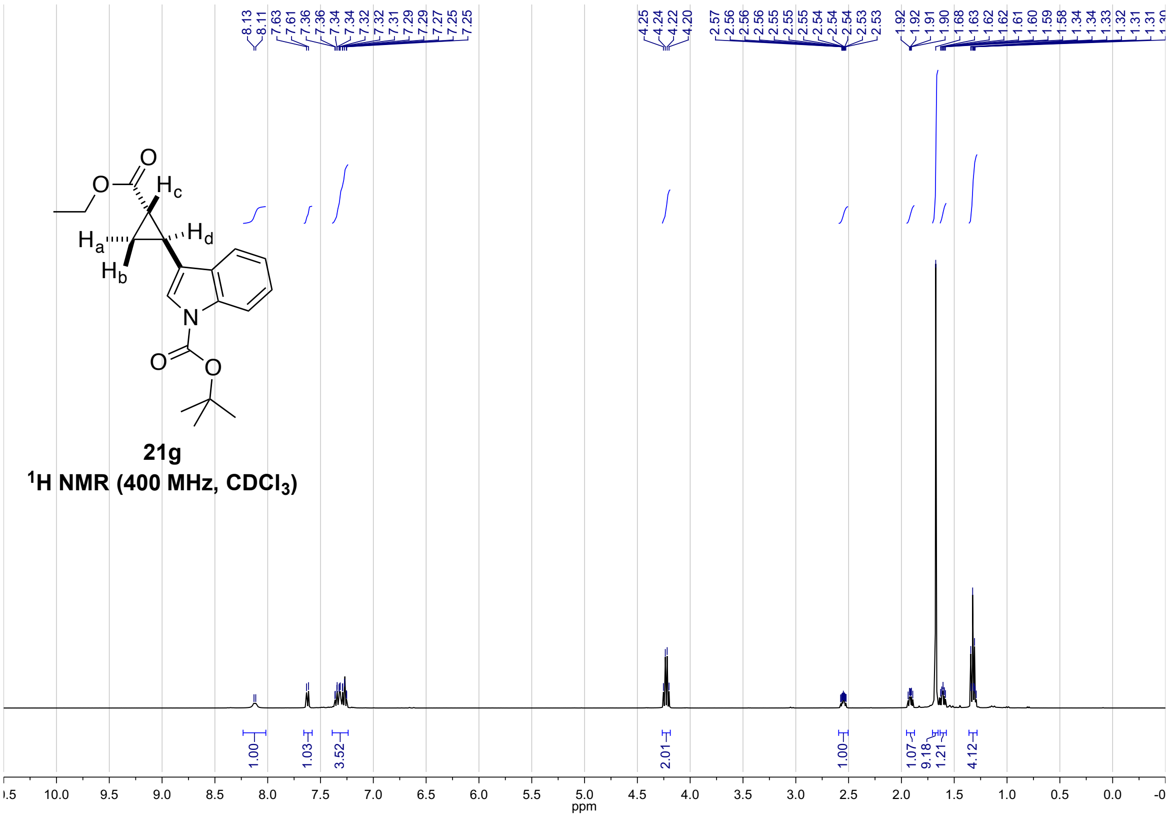
21g
1H NMR (400 MHz, CDCl3)
8.13 8.11 7.63 7.61 7.36 7.34 7.34 7.32 7.31 7.29 7.27 7.25 7.25
4.25 4.24 4.22 4.20
2.57 2.56 2.56 2.55 2.55 2.55 2.54 2.54 2.54 2.53 2.53
1.92 1.92 1.91 1.90 1.68 1.63 1.62 1.62 1.61 1.60 1.59 1.58 1.34 1.34 1.33 1.32 1.31 1.31
1.00 1.03 3.52 2.01 1.00 1.07 9.18 1.21 4.12
ppm

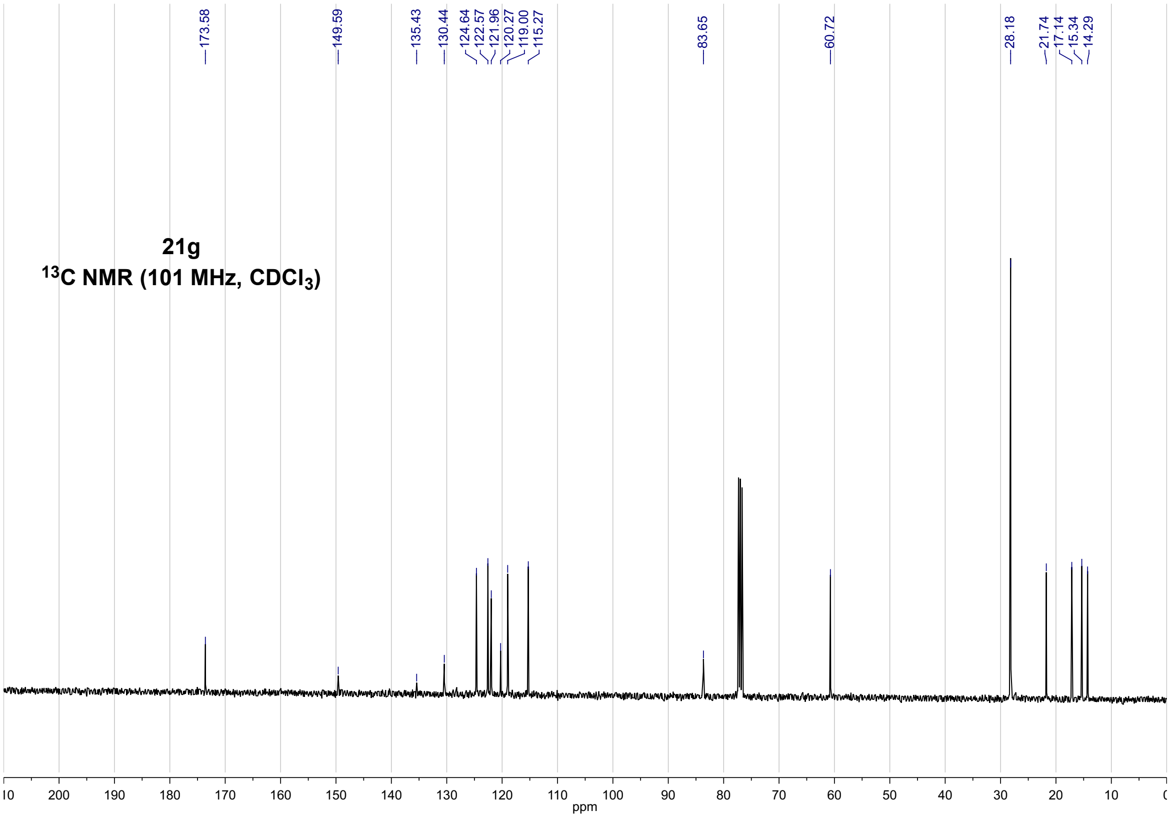
21g
13C NMR (101 MHz, CDCl3)
173.58
149.59
135.43 130.44 124.64 122.57 121.96 120.27 119.00 115.27
83.65
60.72
28.18
21.74 17.14 15.34 14.29
ppm

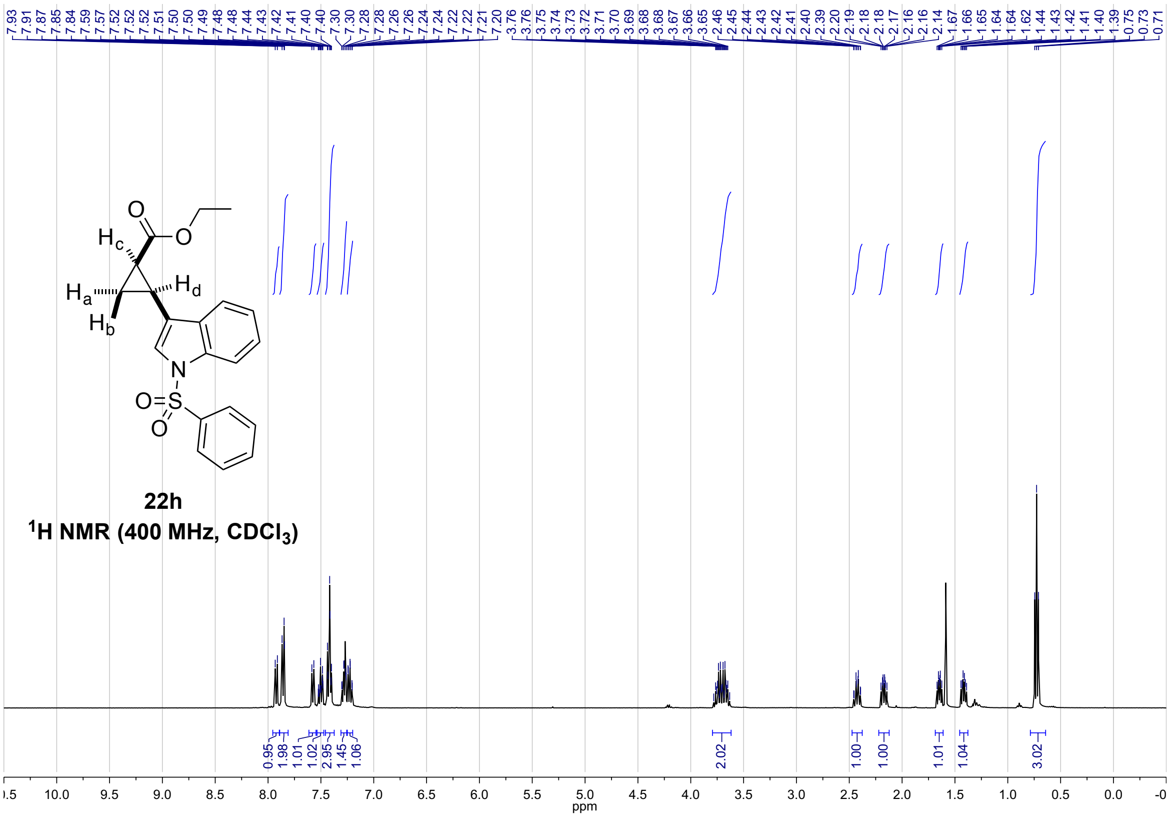

7.93 7.91 7.87 7.85 7.84 7.59 7.57 7.52 7.52 7.52 7.51 7.50 7.50 7.49 7.48 7.48 7.44 7.43 7.42 7.41 7.40 7.40 7.30 7.30 7.28 7.26 7.26 7.24 7.24 7.22 7.22 7.21 7.20 3.76 3.76 3.75 3.74 3.73 3.72 3.71 3.70 3.69 3.68 3.67 3.66 3.65 2.46 2.45 2.44 2.43 2.42 2.41 2.40 2.39 2.20 2.19 2.18 2.18 2.17 2.16 2.16 2.14 1.67 1.66 1.65 1.64 1.64 1.62 1.44 1.43 1.42 1.41 1.40 1.39 0.75 0.73 0.71
Hc
Ha
Hb
Hd
O
O
N
O=S
O
22h
1H NMR (400 MHz, CDCl3)
0.95 1.98 1.01 1.02 2.95 1.45 1.06 2.02 1.00 1.00 1.01 1.04 3.02
ppm

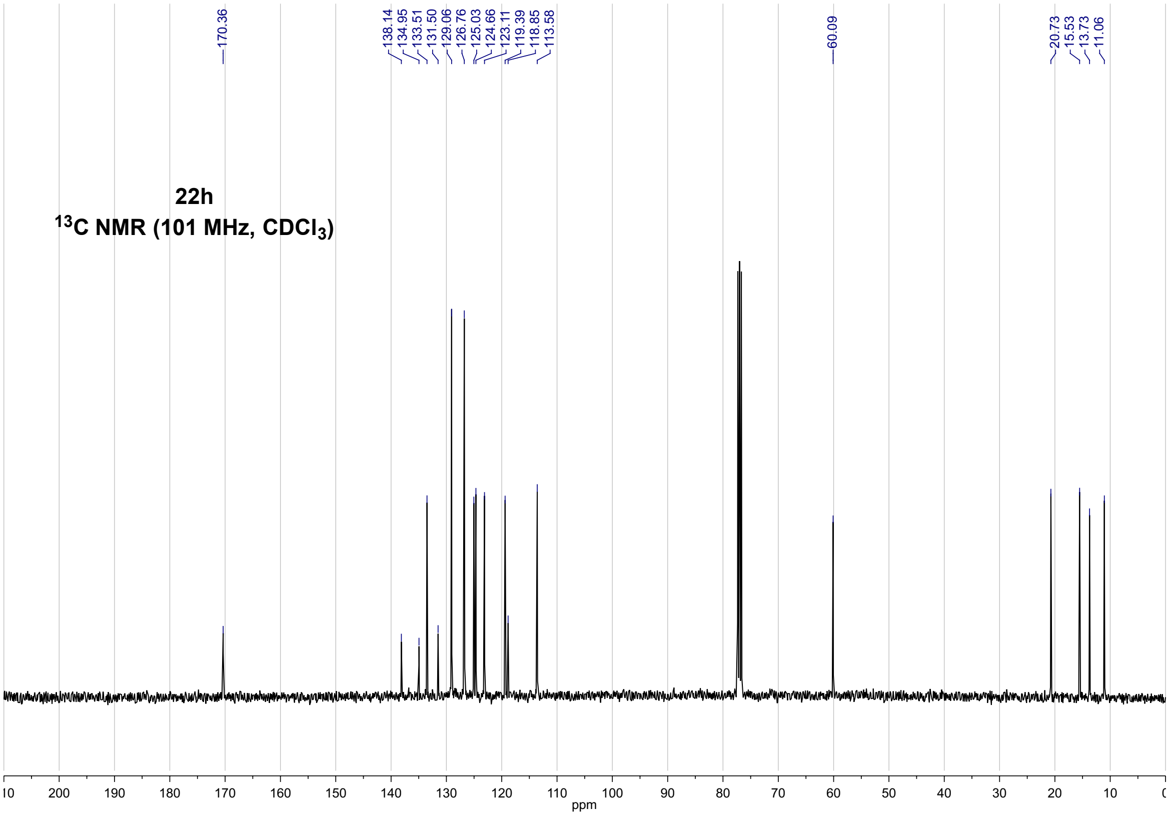

170.36
138.14 134.95 133.51 131.50 129.06 126.76 125.03 124.66 123.11 119.39 118.85 113.58
60.09
20.73 15.53 13.73 11.06
22h
13C NMR (101 MHz, CDCl3)
ppm

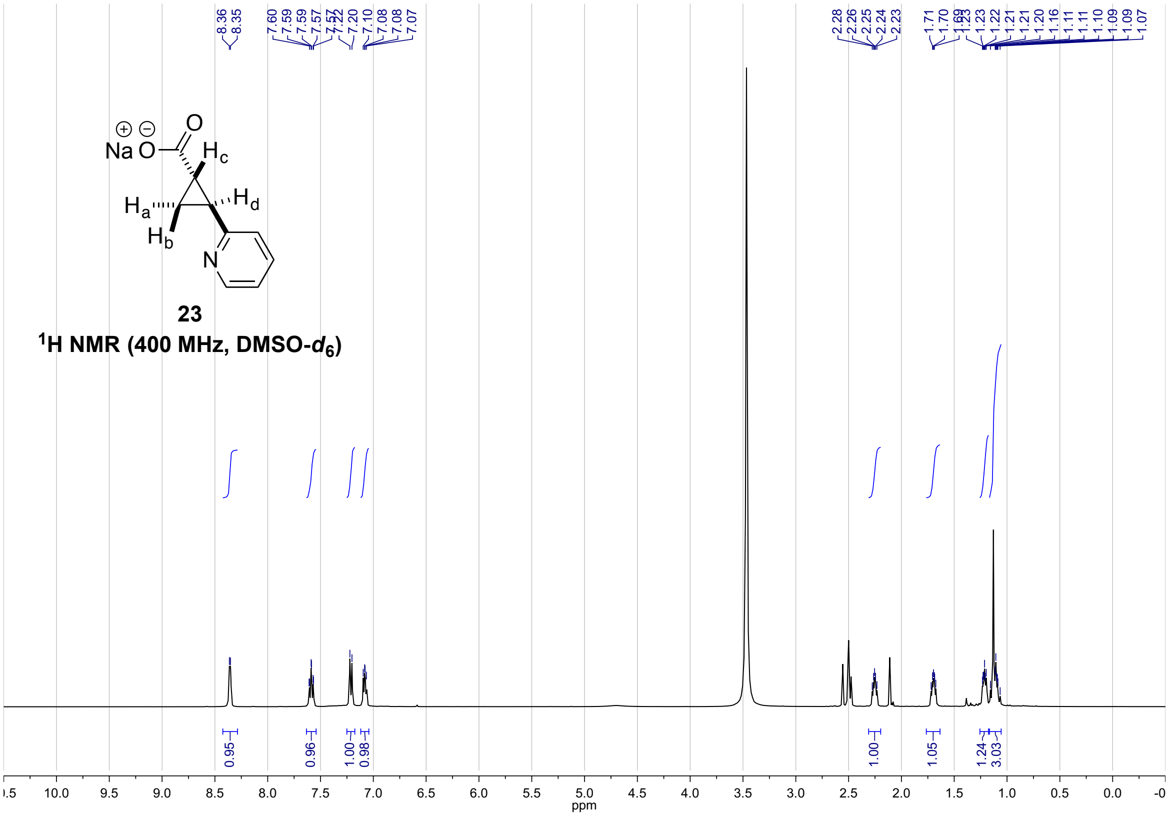
8.36
8.35
7.60
7.59
7.59
7.57
7.22
7.20
7.10
7.08
7.08
7.07
2.28
2.26
2.25
2.24
2.23
1.71
1.70
1.23
1.22
1.21
1.21
1.20
1.16
1.11
1.11
1.10
1.09
1.09
1.07
Na O
O
H_c
H_d
H_a
H_b
N
23
^{1}H NMR (400 MHz, DMSO-d_6)
0.95
0.96
1.00
0.98
1.00
1.05
1.24
3.03
ppm

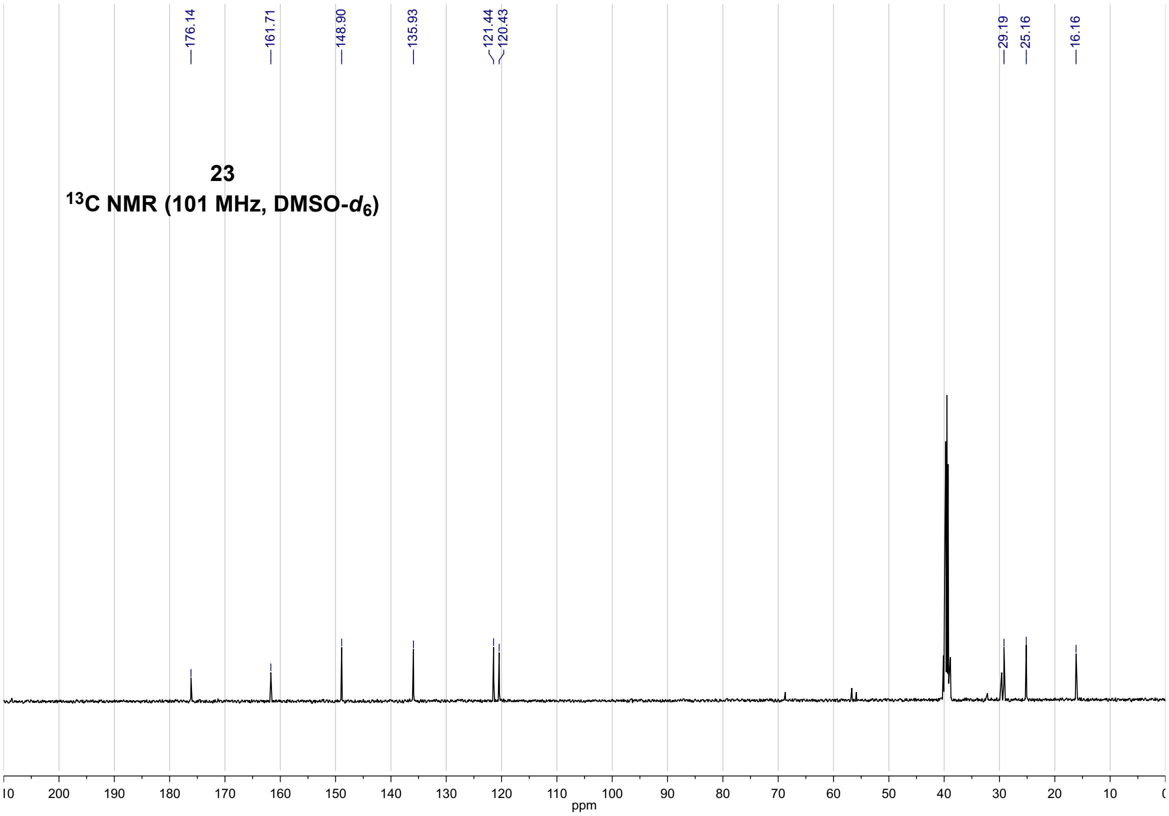
176.14
161.71
148.90
135.93
121.44
120.43
29.19
25.16
16.16
23
^{13}C NMR (101 MHz, DMSO-d_6)
ppm

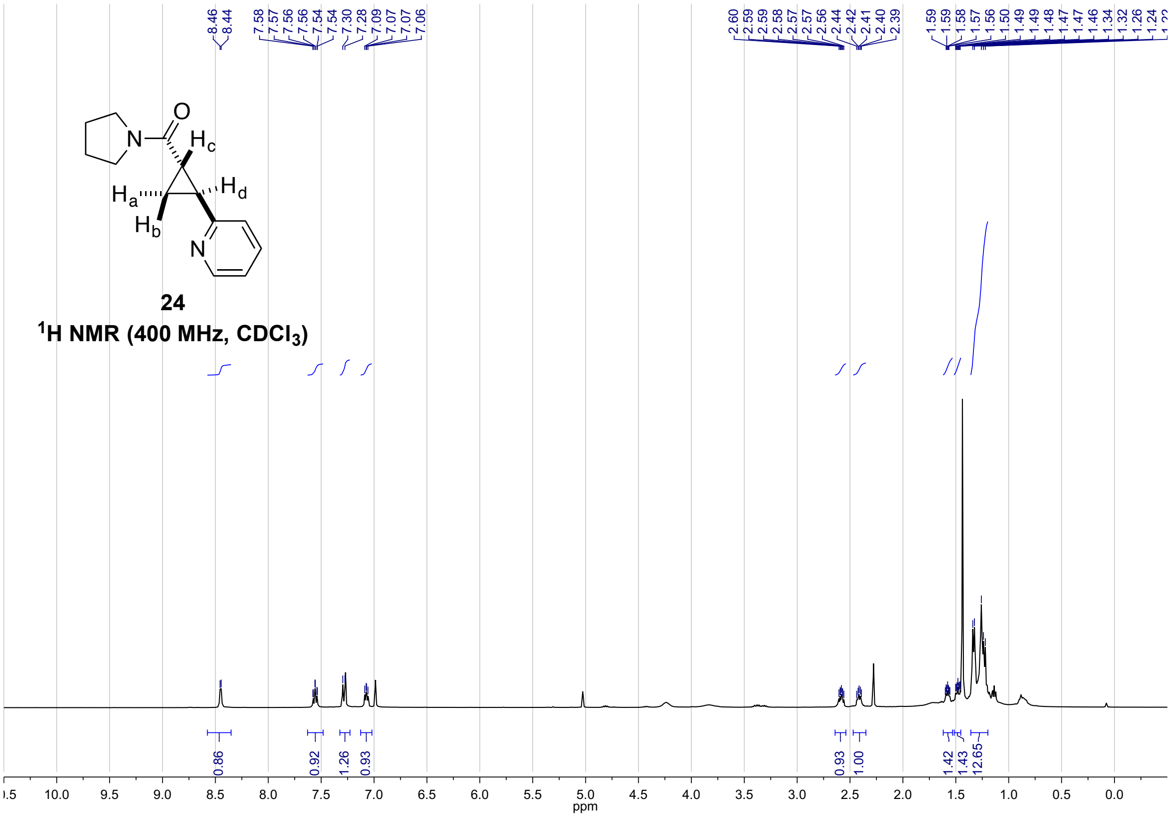

24

^{1}H NMR (400 MHz, $CDCl_3$)

24

^{13}C NMR (101 MHz, $CDCl_3$)

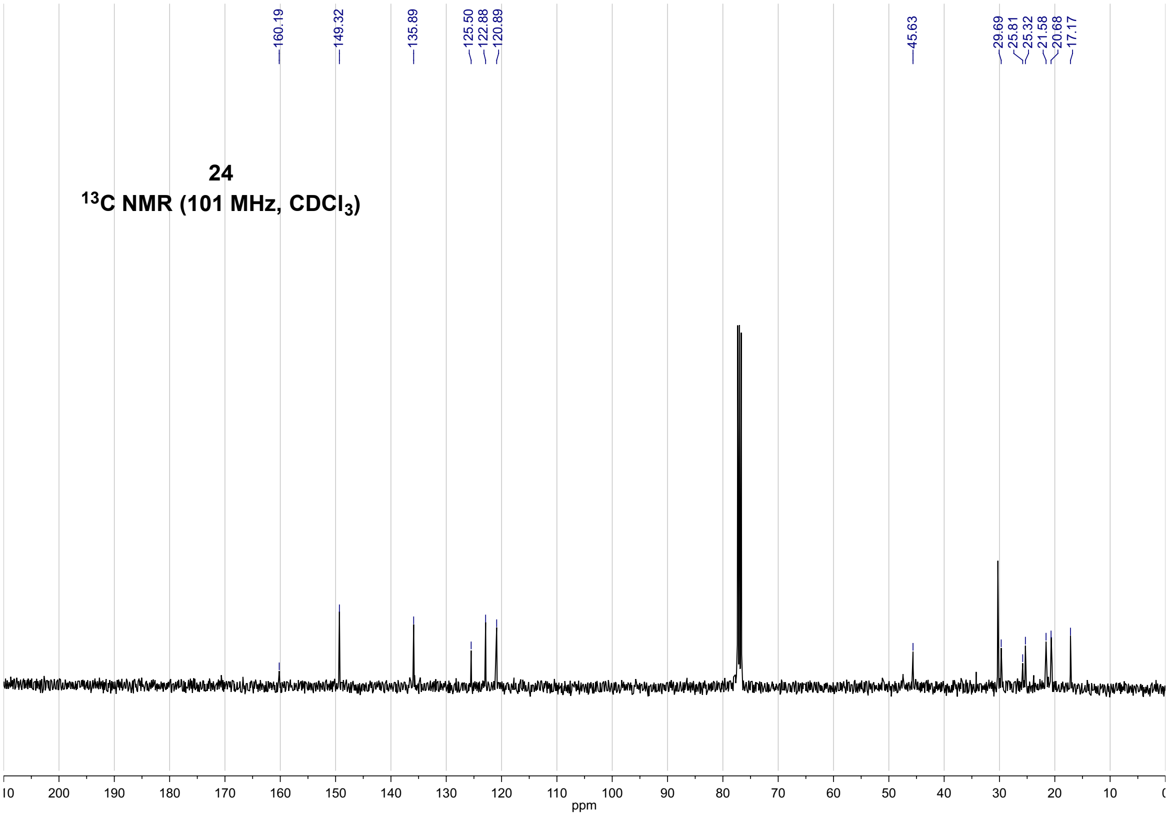

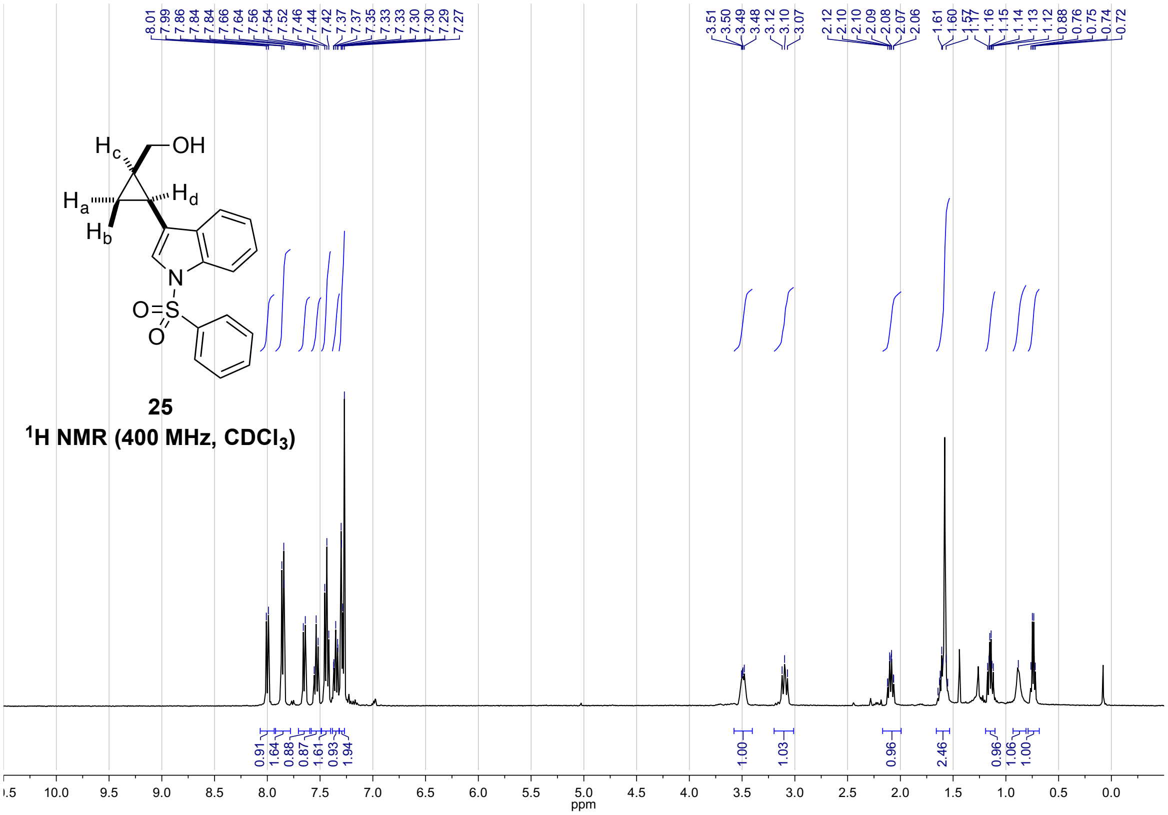

25

^{1}H NMR (400 MHz, $CDCl_3$)

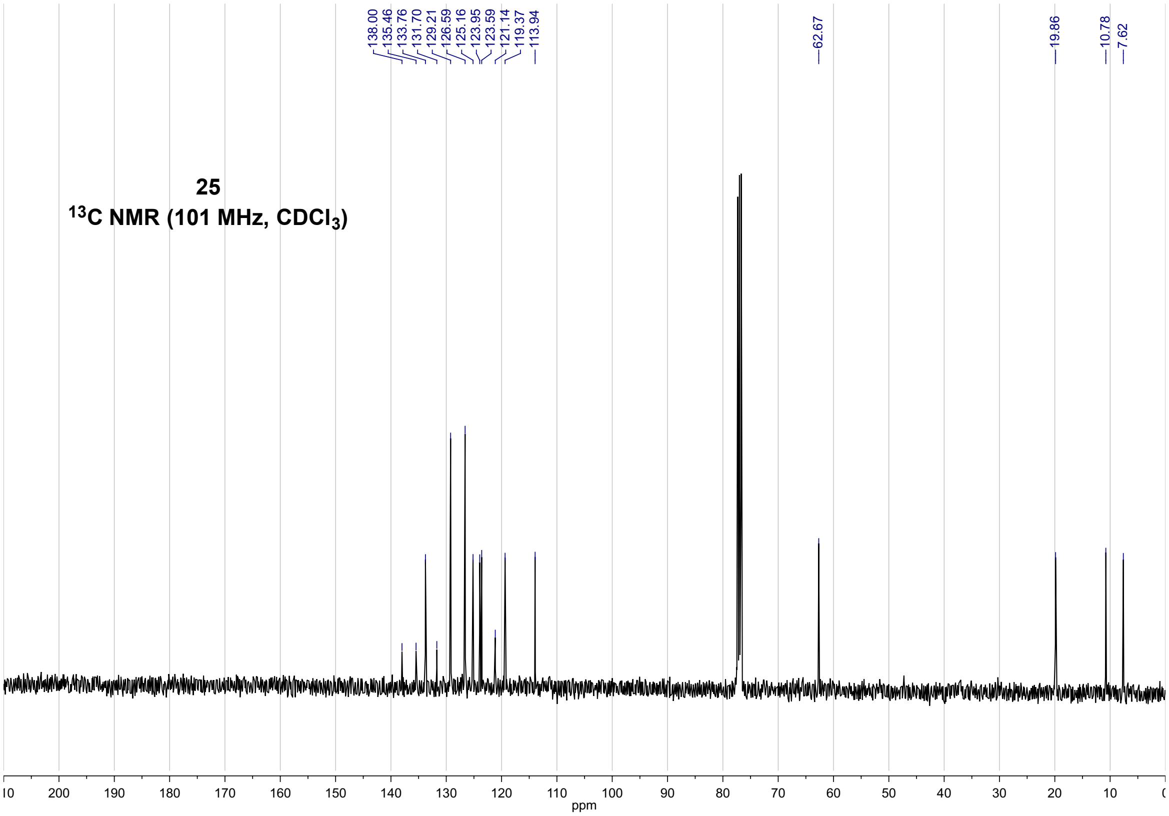

25

^{13}C NMR (101 MHz, $CDCl_3$)

X-ray crystallography data

X-ray crystallography data

Single crystals were grown by dissolving the compound in the minimum volume of ethyl acetate and allowing the solvent to slowly evaporate from a loosely capped vial. CCDC 1548245, CCDC 1548246, CCDC 1550178 and CCDC 1550151 contain the supplementary crystallographic data for compounds (+)-**4**, (–)-**4**, (–)-**5** and (+)-**5,** respectively, for this paper. The data can be obtained free of charge from the Cambridge Crystallographic Data Centre via www.ccdc.cam.ac.uk/structures.

Ethyl (1*R*,2*S*)-2-(benzenesulfonyl)cyclopropane-1-carboxylate (+)-4

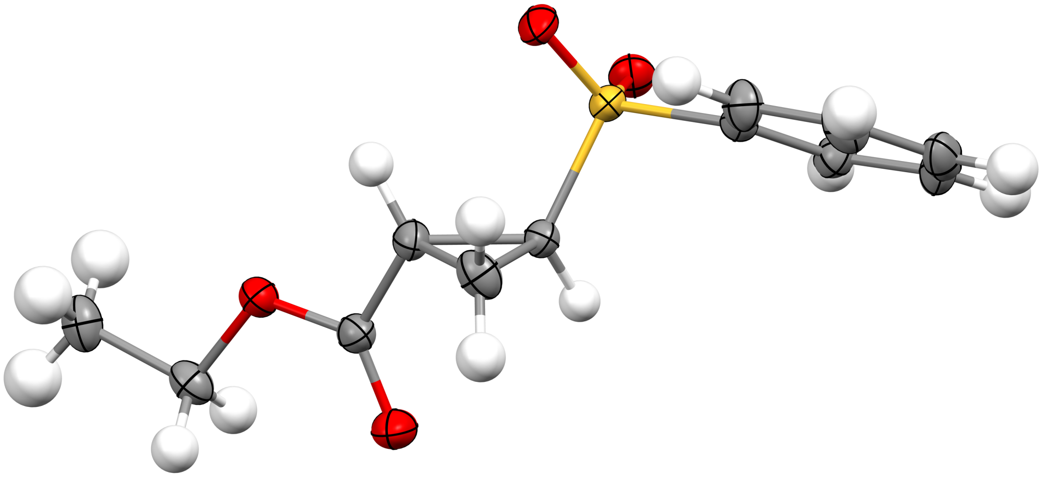

Table **S5**. Crystal data and structure refinement for ethyl (1*R*,2*S*)-2-(benzenesulfonyl)cyclopropane-1-carboxylate **(+)-4**.

Identification code	cu_16094_0m_a	
Empirical formula	C12 H14 O4 S	
Formula weight	254.29	
Temperature	100(2) K	
Wavelength	1.54178 Å	
Crystal system	Monoclinic	
Space group	$P2_1$	
Unit cell dimensions	a = 5.3506(4) Å	= 90°.
	b = 14.0370(10) Å	= 104.317(5)°.
	c = 8.5086(9) Å	= 90°.
Volume	619.20(9) $Å^3$	
Z	2	
Density (calculated)	1.364 Mg/m^3	
Absorption coefficient	2.349 mm^{-1}	
F(000)	268	
Crystal size	0.230 x 0.097 x 0.056 mm^3	
Theta range for data collection	5.365 to 72.241°.	

Index ranges	-6<=h<=6, -17<=k<=17, -10<=l<=10
Reflections collected	9885
Independent reflections	2386 [R(int) = 0.0322]
Completeness to theta = 67.679°	99.6 %
Refinement method	Full-matrix least-squares on F^2
Data / restraints / parameters	2386 / 1 / 156
Goodness-of-fit on F^2	1.049
Final R indices [I>2sigma(I)]	R1 = 0.0274, wR2 = 0.0691
R indices (all data)	R1 = 0.0277, wR2 = 0.0694
Absolute structure parameter	0.070(6)
Extinction coefficient	0.058(5)
Largest diff. peak and hole	0.248 and -0.341 e.Å^{-3}

Table **S6**. Atomic coordinates (x 10^4) and equivalent isotropic displacement parameters (Å^{2}x 10^3) for ethyl (1*R*,2*S*)-2-(benzenesulfonyl)cyclopropane-1-carboxylate **(+)-4**. U(eq) is defined as one third of the trace of the orthogonalized U^{ij} tensor.

	x	y	z	U(eq)
S(1)	5421(1)	4071(1)	4387(1)	20(1)
O(1)	3961(3)	3293(1)	3504(2)	29(1)
O(5)	2322(3)	6212(1)	-105(2)	26(1)
O(2)	7992(3)	4241(1)	4207(2)	30(1)
O(4)	146(3)	6490(1)	1798(2)	36(1)
C(1)	4250(4)	5721(2)	2517(3)	24(1)
C(8)	5591(4)	3918(1)	6479(2)	21(1)
C(2)	3627(4)	5121(1)	3863(2)	21(1)
C(4)	2000(4)	6181(1)	1396(3)	24(1)
C(13)	7643(4)	4315(2)	7611(3)	29(1)
C(9)	3697(4)	3398(2)	6940(3)	27(1)
C(3)	4954(5)	6063(2)	4248(3)	30(1)
C(6)	346(4)	6711(2)	-1309(3)	28(1)
C(10)	3866(5)	3278(2)	8592(3)	32(1)
C(12)	7756(5)	4209(2)	9255(3)	36(1)
C(11)	5862(5)	3692(2)	9733(3)	33(1)
C(7)	1274(5)	6724(2)	-2838(3)	33(1)

Table **S7**. Bond lengths [Å] and angles [°] for ethyl (1*R*,2*S*)-2-(benzenesulfonyl)cyclopropane-1-carboxylate **(+)-4**.

S(1)-O(1)	1.4406(16)
S(1)-O(2)	1.4414(14)
S(1)-C(2)	1.755(2)
S(1)-C(8)	1.7724(19)
O(5)-C(4)	1.331(3)
O(5)-C(6)	1.457(3)
O(4)-C(4)	1.207(3)
C(1)-C(4)	1.486(3)
C(1)-C(3)	1.506(3)
C(1)-C(2)	1.523(3)
C(1)-H(1)	1.0000
C(8)-C(9)	1.382(3)
C(8)-C(13)	1.386(3)
C(2)-C(3)	1.499(3)
C(2)-H(2)	1.0000
C(13)-C(12)	1.393(3)
C(13)-H(13)	0.9500
C(9)-C(10)	1.396(3)
C(9)-H(9)	0.9500
C(3)-H(3A)	0.9900
C(3)-H(3B)	0.9900
C(6)-C(7)	1.502(3)
C(6)-H(6A)	0.9900
C(6)-H(6B)	0.9900
C(10)-C(11)	1.381(4)
C(10)-H(10)	0.9500
C(12)-C(11)	1.387(4)
C(12)-H(12)	0.9500
C(11)-H(11)	0.9500
C(7)-H(7A)	0.9800
C(7)-H(7B)	0.9800
C(7)-H(7C)	0.9800
O(1)-S(1)-O(2)	118.60(10)
O(1)-S(1)-C(2)	108.16(9)

O(2)-S(1)-C(2)	108.07(10)
O(1)-S(1)-C(8)	108.59(9)
O(2)-S(1)-C(8)	108.26(10)
C(2)-S(1)-C(8)	104.23(9)
C(4)-O(5)-C(6)	116.57(16)
C(4)-C(1)-C(3)	117.11(18)
C(4)-C(1)-C(2)	115.46(17)
C(3)-C(1)-C(2)	59.31(13)
C(4)-C(1)-H(1)	117.4
C(3)-C(1)-H(1)	117.4
C(2)-C(1)-H(1)	117.4
C(9)-C(8)-C(13)	121.69(19)
C(9)-C(8)-S(1)	119.32(16)
C(13)-C(8)-S(1)	118.98(16)
C(3)-C(2)-C(1)	59.79(14)
C(3)-C(2)-S(1)	119.08(15)
C(1)-C(2)-S(1)	116.61(14)
C(3)-C(2)-H(2)	116.5
C(1)-C(2)-H(2)	116.5
S(1)-C(2)-H(2)	116.5
O(4)-C(4)-O(5)	124.75(19)
O(4)-C(4)-C(1)	124.5(2)
O(5)-C(4)-C(1)	110.72(16)
C(8)-C(13)-C(12)	119.0(2)
C(8)-C(13)-H(13)	120.5
C(12)-C(13)-H(13)	120.5
C(8)-C(9)-C(10)	118.7(2)
C(8)-C(9)-H(9)	120.6
C(10)-C(9)-H(9)	120.6
C(2)-C(3)-C(1)	60.90(14)
C(2)-C(3)-H(3A)	117.7
C(1)-C(3)-H(3A)	117.7
C(2)-C(3)-H(3B)	117.7
C(1)-C(3)-H(3B)	117.7
H(3A)-C(3)-H(3B)	114.8
O(5)-C(6)-C(7)	106.10(19)
O(5)-C(6)-H(6A)	110.5
C(7)-C(6)-H(6A)	110.5

O(5)-C(6)-H(6B)	110.5
C(7)-C(6)-H(6B)	110.5
H(6A)-C(6)-H(6B)	108.7
C(11)-C(10)-C(9)	120.2(2)
C(11)-C(10)-H(10)	119.9
C(9)-C(10)-H(10)	119.9
C(11)-C(12)-C(13)	119.8(2)
C(11)-C(12)-H(12)	120.1
C(13)-C(12)-H(12)	120.1
C(10)-C(11)-C(12)	120.6(2)
C(10)-C(11)-H(11)	119.7
C(12)-C(11)-H(11)	119.7
C(6)-C(7)-H(7A)	109.5
C(6)-C(7)-H(7B)	109.5
H(7A)-C(7)-H(7B)	109.5
C(6)-C(7)-H(7C)	109.5
H(7A)-C(7)-H(7C)	109.5
H(7B)-C(7)-H(7C)	109.5

Symmetry transformations used to generate equivalent atoms:

Table **S8**. Anisotropic displacement parameters (Å^{2}x 10^3) for ethyl (1*R*,2*S*)-2-(benzenesulfonyl) cyclopropane-1-carboxylate **(+)-4**. The anisotropic displacement factor exponent takes the form: $-2\ ^{2}[\ h^2\ a^{*2}U^{11} + \ldots + 2\ h\ k\ a^*\ b^*\ U^{12}\ ]$

	U^{11}	U^{22}	U^{33}	U^{23}	U^{13}	U^{12}
S(1)	20(1)	22(1)	21(1)	1(1)	9(1)	2(1)
O(1)	35(1)	24(1)	28(1)	-5(1)	9(1)	0(1)
O(5)	26(1)	28(1)	24(1)	4(1)	6(1)	5(1)
O(2)	21(1)	40(1)	32(1)	10(1)	12(1)	5(1)
O(4)	32(1)	43(1)	35(1)	9(1)	14(1)	14(1)
C(1)	23(1)	25(1)	25(1)	4(1)	8(1)	0(1)
C(8)	23(1)	18(1)	24(1)	1(1)	9(1)	2(1)
C(2)	20(1)	20(1)	22(1)	1(1)	7(1)	0(1)
C(4)	24(1)	22(1)	28(1)	2(1)	8(1)	1(1)
C(13)	31(1)	27(1)	30(1)	2(1)	7(1)	-8(1)
C(9)	26(1)	27(1)	30(1)	-1(1)	10(1)	-3(1)
C(3)	35(1)	22(1)	30(1)	-1(1)	2(1)	-4(1)
C(6)	26(1)	25(1)	28(1)	3(1)	1(1)	2(1)
C(10)	37(1)	30(1)	34(1)	6(1)	19(1)	-1(1)
C(12)	47(1)	31(1)	26(1)	-1(1)	1(1)	-8(1)
C(11)	50(1)	28(1)	22(1)	2(1)	14(1)	5(1)
C(7)	38(1)	32(1)	26(1)	4(1)	4(1)	-7(1)

Table **S9**. Hydrogen coordinates (x 10^4) and isotropic displacement parameters ($Å^2$x 10^3) for ethyl (1*R*,2*S*)-2-(benzenesulfonyl)cyclopropane-1-carboxylate **(+)-4**.

	x	y	z	U(eq)
H(1)	5686	5498	2047	29
H(2)	1774	5094	3898	25
H(13)	8953	4655	7271	35
H(9)	2308	3128	6149	32
H(3A)	6801	6059	4830	36
H(3B)	3944	6597	4537	36
H(6A)	122	7368	-945	33
H(6B)	-1326	6373	-1491	33
H(10)	2605	2910	8933	38
H(12)	9127	4490	10046	43
H(11)	5937	3623	10854	39
H(7A)	2949	7046	-2630	49
H(7B)	25	7066	-3687	49
H(7C)	1453	6069	-3193	49

Table **S10**. Torsion angles [°] for ethyl (1*R*,2*S*)-2-(benzenesulfonyl)cyclopropane-1-carboxylate **(+)-4**.

O(1)-S(1)-C(8)-C(9)	-24.98(19)
O(2)-S(1)-C(8)-C(9)	-154.98(17)
C(2)-S(1)-C(8)-C(9)	90.14(18)
O(1)-S(1)-C(8)-C(13)	154.26(17)
O(2)-S(1)-C(8)-C(13)	24.26(19)
C(2)-S(1)-C(8)-C(13)	-90.62(18)
C(4)-C(1)-C(2)-C(3)	-107.7(2)
C(4)-C(1)-C(2)-S(1)	142.57(17)
C(3)-C(1)-C(2)-S(1)	-109.71(18)
O(1)-S(1)-C(2)-C(3)	-163.86(15)
O(2)-S(1)-C(2)-C(3)	-34.30(18)
C(8)-S(1)-C(2)-C(3)	80.72(17)
O(1)-S(1)-C(2)-C(1)	-95.29(16)
O(2)-S(1)-C(2)-C(1)	34.27(18)
C(8)-S(1)-C(2)-C(1)	149.28(15)
C(6)-O(5)-C(4)-O(4)	4.0(3)
C(6)-O(5)-C(4)-C(1)	-175.52(19)
C(3)-C(1)-C(4)-O(4)	-31.5(3)
C(2)-C(1)-C(4)-O(4)	35.5(3)
C(3)-C(1)-C(4)-O(5)	148.05(18)
C(2)-C(1)-C(4)-O(5)	-144.98(18)
C(9)-C(8)-C(13)-C(12)	-1.8(3)
S(1)-C(8)-C(13)-C(12)	178.96(17)
C(13)-C(8)-C(9)-C(10)	0.3(3)
S(1)-C(8)-C(9)-C(10)	179.54(16)
S(1)-C(2)-C(3)-C(1)	105.62(17)
C(4)-C(1)-C(3)-C(2)	104.9(2)
C(4)-O(5)-C(6)-C(7)	174.78(17)
C(8)-C(9)-C(10)-C(11)	1.5(3)
C(8)-C(13)-C(12)-C(11)	1.5(4)
C(9)-C(10)-C(11)-C(12)	-1.8(4)
C(13)-C(12)-C(11)-C(10)	0.3(4)

Symmetry transformations used to generate equivalent atoms:

Ethyl (1*S*,2*R*)-2-(benzenesulfonyl)cyclopropane-1-carboxylate (–)-4

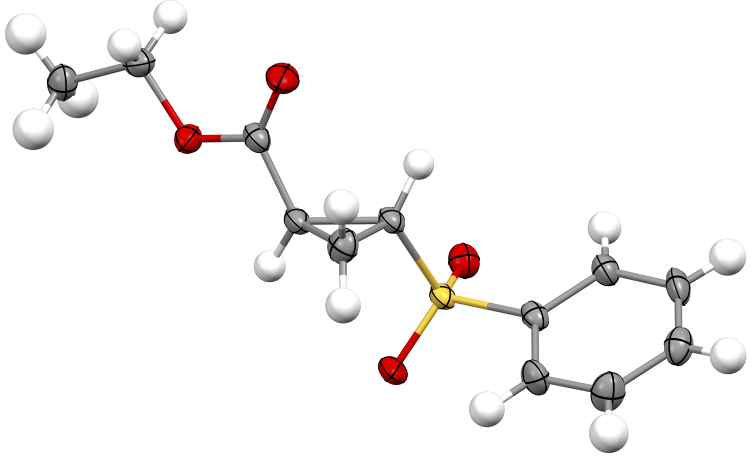

Table **S11**. Crystal data and structure refinement for lot ethyl (1*S*,2*R*)-2-(benzenesulfonyl)cyclopropane-1-carboxylate **(–)-4**.

Identification code	cu_16082_0m_a	
Empirical formula	C12 H14 O4 S	
Formula weight	254.29	
Temperature	100(2) K	
Wavelength	1.54178 Å	
Crystal system	Monoclinic	
Space group	$P2_1$	
Unit cell dimensions	a = 5.3421(7) Å	= 90°.
	b = 14.016(2) Å	= 104.318(13)°.
	c = 8.4966(15) Å	= 90°.
Volume	616.41(17) Å^3	
Z	2	
Density (calculated)	1.370 Mg/m^3	
Absorption coefficient	2.360 mm^{-1}	
F(000)	268	
Crystal size	0.349 x 0.319 x 0.117 mm^3	
Theta range for data collection	5.373 to 72.390°.	
Index ranges	-6<=h<=6, -17<=k<=17, -10<=l<=10	
Reflections collected	8416	
Independent reflections	2356 [R(int) = 0.0592]	
Completeness to theta = 67.679°	99.7 %	
Absorption correction	Multi-scan	
Refinement method	Full-matrix least-squares on F^2	
Data / restraints / parameters	2356 / 1 / 156	

Goodness-of-fit on F^2	1.037
Final R indices [I>2sigma(I)]	R1 = 0.0388, wR2 = 0.1006
R indices (all data)	R1 = 0.0406, wR2 = 0.1020
Absolute structure parameter	0.163(13)
Extinction coefficient	0.022(3)
Largest diff. peak and hole	0.433 and -0.396 e.Å^{-3}

Table **S12**. Atomic coordinates (x 10^4) and equivalent isotropic displacement parameters (Å^{2}x 10^3) for ethyl (1*S*,2*R*)-2-(benzenesulfonyl)cyclopropane-1-carboxylate **(–)-4**. U(eq) is defined as one third of the trace of the orthogonalized U^{ij} tensor.

	x	y	z	U(eq)
S(1)	5418(1)	5931(1)	4393(1)	21(1)
O(2)	3956(5)	6709(2)	3513(3)	29(1)
O(1)	7986(5)	5763(2)	4216(3)	30(1)
O(5)	2335(5)	3789(2)	-99(3)	25(1)
O(4)	159(6)	3512(2)	1801(4)	36(1)
C(2)	4260(7)	4281(2)	2526(4)	23(1)
C(4)	2007(6)	3817(2)	1406(4)	23(1)
C(8)	5590(7)	6083(2)	6477(4)	22(1)
C(1)	3634(6)	4881(3)	3871(4)	22(1)
C(6)	343(7)	3290(3)	-1308(5)	27(1)
C(9)	7637(7)	5681(3)	7617(5)	29(1)
C(13)	3692(7)	6603(3)	6948(4)	26(1)
C(3)	4961(8)	3938(3)	4252(5)	30(1)
C(10)	7743(8)	5789(3)	9256(5)	36(1)
C(11)	5855(8)	6302(3)	9742(5)	31(1)
C(7)	1286(8)	3274(3)	-2829(5)	31(1)
C(12)	3862(8)	6722(3)	8594(5)	32(1)

Table **S13**. Bond lengths [Å] and angles [°] for ethyl (1*S*,2*R*)-2-(benzenesulfonyl)cyclopropane-1-carboxylate **(–)-4**.

S(1)-O(1)	1.436(2)
S(1)-O(2)	1.438(3)
S(1)-C(1)	1.751(4)
S(1)-C(8)	1.763(4)
O(5)-C(4)	1.334(4)
O(5)-C(6)	1.462(4)
O(4)-C(4)	1.197(4)
C(2)-C(4)	1.487(5)
C(2)-C(3)	1.500(5)
C(2)-C(1)	1.521(5)
C(2)-H(2)	1.0000
C(8)-C(13)	1.386(5)
C(8)-C(9)	1.389(5)
C(1)-C(3)	1.497(5)
C(1)-H(1)	1.0000
C(6)-C(7)	1.498(5)
C(6)-H(6A)	0.9900
C(6)-H(6B)	0.9900
C(9)-C(10)	1.388(5)
C(9)-H(9)	0.9500
C(13)-C(12)	1.389(5)
C(13)-H(13)	0.9500
C(3)-H(3A)	0.9900
C(3)-H(3B)	0.9900
C(10)-C(11)	1.382(6)
C(10)-H(10)	0.9500
C(11)-C(12)	1.385(6)
C(11)-H(11)	0.9500
C(7)-H(7A)	0.9800
C(7)-H(7B)	0.9800
C(7)-H(7C)	0.9800
C(12)-H(12)	0.9500
O(1)-S(1)-O(2)	118.68(16)
O(1)-S(1)-C(1)	108.02(16)

O(2)-S(1)-C(1)	108.30(16)
O(1)-S(1)-C(8)	108.15(17)
O(2)-S(1)-C(8)	108.50(16)
C(1)-S(1)-C(8)	104.26(16)
C(4)-O(5)-C(6)	116.3(3)
C(4)-C(2)-C(3)	116.9(3)
C(4)-C(2)-C(1)	115.4(3)
C(3)-C(2)-C(1)	59.4(2)
C(4)-C(2)-H(2)	117.5
C(3)-C(2)-H(2)	117.5
C(1)-C(2)-H(2)	117.5
O(4)-C(4)-O(5)	124.8(3)
O(4)-C(4)-C(2)	124.8(3)
O(5)-C(4)-C(2)	110.4(3)
C(13)-C(8)-C(9)	121.2(3)
C(13)-C(8)-S(1)	119.6(3)
C(9)-C(8)-S(1)	119.2(3)
C(3)-C(1)-C(2)	59.6(2)
C(3)-C(1)-S(1)	119.2(3)
C(2)-C(1)-S(1)	116.6(2)
C(3)-C(1)-H(1)	116.4
C(2)-C(1)-H(1)	116.4
S(1)-C(1)-H(1)	116.4
O(5)-C(6)-C(7)	105.8(3)
O(5)-C(6)-H(6A)	110.6
C(7)-C(6)-H(6A)	110.6
O(5)-C(6)-H(6B)	110.6
C(7)-C(6)-H(6B)	110.6
H(6A)-C(6)-H(6B)	108.7
C(10)-C(9)-C(8)	119.0(3)
C(10)-C(9)-H(9)	120.5
C(8)-C(9)-H(9)	120.5
C(8)-C(13)-C(12)	118.9(3)
C(8)-C(13)-H(13)	120.5
C(12)-C(13)-H(13)	120.5
C(1)-C(3)-C(2)	61.0(2)
C(1)-C(3)-H(3A)	117.7
C(2)-C(3)-H(3A)	117.7

C(1)-C(3)-H(3B)	117.7
C(2)-C(3)-H(3B)	117.7
H(3A)-C(3)-H(3B)	114.8
C(11)-C(10)-C(9)	120.3(4)
C(11)-C(10)-H(10)	119.8
C(9)-C(10)-H(10)	119.8
C(10)-C(11)-C(12)	120.1(4)
C(10)-C(11)-H(11)	119.9
C(12)-C(11)-H(11)	119.9
C(6)-C(7)-H(7A)	109.5
C(6)-C(7)-H(7B)	109.5
H(7A)-C(7)-H(7B)	109.5
C(6)-C(7)-H(7C)	109.5
H(7A)-C(7)-H(7C)	109.5
H(7B)-C(7)-H(7C)	109.5
C(11)-C(12)-C(13)	120.4(3)
C(11)-C(12)-H(12)	119.8
C(13)-C(12)-H(12)	119.8

Symmetry transformations used to generate equivalent atoms:

Table **S14**. Anisotropic displacement parameters ($Å^2$x 10^3) for ethyl (1*S*,2*R*)-2-(benzenesulfonyl) cyclopropane-1-carboxylate **(–)-4**. The anisotropicdisplacement factor exponent takes the form: $-2\ ^2[\ h^2\ a^{*2}U^{11} + ...\ + 2\ h\ k\ a^*\ b^*\ U^{12}\ ]$

	U^{11}	U^{22}	U^{33}	U^{23}	U^{13}	U^{12}
S(1)	19(1)	23(1)	24(1)	-2(1)	12(1)	-2(1)
O(2)	34(1)	26(1)	29(1)	1(1)	13(1)	-1(1)
O(1)	23(1)	39(2)	34(1)	-9(1)	16(1)	-6(1)
O(5)	26(1)	28(1)	23(1)	-4(1)	10(1)	-7(1)
O(4)	30(2)	44(2)	38(2)	-9(1)	18(1)	-14(1)
C(2)	20(2)	24(2)	28(2)	-4(1)	12(1)	-2(1)
C(4)	22(2)	21(2)	29(2)	0(1)	12(1)	0(1)
C(8)	23(2)	19(2)	29(2)	0(1)	13(1)	-2(1)
C(1)	19(2)	24(2)	25(2)	-1(1)	11(1)	-1(1)
C(6)	22(2)	26(2)	32(2)	-3(2)	5(2)	-1(1)
C(9)	29(2)	27(2)	34(2)	-2(1)	11(2)	8(1)
C(13)	26(2)	26(2)	29(2)	-1(1)	13(2)	2(1)
C(3)	34(2)	22(2)	32(2)	-1(1)	7(2)	3(2)
C(10)	43(2)	35(2)	27(2)	2(2)	4(2)	7(2)
C(11)	42(2)	28(2)	26(2)	-2(1)	15(2)	-4(2)
C(7)	35(2)	30(2)	28(2)	-3(2)	8(2)	6(2)
C(12)	37(2)	30(2)	35(2)	-7(2)	24(2)	-1(2)

Table **S15**. Hydrogen coordinates (x 10^4) and isotropic displacement parameters ($Å^2$x 10^3) for ethyl (1*S*,2*R*)-2-(benzenesulfonyl)cyclopropane-1-carboxylate **(–)-4**.

	x	y	z	U(eq)
H(2)	5698	4504	2055	28
H(1)	1777	4906	3904	26
H(6A)	-1327	3631	-1494	32
H(6B)	110	2632	-944	32
H(9)	8946	5337	7280	35
H(13)	2296	6873	6158	31
H(3A)	3949	3402	4539	35
H(3B)	6811	3941	4836	35
H(10)	9121	5510	10048	43
H(11)	5924	6366	10865	37
H(7A)	1456	3930	-3190	47
H(7B)	47	2927	-3679	47
H(7C)	2970	2956	-2612	47
H(12)	2605	7093	8935	38

Table **S16**. Torsion angles [°] for ethyl (1*S*,2*R*)-2-(benzenesulfonyl)cyclopropane-1-carboxylate **(–)-4**.

C(6)-O(5)-C(4)-O(4)	-4.2(5)
C(6)-O(5)-C(4)-C(2)	175.8(3)
C(3)-C(2)-C(4)-O(4)	31.9(5)
C(1)-C(2)-C(4)-O(4)	-35.1(5)
C(3)-C(2)-C(4)-O(5)	-148.1(3)
C(1)-C(2)-C(4)-O(5)	144.9(3)
O(1)-S(1)-C(8)-C(13)	155.1(3)
O(2)-S(1)-C(8)-C(13)	25.1(3)
C(1)-S(1)-C(8)-C(13)	-90.1(3)
O(1)-S(1)-C(8)-C(9)	-24.7(3)
O(2)-S(1)-C(8)-C(9)	-154.7(3)
C(1)-S(1)-C(8)-C(9)	90.0(3)
C(4)-C(2)-C(1)-C(3)	107.5(3)
C(4)-C(2)-C(1)-S(1)	-142.6(3)
C(3)-C(2)-C(1)-S(1)	109.9(3)
O(1)-S(1)-C(1)-C(3)	34.1(3)
O(2)-S(1)-C(1)-C(3)	163.8(3)
C(8)-S(1)-C(1)-C(3)	-80.8(3)
O(1)-S(1)-C(1)-C(2)	-34.3(3)
O(2)-S(1)-C(1)-C(2)	95.4(3)
C(8)-S(1)-C(1)-C(2)	-149.2(3)
C(4)-O(5)-C(6)-C(7)	-174.5(3)
C(13)-C(8)-C(9)-C(10)	1.2(5)
S(1)-C(8)-C(9)-C(10)	-178.9(3)
C(9)-C(8)-C(13)-C(12)	0.2(5)
S(1)-C(8)-C(13)-C(12)	-179.6(3)
S(1)-C(1)-C(3)-C(2)	-105.5(3)
C(4)-C(2)-C(3)-C(1)	-105.0(3)
C(8)-C(9)-C(10)-C(11)	-0.9(6)
C(9)-C(10)-C(11)-C(12)	-0.9(7)
C(10)-C(11)-C(12)-C(13)	2.4(7)
C(8)-C(13)-C(12)-C(11)	-2.0(6)

Symmetry transformations used to generate equivalent atoms:

Ethyl (1*S*,2*S*)-2-(benzenesulfonyl)cyclopropane-1-carboxylate (–)-5

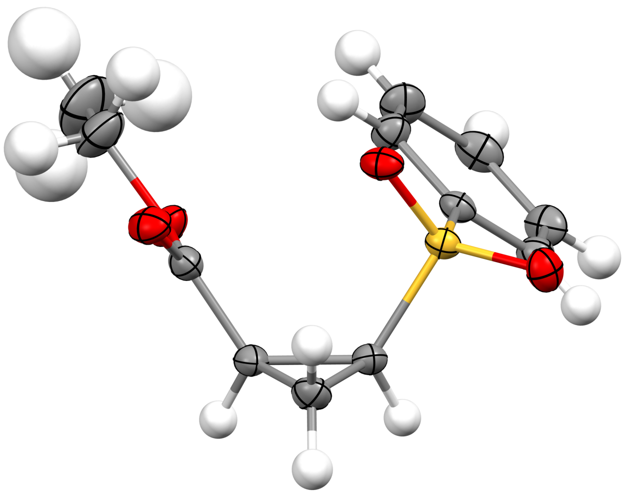

Table **S17**. Crystal data and structure refinement for lot ethyl (1*S*,2*S*)-2-(benzenesulfonyl)cyclopropane-1-carboxylate **(–)-5**.

Identification code	cu_16003_0m_a	
Empirical formula	C12 H14 O4 S	
Formula weight	254.29	
Temperature	100(2) K	
Wavelength	1.54178 Å	
Crystal system	Orthorhombic	
Space group	$P2_12_12_1$	
Unit cell dimensions	a = 5.516(2) Å	= 90°.
	b = 14.424(7) Å	= 90°.
	c = 15.436(9) Å	= 90°.
Volume	1228.1(10) $Å^3$	
Z	4	
Density (calculated)	1.375 Mg/m^3	
Absorption coefficient	2.369 mm^{-1}	
F(000)	536	
Crystal size	0.250 x 0.070 x 0.050 mm^3	
Theta range for data collection	6.505 to 72.585°.	
Index ranges	-6<=h<=6, -15<=k<=17, -18<=l<=18	
Reflections collected	12802	
Independent reflections	2389 [R(int) = 0.0281]	
Completeness to theta = 67.679°	99.2 %	
Absorption correction	Multi-scan	
Refinement method	Full-matrix least-squares on F^2	

Data / restraints / parameters	2389 / 0 / 156
Goodness-of-fit on F^2	1.066
Final R indices [I>2sigma(I)]	R1 = 0.0272, wR2 = 0.0763
R indices (all data)	R1 = 0.0276, wR2 = 0.0766
Absolute structure parameter	0.047(4)
Extinction coefficient	0.0119(16)
Largest diff. peak and hole	0.682 and -0.281 e.Å^{-3}

Table **S18**. Atomic coordinates (x 10^4) and equivalent isotropic displacement parameters (Å^{2}x 10^3) for ethyl (1*S*,2*S*)-2-(benzenesulfonyl)cyclopropane-1-carboxylate **(–)-5**. U(eq) is defined as one third of the trace of the orthogonalized U^{ij} tensor.

	x	y	z	U(eq)
S(1)	3737(1)	5244(1)	1731(1)	18(1)
O(2)	1167(3)	5418(1)	1670(1)	24(1)
O(1)	4754(3)	4484(1)	1254(1)	27(1)
O(4)	-60(3)	7483(1)	1424(1)	29(1)
O(5)	1839(3)	7479(1)	2709(1)	28(1)
C(8)	4501(4)	5102(2)	2833(1)	19(1)
C(2)	4225(4)	7203(2)	1495(2)	21(1)
C(3)	4455(5)	6757(2)	625(2)	24(1)
C(13)	2914(4)	5413(2)	3465(2)	25(1)
C(1)	5296(4)	6245(2)	1418(2)	20(1)
C(4)	1751(4)	7386(1)	1847(2)	22(1)
C(10)	7278(4)	4581(2)	3913(2)	28(1)
C(11)	5716(5)	4895(2)	4553(2)	30(1)
C(9)	6683(4)	4684(2)	3044(2)	24(1)
C(12)	3533(5)	5304(2)	4331(2)	30(1)
C(6)	-460(5)	7704(2)	3124(2)	43(1)
C(7)	37(8)	7809(3)	4059(2)	61(1)

Table **S19**. Bond lengths [Å] and angles [°] for ethyl (1*S*,2*S*)-2-(benzenesulfonyl)cyclopropane-1-carboxylate **(–)-5**.

S(1)-O(1)	1.4345(17)
S(1)-O(2)	1.4427(17)
S(1)-C(1)	1.749(2)
S(1)-C(8)	1.764(2)
O(4)-C(4)	1.201(3)
O(5)-C(4)	1.339(3)
O(5)-C(6)	1.457(3)
C(8)-C(9)	1.385(3)
C(8)-C(13)	1.386(3)
C(2)-C(4)	1.492(3)
C(2)-C(3)	1.494(3)
C(2)-C(1)	1.507(3)
C(2)-H(2)	1.0000
C(3)-C(1)	1.503(3)
C(3)-H(3A)	0.9900
C(3)-H(3B)	0.9900
C(13)-C(12)	1.388(3)
C(13)-H(13)	0.9500
C(1)-H(1)	1.0000
C(10)-C(11)	1.387(4)
C(10)-C(9)	1.389(3)
C(10)-H(10)	0.9500
C(11)-C(12)	1.383(4)
C(11)-H(11)	0.9500
C(9)-H(9)	0.9500
C(12)-H(12)	0.9500
C(6)-C(7)	1.477(5)
C(6)-H(6A)	0.9900
C(6)-H(6B)	0.9900
C(7)-H(7A)	0.9800
C(7)-H(7B)	0.9800
C(7)-H(7C)	0.9800
O(1)-S(1)-O(2)	118.92(10)
O(1)-S(1)-C(1)	107.29(10)

O(2)-S(1)-C(1)	108.76(10)
O(1)-S(1)-C(8)	108.22(11)
O(2)-S(1)-C(8)	108.51(10)
C(1)-S(1)-C(8)	104.16(11)
C(4)-O(5)-C(6)	115.3(2)
C(9)-C(8)-C(13)	121.6(2)
C(9)-C(8)-S(1)	119.04(17)
C(13)-C(8)-S(1)	119.40(19)
C(4)-C(2)-C(3)	118.7(2)
C(4)-C(2)-C(1)	123.30(18)
C(3)-C(2)-C(1)	60.11(15)
C(4)-C(2)-H(2)	114.6
C(3)-C(2)-H(2)	114.6
C(1)-C(2)-H(2)	114.6
C(2)-C(3)-C(1)	60.39(16)
C(2)-C(3)-H(3A)	117.7
C(1)-C(3)-H(3A)	117.7
C(2)-C(3)-H(3B)	117.7
C(1)-C(3)-H(3B)	117.7
H(3A)-C(3)-H(3B)	114.9
C(8)-C(13)-C(12)	119.1(2)
C(8)-C(13)-H(13)	120.4
C(12)-C(13)-H(13)	120.4
C(3)-C(1)-C(2)	59.50(15)
C(3)-C(1)-S(1)	118.59(17)
C(2)-C(1)-S(1)	122.88(16)
C(3)-C(1)-H(1)	114.9
C(2)-C(1)-H(1)	114.9
S(1)-C(1)-H(1)	114.9
O(4)-C(4)-O(5)	123.9(2)
O(4)-C(4)-C(2)	125.7(2)
O(5)-C(4)-C(2)	110.27(18)
C(11)-C(10)-C(9)	120.4(2)
C(11)-C(10)-H(10)	119.8
C(9)-C(10)-H(10)	119.8
C(12)-C(11)-C(10)	120.3(2)
C(12)-C(11)-H(11)	119.9
C(10)-C(11)-H(11)	119.9

C(8)-C(9)-C(10)	118.7(2)
C(8)-C(9)-H(9)	120.7
C(10)-C(9)-H(9)	120.7
C(11)-C(12)-C(13)	120.0(2)
C(11)-C(12)-H(12)	120.0
C(13)-C(12)-H(12)	120.0
O(5)-C(6)-C(7)	106.9(3)
O(5)-C(6)-H(6A)	110.3
C(7)-C(6)-H(6A)	110.3
O(5)-C(6)-H(6B)	110.3
C(7)-C(6)-H(6B)	110.3
H(6A)-C(6)-H(6B)	108.6
C(6)-C(7)-H(7A)	109.5
C(6)-C(7)-H(7B)	109.5
H(7A)-C(7)-H(7B)	109.5
C(6)-C(7)-H(7C)	109.5
H(7A)-C(7)-H(7C)	109.5
H(7B)-C(7)-H(7C)	109.5

Symmetry transformations used to generate equivalent atoms:

Table **S20**. Anisotropic displacement parameters ($Å^2$x 10^3) for ethyl (1*S*,2*S*)-2-(benzenesulfonyl) cyclopropane-1-carboxylate **(–)-5**. The anisotropic displacement factor exponent takes the form: $-2\ ^2[h^2 a^{*2}U^{11} + ... + 2 h k a^* b^* U^{12}]$

	U^{11}	U^{22}	U^{33}	U^{23}	U^{13}	U^{12}
S(1)	19(1)	20(1)	15(1)	2(1)	-1(1)	-2(1)
O(2)	19(1)	30(1)	22(1)	5(1)	-3(1)	-4(1)
O(1)	35(1)	24(1)	22(1)	-3(1)	-1(1)	1(1)
O(4)	20(1)	34(1)	32(1)	6(1)	-2(1)	1(1)
O(5)	26(1)	35(1)	23(1)	0(1)	4(1)	7(1)
C(8)	21(1)	19(1)	17(1)	3(1)	-1(1)	-3(1)
C(2)	18(1)	20(1)	26(1)	2(1)	2(1)	-2(1)
C(3)	26(1)	26(1)	21(1)	7(1)	4(1)	0(1)
C(13)	25(1)	28(1)	21(1)	2(1)	2(1)	3(1)
C(1)	18(1)	21(1)	20(1)	1(1)	3(1)	-1(1)
C(4)	22(1)	20(1)	25(1)	4(1)	1(1)	-1(1)
C(10)	25(1)	32(1)	26(1)	7(1)	-7(1)	1(1)
C(11)	37(1)	33(1)	20(1)	5(1)	-4(1)	-6(1)
C(9)	23(1)	26(1)	22(1)	4(1)	1(1)	1(1)
C(12)	38(1)	36(1)	18(1)	1(1)	5(1)	2(1)
C(6)	36(1)	56(2)	36(2)	2(1)	12(1)	15(1)
C(7)	70(2)	70(2)	41(2)	-12(2)	17(2)	18(2)

Table **S21**. Hydrogen coordinates (x 10^4) and isotropic displacement parameters ($Å^2$ x 10^3) for ethyl (1*S*,2*S*)-2-(benzenesulfonyl)cyclopropane-1-carboxylate **(–)-5**.

	x	y	z	U(eq)
H(2)	5433	7705	1611	25
H(3A)	5708	7001	226	29
H(3B)	2952	6540	340	29
H(13)	1424	5697	3309	30
H(1)	7095	6215	1492	24
H(10)	8765	4294	4070	33
H(11)	6146	4830	5146	36
H(9)	7752	4473	2604	28
H(12)	2457	5509	4772	36
H(6A)	-1652	7202	3025	51
H(6B)	-1125	8288	2884	51
H(7A)	759	7236	4282	91
H(7B)	-1483	7934	4366	91
H(7C)	1163	8325	4149	91

Table **S22**. Torsion angles [°] for ethyl (1*S*,2*S*)-2-(benzenesulfonyl)cyclopropane-1-carboxylate **(–)-5**.

O(1)-S(1)-C(8)-C(9)	32.2(2)
O(2)-S(1)-C(8)-C(9)	162.56(17)
C(1)-S(1)-C(8)-C(9)	-81.71(19)
O(1)-S(1)-C(8)-C(13)	-148.10(18)
O(2)-S(1)-C(8)-C(13)	-17.8(2)
C(1)-S(1)-C(8)-C(13)	98.0(2)
C(4)-C(2)-C(3)-C(1)	-114.0(2)
C(9)-C(8)-C(13)-C(12)	0.0(4)
S(1)-C(8)-C(13)-C(12)	-179.62(19)
C(2)-C(3)-C(1)-S(1)	113.4(2)
C(4)-C(2)-C(1)-C(3)	106.6(2)
C(4)-C(2)-C(1)-S(1)	0.3(3)
C(3)-C(2)-C(1)-S(1)	-106.3(2)
O(1)-S(1)-C(1)-C(3)	85.59(19)
O(2)-S(1)-C(1)-C(3)	-44.2(2)
C(8)-S(1)-C(1)-C(3)	-159.81(18)
O(1)-S(1)-C(1)-C(2)	155.96(18)
O(2)-S(1)-C(1)-C(2)	26.1(2)
C(8)-S(1)-C(1)-C(2)	-89.4(2)
C(6)-O(5)-C(4)-O(4)	0.5(3)
C(6)-O(5)-C(4)-C(2)	177.2(2)
C(3)-C(2)-C(4)-O(4)	-24.5(3)
C(1)-C(2)-C(4)-O(4)	-95.9(3)
C(3)-C(2)-C(4)-O(5)	158.81(19)
C(1)-C(2)-C(4)-O(5)	87.5(2)
C(9)-C(10)-C(11)-C(12)	0.9(4)
C(13)-C(8)-C(9)-C(10)	-0.1(3)
S(1)-C(8)-C(9)-C(10)	179.52(17)
C(11)-C(10)-C(9)-C(8)	-0.3(3)
C(10)-C(11)-C(12)-C(13)	-1.0(4)
C(8)-C(13)-C(12)-C(11)	0.5(4)
C(4)-O(5)-C(6)-C(7)	-178.0(3)

Symmetry transformations used to generate equivalent atoms:

Ethyl (1*R*,2*R*)-2-(benzenesulfonyl)cyclopropane-1-carboxylate (+)-5

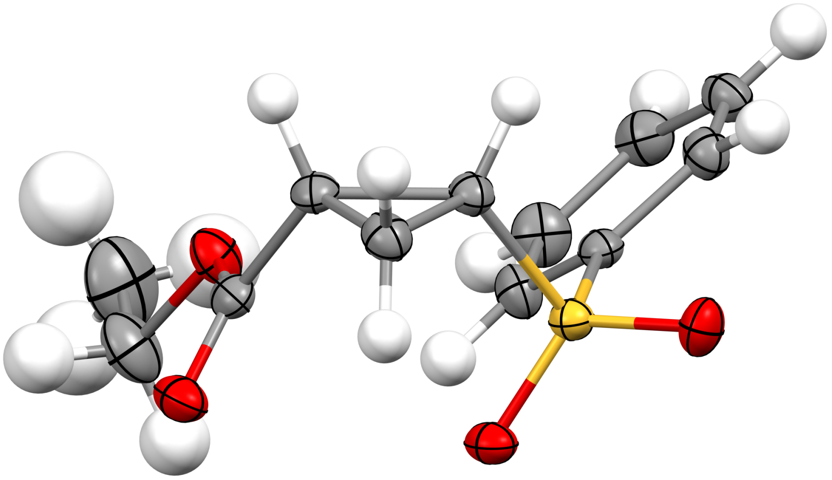

Table **S23**. Crystal data and structure refinement for lot ethyl (1*R*,2*R*)-2-(benzenesulfonyl)cyclopropane-1-carboxylate **(+)-5**.

Identification code	cu_16002_a	
Empirical formula	C12 H14 O4 S	
Formula weight	254.29	
Temperature	100(2) K	
Wavelength	1.54178 Å	
Crystal system	Orthorhombic	
Space group	$P2_12_12_1$	
Unit cell dimensions	a = 5.5189(16) Å	= 90°.
	b = 14.454(6) Å	= 90°.
	c = 15.447(5) Å	= 90°.
Volume	1232.3(7) $Å^3$	
Z	4	
Density (calculated)	1.371 Mg/m^3	
Absorption coefficient	2.361 mm^{-1}	
F(000)	536	
Crystal size	0.180 x 0.035 x 0.015 mm^3	
Theta range for data collection	4.189 to 72.547°.	
Index ranges	-6<=h<=6, -17<=k<=17, -19<=l<=17	
Reflections collected	11656	
Independent reflections	2425 [R(int) = 0.0832]	
Completeness to theta = 67.679°	99.5 %	
Absorption correction	Multi-scan	
Refinement method	Full-matrix least-squares on F^2	
Data / restraints / parameters	2425 / 0 / 156	

Goodness-of-fit on F^2	1.035
Final R indices [I>2sigma(I)]	R1 = 0.0463, wR2 = 0.1147
R indices (all data)	R1 = 0.0525, wR2 = 0.1203
Absolute structure parameter	0.067(17)
Extinction coefficient	0.0067(14)
Largest diff. peak and hole	0.528 and -0.526 e.Å^{-3}

Table **S24**. Atomic coordinates (x 10^4) and equivalent isotropic displacement parameters (Å^{2}x 10^3) for ethyl (1*R*,2*R*)-2-(benzenesulfonyl)cyclopropane-1-carboxylate **(+)-5**. U(eq) is defined as one third of the trace of the orthogonalized U^{ij} tensor.

	x	y	z	U(eq)
S(1)	3736(2)	4758(1)	1729(1)	19(1)
O(2)	1178(5)	4587(2)	1669(2)	24(1)
O(4)	-62(5)	2514(2)	1426(2)	28(1)
O(1)	4751(6)	5517(2)	1254(2)	27(1)
O(5)	1839(5)	2521(2)	2708(2)	29(1)
C(2)	4221(7)	2796(3)	1495(2)	21(1)
C(4)	1759(7)	2613(3)	1841(3)	22(1)
C(8)	4509(7)	4897(3)	2836(3)	20(1)
C(1)	5300(7)	3756(3)	1417(3)	20(1)
C(3)	4444(8)	3240(3)	625(3)	24(1)
C(13)	2906(8)	4590(3)	3463(3)	26(1)
C(10)	7285(7)	5416(3)	3914(3)	27(1)
C(9)	6687(7)	5318(3)	3042(3)	25(1)
C(11)	5713(8)	5103(3)	4552(3)	30(1)
C(12)	3532(9)	4694(3)	4326(3)	31(1)
C(6)	-460(9)	2301(4)	3116(3)	41(1)
C(7)	27(12)	2190(5)	4060(4)	60(2)

Table **S25**. Bond lengths [Å] and angles [°] for ethyl (1*R*,2*R*)-2-(benzenesulfonyl)cyclopropane-1-carboxylate **(+)-5**.

S(1)-O(1)	1.435(3)
S(1)-O(2)	1.436(3)
S(1)-C(1)	1.753(4)
S(1)-C(8)	1.773(4)
O(4)-C(4)	1.201(5)
O(5)-C(4)	1.345(5)
O(5)-C(6)	1.453(5)
C(2)-C(4)	1.484(5)
C(2)-C(3)	1.495(6)
C(2)-C(1)	1.516(5)
C(2)-H(2)	1.0000
C(8)-C(13)	1.385(6)
C(8)-C(9)	1.385(5)
C(1)-C(3)	1.509(6)
C(1)-H(1)	1.0000
C(3)-H(3A)	0.9900
C(3)-H(3B)	0.9900
C(13)-C(12)	1.385(6)
C(13)-H(13)	0.9500
C(10)-C(11)	1.389(6)
C(10)-C(9)	1.393(6)
C(10)-H(10)	0.9500
C(9)-H(9)	0.9500
C(11)-C(12)	1.386(6)
C(11)-H(11)	0.9500
C(12)-H(12)	0.9500
C(6)-C(7)	1.492(8)
C(6)-H(6A)	0.9900
C(6)-H(6B)	0.9900
C(7)-H(7A)	0.9800
C(7)-H(7B)	0.9800
C(7)-H(7C)	0.9800
O(1)-S(1)-O(2)	118.85(17)
O(1)-S(1)-C(1)	107.36(18)

O(2)-S(1)-C(1)	108.93(18)
O(1)-S(1)-C(8)	108.21(18)
O(2)-S(1)-C(8)	108.58(17)
C(1)-S(1)-C(8)	103.91(18)
C(4)-O(5)-C(6)	115.1(3)
C(4)-C(2)-C(3)	118.4(3)
C(4)-C(2)-C(1)	123.4(3)
C(3)-C(2)-C(1)	60.2(3)
C(4)-C(2)-H(2)	114.6
C(3)-C(2)-H(2)	114.6
C(1)-C(2)-H(2)	114.6
O(4)-C(4)-O(5)	123.2(4)
O(4)-C(4)-C(2)	126.5(4)
O(5)-C(4)-C(2)	110.2(3)
C(13)-C(8)-C(9)	122.3(4)
C(13)-C(8)-S(1)	119.0(3)
C(9)-C(8)-S(1)	118.7(3)
C(3)-C(1)-C(2)	59.2(3)
C(3)-C(1)-S(1)	118.5(3)
C(2)-C(1)-S(1)	122.8(3)
C(3)-C(1)-H(1)	115.0
C(2)-C(1)-H(1)	115.0
S(1)-C(1)-H(1)	115.0
C(2)-C(3)-C(1)	60.6(3)
C(2)-C(3)-H(3A)	117.7
C(1)-C(3)-H(3A)	117.7
C(2)-C(3)-H(3B)	117.7
C(1)-C(3)-H(3B)	117.7
H(3A)-C(3)-H(3B)	114.8
C(12)-C(13)-C(8)	118.6(4)
C(12)-C(13)-H(13)	120.7
C(8)-C(13)-H(13)	120.7
C(11)-C(10)-C(9)	120.3(4)
C(11)-C(10)-H(10)	119.8
C(9)-C(10)-H(10)	119.8
C(8)-C(9)-C(10)	118.2(4)
C(8)-C(9)-H(9)	120.9
C(10)-C(9)-H(9)	120.9

C(12)-C(11)-C(10)	120.1(4)
C(12)-C(11)-H(11)	119.9
C(10)-C(11)-H(11)	119.9
C(13)-C(12)-C(11)	120.4(4)
C(13)-C(12)-H(12)	119.8
C(11)-C(12)-H(12)	119.8
O(5)-C(6)-C(7)	106.9(4)
O(5)-C(6)-H(6A)	110.3
C(7)-C(6)-H(6A)	110.3
O(5)-C(6)-H(6B)	110.3
C(7)-C(6)-H(6B)	110.3
H(6A)-C(6)-H(6B)	108.6
C(6)-C(7)-H(7A)	109.5
C(6)-C(7)-H(7B)	109.5
H(7A)-C(7)-H(7B)	109.5
C(6)-C(7)-H(7C)	109.5
H(7A)-C(7)-H(7C)	109.5
H(7B)-C(7)-H(7C)	109.5

Symmetry transformations used to generate equivalent atoms:

Table **S26**. Anisotropic displacement parameters (Å^{2}x 10^3) for ethyl (1*R*,2*R*)-2-(benzenesulfonyl) cyclopropane-1-carboxylate **(+)-5**. The anisotropicdisplacement factor exponent takes the form: -2 ^{2}[h^2 a*^{2}U^{11} + ... + 2 h k a* b* U^{12}]

	U^{11}	U^{22}	U^{33}	U^{23}	U^{13}	U^{12}
S(1)	18(1)	19(1)	19(1)	-1(1)	0(1)	2(1)
O(2)	18(1)	26(1)	26(1)	-4(1)	-2(1)	4(1)
O(4)	18(1)	32(2)	34(2)	-6(1)	-1(1)	0(1)
O(1)	35(2)	21(1)	25(2)	4(1)	1(1)	-2(1)
O(5)	26(2)	35(2)	26(1)	2(1)	4(1)	-7(1)
C(2)	18(2)	20(2)	26(2)	-1(1)	3(1)	4(2)
C(4)	21(2)	18(2)	27(2)	-4(2)	1(2)	2(1)
C(8)	17(2)	19(2)	24(2)	-2(1)	-1(1)	3(1)
C(1)	17(2)	21(2)	23(2)	-2(2)	1(2)	0(2)
C(3)	22(2)	26(2)	25(2)	-4(2)	3(2)	-1(2)
C(13)	26(2)	25(2)	27(2)	-3(2)	1(2)	-5(2)
C(10)	22(2)	28(2)	31(2)	-7(2)	-4(2)	-1(2)
C(9)	25(2)	23(2)	26(2)	-4(2)	1(2)	-2(2)
C(11)	34(2)	32(2)	25(2)	-7(2)	-4(2)	4(2)
C(12)	38(2)	36(2)	20(2)	-2(2)	5(2)	-2(2)
C(6)	35(2)	52(3)	37(3)	1(2)	9(2)	-14(2)
C(7)	68(4)	71(4)	40(3)	11(3)	16(3)	-19(4)

Table **S27**. Hydrogen coordinates (x 10^4) and isotropic displacement parameters ($Å^2$x 10^3) for ethyl (1*R*,2*R*)-2-(benzenesulfonyl)cyclopropane-1-carboxylate **(+)-5**.

	x	y	z	U(eq)
H(2)	5427	2294	1610	26
H(1)	7099	3787	1488	24
H(3A)	2939	3457	341	29
H(3B)	5691	2996	225	29
H(13)	1408	4314	3305	31
H(10)	8775	5698	4072	32
H(9)	7746	5534	2602	30
H(11)	6133	5170	5145	36
H(12)	2461	4483	4765	37
H(6A)	-1131	1721	2873	50
H(6B)	-1643	2805	3018	50
H(7A)	1004	1633	4155	90
H(7B)	-1512	2132	4372	90
H(7C)	909	2732	4274	90

Table **S28**. Torsion angles [°] for ethyl (1*R*,2*R*)-2-(benzenesulfonyl)cyclopropane-1-carboxylate **(+)-5**.

C(6)-O(5)-C(4)-O(4)	-0.6(6)
C(6)-O(5)-C(4)-C(2)	-177.5(4)
C(3)-C(2)-C(4)-O(4)	24.5(6)
C(1)-C(2)-C(4)-O(4)	95.8(5)
C(3)-C(2)-C(4)-O(5)	-158.8(3)
C(1)-C(2)-C(4)-O(5)	-87.5(4)
O(1)-S(1)-C(8)-C(13)	147.6(3)
O(2)-S(1)-C(8)-C(13)	17.3(4)
C(1)-S(1)-C(8)-C(13)	-98.5(4)
O(1)-S(1)-C(8)-C(9)	-31.8(4)
O(2)-S(1)-C(8)-C(9)	-162.1(3)
C(1)-S(1)-C(8)-C(9)	82.1(3)
C(4)-C(2)-C(1)-C(3)	-106.2(4)
C(4)-C(2)-C(1)-S(1)	-0.1(5)
C(3)-C(2)-C(1)-S(1)	106.1(4)
O(1)-S(1)-C(1)-C(3)	-86.1(3)
O(2)-S(1)-C(1)-C(3)	43.8(4)
C(8)-S(1)-C(1)-C(3)	159.4(3)
O(1)-S(1)-C(1)-C(2)	-156.1(3)
O(2)-S(1)-C(1)-C(2)	-26.2(4)
C(8)-S(1)-C(1)-C(2)	89.4(3)
C(4)-C(2)-C(3)-C(1)	114.3(4)
S(1)-C(1)-C(3)-C(2)	-113.2(3)
C(9)-C(8)-C(13)-C(12)	-1.2(6)
S(1)-C(8)-C(13)-C(12)	179.4(3)
C(13)-C(8)-C(9)-C(10)	1.2(6)
S(1)-C(8)-C(9)-C(10)	-179.4(3)
C(11)-C(10)-C(9)-C(8)	-0.5(6)
C(9)-C(10)-C(11)-C(12)	-0.2(7)
C(8)-C(13)-C(12)-C(11)	0.5(7)
C(10)-C(11)-C(12)-C(13)	0.2(7)
C(4)-O(5)-C(6)-C(7)	177.7(4)

Symmetry transformations used to generate equivalent atoms:

References

[1] For observations on peroxo-bridged dimeric structures characterised by X-ray crystallography, see: R. G. Wilkins in *Bioinorganic Chemistry*, *Volume 100*, *Chapter 6,* (Eds.: R. Dessy, J. Dillard, L. Taylor), American Chemical Society, **1971**, pp. 111–134. EPR spectroscopy has also been used to observe the oxidation of paramagnetic Co^{II}-(salen)-type complexes to their diamagnetic Co^{III}-peroxo-bridged dimeric species: E. Vinck, E. Carter, D. M. Murphy, S. Van Doorslaer, *Inorg. Chem*. **2012**, *51*, 8014–8024.

[2] M. Quaranta, M. Murkovic, I. Klimant, *Analyst* **2013**, *138*, 6243–6245.

[3] M. Congreve, R. Carr, C. Murray, H. Jhoti, *Drug Discov. Today* **2003**, *8*, 876–877.

[4] H. Köster, T. Craan, S. Brass, C. Herhaus, M. Zentgraf, L. Neumann, A. Heine, G. Klebe, *J. Med. Chem.* **2011**, *54*, 7784–7796.

[5] A. Nadin, C. Hattotuwagama, I. Churcher, *Angew. Chem. Int. Ed*. **2012**, *51*, 1114–1122.

[6] J. D. Hughes, J. Blagg, D. A. Price, S. Bailey, G. A. DeCrescenzo, R. V. Devraj, E. Ellsworth, Y. M. Fobian, M. E. Gibbs, R. W. Gilles, N. Greene, E. Huang, T. Krieger-Burke, J. Loesel, T. Wager, L. Whiteley, Y. Zhang, *Bioorg. Med. Chem. Lett*. **2008**, *18*, 4872–4875.

[7] a) F. Lovering, J. Bikker, C. Humblet, *J. Med. Chem.* **2009**, *52*, 6752–6756; b) F. Lovering, *Med. Chem. Commun.* **2013**, *4*, 515-519.

[8] I. Colomer, C. J. Empson, P. Craven, Z. Owen, R. G. Doveston, I. Churcher, S. P. Marsden, A. Nelson *Chem. Commun.* **2016**, *52*, 7209–7212.

[9] For characteristic cyclopropane coupling constants, see: H. M. Hutton, T. Schaefer, *Can. J. Chem*. **1963**, *41,* 684–689.

[10] P. Kumar, A. Dubey, A. Harbindu, *Org. Biomol. Chem*. **2012**, *10*, 6987–6994.

[11] M. P. Doyle, R. L. Dorow, W. H. Tamblyn, *J. Org. Chem*. **1982**, *47*, 4059–4068.

[12] R. W. Curley. Jr, H. F. DeLuca, *J. Org. Chem*. **1984**, *49*, 1944–1946.

[13] Y. Kusuyama, Y. Ikeda, *Bull. Chem. Soc. Jpn*. **1977**, *50*, 1784–1787.

[14] S. Cai, M. Dimitroff, T. McKennon, M. Reider, L. Robarge, D. Ryckman, X. Shang, J. Therrien, *Org. Process. Rev. Dev*. **2004**, *8,* 353–359.

[15] V. A. Vu, I. Marek, K. Polborn, P. Knochel, *Angew. Chem. Int. Ed*. **2002**, *41*, 351–352.

[16] R. Csuk, M. J. Schabel, Y. von Scholz, *Tetrahedron: Asymmetry* **1996**, *7*, 3505–3512.

[17] Y. Chen, K. B. Fields, X. P. Zhang, *J. Am. Chem. Soc*. **2004**, *126*, 14718–14719.

[18] T. Niimi, T. Uchida, R. Irie, T. Katsuki, *Adv. Synth. Catal*. **2001**, *343*, 79–88.

[19] M. Davi, H. Lebel, *Chem. Commun*. **2008**, *40*, 4974–4976.

[20] L. Huang, Y. Chen, G.-Y. Gao, X. P. Zhang, *J. Org. Chem*. **2003**, *68*, 8179–8184.

[21] A. Nakamura, A. Konishi, Y. Tatsuno, S. Otsuka, *J. Am. Chem. Soc*. **1978**, *100*, 3443–3448.

[22] T. T. Raj, M. R. Eftink, *Synth. Commun*. **1998**, *28*, 3787–3794.

[23] D. A. Evans, K. A. Woerpel, M. M. Hinman, M. M. Faul, *J. Am. Chem. Soc.* **1991**, *113*, 726–728.

[24] S.-P. Xu, P.-C. Lv, L. Shi, H.-L. Zhu, *Arch. Pharm. Chem. Life. Sci*. **2010**, *343*, 282–290.

[25] Y. Jiang, L. Gong, X. Feng, W. Hu, W. Pan, Z. Li, A. Mi, *Tetrahedron*, **1997**, *53*, 14327–14338.

[26] B. Morandi, B. Mariampillai, E. M. Carreira, *Angew. Chem. Int. Ed*. **2011**, *50*, 1101–1104.

[27] T. Fukuda, T. Katsuki, *Tetrahedron*, **1997**, *53*, 7201–7208.
